# Outer‐Sphere Tuning of Synthetic Iron–Sulfur Clusters Within Nanoscale Cage Frameworks

**DOI:** 10.1002/smll.74001

**Published:** 2026-06-05

**Authors:** Rupal Baliyan, Shelby T. Davis, Jan‐Niklas Boyn, Gwendolyn A. Bailey

**Affiliations:** ^1^ Department of Chemistry University of Minnesota Twin Cities Minnesota USA

**Keywords:** bioinspired chemistry, host‐guest chemistry, hydrophobic effects, iron–sulfur clusters, metallocofactors, outer coordination sphere, X‐ray photoelectron spectroscopy

## Abstract

As atomic‐scale archetypes for multi‐electron transfer, iron–sulfur clusters are compelling candidates for the design of redox‐active, catalytic nanomaterials; yet, translating their enzymatic proficiency into robust synthetic platforms remains a challenge. A key bottleneck lies in replicating the outer‐sphere environment, which modulates the cluster's electronic landscape, provides site‐isolation, and facilitates catalytic pathways via programmable noncovalent interactions. Herein, we demonstrate a host‐guest strategy for systematically tuning iron–sulfur cofactor models within tetrahedral **[M_4_L_6_]^8+^** cages (M = Ni, Fe, Zn). Successful encapsulation of [Fe_4_S_4_(SR)_4_]^2−^ (R = Ph, ─CH_2_CH_2_OH) was confirmed by 1D/2D NMR spectroscopy and ESI mass spectrometry, while titration studies confirmed association constants (*K*_a_) > 10^7^ m ^−1^. Spectroscopic characterization by ^57^Fe Mössbauer, X‐ray photoelectron, IR, and UV–vis spectroscopy reveals significant shifts in Fe─S bond covalency and electron density that converge toward those of the native, protein‐bound cofactors. Control studies with [Fe_4_S_4_(S*^t^* Bu)_4_]^2−^, which associates with the host primarily through outer‐sphere ion pairing, demonstrate that while the electrostatic field generated by the framework's corner vertices is a primary driver of these electronic perturbations, internal confinement effects such as *π–π* stacking and hydrogen bonding provide important secondary modulations. This work establishes a foundation for utilizing cage‐stabilized cofactors in the development of sophisticated, energy‐efficient catalytic systems.

## Graphical abstract

Noncovalent encapsulation of a synthetic iron–sulfur cluster within hydrophobic, naphthalenediimide (NDI)‐paneled cages is shown to lead to dramatic changes in the electronic structure and covalency of the Fe─S core.

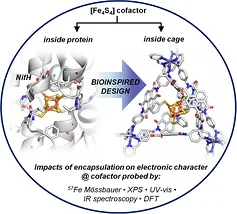

## 1 Introduction

**FIGURE 1:**
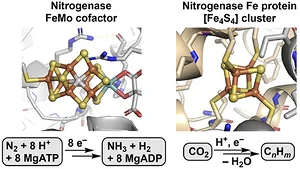
Representative iron–sulfur cofactors employed in nature as high‐efficiency redox modules for challenging multielectron, multiproton‐driven reactions.

Iron–sulfur (Fe–S) clusters are nature's premier redox‐active engines, facilitating the most energetically demanding transformations across all kingdoms of life, including nitrogen fixation, photosynthesis, and carbon dioxide metabolism [1, 2]. In enzymes such as nitrogenase and CO dehydrogenase, these clusters operate as atomic‐scale catalysts that facilitate the ATP‐driven reduction of N_2_ to ammonia, the interconversion of CO and CO_2_, and the selective C─H functionalization of metabolic substrates (Figure 1, left) [3, 4, 5, 6]. Beyond their native roles, Fe─S clusters in artificial and natural scaffolds have demonstrated remarkable proficiency in the catalytic reduction of CO_2_ and CO to value‐added hydrocarbons, including methane, ethylene, and longer alkyl chains [7, 8, 9, 10, 11, 12, 13, 14]. Despite their complexity, these reactions proceed with exceptional atom‐efficiency and low overpotential—performance metrics that remain the gold standard for functional nanomaterials. However, the fragile nature of these clusters outside their protein matrices presents a significant barrier to industrial deployment. Replicating this catalytic power in an all‐synthetic system requires the creation of hierarchical environments that can mimic the protective and modulating effects of the native protein scaffold.

To replicate these biological benchmarks, intensive research has targeted synthetic iron–sulfur clusters with core structures and primary ligand spheres that mirror their native counterparts [15, 16, 17]. These molecular models have provided important insights into bonding and intrinsic electronic structure by isolating the cofactors from their surrounding protein environments [15, 16, 17]. However, a critical limitation of these traditional models is their inability to capture the functional influence of the outer coordination sphere, although it is a decisive regulator of cofactor performance [6, 18, 19]. For example, cysteine‐supported [Fe_2_S_2_], [Fe_3_S_4_], and [Fe_4_S_4_] cofactors are known to cover an impressively large redox window from −700 to +450 mV vs. NHE, even with identical primary ligand spheres [20]. This remarkable electronic tunability is dictated in part by hydrogen bonding between the cofactor, peptide backbone, and water molecules [19], while environmental polarity and asymmetry—governed by local hydrophobicity, water accessibility, and the orientation of protein dipoles—provide a further layer of electrostatic control [21]. Notably, the outer coordination spheres can even override the influence of the primary ligands. While ligand substitutions in synthetic complexes typically cause dramatic redox potential shifts, analogous mutations in enzymatic systems (e.g., His→Cys in *C. acetobutilycum* hydrogenase) can result in negligible potential shifts [22]. This evidence suggests that the cofactor's confined outer‐sphere environment can provide a level of electronic buffering and stabilization that current synthetic platforms lack.

Beyond electronic tuning, the outer coordination sphere is essential for gating and promoting catalysis through dynamic structural responses. In the nitrogenase iron protein (NifH; Figure 1, right), the gating of electron transfer from the [Fe_4_S_4_] cofactor is characterized by discrete “ON” (electron release) and “OFF” (electron storage) states driven by external stimuli, such as nucleotide binding [23] and MoFe protein docking [24]. This switching mechanism is driven by the relative positioning of nearby α‐helix dipoles, which modulate the cofactor's redox potential by up to 320 mV via localized electrostatic field effects [25]. The sensitivity of Fe─S clusters to their local environment is further illustrated by the divergent catalytic responses of NifH from *A. vinelandii* (*Av*) and *M. acetivorans* (*Ma*; Figure 1, right) [10, 11]. Despite possessing identical [Fe_4_S_4_] inorganic cores and primary coordination environments, their catalytic profiles differ dramatically. While *Av*NifH exclusively facilitates the reduction of CO_2_ to CO, *Ma*NifH remarkably produces a mixture of C_1_–C_4_ hydrocarbons through enhanced C─C coupling. This shift in product selectivity has been correlated with a modest 94 mV difference in redox potential (−395 mV for *Ma*NifH vs. −301 mV for *Av*NifH) [11] and stronger S = 3/2 spin contribution of the reduced cluster in *Ma*NifH [13]. Additionally, local arginine residues create a functionalized nanoscale pocket that facilitates CO_2_ capture and stabilization through cooperative noncovalent interactions [13]. However, the precise mechanism linking confinement‐induced electronic shifts to the promotion of C─C coupling remains an open question in the field.

**FIGURE 2:**
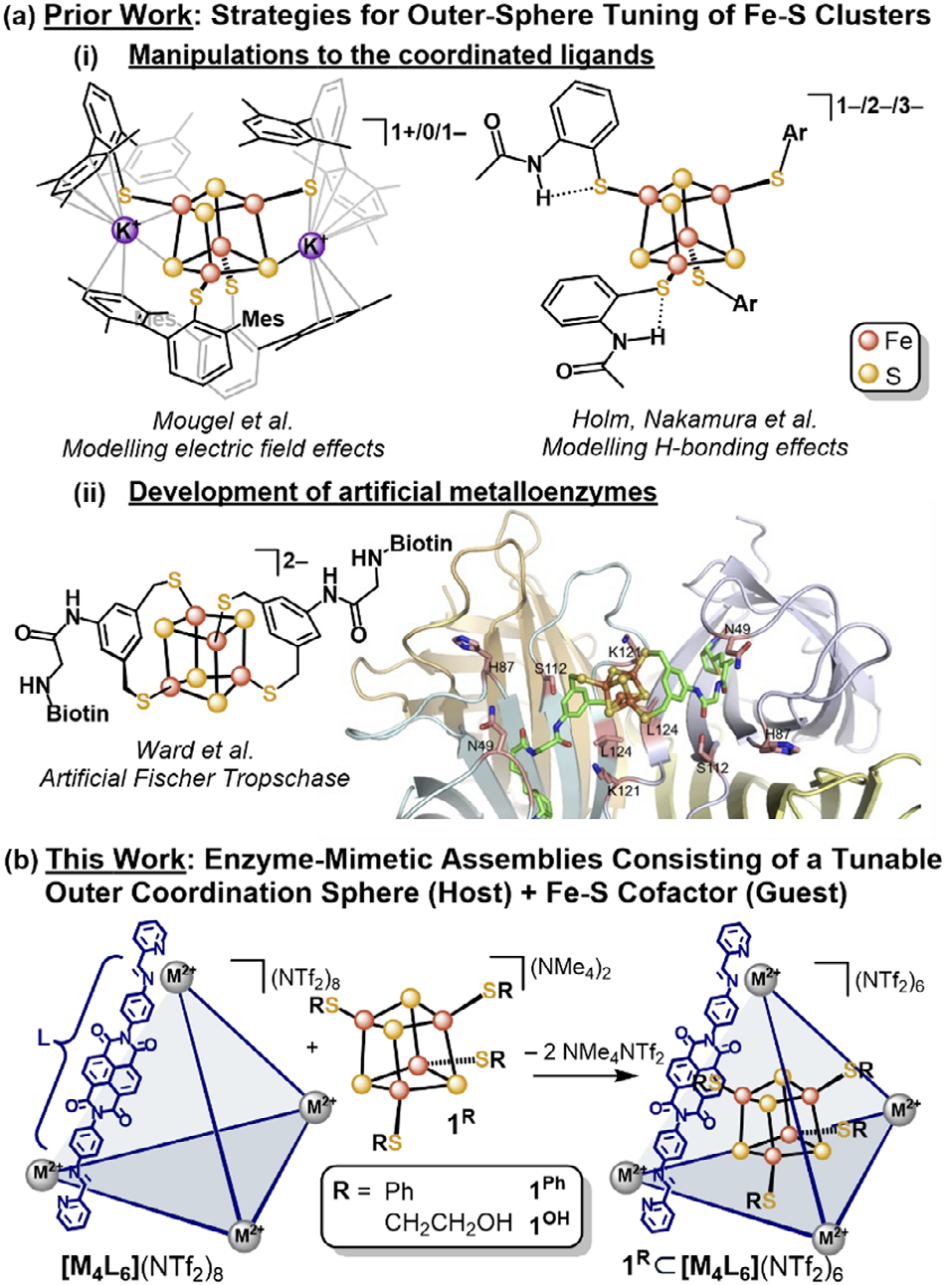
(a) Prior work: Strategies for incorporating outer coordination sphere effects in (i) synthetic models and (ii) artificial enzymes (Mes = 2,4,6‐trimethylphenyl). (b) This work: Synthesis of enzyme‐mimetic assemblies consisting of synthetic iron–sulfur cofactors encapsulated inside molecular cages.

Established strategies for integrating outer‐sphere functionality into synthetic systems have predominantly relied on covalent modification of the primary ligand shell. For example, Mougel and coworkers simulated localized electric field effects by anchoring K^+^ ions in the close periphery of [Fe_4_S_4_(SDmp)_4_] clusters via cation‐π interactions (Dmp = 2,6‐dimesitylphenyl; Figure 2a(i), left) [26, 27]. This strategically positioned charge resulted in a dramatic anodic shift of up to +1.6 V compared to model congeners with the bulkier [N*^n^* Bu_4_]^+^ counterion [27]. In other work, Holm and Nakamura utilized intramolecular hydrogen bonding networks within thiophenolate‐supported [Fe_4_S_4_(SAr_4_)]^2−^ clusters. By functionalizing the ligands with *o*‐hydroxy and *o*‐amide groups, they achieved modular anodic shifts of ∼170 mV per intraligand OH/NH—S interaction, with systematic blueshifts in the S→Fe LMCT transitions indicating a reduction in sulfur electron density (Figure 2a(i), right) [28, 29]. Similar effects were induced in [Fe_4_S_4_(SR)_4_]^2−^ cofactor models functionalized with the dipeptide chains SR = Cys‐Gly‐NH(*p*‐C_6_H_4_X), where the redox potentials correlated strongly with the Hammett σ_p_ parameters of the aromatic *p*‐X group consistent with NH—S hydrogen bonding [30].

Besides discrete molecular models, there is burgeoning interest in biomimetic scaffolds that integrate both primary and secondary spheres into functional, hierarchical architectures. In a pioneering study, Ward and coworkers engineered an artificial metalloenzyme by anchoring a biotin‐functionalized [Fe_4_S_4_(SR)_4_]^2−^ cluster within the cavity of streptavidin (Figure 2a(ii)) [31]. The bio‐hybrid system exhibited significantly enhanced kinetics for CO_2_ reduction compared to the isolated cluster—an effect that was attributed to hydrophobic confinement inside the protein matrix as evidenced by the ∼500 mV anodic shift in the [Fe_4_S_4_(SR)_4_]^3−/4−^ couple upon protein binding. Similarly, Shafaat and coworkers successfully incorporated a synthetic [NiFe_3_S_4_] cluster into the *Pyrococcus furiosus* ferredoxin framework, showing that the embedded cluster was capable of reversible electron transfer and selective CO binding at the Ni site [32, 33]. While these protein‐based scaffolds provide excellent outer‐sphere control, they are inherently limited by their biological complexity, which makes systematic tuning challenging. These limitations have catalyzed the search for fully synthetic, programmable host environments—such as supramolecular nanocages—that can replicate these “matrix effects” with greater precision and structural versatility.

Still, bridging the gap between simple molecular models and complex bio‐hybrids remains a formidable challenge. Primary coordination sphere manipulation is inherently limited by ligand design, a time‐intensive process that lacks the many degrees of freedom afforded by the native protein scaffold. By contrast, metalloenzyme development affords theoretically infinite degrees of freedom but is experimentally non‐trivial and fails to capture the structural simplicity of synthetic cofactor models. In light of these limitations, we hypothesized that the encapsulation of synthetic iron–sulfur clusters within self‐assembled nanocages would provide a tunable platform that would replicate the protein's protective and modulatory functions (Figure 2b). This approach would combine the benefits of nanoscale site‐isolation, which protects the cofactor from decomposition by redox‐induced core interconversion [34], with the ability to precisely and systematically engineer the local environment through the choice of cage panels and cluster ligands.

Despite the potential of this strategy, reports of cage‐encapsulated iron–sulfur clusters remain remarkably scarce. Beyond early examples of [Fe_4_S_4_(SR)_4_]^2−^ clusters encapsulated within synthetic macrocycles [35, 36], the most relevant precedents are [FeFe]‐hydrogenase mimics in which encapsulation of the diiron cofactor was achieved in open cyclodextrin cavities via hydrophobic and dipole/hydrogen bonding interactions [37, 38], or via covalent tethering in the closed cage capsules [39, 40, 41]. In the latter systems, cluster encapsulation led to a decrease in catalytic overpotential by up to 250 mV—a shift attributed to charge donation via the covalent tethers and electrostatic stabilization of anionic intermediates by the charged cage framework [39, 40, 41]. Installed endohedral ammonium groups further enhanced catalytic rates by facilitating substrate delivery inside the cage [41]. In order to expand these precedents to iron–sulfur cofactor assemblies, there is need to develop noncovalent cofactor encapsulation strategies that are both robust and generalizable. This approach would facilitate the independent tuning of the cluster, its ligands, and the surrounding cage framework, enabling the systematic elucidation of structure‐property relationships within a precisely defined supramolecular pocket.

Herein, we report a robust and generalizable strategy for the construction of biomimetic assemblies consisting of synthetic iron–sulfur cofactor models [Fe_4_S_4_(SR)_4_]^2−^ **[1^R^]^2^** ^**−**^ noncovalently encapsulated inside self‐assembled molecular cages (Figure 2b). By employing tetrahedral **[M_4_L_6_]^8+^** cages (M = Fe, Ni, Zn) functionalized with naphthalenediimide (NDI) panels, [42, 43] we generate a precisely defined, electrostatically and size‐matched environment for cofactor guests **[1^R^]^2^** ^**−**^, where R = Ph (**[1^Ph^]^2^** ^**−**^) and ‐CH_2_CH_2_OH (**[1^OH^]^2^** ^**−**^). The M^2+^ ions at the cationic cage vertices provide the charge complementarity requisite for favorable guest association, while the NDI panels provide the spatial confinement, hydrophobic interactions, and noncovalent interactions characteristic of an enzyme‐like microenvironment [18, 19, 44]. Simultaneously, these panels act as an electroactive reservoir, mimicking the redox‐relay systems found in natural electron‐transport chains. The NDI group can be easily replaced with a range of other units, offering systematic control over the outer coordination sphere [45, 46]. The influence of the nanoscale environment on the cluster's electronic structure was interrogated by X‐ray photoelectron spectroscopy (XPS), ^57^Fe Mössbauer spectroscopy, and UV–vis/IR analysis. These techniques reveal a significant increase in Fe─S bond covalency and net electron withdrawal from the Fe centers. Control experiments reveal that while the electrostatic field generated by the cationic vertices is a primary driver of these perturbations, specific noncovalent interactions, including *π–π* stacking and hydrogen bonding contribute substantially to the observed electronic and redox modulations. Combined, these studies demonstrate that supramolecular encapsulation is a powerful means of integrating protein‐like outer‐sphere interactions to regulate the electronic structure and functional potential of iron–sulfur clusters through precisely tuned noncovalent environments.

## 2 Results and Discussion

### 2.1 Cofactor Encapsulation

A defining feature of iron–sulfur clusters, when supported by biologically relevant thiolate ligand spheres, is their anionic character (−1 to −4, depending on the cluster charge state). We envisioned that this inherent negative charge—particularly pronounced in their highly reduced, catalytically active [Fe_4_S_4_]^1+/0^ states—could be leveraged to drive robust host‐guest encapsulation through complementary electrostatic interactions with cationic cage frameworks. This strategy represents a conceptual inversion of the seminal work by Raymond, Bergman, Toste, and others, who utilized anionic **[M_4_L_6_]^12^** ^**−**^ tetrahedral cages to sequester cationic transition metal catalysts for applications such as C─H functionalization and alkyl‐alkyl cross‐coupling [47, 48, 49, 50, 51, 52]. We initially targeted [Fe_4_S_4_(SR)_4_]^2−^ clusters **[1^Ph^]^2^** ^**−**^ and **[1^OH^]^2^** ^**−**^, anticipating that the aromatic and hydroxyl functionalities would strengthen the binding affinity to functionalized hosts via *π–π* stacking interactions and hydrogen bonding. In addition, we employed **[1^t^ ^Bu^]^2^** ^**−**^ as a control to isolate the specific influence of these noncovalent interactions. These clusters are ideal candidates for initial studies due to their physiological relevance, synthetic accessibility, and versatility as platforms for multielectron‐driven catalysis [11, 53, 54].

To facilitate encapsulation, we selected tetrahedral **[M**_**4**_ **L**_**6**_ **]** ^**8+**^ nanocages (M = Fe [42], Zn [43], and Ni) featuring NDI‐functionalized linkers (L). These hosts were selected for their well‐defined internal cavities, which are size‐matched to the [Fe_4_S_4_] dianions. For example, **[** **1** ^**Ph**^ **]** ^**2−**^ in **[** **Zn**_**4**_ **L**_**6**_ **]** ^**8+**^ exhibits a packing coefficient of 52%, aligning closely with the 55% rule established by Rebek for optimal occupancy (Figure 2b and Table 1) [55, 56]. Meanwhile, the volume of hydroxyl‐functionalized cluster **[** **1** ^**OH**^ **]** ^**2−**^ falls slightly below this idealized threshold, providing a unique opportunity to deconvolute size effects from second‐sphere hydrogen‐bonding interactions in driving assembly. We anticipated that these noncovalent interactions would be further optimized by the conformational plasticity of the NDI panels and guest‐induced diastereomeric reconfiguration of the [M_4_L_6_]^8+^ cage framework—a dynamic adaptive response previously observed in related aromatic‐paneled systems [57].

**TABLE 1:** Volumes and packing coefficients for cluster dianions **[** **1** ^**R**^ **]^2^** ^**−**^ inside **[** **Zn**_**4**_ **L**_**6**_ **]** ^**8+**^.^a^

	**[** **1** ^**Ph**^ **]** ^**2−**^	**[** **1** ^**OH**^ **]** ^**2−**^	**[** **1** ^**tBu**^ **]** ^**2−**^
Volume	574 Å^3^	432 Å^3^	529 Å^3^
Packing coefficient^b^	52%	39%	47%

^a^ Cavity and guest volumes calculated from the crystallographic structures using MoloVol [55].

^b^ Packing coefficient = (molecular volume of guest) ÷ (cavity volume of host) x 100% using a calculated host volume of 1113 Å^3^ for **[** **Zn**_**4**_ **L**_**6**_ **]** ^**8+**^. For details, see Section S2.

**FIGURE 3:**
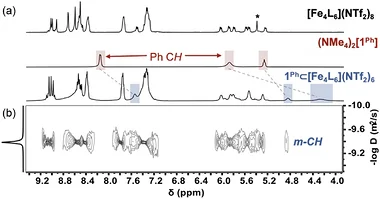
(a) ^1^H NMR spectra (500 MHz, CD_3_CN) of the free cage **[Fe_4_L_6_]**(NTf_2_)_8_, free cluster (NMe_4_)_2_ **[1^Ph^]**, and host‐guest complex **1^Ph^⊂[Fe_4_L_6_]**(NTf_2_)_6_ (^*^residual CH_2_Cl_2_). Dashed lines highlight upfield shifts in thiolate Ph C*H* resonances on encapsulation. (b) ^1^H DOSY NMR spectrum of **1^Ph^⊂[Fe_4_L_6_]**(NTf_2_)_6_ (500 MHz, CD_3_CN) showing a diffusion coefficient of 4.2 × 10^−10^ m^2^/s corresponding to a hydrodynamic radius of r_H_ = 15.6 Å according to the Stokes‐Einstein equation; for details, see the Section S4. Spectra for **1^OH^⊂[Zn_4_L_6_]**(NTf_2_)_6_ and **[Fe_4_L_6_]**[**1^tBu^]**(OTf)_6_ are presented in Figures S3, S4, and S23–S27.

Thus, host‐guest complexes **1^R^⊂[M_4_L_6_]**(NTf_2_)_6_ (M = Fe, Ni, Zn) were synthesized by mixing clusters (NMe_4_)_2_ **[1^R^]** with cages **[M_4_L_6_]**(NTf_2_)_8_ in equimolar amounts at ambient temperature in CD_3_CN (Figure 2b). On mixing, instantaneous color changes from maroon (**[1^Ph^]^2^** ^**−**^) or green‐brown (**[1^OH^]^2^** ^**−**^) to vibrant magenta or brown were observed. Immediate in situ ^1^H NMR analysis revealed broadening of the host framework signals together with significant upfield shifts in the cluster thiolate resonances (Δδ_H_ 0.4–12.3 ppm), characteristic of fast exchange kinetics on the NMR timescale (Figure 3a; Figure S3) [50, 58]. The upfield shifting of cluster thiolate resonances is attributed to magnetic shielding effects arising from their close proximity to the interior aromatic ring current, which is not expected for loosely associated ion pairs [50]. Notably, even for the smallest guest [1^OH^]^2−^, significant shielding was observed (e.g., δ_H_ 3.57 ppm for SC*H*_2_ in **1^OH^⊂[M_4_L_6_]**(NTf_2_)_6_, vs. 12.3 ppm for free [1^OH^]^2−^). To quantify the thermodynamic driving force for assembly, high‐field NMR titration studies were performed [59], revealing exceptionally high association constants with lower bounds of *K*_a_ = 4.8 × 10^7^ and 1.9 × 10^8^ m ^−1^ for **[1^Ph^]^2^** ^**−**^ and **[1^OH^]^2^** ^**−**^, respectively. These values correspond to a Gibbs free energy of dissociation (ΔG) ≤ 11.3 kcal/mol, indicating that while cluster release is likely energetically accessible under the reaction conditions, the equilibrium heavily favors the assembled state, with only a very small fraction existing as the free cluster at any given time. The structural integrity of the assemblies was further interrogated by ^1^H DOSY NMR analysis, which revealed slightly larger diffusion coefficients—and consequently smaller hydrodynamic radii (*r*_H_)—for the host‐guest complexes relative to the free hosts (e.g., **1^Ph^⊂[Fe_4_L_6_]**(NTf_2_)_6_ r_H_ = 15.6 Å, vs. **[Fe_4_L_6_]**(NTf_2_)_8_ r_H_ = 21.5 Å**;** Figure 3b and Figures S16 and S17). This hydrodynamic contraction is attributed to guest‐induced conformational tightening and/or the elimination of counterions. While ^1^H DOSY NMR signals for the guest are often not observable due to peak broadening [39, 60, 61], *m*‐C*H* signals were observed for **1^Ph^⊂[Fe_4_L_6_]**(NTf_2_)_6_ at an identical diffusion coefficient to the host, confirming that the thermodynamics of host‐guest binding are strong.

To isolate the specific contributions of second‐sphere noncovalent forces in the assembly process, we investigated the encapsulation of the *tert*‐butyl functionalized cluster, **[1^tBu^]^2^** ^**−**^. In contrast to the **[1^Ph^]^2^** ^**−**^ and **[1^OH^]^2^** ^**−**^ analogs, the addition of **[1^tBu^]^2^** ^**−**^ to solutions of **[M_4_L_6_]**(X)_8_ (M = Ni, Fe, X = NTf_2_, OTf) resulted in negligible ^1^H NMR perturbations, with shifts of only ∼0.05 ppm observed for the *^t^* Bu resonance. Meanwhile, the use of **[Zn_4_L_6_]**(NTf_2_)_8_ led to immediate precipitation of an insoluble solid, precluding further characterization and analysis. While a slight broadening of the host framework signals was observed, the lack of significant shielding suggests a regime of weak, non‐specific association and rapid guest exchange rather than robust encapsulation [58, 62]. Subsequent high‐field ^1^H NMR titrations further confirmed this observation, with no detectable change in the *^t^* Bu resonance even in the presence of a twenty‐fold molar excess of the host. Interestingly, ^1^H DOSY NMR analysis again revealed a significantly contracted hydrodynamic radius (*r*_H_ = 17.5 Å, vs. 21.5 Å for the parent cage), suggesting that counterion displacement leads to a more hydrodynamically streamlined ion‐paired assembly. These data underscore that electrostatic complementarity alone is insufficient to drive high‐affinity encapsulation within these frameworks. Instead, the negligible binding affinity of **[1^tBu^]^2^** ^**−**^ relative to **[1^OH^]^2^** ^**−**^ and **[1^Ph^]^2^** ^**−**^ demonstrates that specific, higher‐order interfacial contacts such as H‐bonding and *π–π* stacking are required.

**FIGURE 4:**
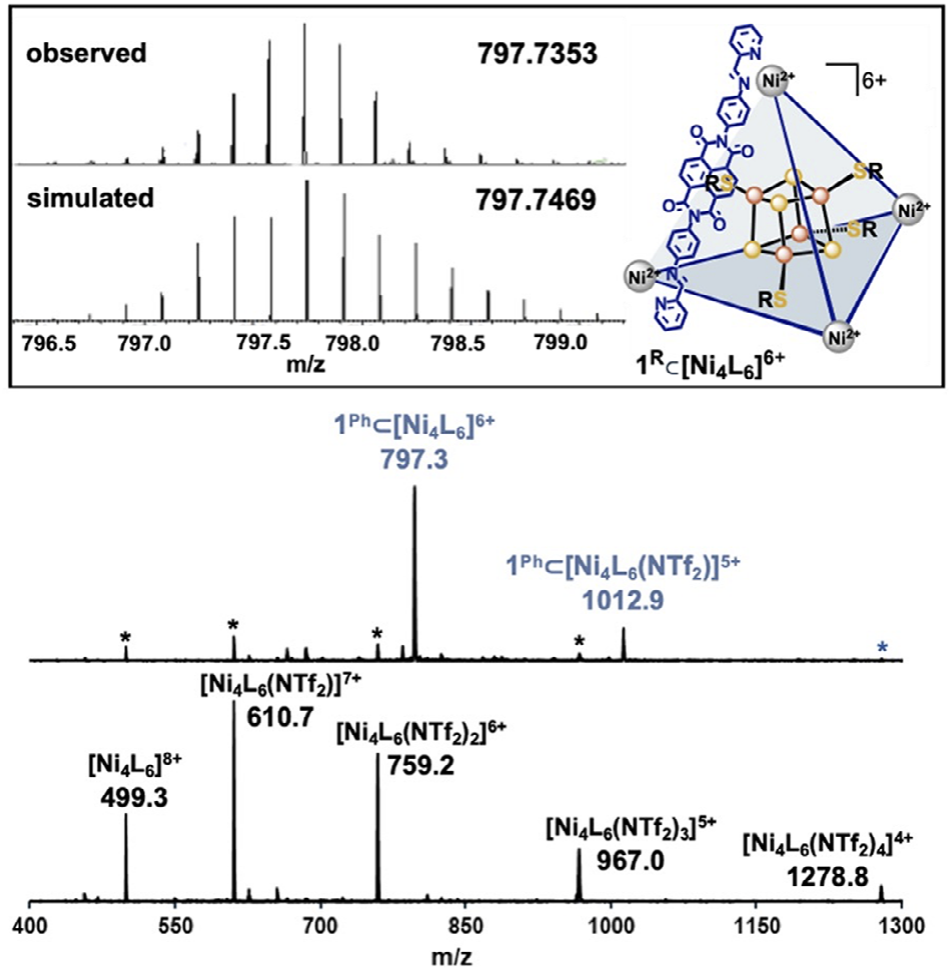
ESI mass spectra of **1^Ph^⊂[Ni_4_L_6_]**(NTf_2_)_6_ (top) and empty cage **[Ni_4_L_6_]**(NTf_2_)_8_ (bottom; both BioTOF II ESI‐MS; ^*^denotes peaks assigned to empty cage). Inset: high‐resolution ESI mass spectrum of the **1^Ph^⊂[Ni_4_L_6_]^6+^** peak showing that the isotopic distribution closely matches the simulated pattern (Orbitrap IQ‐X Tribrid MS). For the ESI mass spectra of **1^R^⊂[Fe_4_L_6_]**(NTf_2_)_6_ (R = Ph, OH) and **[Fe_4_L_6_][1^tBu^]**(NTf_2_)_6_, see Section S5.

The structural integrity of the host‐guest assemblies was further corroborated by high‐resolution electrospray ionization–mass spectrometry (ESI‐MS). In these systems, the observation of intact host‐guest complexes under ESI conditions serves as a robust indicator of kinetic stability, since host‐guest dissociation is typically driven by the rate of guest exchange rather than equilibrium thermodynamics alone [58]. For the phenyl‐functionalized system, an intense signal was observed for the intact hexacationic complex **1^Ph^⊂[Ni_4_L_6_]^6+^** together with smaller peaks for the free cage (Figure 4; Figure S29; similar behavior was observed for **1^Ph^⊂[Fe_4_L_6_]**(NTf_2_)_6_ in Figure S32). The isotopic distribution seen in the high‐resolution Orbitrap mass spectrum of **1^Ph^⊂[Ni_4_L_6_]**(NTf_2_)_6_ closely matches the simulated pattern (Figure 4, inset). By comparison, low‐resolution spectra of **1^OH^⊂[Fe_4_L_6_]**(NTf_2_)_6_ exclusively showed signals for the dissociated cage (Figure S33), while the high‐resolution measurements revealed the presence of intact **1^OH^⊂[Fe_4_L_6_]^6+^** (Figure S34). These results indicate that there is a clear spectrum of guest exchange rates governed by the interplay of steric occupancy and interfacial forces. Guest exchange is slowest for **1^Ph^⊂[Fe_4_L_6_]^6+^** containing the largest guest (52% occupancy; Table 1) [56]—likely stabilized by the synergistic effects of phenyl/NDI π–π stacking interactions. Conversely, the accelerated exchange observed for the hydroxyl‐functionalized cluster **[1^OH^]^2^** ^**−**^ (39% occupancy) suggests that its smaller steric footprint significantly lowers the kinetic barrier for guest egress, even though the thermodynamics of binding are strong [62]. Finally, only a small signal for **[Fe_4_L_6_][1^tBu^]^6+^** was observed in the low‐resolution mass spectra, while no matching signal was observed in the high‐resolution spectrum, consistent with the weakly associated nature of the ion pair (Figure S36).

### 2.2 Computational Structures

**FIGURE 5:**
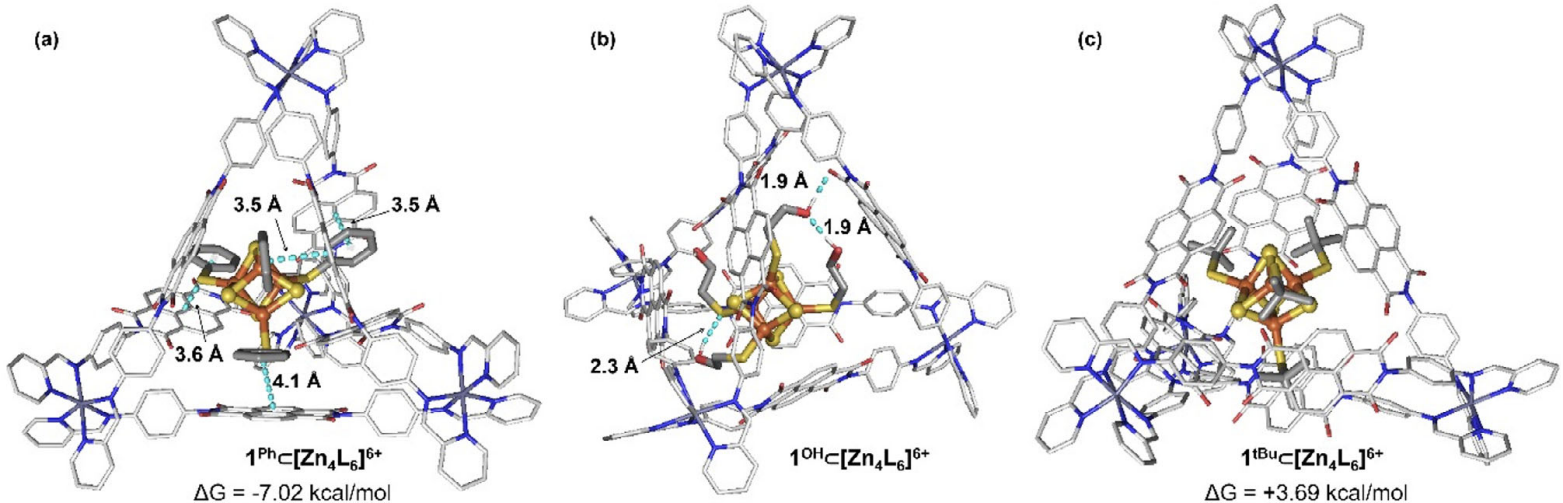
DFT‐optimized structures of the host‐guest assemblies **1^Ph^⊂[Zn_4_L_6_]^6+^** and **1^OH^⊂[Zn_4_L_6_]^6+^**, alongside the hypothetical **1^tBu^⊂[Zn_4_L_6_]^6+^** complex. Level of theory: TPSS/D3BJ/def2‐SVP for geometry optimizations and TPSS/D3BJ/MINIX for single‐point and frequency calculations. These calculations revealed Gibbs free energies of −7.0 kcal/mol for encapsulation of **[1^Ph^]^2^** ^**−**^ and +3.7 kcal/mol for encapsulation of **[1^tBu^]^2^** ^**−**^. See Section S12 for details.

To elucidate the specific noncovalent driving forces that govern high‐affinity encapsulation, unrestricted Kohn‐Sham density functional theory (DFT) was employed to simulate the structural landscape of **1^Ph^⊂[Zn_4_L_6_]^6+^** and **1^OH^⊂[Zn_4_L_6_]^6+^**, alongside the hypothetical **1^tBu^⊂[Zn_4_L_6_]^6+^** complex. Starting geometries were derived from the crystallographic coordinates of the **[1^R^]^2^** ^−^ clusters [63, 64, 65] and the **[Zn_4_L_6_]^8+^** framework (Figure 5) [43]. Optimizations were performed using the meta‐GGA TPSS [66] functional with the def2‐SVP [67] basis set, integrated with Grimme's D3 dispersion corrections and Becke‐Johnson damping [68] to accurately capture the long‐range van der Waals interactions critical to host‐guest complex stability. Vibrational frequency calculations were subsequently conducted at the TPSS‐D3BJ/MINIX [69] level of theory to confirm the nature of the stationary points.

The optimized structure of **1^Ph^⊂[Zn_4_L_6_]^6+^** revealed a highly organized interfacial architecture, where all four thiophenolate ligands are perfectly oriented to engage in *π–π* stacking with the NDI panels. These interactions are characterized by centroid–centroid distances of 3.5–4.1 Å, indicating a tight packing within the aromatic framework (blue dashes, Figure 5). In contrast, the structural model of **1^OH^⊂[Zn_4_L_6_]^6+^** revealed a more complex hydrogen‐bonding manifold. In this framework, one hydroxyl group acts as a simultaneous H‐bond donor to an NDI carbonyl and an acceptor to a neighboring hydroxyl (both 1.9 Å, blue dashes, Figure 5), while another anchors to an adjacent thiolate sulfur (2.3 Å). These findings demonstrate a clear bifurcation in supramolecular driving forces: while the sequestration of **[1^Ph^]^2^** ^**−**^ is governed by dispersive π‐π interactions within a sterically congested environment, the encapsulation of **[1^OH^]^2^** ^**−**^ is driven by a cooperative effect where the four hydroxyl groups engage in a fluxional network of interactions with the 24 internal carbonyl sites of the cage, effectively stabilizing the host‐guest complex despite the cluster's smaller steric footprint. These two mechanisms of cooperative anchoring present powerful design principles for the high‐affinity sequestration of iron–sulfur cofactors within synthetic nanoreactors.

In stark contrast to the anchored analogs, the *tert*‐butyl groups of **[1^tBu^]^2^** ^**−**^ exhibited no appreciable affinity for the NDI panels. Instead, the computational model revealed that the bulky *^t^* Bu groups protrude through the cage apertures, reflecting a lack of stabilizing interfacial contact. This structural mismatch was further underscored by Gibbs free energy calculations: while the formation of **1^Ph^⊂[Zn_4_L_6_]^6+^** is exergonic (−7.0 kcal/mol), encapsulation of **[1^tBu^]^2^** ^**−**^ is energetically disfavored by +3.7 kcal/mol. Meanwhile, reliable free energy values for **1^OH^⊂[Zn_4_L_6_]^6+^** remained elusive, likely due to the high‐dimensional conformational landscape and the fluxionality of the hydrogen bonding ensemble. The complexity of these dynamic host–guest interactions warrants a more granular computational investigation, which will be the subject of a forthcoming report.

### 2.3 Redox Properties

**FIGURE 6:**
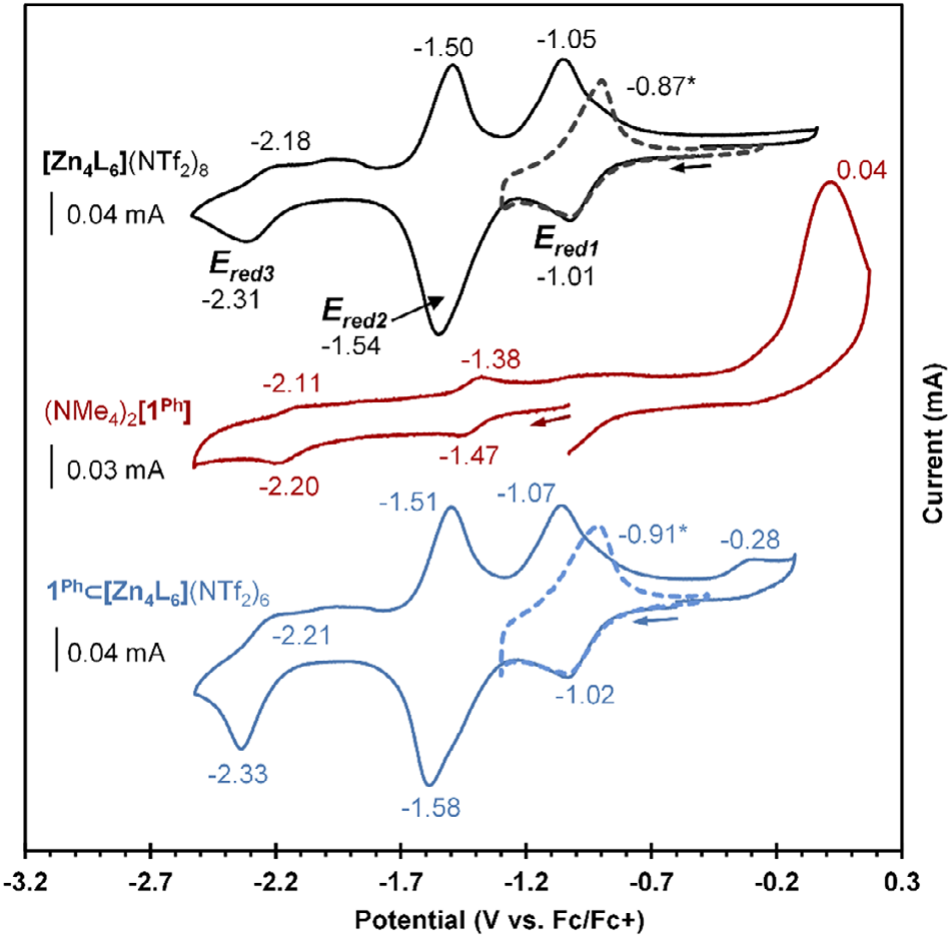
Cyclic voltammograms of **[Zn_4_L_6_]**(NTf_2_)_8_, (NMe_4_)_2_ **[1^Ph^]**, and **1^Ph^⊂[Zn_4_L_6_]**(NTf_2_)_6_. Conditions: 0.1 mm in MeCN; 0.1 m NBu_4_PF_6_; 2 V/s. Scans were conducted starting from the measured open circuit potential of each species in the direction indicated by the arrows. The first redox event for **[Zn_4_L_6_]**(NTf_2_)_8_ and **1^Ph^⊂[Zn_4_L_6_]**(NTf_2_)_6_ is isolated and overlaid on the full‐scan CV as dashed lines; the corresponding oxidation peaks are marked with asterisks. For wider scan windows, see Figure S39.

To characterize the electronic landscape of the frameworks and their corresponding host‐guest assemblies, comprehensive cyclic voltammetry (CV) studies were performed. These experiments were conducted at reduced analyte concentrations (down to 0.1 mm) to mitigate issues of precipitation associated with highly charged ionic cages [70]. Using **[Zn_4_L_6_]^8+^** as a representative for the cage series, the CV collected at 2 V/s revealed three prominent reduction features centered at ca. −1.01, −1.54, and −2.31 V vs. Fc^0/+^ (denoted *E*_red1_, *E*_red2_, and *E*_red3_, respectively: Figure 6). Scanning at slower rates down to 100 mV/s enabled resolution of *E*_red2_ as two distinct events located at −1.35 and −1.54 V vs. Fc^0/+^ (*E*_red2a_ and *E*_red2b_, respectively; Figure S49). Supporting the assignment of *E*_red2_ as two distinct events, the resolution of *E*_red2a_ and *E*_red2b_ was even more distinct in **[Fe_4_L_6_]^8+^** and **[Ni_4_L_6_]^8+^** (Figure S37). By comparison to the CV of the *N, N’*‐(*p*‐aminophenyl)naphthalenediimide precursor (Figure S43), *E*_red1_ and *E*_red2a_ are assigned to the sequential first and second reduction events of the NDI panels, [NDI]^0/−^ and [NDI]^1−/2−^, respectively [71]. Meanwhile, based on previous benchmarks for [M(iminopyridine)_n_]^2+^ and [M(bipyridine)_3_]^2+^ complexes (*n* = 2, 3; M = Zn, Fe, Ni) [72, 73, 74, 75, 76]. *E*_red2b_ and *E*_red3_ are assigned to the ligand‐based reductions of the iminopyridine‐ligated corner nodes.

A Randles‐Sevčik analysis revealed a linear dependence of peak current on the square root of scan rate ($\sqrt{\nu }$) for *E*_red1_ and *E*_red2_, consistent with both of these reduction events being diffusion‐controlled, solution‐based processes [40]. Quantitative analysis yielded an estimated electron transfer of *n* = 6 for *E*_red1_ and *n* = 11 for *E*_red2_ (Section S6). These data indicate that all six panels undergo a simultaneous one‐electron reduction under *E*_red1_, while *E*_red2_ likely involves the concurrent reduction of the six panels to the dianionic state alongside the reduction of the four corner nodes. In contrast, the peak current for *E*_red3_ exhibited a non‐linear dependence on $\sqrt{\nu }$, suggesting the onset of adsorptive processes or competing precipitation upon high‐level reduction (Figure S51). Meanwhile, the clear irreversibility of *E*_red3_ accounts for the observed asymmetry in the redox waves and the apparent shift in peak potentials (e.g., *E*_red1,c_ = −1.01 V vs. *E*_red1, a_ = −1.05 V) when scanning the full window. These features are indicative of a chemical‐electrochemical (EC) mechanism, wherein the highly reduced state of the [M_4_L_6_] framework undergoes structural rearrangement or degradation. Narrowing the scan window to isolate *E*_red1_ confirms that the couple remains reversible provided the onset of higher‐order reductions is avoided (Figure 6, dashed lines; Figures S40 and S41). These results mirror the behavior of a perylenebisimide (PBI)‐paneled cage with [Fe(bpy)_3_]^2+^ nodes, which exhibited redox features assigned to [PBI]^0/−^ and [PBI]^1−/2−^ at –0.94 at −1.11 V vs. Fc^0/+^ followed by three bipyridine‐centered reductions at −1.65, −1.85, and −2.04 V vs. Fc^0/+^ [77]. Similar features were observed in the CV of cages **[Fe_4_L_6_]^8+^** and **[Ni_4_L_6_]^8+^**, with only minor shifts in the redox potentials for the NDI panels and slightly larger shifts in those of the corners (Figure S37).

The electrochemical response of the host‐guest assemblies was evaluated using **1^Ph^⊂[M_4_L_6_]^6+^** as a structural archetype. Thus, **1^Ph^⊂[Zn_4_L_6_]^6+^** exhibited only minor cathodic shifts (10–40 mV) in the framework‐centered redox features, accompanied by a slight increase in peak current density consistent with the increased diffusion coefficients measured via ^1^H DOSY of the host‐guest assemblies. In turn, the reduction features of sequestered **[1^Ph^]^2^** ^**−**^ were either obscured by the high‐density frameworks or suppressed. This attenuation of guest reduction could be due to the Frumkin effect, wherein the organized layer of NDI^•−^ at the electrode interface creates a localized electrostatic barrier, hindering electron flux to the internal cavity [78]. Furthermore, the significant hydrodynamic volume of the supramolecular assembly likely imposes a mass transport penalty, further reducing the observable signal intensity of the guest. In contrast to the reduction behavior, a discrete, irreversible oxidation peak assigned to the encapsulated cofactor was resolved at −0.28 V vs Fc^0/+^. This represents a cathodic shift of ca. 320 mV relative to the free cluster—a perturbation of comparable magnitude to the ON/OFF redox trigger of NifH (see Introduction) driven by large‐scale conformational changes and the relative positioning of α‐helix dipoles [25]. The magnitude of this shift is also comparable to the redox divergence of ferredoxins vs. high‐potential iron proteins (HiPIPs), which is dictated largely by the extent of [Fe_4_S_4_] cofactor solvation [79]. In the present system, the 320 mV shift is likely indicative of the low‐dielectric, hydrophobic nanoenvironment provided by the cage cavity, which destabilizes the anionic charge states relative to the bulk polar solvent—in line with the electronic shielding reported for Fe‐S clusters in dendrimeric capsules and other enclosed environments [80, 81]. Overall, similar effects were observed for **1^Ph^⊂[M_4_L_6_]^6+^** (M = Fe, Ni; Figures S47–S55), indicating that these trends are generalizable to other corner metals [82].

### 2.4 Impact of Encapsulation on Electronic Structure and Bonding

Having quantified the host‐guest dynamics, we next turned to XPS, ^57^Fe Mössbauer, UV–vis, and IR spectroscopy to probe the electronic and bonding perturbations induced by the cage environment. These experiments focused on the Zn^2+^ cage derivatives (or Ni^2+^ for **[1^tBu^]^2^** ^**−**^, for which use of Zn^2+^ was precluded by insolubility) to avoid interfering signals from the cage corner metal centers. For these studies, solid samples of **1^R^⊂[M_4_L_6_]**(NTf_2_)_6_ were prepared via slow crystallization from MeCN/ether at 0 °C. Importantly, the unencapsulated assembly **[Ni_4_L_6_][1^tBu^]**(NTf_2_)_6_ was prepared under identical conditions to serve as a non‐encapsulated ion‐paired control, allowing for the deconvolution of external electrostatic effects—stemming from the cationic metal corners—from the internal confinement effects arising from H‐bonding and *π–π* stacking. While we cannot exclude the possibility that **[1^tBu^]^2^** ^**−**^ enters the cage upon crystallization, this species nonetheless provides an important baseline since any noncovalent effects arising from the *^t^* Bu group are expected to be very weak.

**FIGURE 7:**
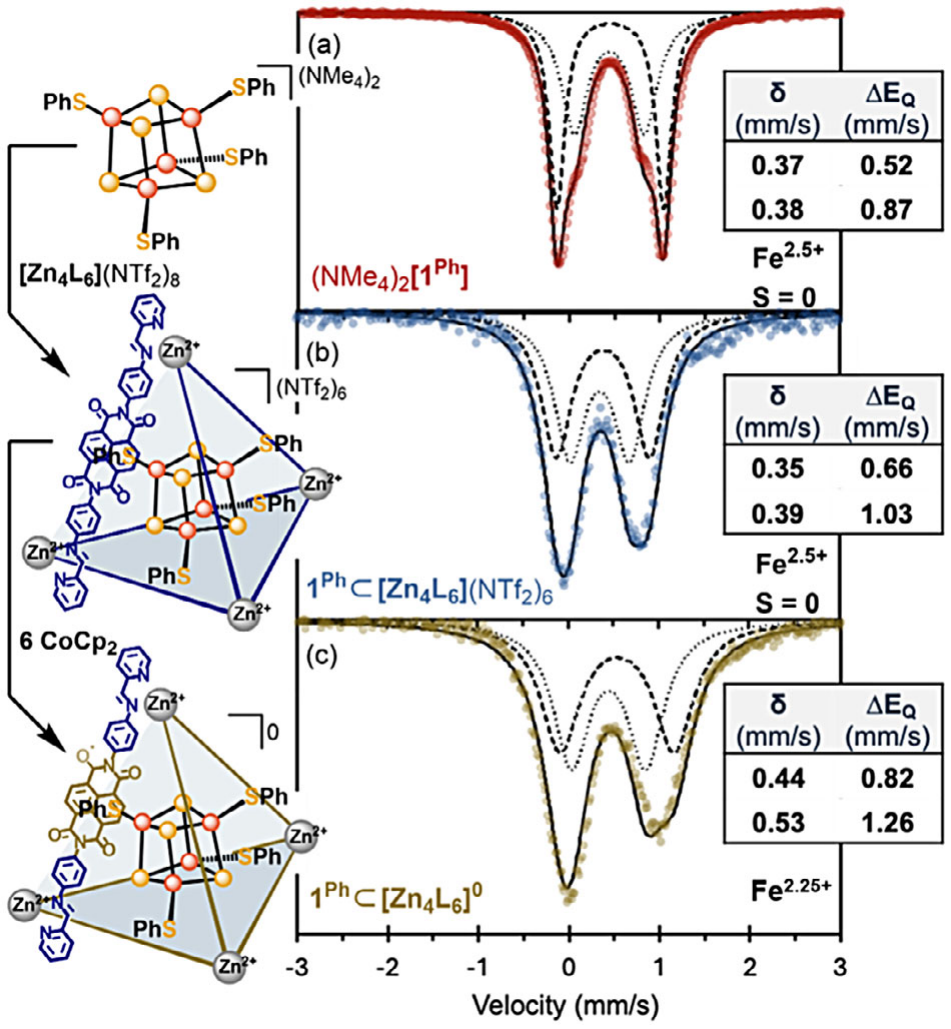
Experimental (colored dots) and simulated (black lines) ^57^Fe Mössbauer spectra of (NMe_4_)_2_ **[1^Ph^]**, **1^Ph^⊂[Zn_4_L_6_]**(NTf_2_)_6_, and the reduced complex **1^Ph^⊂[Zn_4_L_6_]** recorded at 200 K and zero magnetic field. Simulated doublets for inequivalent Fe_2_S_2_ subsites are shown with dashed and dotted lines. [Correction added on August 11, 2026, after first online publication: The oxidation state in Figure 7c was incorrectly given as Fe^2.5+^ and has been corrected to Fe^2.25+^ in this version.]

The oxidation states and structural integrity of the clusters was confirmed by ^57^Fe Mössbauer spectroscopy. Thus, the 70 K spectrum of encapsulated **1^Ph^⊂[Ni_4_L_6_]**(NTf_2_)_6_ was satisfactorily fit using two quadrupole doublets of equal populations (δ = 0.46 and 0.47 mm/s), mirroring the profile of the free cluster (NMe_4_)_2_ **[1^Ph^]** and consistent with a singlet (S = 0) ground state (Figures S62 and S63; Table 2) [83]. This two‐doublet pattern reflects the characteristic electronic structure of the [Fe_4_S_4_]^2+^ core, comprising two inequivalent mixed‐valent, antiferromagnetically coupled [Fe^2.5+^_2_S_2_]^+^ dimers [84, 85]. At 200 K, the isomer shifts of **1^Ph^⊂[Zn_4_L_6_]**(NTf_2_)_6_ and (NMe_4_)_2_ **[1^Ph^]** appeared slightly lower (e.g., **1^Ph^⊂[Zn_4_L_6_]**(NTf_2_)_6_: δ = 0.35 and 0.39 mm/s), reflecting the thermal population of excited states (Figure 7a,b and Table 2) [83]. The negligible change in isomer shifts between the free cluster and the host‐guest complex may indicate that the *σ*‐orbital electron density at the iron nuclei remains unperturbed; alternatively, it could reflect competing effects where a change in electron density is accompanied by a shift in Fe─S covalency. Meanwhile, the isomer shifts and two‐doublet patterns of **1^OH^⊂[Zn_4_L_6_]**(NTf_2_)_6_ and **[Ni_4_L_6_][1^tBu^]**(NTf_2_)_6_ were broadly similar and on par with those of the free cofactors (Table 2).

**TABLE 2:** ^57^Fe Mössbauer parameters of clusters (NMe_4_)_2_ **[1^R^]** and complexes **1^R^⊂[M_4_L_6_]** ^x^ and **[Ni_4_L_6_][1^tBu^]** ^x^ taken at zero magnetic field (x = +6, 0 and M═Zn, Ni).

**@70K**	**δ, mm/s**		**ΔE_Q_, mm/s**		**σ,** ^a^ **mm/s** **(×10^−3^)**
	**Fe1**	**Fe2**	**Fe1**	**Fe2**	
(NMe_4_)_2_ **[1^Ph^]**	0.44	0.45	0.77	1.18	3.0
**1^Ph^⊂[Ni_4_L_6_]**(NTf_2_)_6_	0.46	0.47	0.93	1.25	1.3
**@200 K**	**δ, mm/s**		**ΔE_Q_, mm/s**		**σ,** ^a^ **mm/s** **(×10^−3^)**
	**Fe1**	**Fe2**	**Fe1**	**Fe2**	
(NMe_4_)_2_ **[1^Ph^]**	0.37	0.38	0.52	0.87	1.5
**1^Ph^⊂[Zn_4_L_6_]**(NTf_2_)_6_	0.35	0.39	0.66	1.03	0.7
**1^Ph^⊂[Zn_4_L_6_]**	0.44	0.53	0.82	1.26	1.1
(NMe_4_)_2_ **[1^OH^]**	0.36	0.38	1.19	1.01	0.6
**1^OH^⊂[Zn_4_L_6_]**(NTf_2_)_6_	0.31	0.35	0.53	1.07	0.7
**1^OH^⊂[Zn_4_L_6_]**	0.40	0.47	0.80	1.27	1.6
(NMe_4_)_2_ **[1^tBu^]**	0.37	0.37	0.86	0.71	0.4
**[Ni_4_L_6_][1^tBu^]**(NTf_2_)_6_	0.36	0.38	0.78	1.12	0.7
**[Ni_4_L_6_][1^tBu^]**	0.38	0.40	0.68	1.01	1.0

^a^ Root mean square standard deviation.

**FIGURE 8:**
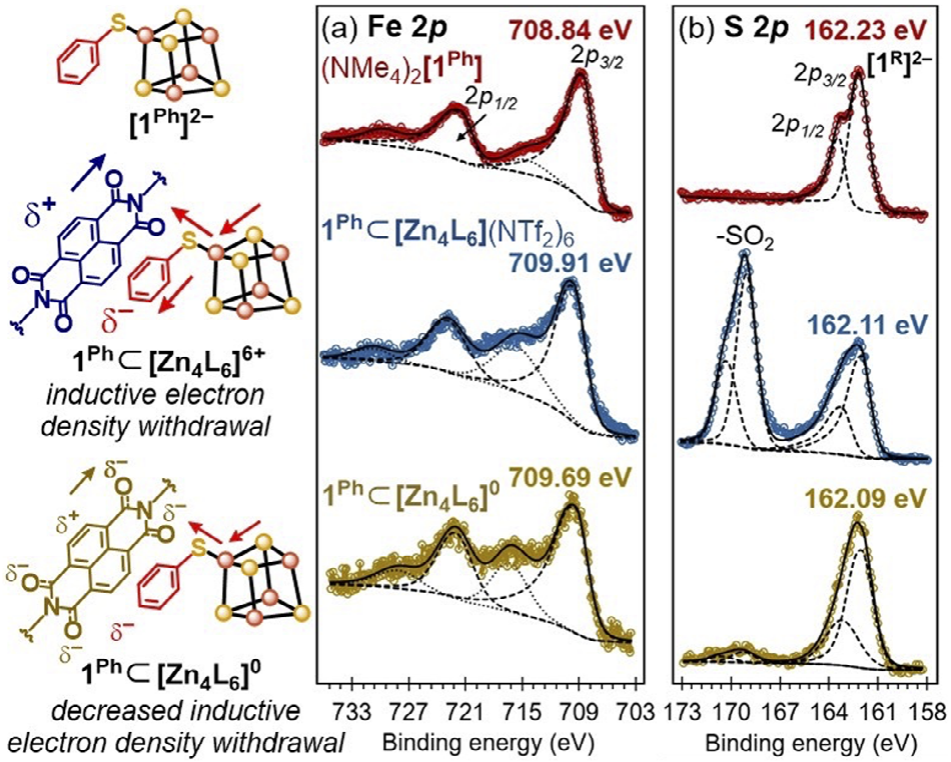
High‐resolution experimental (colored circles) and simulated (black lines) X‐ray photoelectron spectra showing (a) Fe 2*p* and (b) S 2*p* regions of (NMe_4_)_2_[**1^Ph^**], **1^Ph^⊂[Zn_4_L_6_]**(NTf_2_)_6_, and **1^Ph^⊂[Zn_4_L_6_]**. The individual peak fittings for the 2*p*_1/2_ and 2*p*_3/2_ peaks and corresponding satellites are shown with dashed and dotted lines, respectively.

High‐resolution XPS analysis of the Fe 2*p* region for the free clusters, **1^R^⊂[Zn_4_L_6_]**(NTf_2_)_6_, and the ion‐paired control **[Ni_4_L_6_][1^tBu^]**(NTf_2_)_6_ revealed characteristic peaks in the expected 2*p*_3/2_ and 2*p*_1/2_ regions. For the free cofactors (NMe_4_)**[1^R^],** the Fe 2*p*_3/2_ peak binding energies (BEs) ranged from 708.6 to 709.5 eV, broadly consistent with the assigned mixed‐valent Fe^2.5+^ state (707.2–710.2 eV expected for Fe^2+^‐Fe^3+^; Figure 8, Table 3, and Figures S83 and S84) [86]. These values are also consistent with those reported for *Clostiridium* and spinach ferredoxins and *Chromatium* HiPIPs in the same oxidation state (708.0–708.7 eV) [87], yet slightly higher than that reported for the sterically encumbered synthetic analog (K)_2_[Fe_4_S_4_(SDmp)_4_] (Dmp = 2,6‐dimesitylphenyl: 707.5 eV) [27]. Distinct shake‐up satellite peaks were resolved between 714.3 and 715.1 eV, ca. 5.2–6.3 eV higher than the main 2*p*_3/2_ photoemission lines [88, 89]. These features arise from two‐electron transitions involving core‐level ionization coupled with valence‐level excitations. In [FeX_4_]^2−^ systems (X = Cl, SPh), the intensity of these satellites serves as a sensitive probe of metal–ligand covalency [88, 89]. Briefly, increased mixing of Fe 3*d* and S 3*p* orbitals facilitates electronic relaxation via ligand‐to‐metal charge transfer in the photoexcited state, thereby enhancing the satellite transition probability. According to a valence bond configuration interaction (VBCI) model [88, 89], the relaxation energy (*E*_rlx_)—defined as the energy associated with the redistribution of electron density following ionization—is positively correlated with covalency and can be derived via Equation (1):

(1) $$\;E_{\mathrm{rlx}}=W_{f}\;\left(\frac{I_{s}/I_{M}}{1+\left(I_{s}/I_{M}\right)}\right)$$

where *W*_f_ is the energy separation between the main 2*p*_3/2_ peak and its satellite, and *I*_S_ */I*_M_ is the relative intensity of the satellite (*I*_S_) relative to the main 2*p*_3/2_ peak (*I*_M_) [88, 89]. As summarized in Table 3, the calculated *E*_rlx_ values for free cofactors **[1^R^]^2^** ^−^ follow a clear ascending trend, with **[1^Ph^]^2^** ^**−**^ < **[1^OH^]^2^** ^**−**^ < **[1^tBu^]^2^** ^**−**^ (*E*_rlx_ = 0.77, 0.91, and 1.04, respectively). This progression is consistent with an increase in Fe─S bond covalency across the series, a finding that is highly consistent with previously reported X‐ray absorption spectroscopy (XAS) on related synthetic clusters showing a 25% increase in total covalency from **[1^Ph^]^2^** ^−^ to **[1^Et^]^2^** ^−^ [90].

**TABLE 3:** Fe 2*p* XPS parameters of clusters (NMe_4_)_2_ **[1^R^]** and complexes **1^R^⊂[Zn_4_L_6_]** ^x^ and **[Ni_4_L_6_][1^tBu^]** ^x^ (x = +6, 0; RT). Energies are given in eV and referenced to the Au 4f_7/2_ peak of gold foil.

	**Fe 2*p*_3/2_**						
**Complex**	**BE**	**Shift** ^a^	**Satellite** ^b^	***I*_S_/*I*_P_**	***W*_f_**	***E*_rlx_**	**χ^2^** ^c^
(NMe_4_)_2_ **[1^Ph^]**	708.8	0.0	715.1	0.14	6.3	0.77	2.0
**1^Ph^⊂[Zn_4_L_6_]**(NTf_2_)_6_	709.9	+1.1	715.9	0.48	6.0	1.95	2.2
**1^Ph^⊂[Zn_4_L_6_]**	709.7	+0.9	716.5	0.36	6.8	1.80	1.3
(NMe_4_)_2_ **[1^OH^]**	708.6	0.0	714.3	0.19	5.7	0.91	1.7
**1^OH^⊂[Zn_4_L_6_]**(NTf_2_)_6_	709.1	+0.5	715.9	0.52	6.8	2.33	1.6
**1^OH^⊂@[Zn_4_L_6_]**	709.5	+0.9	715.9	0.42	6.4	1.89	1.2
(NMe_4_)_2_ **[1^tBu^]**	709.5	0.0	714.7	0.25	5.2	1.04	2.9
**[Ni_4_L_6_][1^tBu^]**(NTf_2_)_6_	710.0	+0.5	716.0	0.58	6.0	2.20	1.6
**[Ni_4_L_6_][1^tBu^]**	708.9	−0.6	716.2	0.37	7.3	1.97	1.6

^a^ Binding energy (BE) shift vs. unencapsulated cofactor.

^b^ Energy of the shake‐up satellite transition associated with the Fe 2*p*_3/2_ core to valence transition.

^c^ Goodness‐of‐fit parameter.

Upon assembly of the **1^R^⊂[Zn_4_L_6_]**(NTf_2_)_6_ complexes and **[Ni_4_L_6_][1^tBu^]**(NTf_2_)_6_ ion pair, significant positive shifts in the Fe 2*p* binding energies (BE) were observed (Table 3). The greatest change occurred for **1^Ph^⊂[Zn_4_L_6_]**(NTf_2_)_6_, for which the BE shifted by +1.1 eV relative to the free cluster. In contrast, **1^OH^⊂[Zn_4_L_6_]**(NTf_2_)_6_ and the ion‐paired control **[Ni_4_L_6_][1^tBu^]**(NTf_2_)_6_ exhibited identical, smaller shifts of +0.5 eV. These shifts suggest a decrease in Fe electron density upon association with the host, although we acknowledge that subtle variations in Fe─S covalency, electronic symmetry, or magnetic coupling may also contribute. Crucially, the +0.5 eV shift for the ion pair **[Ni_4_L_6_][1^tBu^]**(NTf_2_)_6_ quantifies the baseline electrostatic influence arising from the electrostatic field engendered by the corner metal vertices. The additional +0.6 eV shift in the **[1^Ph^]^2^** ^**−**^ system is thus assigned to direct π‐interfacial contact and the resulting charge redistribution. Meanwhile, single‐component fitting of the S 2*p* region yielded 2*p*_3/2_ binding energies of 162.0 ± 0.3 eV across all three complexes, consistent with the S^2−^ state and consistent with measured values for pyrite (FeS_2_) and (K)_2_[Fe_4_S_4_(SDmp)_4_] (Figure 8b and Figures S83 and S84) [86, 91]. The observation of a single, well‐defined sulfur environment suggests that the binding energy differential between the bridging sulfides and terminal thiolate ligands is below the resolution limit of the instrument, resulting in a single composite photoemission envelope.

The electronic perturbations induced by encapsulation were further evidenced by vibrational and electronic spectroscopies, which revealed subtle but meaningful shifts upon encapsulation. For example, the characteristic C═C stretching frequency of the thiophenolate ligand of **[1^Ph^]^2^** ^**−**^ increased by +2.4 cm^−1^ upon encapsulation, while the energy of the cage NDI C═C and C═O vibrations decreased (up to −1.5 cm^−1^; Table S7). These shifts suggest a trans‐interfacial inductive effect where the electropositive character of NDI framework induces electron density withdrawal from the Fe─S core toward the thiophenolate arene, strengthening internal ligand bonding while simultaneously weakening the NDI framework's bonds via π‐cloud perturbations (Figure 8, left). Meanwhile, the C═O stretching frequencies exhibited the biggest increase upon encapsulation of **[1^OH^]^2^** ^**−**^ (up to −2.9 cm^−1^), consistent with the formation of hydrogen bonds to the mercaptoethanol groups. In the UV–vis spectra, the characteristic ligand‐to‐metal charge transfer (LMCT) bands of **[1^Ph^]^2^** ^**−**^ and **[1^OH^]^2^** ^**−**^ red‐shifted upon encapsulation (0.18 and 0.11 eV, respectively), reflecting the stabilization of acceptor iron orbitals as they become electron‐deficient (Figures S93–S95). For context, ground‐state energy perturbations on the order of 0.2 eV are sufficient to reorder to the spin topology and ground state geometry of the [Fe_4_S_4_] core [92]. While the total ground‐state energy difference and vertical LMCT transition energies are distinct physical quantities, our results demonstrate that the cage environment exerts an electronic influence of a magnitude known to have profound structural and magnetic consequences. This is particularly relevant given the multitude of electronic states accessible within a narrow energy regime, where even subtle perturbations can significantly alter the cluster's potential energy surface [93]. Meanwhile, the UV–vis and IR spectra of the ion‐paired **[Ni_4_L_6_][1^tBu^]**(NTf_2_)_6_ control exhibited negligible shifts. Collectively, these results demonstrate that while the electrostatic field of the cage results in baseline shifts to the Fe 2*p* binding energies, the specific nanoconfinement effects of interfacial π‐stacking and hydrogen bonding drive a significant spatial charge reorganization characterized by withdrawal of electron density from the [Fe_4_S_4_] core.

Further analysis of the XPS photoemission envelopes indicated that the relaxation energies (*E*_rlx_) of **[1^R^]^2^** ^−^ increased by a factor of **2.1–2.6x** upon encapsulation (Table 3). The most significant perturbations were observed for the encapsulated clusters **[1^Ph^]⊂[Zn_4_L_6_]**(NTf_2_)_6_ and **[1^OH^]⊂[Zn_4_L_6_]**(NTf_2_)_6_, where *E*_rlx_ increased by **2.5‐2.6x** relative to the free cofactor. Meanwhile, the **2.1x** increase observed for the ion‐paired **[1^tBu^]^2^** ^**−**^ control established a baseline for purely outer‐sphere electrostatic interactions. The magnitude of these perturbations is remarkably high when compared to traditional ligand‐substitution effects. For example, the transition from **[1^Ph^]^2^** ^**−**^ to **[1^tBu^]^2−^** yielded only a ∼35% increase in *E*_rlx_, corresponding to a 25% increase in total Fe–S covalency as assessed previously using quantitative sulfur K‐edge XAS [90]. While these studies do not permit the assignment of an absolute covalency value, the **2.5‐2.6x** enhancement in *E*_rlx_ for the encapsulated clusters stands in stark contrast to the modest 35% change upon transitioning from **[1^Ph^]^2−^** to **[1^tBu^]^2−^** [90]. This disparity implies that encapsulation induces a covalency increase significantly exceeding the 25% threshold established by traditional ligand substitution. For comparison, in the redox series [K]_n_[Fe_4_S_4_(SDmp)_4_] (*n* = 0–4), a full one‐electron oxidation resulted in a 10%–40% increase in total Fe─S covalency [91]. However, in that system, the intrinsic impact of oxidation is convoluted with the electrostatic influence of the associated potassium counterions, complicating a direct assessment of the electronic changes.

Finally, Fe─S covalency shifts of comparable magnitude have been correlated with the extensive property differences across distinct protein classes. In a landmark sulfur K‐edge XAS study, Solomon and coworkers demonstrated that the covalency difference between *C. vinosum* HiPIP and *Bt* ferredoxin—which operate at opposite ends of the physiological redox scale—is approximately 10% per Fe─S bond [79]. This study also established covalency as a direct proxy for redox potential, where increased Fe─S covalency decreases the reduction potential of the [Fe_4_S_4_] core (ΔE_1/2_ ca. −250 mV for the [Fe_4_S_4_]^2+/1+^ couple for these two proteins). This natural tuning was tied to the electrostatic environment of the clusters—specifically, H‐bonding from internal water molecules to the sulfide ligands, which weakens the Fe─S bonds and destabilizes the oxidized state. In the **[1^R^]⊂[Zn_4_L_6_]**(NTf_2_)_6_ system, the cumulative covalency increase of >25% upon encapsulation is similarly triggered by noncovalent, outer‐sphere interactions, achieving an electronic perturbation approaching the range observed to differentiate between HiPIPs and ferredoxins or the discrete oxidation state changes of [K]_n_[Fe_4_S_4_(SDmp)_4_]. Importantly, a substantial portion of this covalency change appears to be driven by the electrostatic influence of the cationic metal ions, as evidenced by the ion‐paired **[Ni_4_L_6_][1^tBu^]**(NTf_2_)_6_ control, which exhibits similar perturbations. These electrostatic effects on Fe─S covalency mirror similar electronic shifts known to occur in biological systems, such as those induced by the proximity of an a‐helix dipole or by modifications to the local dielectric environment surrounding the clusters [19, 25, 79].

To further understand the nature of NDI–thiolate interactions and their impact on electronic character and bonding at Fe, we conducted ^57^Fe Mössbauer and XPS studies of the six electron‐reduced complexes **1^R^⊂[Zn_4_L_6_]^0^** and **[Ni_4_L_6_][1^tBu^]^0^**. For these studies, solid samples were prepared via reduction by 6 equiv CoCp_2_ in MeCN. This process induced immediate precipitation of the reduced species, ensuring kinetic trapping of the host‐guest complexes before guest expulsion could occur [94]. The integrity of the precipitated products was confirmed via two methods: first, ^1^H NMR analysis of the filtrate established the absence of liberated cofactor; second, a redox‐cycle titration in which the complexes were treated sequentially with CoCp_2_ and AgNTf_2_ demonstrated recovery of the original host‐guest assemblies (Section S7), confirming that the framework remains intact throughout the reduction process. The ^57^Fe Mössbauer isomer shifts measured for **1^Ph^⊂[Zn_4_L_6_]^0^** and **1^OH^⊂[Zn_4_L_6_]^0^** were 0.09–0.14 mm/s and 0.09–0.12 mm/s more positive, respectively, than those of **1^Ph^⊂[Zn_4_L_6_](NTf_2_)_6_** and **1^OH^⊂[Zn_4_L_6_](NTf_2_)_6_** (Table 2). These increases are consistent with the expected change accompanying 1 e‐ reduction of the cluster to the mixed‐valent [Fe_4_S_4_]^1+^ state [26, 83]. In contrast, the isomer shift of **[Ni_4_L_6_][1^tBu^]^0^** increased by only 0.02 mm/s, remaining within the expected range for the [Fe_4_S_4_]^2+^ state. These results suggest that the encapsulated **[1^Ph^]^2−^** and **[1^OH^]^2−^** may be more easily reduced than the free clusters (E_1/2_([Fe_4_S_4_]^2+/1+^: −1.43 and −1.53 V vs. Fc^0/+^ [82], respectively, vs. −0.94 V for [Zn_4_L_6_]^8+/2+^ and −1.33 V for CoCp_2_^1+/0^ in MeCN), [Correction added on August 11, 2026, after first online publication: the previous paragraph, starting with “Mössbauer Isomer shifts” has been updated in this version]. Meanwhile, in the XPS spectra of **1^Ph^⊂[Zn_4_L_6_]^0^** and outer‐sphere control **[Ni_4_L_6_][1^tBu^]^0^**, a negative Fe 2*p* BE shift was observed relative to the non‐reduced assemblies **1^Ph^⊂[Zn_4_L_6_]**(NTf_2_)_6_ (–0.2 eV) and **[Ni_4_L_6_][1^tBu^]**(NTf_2_)_6_ (−1.1 eV). These shifts represent a partial or complete reversal of the electronic perturbations seen upon encapsulation. In contrast, the Fe 2*p* BE of **1^OH^⊂[Zn_4_L_6_]^0^** increased slightly, suggesting that the enhanced H‐bond basicity of the reduced panels intensifies through‐space polarization of the hydroxylated ligands, counteracting the general electrostatic effect. Despite these divergent BE shifts, a consistent decrease in Fe─S covalency was observed across all three systems, as judged from the satellite peak integrations and relaxation energies (*E*_rlx_). This overall trend likely reflects a partial attenuation of the electrostatic influence exerted by the metal corners on the Fe─S bond covalency, modulated by additional dispersive noncovalent interactions.

## 3 Conclusions

Herein, we present a robust and generalizable supramolecular strategy for the systematic modulation of the second and outer coordination spheres of iron–sulfur cofactor models. By leveraging naphthalenediimide (NDI)‐paneled nanocages (**[M_4_L_6_]^8+^**) as biomimetic scaffolds, we successfully encapsulated a series of synthetic [Fe_4_S_4_(SR)_4_]^2−^ clusters (R = Ph, ─CH_2_CH_2_OH), demonstrating that strong binding affinities (*K*_a_ > 10^7^ m ^−1^) can be achieved by coupling size and charge complementarity with specific interfacial interactions, including *π–π* stacking and hydrogen bonding interactions. Multimodal spectroscopic analysis, including ^57^Fe Mössbauer and X‐ray photoelectron spectroscopy, revealed that these host‐guest interactions induce a significant increase in both the electropositive character at the iron centers and the Fe─S bond covalency. These perturbations arise from a combination of the electrostatic field generated by the cationic vertices as well as specific noncovalent interactions between the cage framework and cluster ligands. Remarkably, these framework‐induced electronic shifts exceed the effects of direct primary‐ligand modifications and surpass or approach the electronic changes associated with discrete biological states, such as the ON/OFF switching of electron transfer by the nitrogenase iron protein (NifH) or the distinct redox regimes of ferredoxins vs. high‐potential iron proteins (HiPIPs).

Our results elucidate a dual‐mode tuning mechanism involving interfacial *π–π* stacking or hydrogen bonding operating in tandem with electrostatic effects. These interactions generate a trans‐interfacial inductive effect that withdraws electron density from the [Fe_4_S_4_] core, thereby increasing core covalency. Even relatively weak interactions—as observed in **1^Ph^⊂[M_4_L_6_]**(NTf_2_)_6_—result in measurable shifts in electron density and Fe─S bond covalency due to the close proximity between the Ph groups and NDI. Beyond their fundamental implications, these findings provide critical design principles for modulating and predicting reactivity in complex metalloclusters. Given that Fe─S bond covalency is a decisive factor in sulfur basicity and proton‐relay efficiency, the ability to tune these parameters noncovalently is a significant step toward enzyme‐mimetic catalysis. While conventional ligand derivatization and artificial metalloenzyme engineering often face synthetic bottlenecks, our host‐guest platform allows for the systematic and reversible control of outer‐sphere effects. This research thus opens new avenues for leveraging bio‐inspired iron–sulfur cluster assemblies in industrially relevant catalytic transformations.

## Author Contributions

R.B. and G.B. jointly conceived and planned the experiments. R.B. performed the synthesis of all compounds and collected experimental data. R.B. and G.B. jointly wrote the manuscript. S.D. and J.B. performed the theoretical calculations and prepared the corresponding Supporting Information section.

## Funding

This research was supported by startup funds through the University of Minnesota (UMN), Twin Cities, and by a PPG Excellence Fellowship and a UMN Doctoral Dissertation Fellowship to RB. The authors are grateful to the LeClaire–Dow Instrumentation Facility for NMR and MS characterization, which was supported in part by the National Institute of Health Office of the Director (Award #S10OD011952). The Minnesota NMR Center is thanked for the high‐field NMR measurements. Funding for NMR instrumentation was provided by the Office of the Vice President for Research, the Medical School, the College of Biological Sciences, the College of Pharmacy, NIH, NSF, and the Minnesota Medical Foundation. The Institute for Rock Magnetism (IRM) is thanked for the acquisition of ^57^Fe Mössbauer data. The IRM is a US National Multi‐user Facility supported through the Instrumentation and Facilities program of the National Science Foundation, Earth Sciences Division (NSF EAR‐2153786), and by funding from the UMN. UMN Characterization Facility (Charfac) is thanked for XPS data collection. Charfac receives partial support from the NSF through the MRSEC (Award Number DMR‐2011401) and the NNCI (Award Number ECCS‐2025124) programs.

## Conflicts of Interest

The authors declare no conflicts of interest.

## Supporting information




**Supporting File 1**: smll74001‐sup‐0001‐SuppMat.pdf.

**Supporting File 2**: smll74001‐sup‐0002‐SuppMat.zip.

## Data Availability

The data that support the findings of this study are available from the corresponding author upon reasonable request.
