# Supplementary material for: Outer‐Sphere Tuning of Synthetic Iron–Sulfur Clusters Within Nanoscale Cage Frameworks
Source: Small. 2026 Jun 5;22(49):e74001. doi: 10.1002/smll.74001 (PMC13533253; doi:10.1002/smll.74001)
Supplement: Supplementary file 1 [file SMLL-22-e74001-s002.pdf]

Supplementary Information for:

Outer-Sphere Tuning of Synthetic Iron-Sulfur Clusters within Nanoscale Cage Frameworks

Rupal Baliyan, Shelby T. Davis, Jan-Niklas Boyn, Gwendolyn A. Bailey*

Department of Chemistry, University of Minnesota, Twin Cities, United States, MN-55455

Table of contents:

S1. General Considerations ***S5***

S2. Volume Calculations ***S5***

Figure S1. MoloVol-calculated void space within the crystal structure of **[Zn_4L_6]**$(NTf_2)_8$

Figure S2. MoloVol-calculated molecular volumes of guest clusters $(NMe_4)_2$[**1^R**]

S3. Synthetic Protocols S7

Synthesis of *N,N'*-*p*-(aminophenyl)naphthalenediimide S7

Synthesis of $(NMe_4)_2$**[1^{Ph}]** S7

Synthesis of $(NMe_4)_2$**[1^{tBu}]** S7

Synthesis of $(NMe_4)_2$**[1^{OH}]** S8

Synthesis of **[Ni_4L_6]**$(NTf_2)_8$ S8

Synthesis of host-guest complexes **1^R⊂[M_4L_6]**$(X)_6$ and ion pair **[M_4L_6][1^{tBu}]**$(X)_6$ S9

Synthesis of reduced host-guest complexes **1^R⊂[Zn_4L_6]** and ion pair **[Ni_4L_6][1^{tBu}]** S11

Figure S3. ^{1}H NMR spectra (400 MHz, CD_3CN) of **[Zn_4L_6]**$(NTf_2)_8$, $(NMe_4)_2$**[1^{OH}]**, and **1^{OH}⊂[Zn_4L_6]**$(NTf_2)_6$

Figure S4. ^{1}H NMR spectra (400 MHz, CD_3CN) of **[Fe_4L_6]**$(OTf)_8$, $[NMe_4]_2$**[1^{tBu}]**, and **[Fe_4L_6][1^{tBu}]**$(OTf)_6$

Figure S5. ^{1}H NMR spectra (400 MHz, CD_3CN) of **1^{Ph}⊂[M_4L_6]**$(NTf_2)_6$ (M = Fe, Zn, Ni)

Figure S6. ^{1}H NMR spectra (400 MHz, CD_3CN) of **1^{OH}⊂[M_4L_6]**$(NTf_2)_6$ (M = Fe, Zn, Ni)

Figure S7. ^{1}H NMR spectra (400 MHz, CD_3CN) of **[M_4L_6][1^{tBu}]**$(X)_6$ (X = OTf for M = Fe and NTf_2 for Ni)

S4. NMR Spectroscopy

Calculation of hydrodynamic radii S16

Figure S8. ^{1}H NMR spectra (400 MHz, CD_3CN) of **[Ni_4L_6]**$(NTf_2)_8$

Figure S9. ^{1}H DOSY NMR spectra (400 MHz, CD_3CN) of **[Ni_4L_6]**$(NTf_2)_8$

Figure S10. ^{1}H NMR spectra (400 MHz, CD_3CN) of **[Fe_4L_6]**$(OTf)_8$

Figure S11. ^{1}H DOSY NMR spectra (400 MHz, CD_3CN) of **[Fe_4L_6]**$(OTf)_8$

Figure S12. ^{1}H NMR spectra (400 MHz, CD_3CN) of **1^{Ph}⊂[Fe_4L_6]**$(NTf_2)_6$

Figure S13. ^{1}H NMR spectra (400 MHz, CD_3CN) of **1^{Ph}⊂[Zn_4L_6]**$(NTf_2)_6$

Figure S14. ^{1}H NMR spectra (400 MHz, CD_3CN) of **1^{Ph}⊂[Ni_4L_6]**$(NTf_2)_6$

Figure S15. ^{1}H DOSY NMR spectra (400 MHz, CD_3CN) of $(NMe_4)_2$**[1^{Ph}]**

Figure S16. ^{1}H DOSY NMR spectra (400 MHz, CD_3CN) of **[Fe_4L_6]**$(NTf_2)_8$

Figure S17. ^{1}H DOSY NMR spectra (400 MHz, CD_3CN) of **1^{Ph}⊂[Fe_4L_6]**$(NTf_2)_6$

Figure S18. ^{1}H DOSY NMR spectra (400 MHz, CD_3CN) of **1^{Ph}⊂[Zn_4L_6]**$(NTf_2)_6$

Figure S19. ^{1}H NMR spectra (400 MHz, CD_3CN) of **1^{OH}⊂[Fe_4L_6]**$(NTf_2)_6$

Figure S20. ^{1}H NMR spectra (400 MHz, CD_3CN) of **1^{OH}⊂[Zn_4L_6]**$(NTf_2)_6$

Figure S21. ^{1}H NMR spectra (400 MHz, CD_3CN) of **1^{OH}⊂[Ni_4L_6]**$(NTf_2)_6$

Figure S22. ^{1}H DOSY NMR spectra (400 MHz, CD_3CN) of $(NMe_4)_2$**[1^{OH}]**

Figure S23. ^{1}H DOSY NMR spectra (400 MHz, CD_3CN) of **1^{OH}⊂[Zn_4L_6]**$(NTf_2)_6$

Figure S24. ^{1}H NMR spectra (400 MHz, CD_3CN) of **[Fe_4L_6][1^{tBu}]**$(OTf)_6$

Figure S25. ^{1}H NMR spectra (400 MHz, CD_3CN) of **[Ni_4L_6][1^{tBu}]**$(NTf_2)_6$

Figure S26. ^{1}H DOSY NMR (400 MHz, CD_3CN) of $(NMe_4)_2$**[1^{tBu}]**

Figure S27. ^{1}H DOSY NMR (400 MHz, CD_3CN) of **[Fe_4L_6][1^{tBu}]**$(OTf)_6$

S5. Mass Spectrometry ***S36***

Figure S28. Low-resolution ESI mass spectrum of **[Ni_4L_6]**(NTf_2)$_8$
Figure S29. Low-resolution ESI mass spectrum of **1^{Ph}⊂[Ni_4L_6]**(NTf_2)$_6$
Figure S30. High-resolution ESI mass spectra of **1^{Ph}⊂[Ni_4L_6]$^{6+}$** peak in **1^{Ph}⊂[Ni_4L_6]**(NTf_2)$_6$
Figure S31. High-resolution ESI mass spectra of **1^{Ph}⊂[Ni_4L_6(NTf_2)]$^{5+}$** peak in **1^{Ph}⊂[Ni_4L_6]**(NTf_2)$_6$
Figure S32. Low-resolution ESI mass spectra of **1^{Ph}⊂[Fe_4L_6]**(NTf_2)$_6$
Figure S33. Low-resolution ESI mass spectra of **1^{OH}⊂[Fe_4L_6]**(NTf_2)$_6$
Figure S34. High-resolution ESI mass spectra of **1^{OH}⊂[Fe_4L_6]$^{6+}$** peak in **1^{OH}⊂[Fe_4L_6]**(NTf_2)$_6$
Figure S35. Low-resolution ESI mass spectra of **[Fe_4L_6][1^{tBu}]**(NTf_2)$_6$
Figure S36. High-resolution ESI mass spectra of **[Fe_4L_6][1^{tBu}]$^{6+}$** peak in **[Fe_4L_6][1^{tBu}]**(NTf_2)$_6$

S6. Cyclic Voltammetry ***S41***
Experimental details S39
Table S1. Summary of E_{red}, n, and assignments for redox events in the cyclic voltammograms of **[M_4L_6]**$^{8+}$ (M= Fe, Zn, Ni) and **1^{Ph}⊂[M_4L_6]**$^{6+}$ (M = Fe, Zn).
Table S2. Summary of $E_{1/2}$ values for [M(bpy)$_3$]$^{2+}$ monometallic complexes, with M = Fe, Ni and Zn
Figure S37. CV of **[Fe_4L_6]**(NTf_2)$_8$, **[Ni_4L_6]**(NTf_2)$_8$, and **[Zn_4L_6]**(NTf_2)$_8$ at 2 V/s
Figure S38. CV of **[Zn_4L_6]**(NTf_2)$_8$, (NMe_4)$_2$**[1^{Ph}]**, and **1^{Ph}⊂[Zn_4L_6]**(NTf_2)$_6$ at 2 V/s
Figure S39. CV of **[Zn_4L_6]**(NTf_2)$_8$, (NMe_4)$_2$**[1^{Ph}]**, and **1^{Ph}⊂[Zn_4L_6]**(NTf_2)$_6$ at 2 V/s including a broader scan range for **1^{Ph}⊂[Zn_4L_6]**(NTf_2)$_6$
Figure S40. CV of **[Zn_4L_6]**(NTf_2)$_8$ with different windows of scan at 2 V/s
Figure S41. CV of **1^{Ph}⊂[Zn_4L_6]**(NTf_2)$_8$ with different scan windows at 2 V/s
Figure S42. CV of **[Fe_4L_6]**(NTf_2)$_8$, (NMe_4)$_2$**[1^{Ph}]**, and **1^{Ph}⊂[Fe_4L_6]**(NTf_2)$_6$ at 2 V/s
Figure S43. CV of **A** at 0.2 V/s and at variable scan rates
Figure S44. CV of (NMe_4)$_2$**[1^{Ph}]** at 1 V/s with DPV, and at variable scan rates
Figure S45. CV of **[Fe_4L_6]**(NTf_2)$_8$ at 1 V/s with DPV, and at variable scan rates
Figure S46. (Left) CV of the first redox event of **[Fe_4L_6]**(NTf_2)$_8$ at variable scan rates and (Right) Randles-Ševčík plot of **[Fe_4L_6]**(NTf_2)$_8$
Figure S47. (Left) CV at 1 V/s and DPV of **1^{Ph}⊂[Fe_4L_6]**(NTf_2)$_6$ and (Right) CV at variable scan rates
Figure S48. (Left) CV of the first redox event of **1^{Ph}⊂[Fe_4L_6]**(NTf_2)$_8$ at variable scan rates and (Right) Randles-Ševčík plot of **1^{Ph}⊂[Fe_4L_6]**(NTf_2)$_8$
Figure S49. (Left) CV at 1 V/s and DPV of **[Zn_4L_6]**(NTf_2)$_8$ and (Right) CV at variable scan rates
Figure S50. Randles-Ševčík plot for the first and second cathodic event of **[Zn_4L_6]**(NTf_2)$_6$
Figure S51. Randles-Ševčík plot for the third cathodic event of **[Zn_4L_6]**(NTf_2)$_6$
Figure S52. (Left) CV at 1 V/s and DPV of **1^{Ph}⊂[Zn_4L_6]**(NTf_2)$_6$ and (Right) CV at variable scan rates
Figure S53. Randles-Ševčík plot of the first and second cathodic event of **1^{Ph}⊂[Zn_4L_6]**(NTf_2)$_6$
Figure S54. (Left) CV at 1 V/s and DPV of **[Ni_4L_6]**(NTf_2)$_8$ and (Right) CV at variable scan rates
Figure S55. (Left) CV at 1 V/s and DPV of **1^{Ph}⊂[Ni_4L_6]**(NTf_2)$_6$ and (Right) CV at variable scan rates
Figure S56. Randles-Ševčík plot of the first cathodic event of **[Ni_4L_6]**(NTf_2)$_8$ and **1^{Ph}⊂[Ni_4L_6]**(NTf_2)$_6$
Figure S57. CV of (NMe_4)$_2$**[1^{Ph}]**, (NMe_4)$_2$**[1^{OH}]**, and (NMe_4)$_2$**[1^{tBu}]** at 0.2 V/s

S7. Redox Titrations ***S56***
Figure S58. ^{1}H NMR spectra (400 MHz, CD_3CN) for the redox titration of **[Fe_4L_6]**(NTf_2)$_8$
Figure S59. ^{1}H NMR spectra (400 MHz, CD_3CN) for the redox titration of **1^{Ph}⊂[Fe_4L_6]**(NTf_2)$_6$
Figure S60. ^{1}H NMR spectra (400 MHz, CD_3CN) for the redox titration of **1^{OH}⊂[Fe_4L_6]**(NTf_2)$_6$
Figure S61. ^{1}H NMR spectra (400 MHz, CD_3CN) for the redox titration of **[Fe_4L_6][1^{tBu}]**(NTf_2)$_6$

S8. ^{57}Fe Mössbauer Spectroscopy ***S60***
Experimental details S60
Table S3. Mössbauer parameters of (NMe_4)$_2$**[1^R]** and **1^R⊂[M_4L_6]**^{x}; x= +6, 0, and M = Zn, Ni
Figure S62. ^{57}Fe Mössbauer spectra of (NMe_4)$_2$**[1^{Ph}]** at 70 K and 200 K
Figure S63. ^{57}Fe Mössbauer spectra of **1^{Ph}⊂[Ni_4L_6]**(NTf_2)$_6$ (70 K) in two different fits
Figure S64. ^{57}Fe Mössbauer spectra of **1^{Ph}⊂[Zn_4L_6]**(NTf_2)$_6$ (200 K) in two different fits.
Figure S65. ^{57}Fe Mössbauer spectra of **1^{Ph}⊂[Zn_4L_6]** (200 K) in two different fits
Figure S66. ^{57}Fe Mössbauer spectra of (NMe_4)$_2$**[1^{OH}]** (200 K) in two different fits
Figure S67. ^{57}Fe Mössbauer spectra of **1^{OH}⊂[Zn_4L_6]**(NTf_2)$_6$ (200 K)
Figure S68. ^{57}Fe Mössbauer spectra of **1^{OH}⊂[Zn_4L_6]** (200 K) in two different fits
Figure S69. ^{57}Fe Mössbauer spectra of (NMe_4)$_2$**[1^{tBu}]** (200 K) in two different fits

Figure S70. ^{57}Fe Mössbauer spectra of **[Ni$_4$L$_6$][1^{tBu}]**(NTf$_2$)$_6$ (200 K) in two different fits
Figure S71. ^{57}Fe Mössbauer spectra of **[Ni$_4$L$_6$][1^{tBu}]** (200 K) in two different fits
Figure S72. ^{57}Fe Mössbauer spectra with Residuals (data – global fit) of (NMe$_4$)$_2$**[1^{Ph}]** at 70 K and 200 K
Figure S73. ^{57}Fe Mössbauer spectra with Residuals (data – global fit) of **1^{Ph}⊂[Ni$_4$L$_6$]**(NTf$_2$)$_6$ at 70 K
Figure S74. ^{57}Fe Mössbauer spectra with Residuals (data – global fit) of **1^{Ph}⊂[Zn$_4$L$_6$]**(NTf$_2$)$_6$ at 200 K
Figure S75. ^{57}Fe Mössbauer spectra with Residuals (data – global fit) of **1^{Ph}⊂[Zn$_4$L$_6$]** at 200 K
Figure S76. ^{57}Fe Mössbauer spectra with Residuals (data – global fit) of (NMe$_4$)$_2$**[1^{OH}]** at 200 K
Figure S77. ^{57}Fe Mössbauer spectra with Residuals (data – global fit) of **1^{OH}⊂[Zn$_4$L$_6$]**(NTf$_2$)$_6$ at 200 K
Figure S78. ^{57}Fe Mössbauer spectra with Residuals (data – global fit) of **1^{OH}⊂[Zn$_4$L$_6$]** at 200 K
Figure S79. ^{57}Fe Mössbauer spectra with Residuals (data – global fit) of (NMe$_4$)$_2$**[1^{tBu}]** at 200 K
Figure S80. ^{57}Fe Mössbauer spectra with Residuals (data – global fit) of **[Ni$_4$L$_6$][1^{tBu}]**(NTf$_2$)$_6$ at 200 K
Figure S81. ^{57}Fe Mössbauer spectra with Residuals (data – global fit) of **[Ni$_4$L$_6$][1^{tBu}]** at 200 K

S9. X-Ray Photoelectron Spectroscopy ***S75***
Experimental details S75
Table S4. XPS parameters of Fe 2*p* state for the (NMe$_4$)$_2$**[1^{R}]** and **1^{R}⊂[M$_4$L$_6$]**^{x}; x= +6, 0, and M = Zn, Ni
Table S5. XPS parameters of S 2*p* state for the (NMe$_4$)$_2$**[1^{R}]** and **1^{R}⊂[M$_4$L$_6$]**^{x}; x= +6, 0, and M = Zn, Ni
Figure S82. Fe 2*p* and S 2*p* XPS spectra of (NMe$_4$)$_2$**[1^{Ph}]**, **1^{Ph}⊂[Zn$_4$L$_6$]**(NTf$_2$)$_6$ and **1^{Ph}⊂[Zn$_4$L$_6$]**
Figure S83. Fe 2*p* and S 2*p* XPS spectra of (NMe$_4$)$_2$**[1^{OH}]**, **1^{OH}⊂[Zn$_4$L$_6$]**(NTf$_2$)$_6$ and **1^{OH}⊂[Zn$_4$L$_6$]**
Figure S84. Fe 2*p* and S 2*p* XPS spectra of (NMe$_4$)$_2$**[1^{tBu}]**, **1^{tBu}⊂[Ni$_4$L$_6$]**(NTf$_2$)$_6$ and **1^{tBu}⊂[Ni$_4$L$_6$]**
Figure S85. Fe *2p* XPS spectra with residuals (data – fit) of (NMe$_4$)$_2$**[1^{Ph}]**, **1^{Ph}⊂[Zn$_4$L$_6$]**(NTf$_2$)$_6$ and **1^{Ph}⊂[Zn$_4$L$_6$]**
Figure S86. S *2p* XPS spectra with residuals (data – fit) of (NMe$_4$)$_2$**[1^{Ph}]**, **1^{Ph}⊂[Zn$_4$L$_6$]**(NTf$_2$)$_6$ and **1^{Ph}⊂[Zn$_4$L$_6$]**
Figure S87. Fe *2p* XPS spectra with residuals (data–fit) of (NMe$_4$)$_2$**[1^{OH}]**, **1^{OH}⊂[Zn$_4$L$_6$]**(NTf$_2$)$_6$ and **1^{OH}⊂[Zn$_4$L$_6$]**
Figure S88. S *2p* XPS spectra with residuals (data–fit) of (NMe$_4$)$_2$**[1^{OH}]**, **1^{OH}⊂[Zn$_4$L$_6$]**(NTf$_2$)$_6$ and **1^{OH}⊂[Zn$_4$L$_6$]**
Figure S89. Fe *2p* XPS spectra with residuals (data–fit) of (NMe$_4$)$_2$**[1^{tBu}]**, **[Zn$_4$L$_6$][1^{tBu}]**(NTf$_2$)$_6$ and **[Zn$_4$L$_6$][1^{tBu}]**
Figure S90. S *2p* XPS spectra with residuals (data–fit) of (NMe$_4$)$_2$**[1^{tBu}]**, **[Zn$_4$L$_6$][1^{tBu}]**(NTf$_2$)$_6$ and **[Zn$_4$L$_6$][1^{tBu}]**

S10. UV-vis and IR Spectroscopy ***S86***
Table S6. UV-vis spectral parameters for (NMe$_4$)$_2$**[1^{R}]** and **1^{R}⊂[M$_4$L$_6$]**(NTf$_2$)$_6$ (M = Fe, Zn, Ni)
Table S7. IR spectral parameters for (NMe$_4$)$_2$**[1^{R}]** and **1^{R}⊂[M$_4$L$_6$]**(NTf$_2$)$_6$ (M = Zn, Ni)
Figure S91. (Left) UV-Visible spectra of (NMe$_4$)$_2$**[1^{Ph}]** and **1^{Ph}⊂[M$_4$L$_6$]**(NTf$_2$). (Right) IR spectra of **[Zn$_4$L$_6$]**(NTf$_2$)$_8$ and **1^{Ph}⊂[Zn$_4$L$_6$]**(NTf$_2$)$_6$ with the IR stretching frequencies for C=C bond of the linker
UV-Vis Spectroscopy S88
Figure S92. Stacked UV-vis spectra of **[M$_4$L$_6$]**(NTf$_2$)$_8$ (M = Fe, Ni and Zn) with full length, and the region of interest
Figure S93. Stacked UV-vis spectra of (NMe$_4$)$_2$**[1^{R}]** with full length and the region of interest
Figure S94. Stacked UV-vis spectra of **1^{Ph}⊂[M$_4$L$_6$]**(NTf$_2$)$_6$, **1^{OH}⊂[M$_4$L$_6$]**(NTf$_2$)$_6$, and **1^{tBu}⊂[M$_4$L$_6$]**(NTf$_2$)$_6$ (M = Fe, Ni and Zn) with x axis in nm and shown region of interest
Figure S95. Stacked UV-vis spectra of **1^{Ph}⊂[M$_4$L$_6$]**(NTf$_2$)$_6$, **1^{OH}⊂[M$_4$L$_6$]**(NTf$_2$)$_6$, and **[M$_4$L$_6$][1^{tBu}]**(NTf$_2$)$_6$ (M= Fe, Ni and Zn) with x axis in eV and shown region of interest
Figure S96. Stacked UV-vis spectra of (NMe$_4$)$_2$**[1^{tBu}] + [Ni$_4$L$_6$]**(NTf$_2$)$_8$ and **[Ni$_4$L$_6$][1^{tBu}]**(NTf$_2$)$_6$ with full length and region of interest
Figure S97. Stacked UV-vis spectra of **[M$_4$L$_6$]**(NTf$_2$)$_8$, **1^{Ph}⊂[M$_4$L$_6$]**(NTf$_2$)$_6$, and **1^{OH}⊂[M$_4$L$_6$]**(NTf$_2$)$_6$, and **1^{tBu}⊂[M$_4$L$_6$]**(NTf$_2$)$_6$ (M = Fe, Ni and Zn) with the full length
IR Spectroscopy S93
Figure S98. ATIR spectra of **[Zn$_4$L$_6$]**(NTf$_2$)$_8$, **[Fe$_4$L$_6$]**(NTf$_2$)$_8$, and **[Ni$_4$L$_6$]**(NTf$_2$)$_8$
Figure S99. ATIR spectra of (NMe$_4$)$_2$**[1^{Ph}]**, **[Zn$_4$L$_6$]**(NTf$_2$)$_8$, and **1^{Ph}⊂[Zn$_4$L$_6$]**(NTf$_2$)$_6$
Figure S100. ATRIR spectra of (NMe$_4$)$_2$**[1^{OH}]**, **[Zn$_4$L$_6$]**(NTf$_2$)$_8$, and **1^{OH}⊂[Zn$_4$L$_6$]**(NTf$_2$)$_6$
Figure S101. Stacked ATIR spectra of **[Zn$_4$L$_6$]**(NTf$_2$)$_8$ and **1^{OH}⊂[Zn$_4$L$_6$]**(NTf$_2$)$_6$ highlighting C=O stretching bands, and (NMe$_4$)$_2$**[1^{OH}]** and **1^{OH}⊂[Zn$_4$L$_6$]**(NTf$_2$)$_6$ highlighting O-H stretching band
Figure S102. ATIR spectra of (NMe$_4$)$_2$**[1^{tBu}]**, **[Ni$_4$L$_6$]**(NTf$_2$)$_8$, and **1^{tBu}⊂[Ni$_4$L$_6$]**(NTf$_2$)$_6$

S11. Binding Titration Experiments ***S97***
Table S8. Summary of ^{1}H NMR chemical shift values of cluster (NMe$_4$)$_2$**[1^{R}]** in CD_3CN at RT
Table S9. Summary of ^{1}H NMR chemical shift values of cluster **[1^{R}]$^{2-}$** in **1^{R}⊂[Fe$_4$L$_6$]**(NTf$_2$)$_6$ and **[Fe$_4$L$_6$][1^{tBu}]**(NTf$_2$)$_6$ in CD_3CN at RT

Table S10. Summary of association constant, [HG] value and probability of binding of cluster **[1^{R}]$^{2-}$** in complexes **1^{R}⊂[Fe$_4$L$_6$]**(NTf$_2$)$_6$ and **[Fe$_4$L$_6$][1^{tBu}]**(NTf$_2$)$_6$ in CD$_3$CN at RT
Figure S103. ^{1}H NMR spectra (900 MHz, CD$_3$CN) of 1:1 and 10:1 mixture of **[Fe$_4$L$_6$](**NTf$_2$)$_8$ (H) and (NMe$_4$)$_2$**[1^{Ph}]** (G)
Figure S104. ^{1}H NMR spectra (900 MHz, CD$_3$CN) of 1:1 and 10:1 mixture of **[Fe$_4$L$_6$](**NTf$_2$)$_8$ (H) and (NMe$_4$)$_2$**[1^{OH}]** (G)
Figure S105. ^{1}H NMR spectra (900 MHz, CD$_3$CN) of 1:1 and 10:1 mixture of **[Fe$_4$L$_6$](**NTf$_2$)$_8$ (H) and (NMe$_4$)$_2$**[1^{tBu}]** (G)
Figure S106. ^{1}H NMR spectra (850 MHz, CD$_3$CN) of mixtures of **[Fe$_4$L$_6$](**NTf$_2$)$_8$ (H) and (NMe$_4$)$_2$**[1^{tBu}]** (G) at increasing host concentrations in H:G from 1:1 to 50:1
Figure S107. UV-VIS absorption spectra of mixtures of **[Ni$_4$L$_6$](**NTf$_2$)$_8$ (H) and (NMe$_4$)$_2$**[1^{tBu}]** (G) at H:G ratios of 1:1 and 10:1
S12. Computational Methods ***S104***
Figure S108. DFT-optimized structure of **1^{Ph}**⊂**[Zn$_4$L$_6$]$^{6+}$**. Level of theory: TPSS/D3BJ/def2-SVP for geometry optimizations and TPSS/D3BJ/MINIX for single-point and frequency calculations
Figure S109. DFT-optimized structure of **1^{OH}**⊂**[Zn$_4$L$_6$]$^{6+}$**. Level of theory: TPSS/D3BJ/def2-SVP for geometry optimizations and TPSS/D3BJ/MINIX for single-point and frequency calculations.
Figure S110. DFT-optimized structure of **1^{tBu}**⊂**[Zn$_4$L$_6$]$^{6+}$**. Level of theory: TPSS/D3BJ/def2-SVP for geometry optimizations and TPSS/D3BJ/MINIX for single-point and frequency calculations
S13. References ***S107***

S1. GENERAL CONSIDERATIONS

Unless otherwise specified, all synthetic procedures and electrochemical measurements were performed under a dry N_2 atmosphere using an MBraun glovebox or a Schlenk line. The organic solvents acetonitrile (MeCN), diethyl ether (Et_2O), and *N,N*-dimethyl formamide (DMF) were dried by passage through double Pure Process Technology alumina solvent purification columns and degassed by sparging with Ar. DMF was additionally purified by passage through an isocyanate scrubbing column. Dry, degassed methanol and ethyl acetate were obtained by drying over calcium hydride for 12 h and distilling under N_2 on the Schlenk line under an inert atmosphere prior to use. Solvents, once dried and degassed, were stored in the glovebox over 3 Å molecular sieves. NMR solvents (MeCN, DMSO) were purchased from Cambridge Isotopes, degassed via at least three consecutive freeze/pump/thaw cycles and dried over freshly activated 3 Å molecular sieves for at least 16 h prior to use. Crystalline samples of $Fe(NTf_2)_2$[1] and **[M$_4$L$_6$]**$(NTf_2)_8$ (M= Zn, Fe)[2,3] were prepared according to literature procedures (NTf_2 = bistriflimide). **[Fe$_4$L$_6$]**$(OTf)_8$ was prepared following a similar procedure for **[Fe$_4$L$_6$]**$(NTf_2)_8$,[3] but using $Fe(OTf)_2$ as the Fe^{2+} source (OTf = triflate). The ^{1}H NMR data matched that reported for **[Fe$_4$L$_6$]**$(NTf_2)_8$. All other reagents including trimethoxy benzene, 1,4,5,8-naphthalenetetracarboxylic dianhydride, 1,4-diaminobenzene, triethylamine, water, acetone, chloroform, sodium chunks, thiophenol, anhydrous ferric chloride ($FeCl_3$), elemental sulfur, tetramethylammonium bromide, *tert*-butyl thiol, mercaptoethanol, cobaltacene ($CoCp_2$), silver triflimide ($AgNTf_2$), gold foil, SpectroBlend® powder, tetraalkylammonium hexafluorophosphate $[NR_4][PF_6]$ (R= ^{n}Bu, Me), zinc triflimide ($Zn(NTf_2)_2$), nickel triflimide ($Ni(NTf_2)_2$), iron triflate ($Fe(OTf)_2$) were purchased from commercial sources and used as received.

NMR spectra were recorded on a Bruker Avance III HD 400 MHz spectrometer at 298 K and referenced to residual solvent peaks. ^{1}H chemical shifts (δ) are reported in parts per million (ppm). ESI mass spectra were recorded on a Bruker BioTOF II ESI/TOF-MS (low resolution) or an Orbitrap IQ-X Tribrid mass spectrometer (high resolution) in positive ion mode. Samples for ESI were prepared inside the glovebox and maintained at –40 °C (MeCN/dry ice) in an air-sealed GC vial prior to injection. For analysis on the Orbitrap IQ-X Tribrid MS, the samples were infused into the instrument from a gas-tight syringe pump at a rate of 2 µL per minute. The simulated isotopic distribution patterns of mass spectra were calculated using Mass Spectrum Simulator online tool.[5] ATR-IR spectra were recorded on a Thermo Scientific™ iS5 FTIR with an iD5 diamond ATR. UV-vis spectra were recorded using glass cuvettes on a Varian Cary 300 Bio spectrophotometer. Generative AI (Google Gemini and OpenAI ChatGPT) was employed to polish language and improve structural flow; the conceptualization, data interpretation, and original drafting of the manuscript remain the work of the authors, who maintain full accountability for the manuscript's content.

S2. VOLUME CALCULATIONS

MoloVol calculates the cavity and molecular volumes of chemical structures by simulating the "rolling" of probes around atoms.[4] These probes mimic solvent molecules and are used to define the accessible and excluded volumes of a structure. Regions occupied by the probe but enclosed within the molecule are identified as cavities. The single-probe mode is used to calculate the molecular volume of a guest molecule by rolling a probe along its surface. In contrast, the two-probe mode is employed to analyze large internal cavities that are connected to the exterior. In this mode, a larger "outer" probe defines the external surface of the cage, while a smaller "inner" probe identifies the internal space accessible within the cavity.

Host Cavity Volume. To estimate the accessible void space within the structure of **[M$_4$L$_6$]**$(NTf_2)_8$, cavity volume calculations were performed using MoloVol v1.1.0[4] using the reported crystal structure of **[Zn$_4$L$_6$]**$(NTf_2)_8$.[2] The two-probe method was employed, using an outer probe radius of 4.3 Å to define the exterior surface which lies near the lower limit required to block access to the internal cavity, thereby accurately defining the exterior boundary. An inner probe radius of 1.2 Å was used to map the accessible internal cavity. The following standard parameters were used:

- Probe mode: Two-probe
- Outer probe radius: 4.3 Å
- Inner probe radius: 1.2 Å
- Grid resolution: 0.1 Å
- Optimization depth: 4

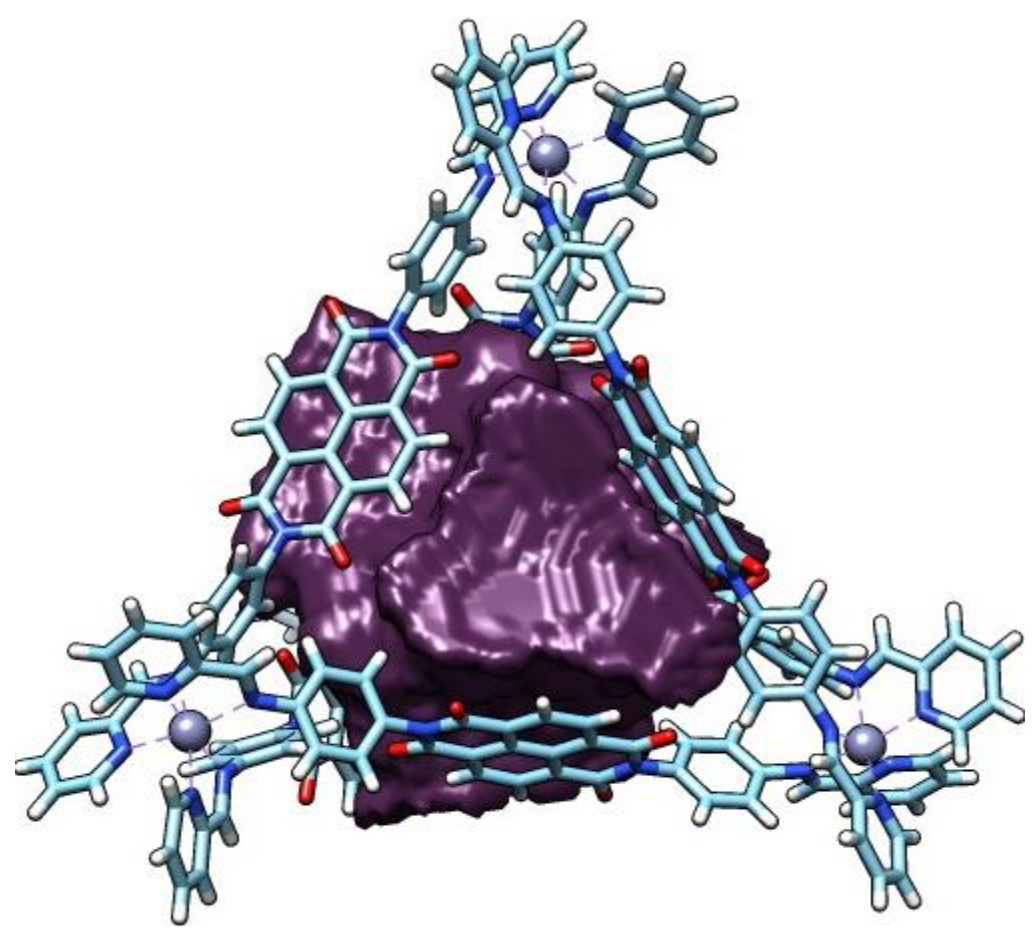

Figure S1. Molecular diagram showing MoloVol-calculated void space (purple) within the reported crystal structure of **[Zn_4L_6]**(NTf_2)$_8$,[2] with a total cavity volume of 1113 Å^3. For the purpose of this calculation, the countercations and solvent molecules were omitted.

Guest Volume. To determine the molecular volumes of the guest clusters (NMe_4)$_2$**[1^R]**, calculations were performed using MoloVol v1.1.0 starting from the reported crystallographic coordinates.[5–7] The single-probe method was applied with a probe radius of 1.2 Å to estimate the solvent-accessible molecular volume. The following parameters were used:

- Probe mode: Single-probe
- Probe radius: 1.2 Å
- Grid resolution: 0.1 Å
- Optimization depth: 4

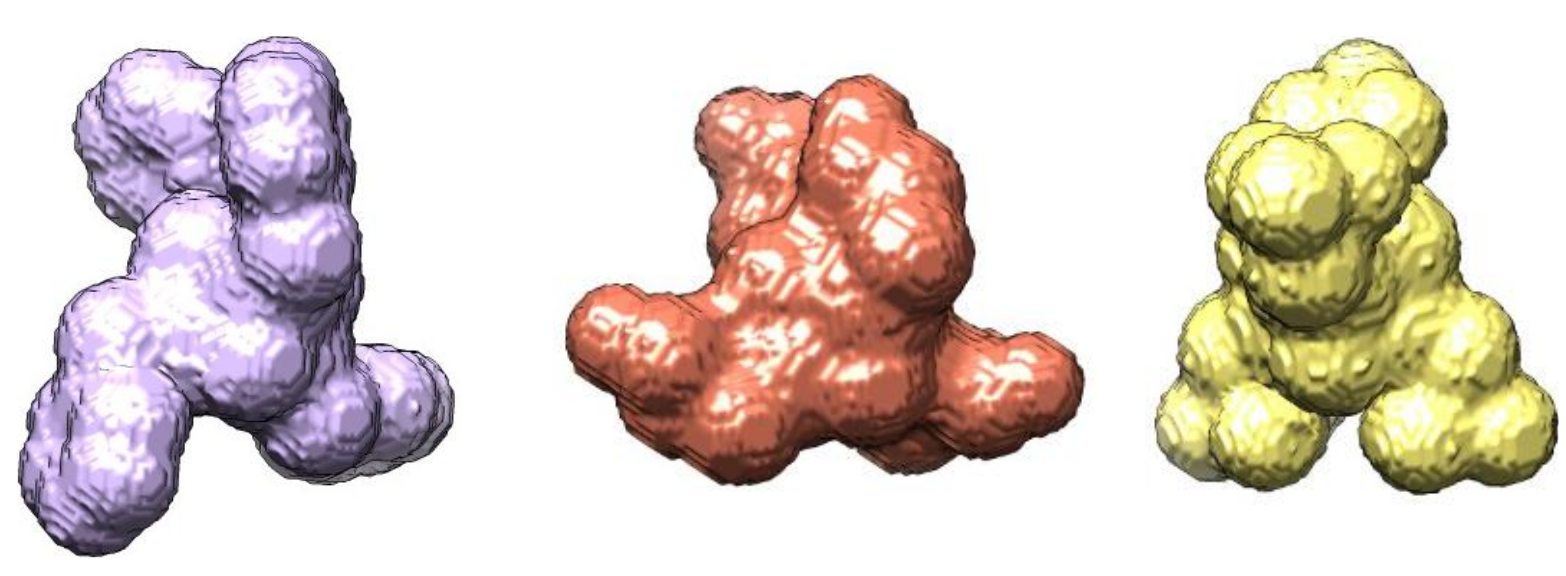

Figure S2. Molecular diagram showing MoloVol-calculated molecular volumes of guest clusters (NMe_4)$_2$**[1^R]**, based on their crystal structures.[5–7] From left to right: (NMe_4)$_2$**[1^{Ph}]**, (NMe_4)$_2$**[1^{OH}]** and (NMe_4)$_2$**[1^{tBu}]** with calculated volumes of 574 Å^3, 432 Å^3 and 529 Å^3, respectively. For the purposes of this calculation, the countercations NMe_4^+ were omitted from the crystal structures.

S3. SYNTHETIC PROTOCOLS

Synthesis of N,N'-p-(aminophenyl)naphthalenediimide (A)

Synthesis of diimide **A** was carried out according to the reported procedure,[2] but with heating via a hotplate instead of microwave irradiation. 1,4,5,8-naphthalenetetracarboxylic dianhydride (4.00 g, 14.9 mmol) and 1,4-diaminobenzene (8.06 g, 74.6 mmol, 5.1 equiv) were suspended in 250 mL of dry DMF, in a 500 mL round bottom flask. Triethylamine (12 mL, 86.3 mmol) was added and the reaction mixture was heated to reflux for 12 h. The resulting mixture was filtered, and the residue was washed with copious amounts of water, acetone and then chloroform. Drying in vacuo yielded diimide **A** as a crude dark brown powder (6.05 g, 91%) which was used without further purification. The ^{1}H NMR spectra matched with those reported in literature.[2]

Synthesis of $(NMe_4)_2$[1^{Ph}]

Synthesis of $(NMe_4)_2$**[1^{Ph}]** was carried out according to the reported procedure,[5,8] with minor modifications: 1) the reaction was carried out exclusively in the glovebox, and 2) crystallization was accomplished by layering diethyl ether over an acetonitrile solution of the crude product, instead of slow cooling of a hot methanol solution. Thus, dry, degassed methanol (80 mL) was added to a 250 mL Schlenk flask with a stir bar and then cooled to ~5 °C inside the glovebox cold well (ice/water bath). Small sodium chunks (1.8 g, 80 mmol, 4 equiv) were added slowly with continued cooling and stirring and allowed to fully dissolve before warming the flask to room temperature. Thiophenol (8.2 mL, 80 mmol, 4 equiv) was then injected into the reaction mixture, forming sodium thiophenolate. In a separate 250 mL Schlenk flask, $FeCl_3$ (3.2 g, 20 mmol, 1 equiv) was dissolved in methanol (50 mL) and the solution was transferred to the flask containing the sodium thiophenolate. Finally, elemental sulfur (0.64 g, 20 mmol, 1 equiv) was added and the reaction mixture stirred at room temperature for 12 h. This mixture was then filtered into a solution of $[NMe_4][Br]$ (2.3 g, 15 mmol) in methanol (45 mL), resulting in precipitation of the crude product as a black solid. This suspension was filtered, and the resulting solid was redissolved in MeCN (40 mL) and layered with 100 mL of diethyl ether. Cooling to −40 °C resulted in the formation of large black crystals that were collected by filtration, washed with ether, and dried in vacuo yielding (NMe_4)**[1^{Ph}]** as a black crystalline solid (4.05 g, 87%). The ^{1}H NMR spectra matched with those reported in the literature.[5,8]

Synthesis of $(NMe_4)_2$[1^{tBu}]

Synthesis of $(NMe_4)_2$**[1^{tBu}]** was carried out according to the reported procedure,[9] but with minor modifications: 1) the reaction was entirely carried out in the glovebox, and 2) crystallization was accomplished by layering diethyl ether over an acetonitrile solution of the crude product, instead of layering the hot acetonitrile solution with ethyl acetate. Dry, degassed methanol (20 mL) was added to a 100 mL Schlenk flask with a stir bar and then cooled to ~5 °C inside the glovebox cold well (ice/water bath). Small sodium chunks (0.45 g, 20 mmol) were added slowly with continued cooling and stirring and allowed to fully dissolve before warming the flask to room temperature. *tert*-Butyl thiol (1.8 mL, 20 mmol) was then injected into the reaction mixture, forming sodium *tert*-butyl thiolate. In a separate 100 mL Schlenk flask, $FeCl_3$ (0.8 g, 5 mmol) was dissolved in MeOH (10 mL) and the solution was transferred to the flask containing the sodium *tert*-butyl thiolate. Finally, elemental sulfur (0.16 g, 5 mmol) was added and the reaction mixture stirred at room temperature for 12 h. This mixture was then filtered into a solution of $[NMe_4][Br]$ (0.575 g, 3.75 mmol) in methanol (15 mL), resulting in precipitation of the crude product as a black solid. This suspension was filtered, and the resulting solid was dissolved in acetonitrile (10 mL) and layered with 25 mL of diethyl ether. Cooling to −40 °C resulted in the formation of large black crystals that were collected by filtration, washed with ether, and dried in vacuo yielding (NMe_4)**[1^{tBu}]** as a black crystalline solid (0.69 g, 66%). The ^{1}H NMR spectra matched with those reported in the literature.[6]

Synthesis of $(NMe_4)_2$[1^{OH}]

Synthesis of $(NMe_4)_2$**[1^{OH}]** was carried out according to the reported procedure,[9] but in the glovebox instead of on Schlenk line.[9] To a solution of $(NMe_4)_2$**[1^{tBu}]** (0.5 g, 0.58 mmol) in acetonitrile (10 mL) were added 5 equivalents of mercaptoethanol (0.21 mL, 0.29 mmol). The solution was stirred for 1 h, after which time the solvent was removed in vacuo. The residue was redissolved in warm acetonitrile (50 °C, 10 mL) and filtered, where upon ethyl acetate (10 mL) was added to the filtrate. Cooling to −40 °C resulted in the formation of black crystals that were collected by filtration, washed with ethyl acetate, and dried in vacuo yielding (NMe_4)**[1^{OH}]** as a black crystalline solid (0.36 g, 76%). The ^{1}H NMR spectra matched with those reported in the literature.[9]

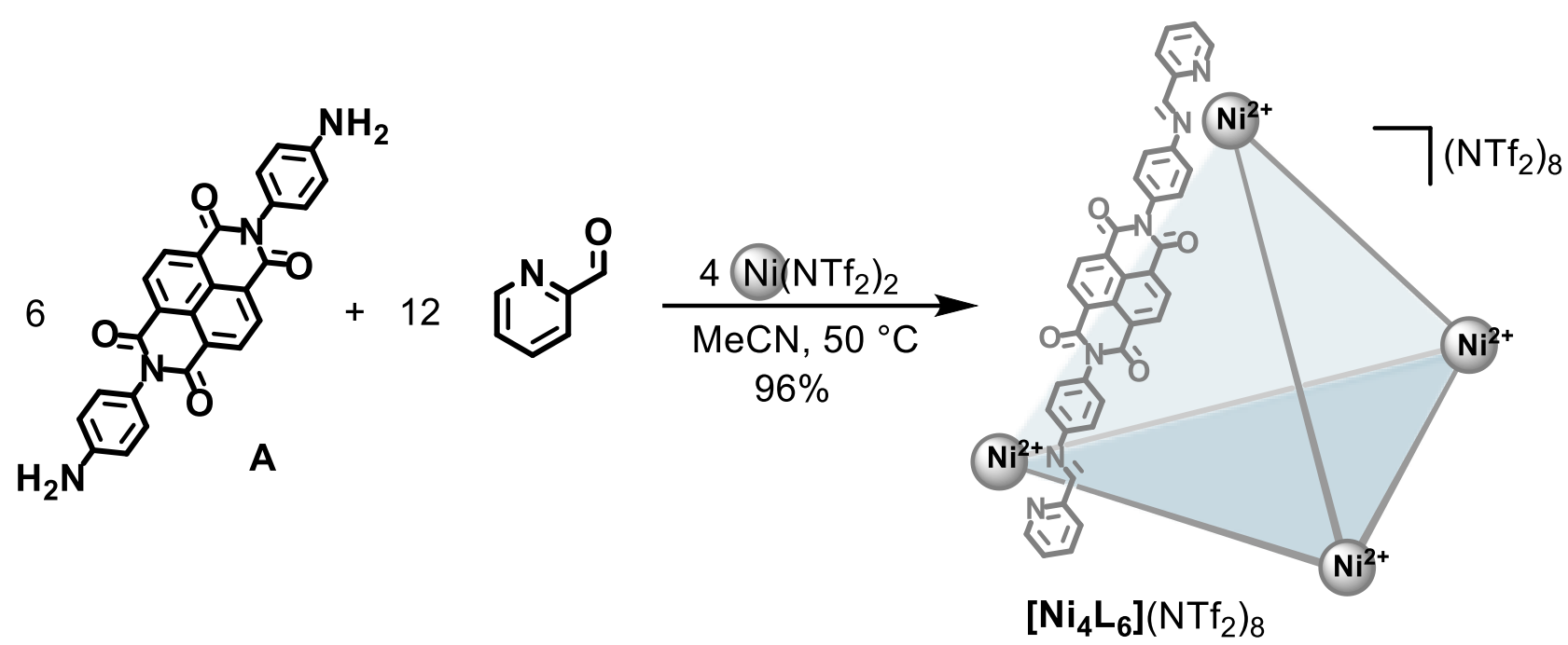

Scheme S1. Synthesis of **[Ni_4L_6]**$(NTf_2)_8$.

Synthesis of [Ni_4L_6](NTf_2)$_8$

[Ni_4L_6]$(NTf_2)_8$ was synthesized according to a modified literature protocol for **[Zn_4L_6]**$(NTf_2)_8$,[2] but with $Ni(NTf_2)_2$ in place of $Zn(NTf_2)_2$. In the glovebox, a 20 mL scintillation vial was charged with diamine **A** (90 mg, 0.2 mmol), $Ni(NTf_2)_2$ (85 mg, 0.13 mmol), and 2-formylpyridine (38 µL, 0.4 mmol) in acetonitrile (15 mL) with a magnetic stir bar. The vial was sealed and heated at 50 °C with stirring for 12 h, during which time the color changed from brown to orange. The crude reaction mixture was filtered through Celite and the filtrate precipitated with diethyl ether (15 mL) at 0 °C. Filtration afforded **[Ni_4L_6]**$(NTf_2)_8$ as an orange crystalline solid (198 mg, 96%). ^{1}H NMR (400 MHz, CD_3CN; Figure S8) δ: 52.90, 19.92, 18.54, 16.20, 14.33, 13.44, 8.73, -11.58. ESI-MS (MeCN, m/z; Figure S28): 499.3 (**[Ni_4L_6]**$^{8+}$, 43.4%), 610.7 (**[Ni_4L_6**(NTf_2)**]**$^{7+}$, 100%), 759.2 (**[Ni_4L_6**$(NTf_2)_2$**]**$^{6+}$, 72.1%), 967.0 (**[Ni_4L_6**$(NTf_2)_3$**]**$^{5+}$, 25.3%), 1278.8 (**[Ni_4L_6**$(NTf_2)_4$**]**$^{4+}$, 6.1%). ^{1}H DOSY NMR (CD_3CN, 400 MHz, 25 °C; Figure S9): D = 3.2×10^{-10} m^2/s; r_H = 19.8 Å.

Synthesis of host-guest complexes $1^R \subset [M_4L_6](X)_6$ and ion pair $[M_4L_6][1^{tBu}](X)_6$

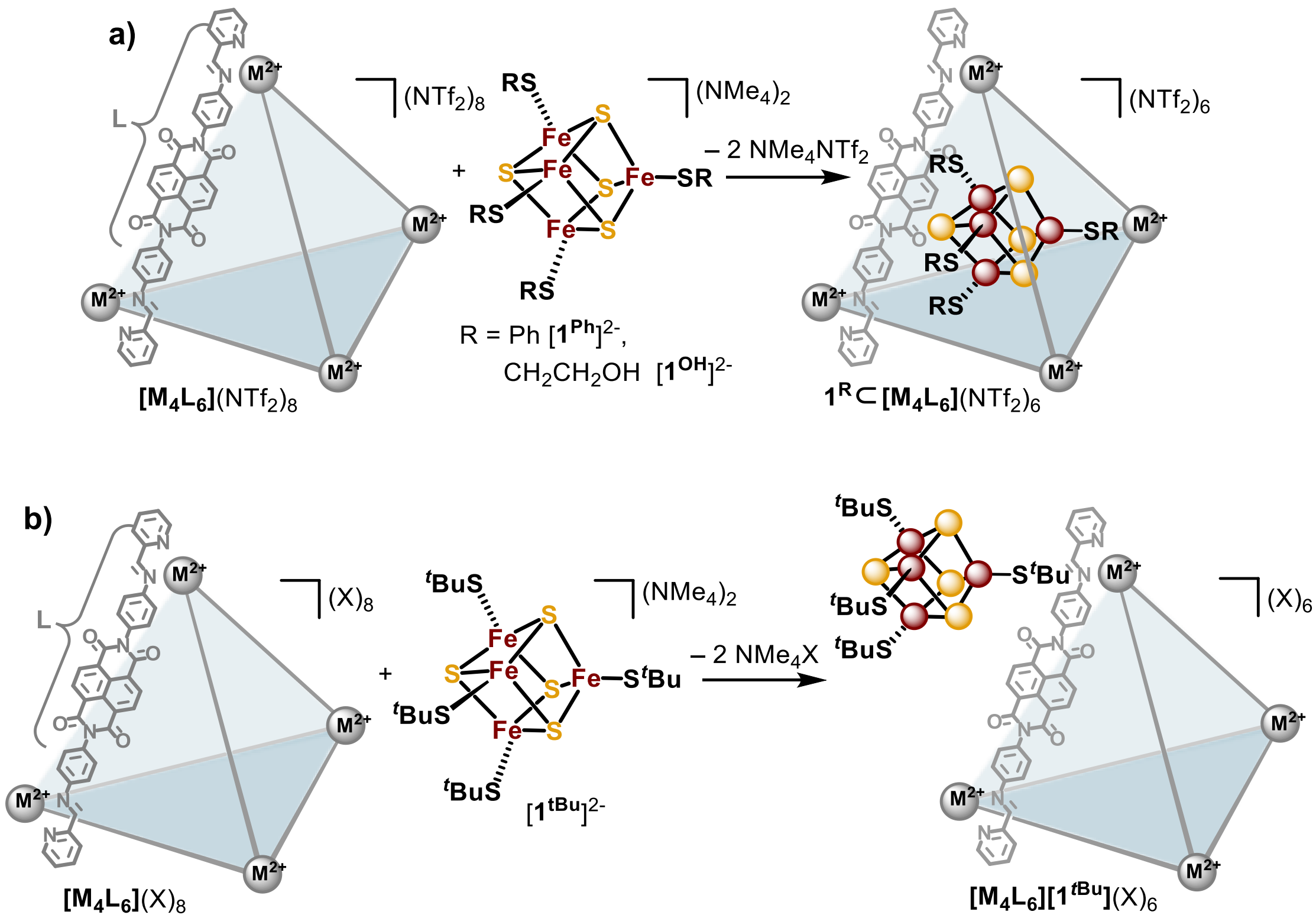

Scheme S2. Synthesis of host-guest complexes a) $\mathbf{1^R\subset[M_4L_6]}(NTf_2)_6$ and b) ion pair $\mathbf{[M_4L_6][1^{tBu}]}(X)_6$.

General procedure. The host-guest complexes $\mathbf{1^R\subset[M_4L_6]}(X)_6$ and ion pair $\mathbf{[M_4L_6][1^{tBu}]}(X)_6$ (M = Ni, Fe, Zn; X = NTf_2, OTf) were prepared on a 3.8 µmol scale by mixing $\mathbf{[M_4L_6]}(X)_8$ and $(NMe_4)_2\mathbf{[1^R]}$ in a 1:1 molar ratio in CD_3CN (500 µL) in the glovebox. Complexation was confirmed by a combination of ^{1}H NMR spectroscopy, ESI-MS, DOSY NMR spectroscopy, and ^{57}Fe Mössbauer spectroscopy (see sections S4 and S8 below). Representative stacked NMR spectra are provided below; full spectra are provided in Section S4.

$\mathbf{1^{Ph}\subset[Fe_4L_6]}(NTf_2)_6$. Mixing of a purple solution of $\mathbf{[Fe_4L_6]}(NTf_2)_8$ with a maroon solution of $(NMe_4)_2\mathbf{[1^{Ph}]}$ resulted in a color change to magenta.^{1}H NMR (400 MHz, CD_3CN; Figure S12), δ: 9.12−8.97 (12H, m, imine C*H*), 8.60−8.50 (36H, m, NDI and py C*H*), 8.43-8.38 (12H, m, py C*H*), 7.8-7.75 (12H, m, py C*H*), 7.56 (8H, s, SPh *m*-C*H*), 7.40−7.33 (36H, m, Ph and py C*H*), 6.04−5.3 (24H, m, Ph C*H*). 4.85 (4H, s, SPh *p*-C*H*), 4.29 (8H, s, SPh *o*-C*H*). ^{1}H DOSY NMR: (CD_3CN, 400 MHz, 25 °C; Figure S17): D = 4.2×10^{-10} m^2/s; r_H = 15.6 Å. ESI-MS (MeCN, m/z; Figure S32): 498.3 ($\mathbf{[Fe_4L_6]}^{8+}$, 14%), 609.3 ($\mathbf{[Fe_4L_6}(NTf_2)\mathbf{]}^{7+}$, 18%), 757.4 ($\mathbf{[Fe_4L_6}(NTf_2)_2\mathbf{]}^{6+}$, 18%), 795.5 ($\mathbf{1^{Ph}\subset[Fe_4L_6]}^{6+}$, 100%), 962.5 ($\mathbf{[Fe_4L_6}(NTf_2)_3\mathbf{]}^{5+}$, 5%), 1010.6 ($\mathbf{1^{Ph}\subset[Fe_4L_6}(NTf_2)\mathbf{]}^{5+}$, 21%).

$\mathbf{1^{Ph}\subset[Zn_4L_6]}(NTf_2)_6$. Mixing of a yellow solution of $\mathbf{[Zn_4L_6]}(NTf_2)_8$ with a maroon solution of $(NMe_4)_2\mathbf{[1^{Ph}]}$ resulted in a color change to brown. ^{1}H NMR (400 MHz, CD_3CN; Figure S13), δ: 8.79−8.67 (24H, m, imine and NDI C*H*), 8.50 (12H, br s, py C*H*), 8.33-8.07 (24H, m, py C*H*), 7.96 (12H, br s, py C*H*), 7.44 (8H, s, SPh *m*-C*H*), 7.38 (24H, br s, Ph C*H*), 6.21−6.05 (24H, m, Ph C*H*), 4.88 (4H, s, SPh *p*-C*H*), 4.39 (8H, s, SPh *o*-C*H*).^{1}H DOSY NMR: (CD_3CN, 400 MHz, 25 °C; Figure S18): D = 4.4×10^{-10} m^2/s; r_H = 14.6 Å.

$\mathbf{1^{Ph}\subset[Ni_4L_6]}(NTf_2)_6$. Mixing of the orange solution of $\mathbf{[Ni_4L_6]}(NTf_2)_8$ with a maroon solution of $(NMe_4)_2\mathbf{[1^{Ph}]}$ resulted in a color change to brown. ^{1}H NMR (400 MHz, CD_3CN; Figure S14), δ: 53.09, 19.68, 18.54, 16.12, 14.36, 13.44, 8.67, 7.55 (8H, s, SPh *m*-C*H*), 4.94 (4H, s, SPh *p*-C*H*), 4.45 (8H, s, SPh

o-C*H*), 3.07, -11.64. ESI-MS (MeCN, m/z; Figure S29): 499.3 (**$[Ni_4L_6]$**$^{8+}$, 5%), 610.7 (**$[Ni_4L_6$**$(NTf_2)]^{7+}$, 12%), 759.2 (**$[Ni_4L_6$**$(NTf_2)_2]^{6+}$, 7%), 797.3 (**$1^{Ph}\subset[Ni_4L_6]$**$^{6+}$, 100%), 967.0 (**$[Ni_4L_6$**$(NTf_2)_3]^{5+}$, 2%), 1012.9 (**$1^{Ph}\subset[Ni_4L_6$**$(NTf_2)]^{5+}$, 17%), 1278.8 (**$[Ni_4L_6$**$(NTf_2)_4]^{4+}$, 0.2%).

$1^{OH}\subset[Fe_4L_6]$ $(NTf_2)_6$. Mixing of a purple solution of **$[Fe_4L_6]$**$(NTf_2)_8$ with a green solution of $(NMe_4)_2$**$[1^{OH}]$** resulted in a color change to magenta. ^{1}H NMR (400 MHz, CD_3CN; Figure S19), δ: 9.06−8.98 (12H, m, imine *CH*), 8.75−8.56 (36H, m, NDI and py *CH*), 8.41 (12H, br s, py *CH*), 7.78 (12H, br s, py *CH*), 7.56−7.37 (36H, m, Ph and py *CH*), 6.07−5.28 (24H, m, Ph *CH*). 3.57 (8H, s, SCH_2CH_2OH), 2.58 (8H, s, SCH_2CH_2OH), 1.62 (4H, s, SCH_2CH_2OH). ESI-MS (MeCN, m/z; Figure S33): 498.1 (**$[Fe_4L_6]$**$^{8+}$, 90%), 609.3 (**$[Fe_4L_6$**$(NTf_2)]^{7+}$, 100%), 757.5 (**$[Fe_4L_6$**$(NTf_2)_2]^{6+}$, 93%) 962.4 (**$[Fe_4L_6$**$(NTf_2)_3]^{5+}$, 18%), 1282.7 (**$[Fe_4L_6$**$(NTf_2)_4]^{4+}$, 0.3%). The peaks for the host-guest complex were not observed due to the lability of **$[1^{OH}]^{2-}$** due to its small size (packing coefficient = 0.39).

$1^{OH}\subset[Zn_4L_6]$ $(NTf_2)_6$. Mixing a yellow solution of **$[Zn_4L_6]$**$(NTf_2)_8$ with a green solution of $(NMe_4)_2$**$[1^{OH}]$** resulted in a color change to brown. ^{1}H NMR (400 MHz, CD_3CN; Figure S20), δ: 8.73−8.66 (24H, m, imine and NDI C*H*), 8.50 (12H, br s, py C*H*), 8.28-8.05 (24H, m, py C*H*), 7.96 (12H, br s, py C*H*), 7.48-7.38 (24H, m, Ph C*H*), 6.34−6.05 (24H, m, Ph C*H*), 3.57 (8H, s, SCH_2CH_2OH), 2.59 (8H, s, SCH_2CH_2OH), 1.63 (4H, s, SCH_2CH_2OH). ^{1}H DOSY NMR: (CD_3CN, 400 MHz, 25 °C; Figure S23): D = 3.4×10^{-10} m^2/s; r_H = 18.7 Å.

$1^{OH}\subset[Ni_4L_6]$ $(NTf_2)_6$. Mixing of the orange solution of **$[Ni_4L_6]$**$(NTf_2)_8$ with a green solution of $(NMe_4)_2$**$[1^{OH}]$** resulted in a color change to brown. ^{1}H NMR (400 MHz, CD_3CN; Figure S21), δ: 53.52, 19.87, 18.45, 16.11, 14.27, 13.45, 8.74, 3.56 (8H, s, SCH_2CH_2OH), 2.59 (8H, s, SCH_2CH_2OH), 1.62 (4H, s, SCH_2CH_2OH), -11.78.

$[Fe_4L_6][1^{tBu}]$ $(X)_6$. Mixing of a purple solution of **$[Fe_4L_6]$**$(X)_8$ with a green solution of $(NMe_4)_2$**$[1^{tBu}]$** resulted in a color change to magenta. ^{1}H NMR (400 MHz, CD_3CN; Figure S24, X = OTf), δ: 9.05 (12H, br s, imine *CH*), 8.60−8.57 (36H, m, NDI and py *CH*), 8.42 (12H, br s, py *CH*), 7.79 (12H, br s, py *CH*), 7.55−7.37 (36H, m, Ph and py *CH*), 6.07−5.29 (24H, m, Ph *CH*). 2.61 (36H, br s, S^tBu CH_3). ESI-MS (MeCN, m/z; Figure S35, X = NTf_2): 498.1 (**$[Fe_4L_6]$**$^{8+}$, 50%), 609.2 (**$[Fe_4L_6$**$(NTf_2)]^{7+}$, 95%), 757.5 (**$[Fe_4L_6$**$(NTf_2)_2]^{6+}$, 100%), 782.3 (**$[Fe_4L_6][1^{tBu}]$**$^{6+}$, 22%), 962.4 (**$[Fe_4L_6$**$(NTf_2)_3]^{5+}$, 9%), 994.6 (**$[Fe_4L_6][1^{tBu}]$**$(NTf_2)]^{5+}$, 39%), 1283 (**$[Fe_4L_6$**$(NTf_2)_4]^{4+}$, 2%). ^{1}H DOSY NMR: (CD_3CN, 400 MHz, 25 °C; Figure S27, X = OTf): D = 3.6×10^{-10} m^2/s; r_H = 17.5 Å. The $SC(CH_3)_3$ ^{1}H NMR signal is only slightly shifted relative to unencapsulated **$[1^{tBu}]^{2-}$** (δ_H 2.66) indicating that the dispersion forces between **$[M_4L_6]^{8+}$** and **$[1^{tBu}]^{2-}$** are relatively weak. Nevertheless, the ^{57}Fe Mössbauer data support encapsulation of **$[1^{tBu}]^{2-}$** (Section S8).

$[Ni_4L_6][1^{tBu}]$ $(NTf_2)_6$. Mixing of an orange solution of **$[Ni_4L_6]$**$(NTf_2)_8$ with a green solution of $(NMe_4)_2$**$[1^{tBu}]$** resulted in a color change to brown. ^{1}H NMR (400 MHz, CD_3CN; Figure S25), δ: 53.18, 19.84, 18.44, 16.12, 14.25, 13.40, 8.73, 2.59 (36H, br s, S^tBu CH_3), -11.88.

Attempted synthesis of $[Zn_4L_6][1^{tBu}]$ $(NTf_2)_6$. Mixing of a yellow solution of **$[Zn_4L_6]$**$(NTf_2)_8$ with a green solution of $(NMe_4)_2$**$[1^{tBu}]$** resulted in the solution becoming turbid with a color change to brown. ^{1}H NMR spectroscopy of the brown filtered solution revealed only peaks for the free cage without any signals for **$[1^{tBu}]^{2-}$**, precluding confirmation of encapsulation. ^{1}H NMR (400 MHz, CD_3CN; Figure S20), δ: 8.73−8.66 (24H, m, imine and NDI C*H*), 8.50 (12H, br s, py C*H*), 8.28-8.05 (24H, m, py C*H*), 7.96 (12H, br s, py C*H*), 7.48-7.38 (24H, m, Ph C*H*), 6.34−6.05 (24H, m, Ph C*H*).

Synthesis of reduced host-guest complexes $1^R \subset [Zn_4L_6]$ and ion pair $[Ni_4L_6][1^{tBu}]$

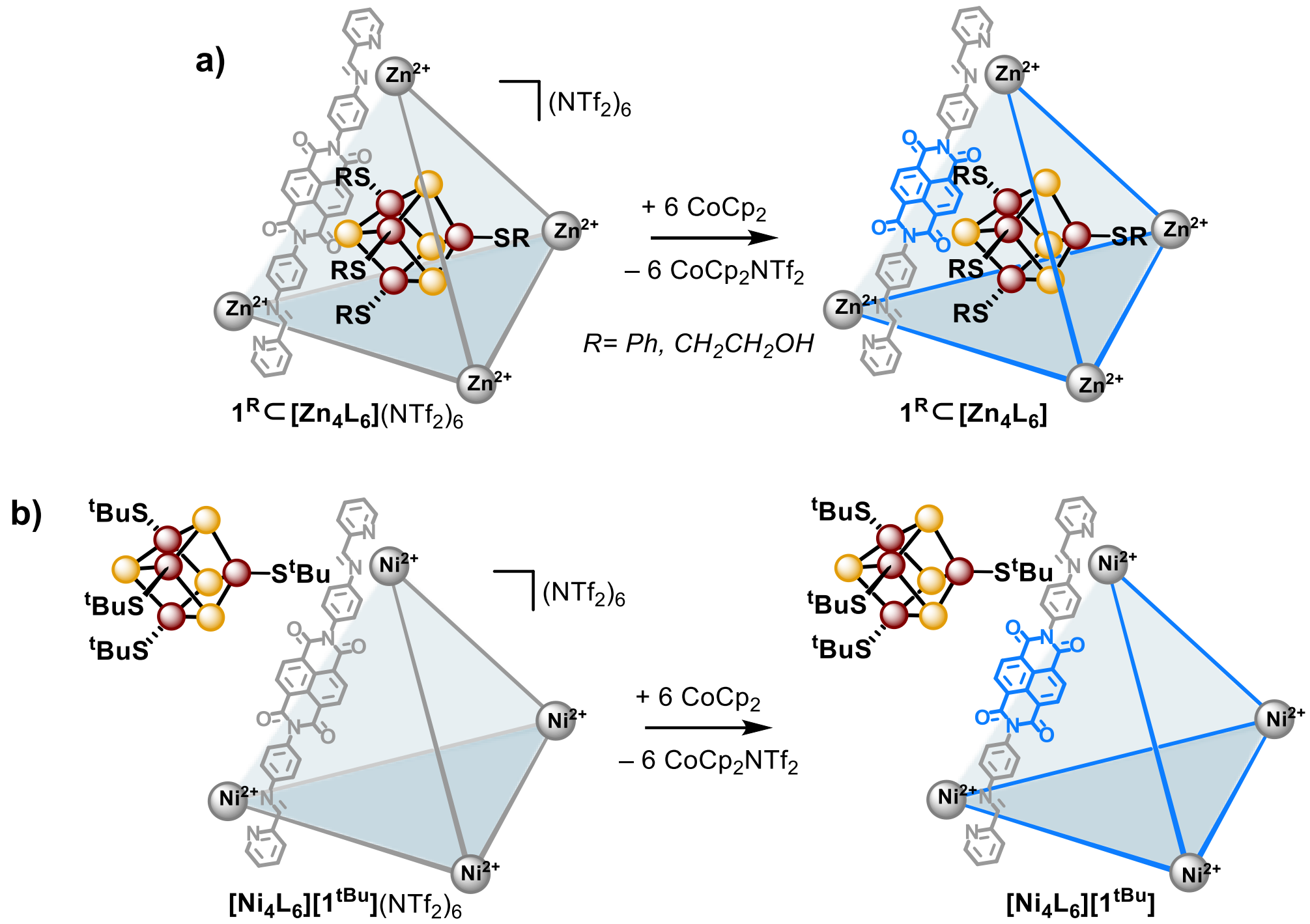

Scheme S3. Preparation of a) $\mathbf{1^R \subset [Zn_4L_6]}$ and b) $\mathbf{[Ni_4L_6][1^{tBu}]}$ via reduction of $\mathbf{1^R \subset [Zn_4L_6]}(NTf_2)_6$ and $\mathbf{[Ni_4L_6][1^{tBu}]}(NTf_2)_6$, respectively with $CoCp_2$

Representative procedure. The reduced host-guest complexes $\mathbf{1^R \subset [Zn_4L_6]}$ and ion pair $\mathbf{[Ni_4L_6][1^{tBu}]}$ were synthesized from in situ-generated $\mathbf{1^R \subset [Zn_4L_6]}(NTf_2)_6$ and $\mathbf{[Ni_4L_6][1^{tBu}]}(NTf_2)_6$ prepared as described above. $\mathbf{[M_4L_6]}(X)_8$ and $(NMe_4)_2$ $\mathbf{[1^R]}$ (each 38 mmol) were combined in CH_3CN (15 mL) and stirred for 2 h. $CoCp_2$ (44 mg, 230 µmol, 6 equiv) was added and the reaction mixture was stirred for an additional 2 h, yielding a dark brown solution. After concentrating in vacuo to dryness, the residue was re-dissolved in CH_3CN (5 mL) and precipitated at 0 °C by adding Et_2O (15 mL). The precipitate was filtered, washed with copious 1:1 Et_2O/CH_3CN, and dried in vacuo yielding the reduced host-guest complexes $\mathbf{1^R \subset [M_4L_6]}(X)_6$ and ion pair $\mathbf{[Ni_4L_6][1^{tBu}]}$ as crystalline brown solids. Characterization by ^{1}H and DOSY NMR and ESI-MS was precluded by insolubility and instability in more polar solvents (DMSO).

$\mathbf{1^{Ph} \subset [Zn_4L_6]}$. ^{57}Fe Mössbauer spectroscopy (0 T, 200 K, Figure S64): δ 0.44, 0.53; ΔE_Q 0.82, 1.26 (1:1)

$\mathbf{1^{OH} \subset [Zn_4L_6]}$. ^{57}Fe Mössbauer spectroscopy (0 T, 200 K, Figure S67): δ 0.40, 0.47; ΔE_Q 0.80, 1.27 (1:1)

$\mathbf{[Ni_4L_6][1^{tBu}]}$. ^{57}Fe Mössbauer spectroscopy (0 T, 200 K, Figure S70): δ 0.38, 0.40; ΔE_Q 0.68, 1.01 (1:1)

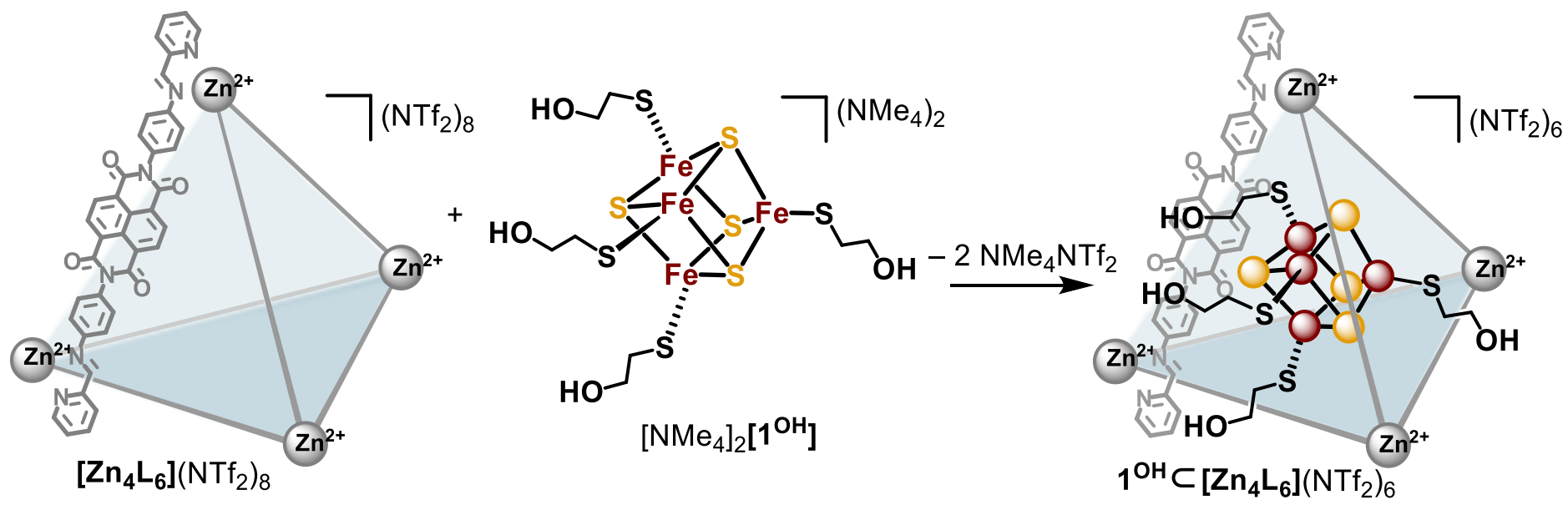

Scheme S4. Synthesis of $\mathbf{1^{OH}}$⊂**[Zn$_4$L$_6$]**(NTf$_2$)$_6$.

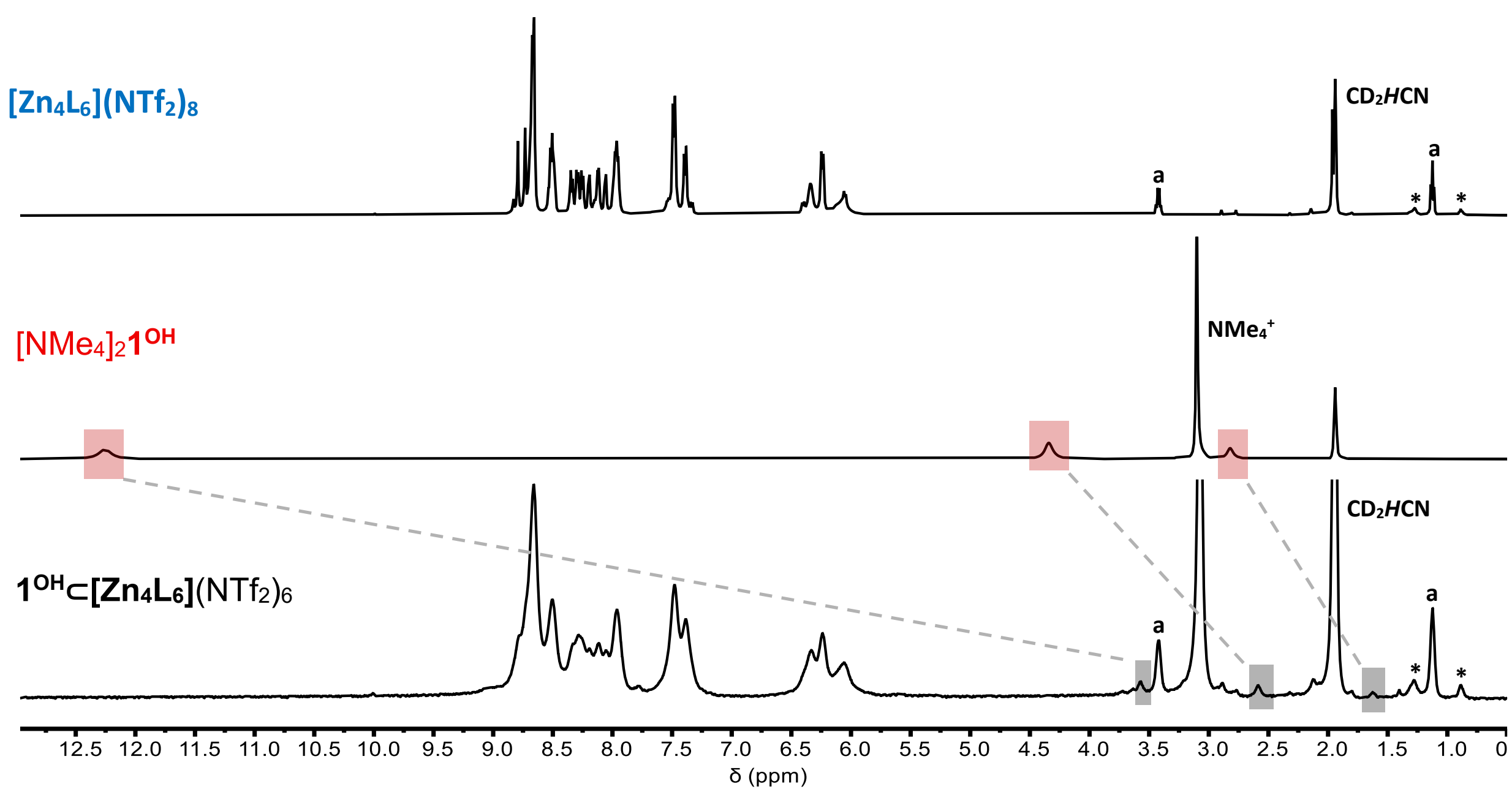

Figure S3. ^{1}H NMR spectra (400 MHz, CD$_3$CN) of **[Zn$_4$L$_6$]**(NTf$_2$)$_8$, [NMe$_4$]$_2$$\mathbf{1^{OH}}$, and $\mathbf{1^{OH}}$⊂**[Zn$_4$L$_6$]**(NTf$_2$)$_6$. Upfield shifted peaks in the complex are indicated with dashed lines ([a]residual Et$_2$O; *unidentified impurities).

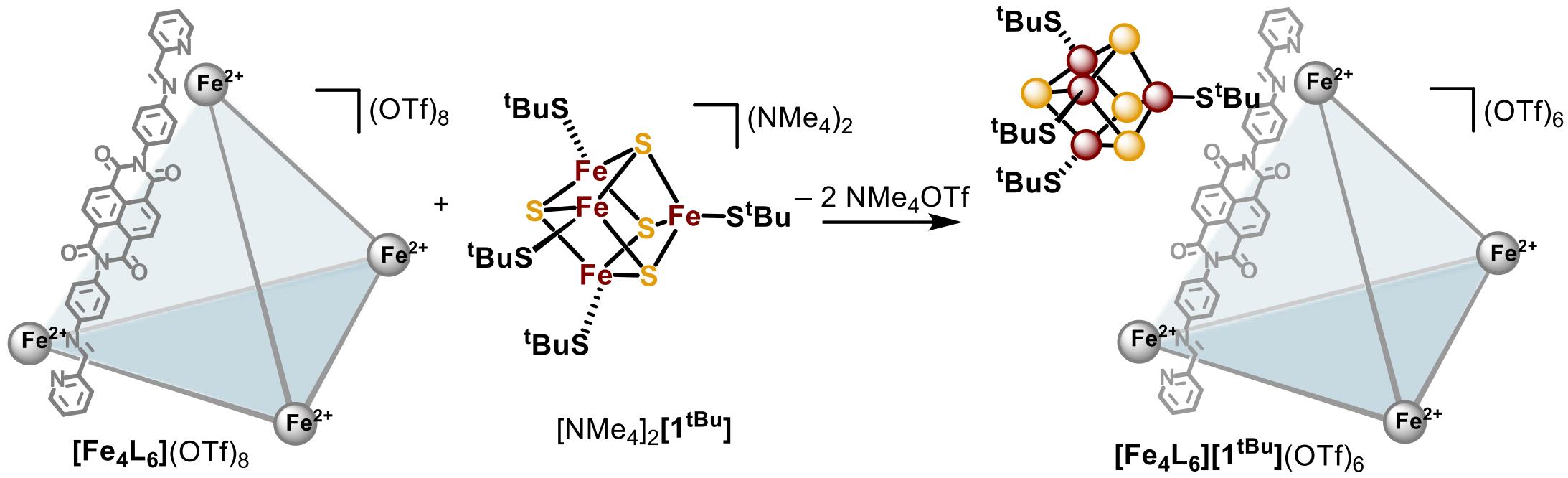

Scheme S5. Synthesis of **$[Fe_4L_6][1^{tBu}]$**$(OTf)_6$.

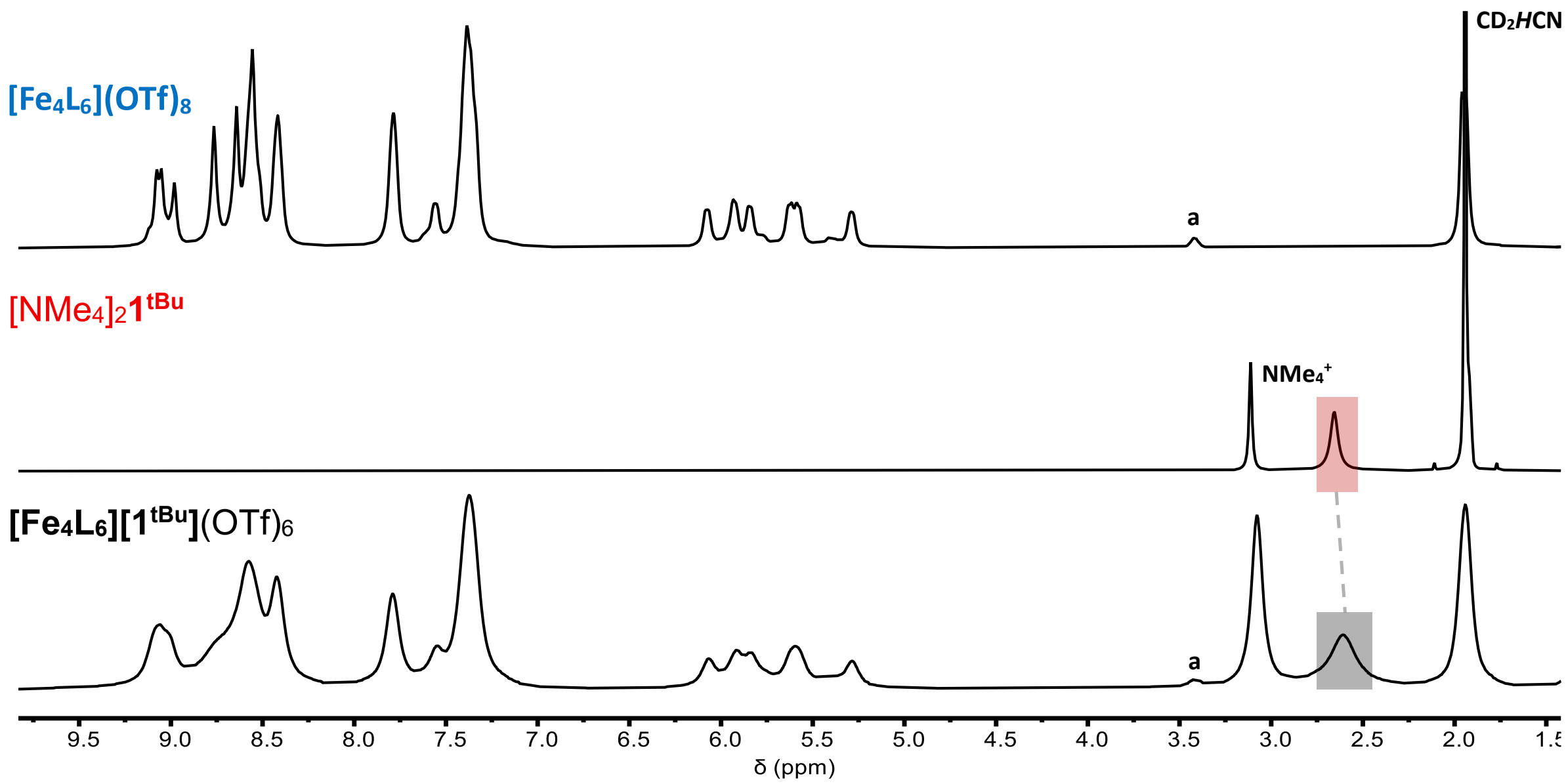

Figure S4. ^{1}H NMR spectra (400 MHz, CD_3CN) of **$[Fe_4L_6]$**$(OTf)_8$, $[NMe_4]^2$**$[1^{tBu}]$**, and **$[Fe_4L_6][1^{tBu}]$**$(OTf)_6$. Upfield shifted peaks for encapsulated **$[1^{tBu}]^{2-}$** are indicated with dashed lines (^{a}residual Et_2O).

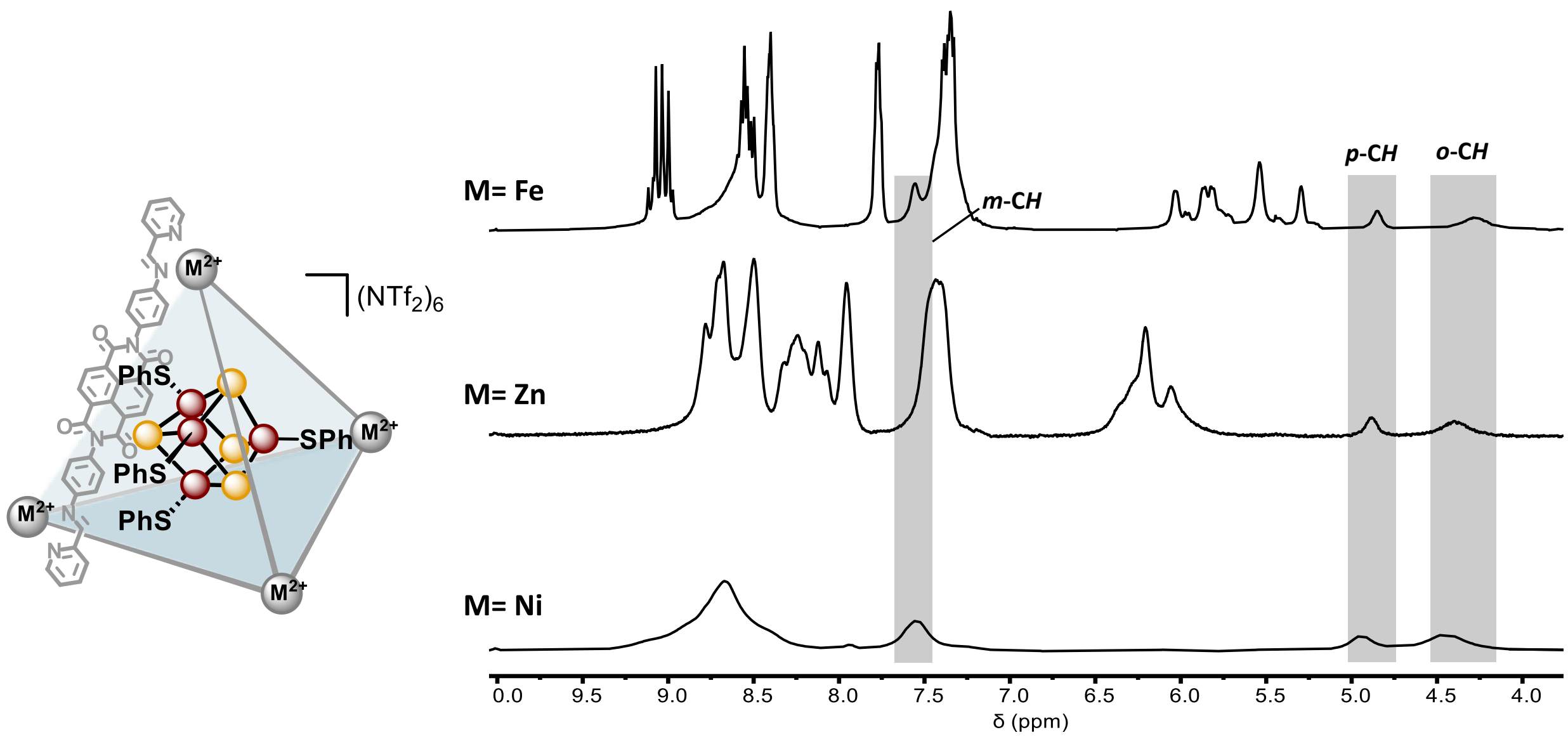

Figure S5. ^{1}H NMR spectra (400 MHz, CD_3CN) of $\mathbf{1^{Ph}{\subset}[M_4L_6]}(NTf_2)_6$ (M = Fe, Zn, Ni) showing that the chemical shifts for the encapsulated cluster $\mathbf{[1^{Ph}]^{2-}}$ thiolate ligands (grey boxes) are minimally affected by the identity of the corner ions.

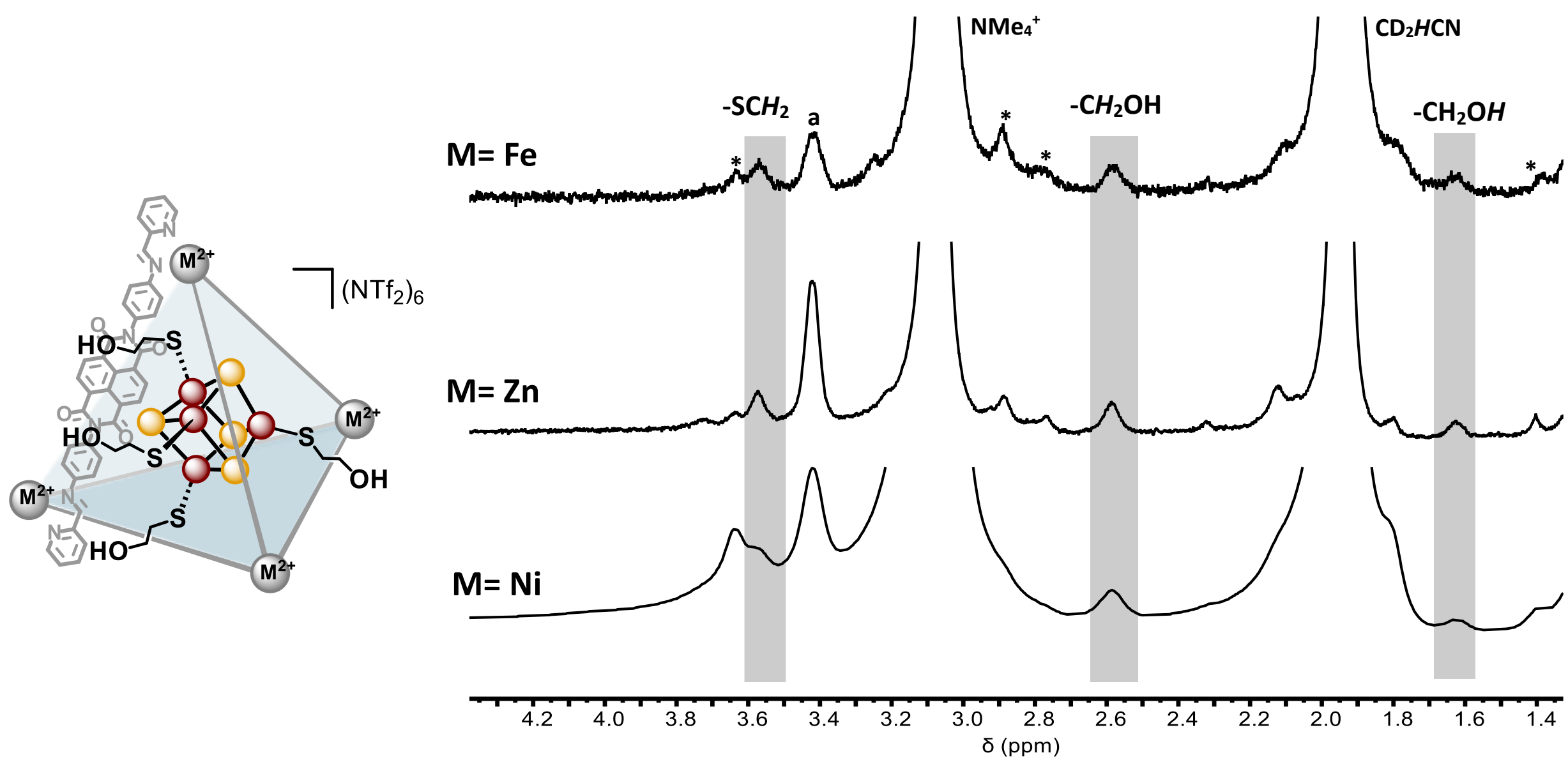

Figure S6. ^{1}H NMR spectra (400 MHz, CD_3CN) of $\mathbf{1^{OH}{\subset}[M_4L_6]}(NTf_2)_6$ (M = Fe, Zn, Ni) showing that the chemical shifts for the encapsulated cluster $\mathbf{[1^{OH}]^{2-}}$ thiolate ligands (grey boxes) are minimally affected by the identity of the corner ions ([a]residual Et_2O; *unidentified impurities).

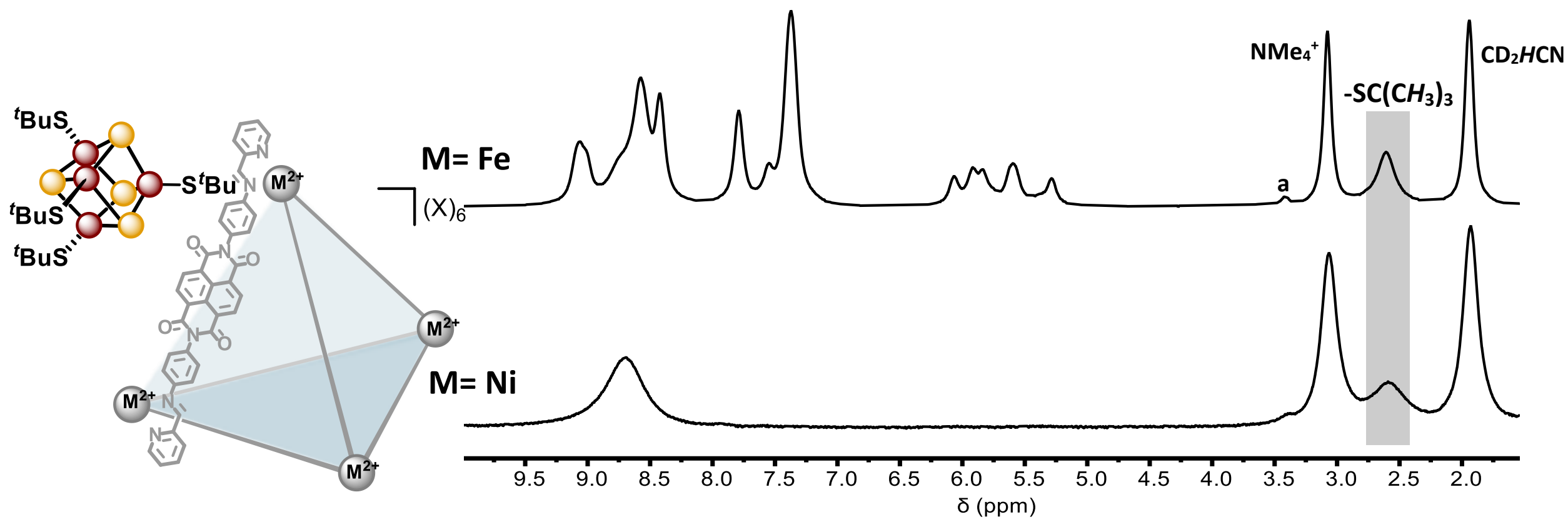

Figure S7. ^{1}H NMR spectra (400 MHz, CD_3CN) of **$[M_4L_6][1^{tBu}]$**$(X)_6$ (M = Fe, Ni; X= OTf for Fe and NTf_2 for Ni) showing that the chemical shifts for the encapsulated cluster **$[1^{tBu}]^{2-}$** thiolate ligands (grey boxes) are minimally affected by the identity of the corner ions (^{a} esidual Et_2O).

S4. NMR SPECTROSCOPY

^{1}H DOSY NMR spectra were recorded using a Bruker Avance III 500 MHz spectrometer. Experiments were performed at 298 K without spinning of the NMR tube. For each sample, a series of experiments were conducted to determine the most accurate parameters. This involved determining the 90° pulse for each complex and completing a T1 estimation. The T1 estimation was completed with pulse sequence t1ir1d where the D7 parameter was varied to find the null position of the peaks of the complex, then T1 = D7 / ln2. Finally, the DOSY parameters for gradient pulse duration and diffusion delay time (p30 and d20, respectively) were optimized by trial and error to achieve approx. 95% decay of the NMR signals of the metal complex over the course of the experiment. ^{1}H DOSY NMR spectra of each complex was recorded using a pulsed-field gradient stimulated echo (PFGSTE) sequence with bipolar gradients using the optimized parameters determined from the above experiments (Bruker: ledbpgp2s). Each experiment consisted of 19 gradient strength increments with 8 or 16 scans accumulated per increment. To obtain accurate diffusion coefficients (D), the diffusion decay curves of individual peaks were fit to a diffusion curve using the Bruker dynamics center, T1/T2 package of 4.3.0 Bruker TopSpin program and the diffusion coefficients (D) were obtained from the slope. 2D DOSY plots were processed using the Bayesian method of the DOSY transform in Mestrenova (v14.2.0-26256).

Calculation of hydrodynamic radii. The hydrodynamic radii r_H of solvated species were calculated from the measured diffusion coefficients D according to the Stokes-Einstein equation (Equation S1), where as part of this analysis, dissolved analyte molecules are assumed to be spherical:

$$D = \frac{k_B\, T}{6\pi\, \eta\, r_H} \qquad \textbf{(Equation S1)}$$

In Equation S1, k_B is the Boltzmann constant (1.38 x 10^{-23} J K^{-1}), T is temperature (298 K), and η is the viscosity of the solvent (0.369 mPa•s for acetonitrile).

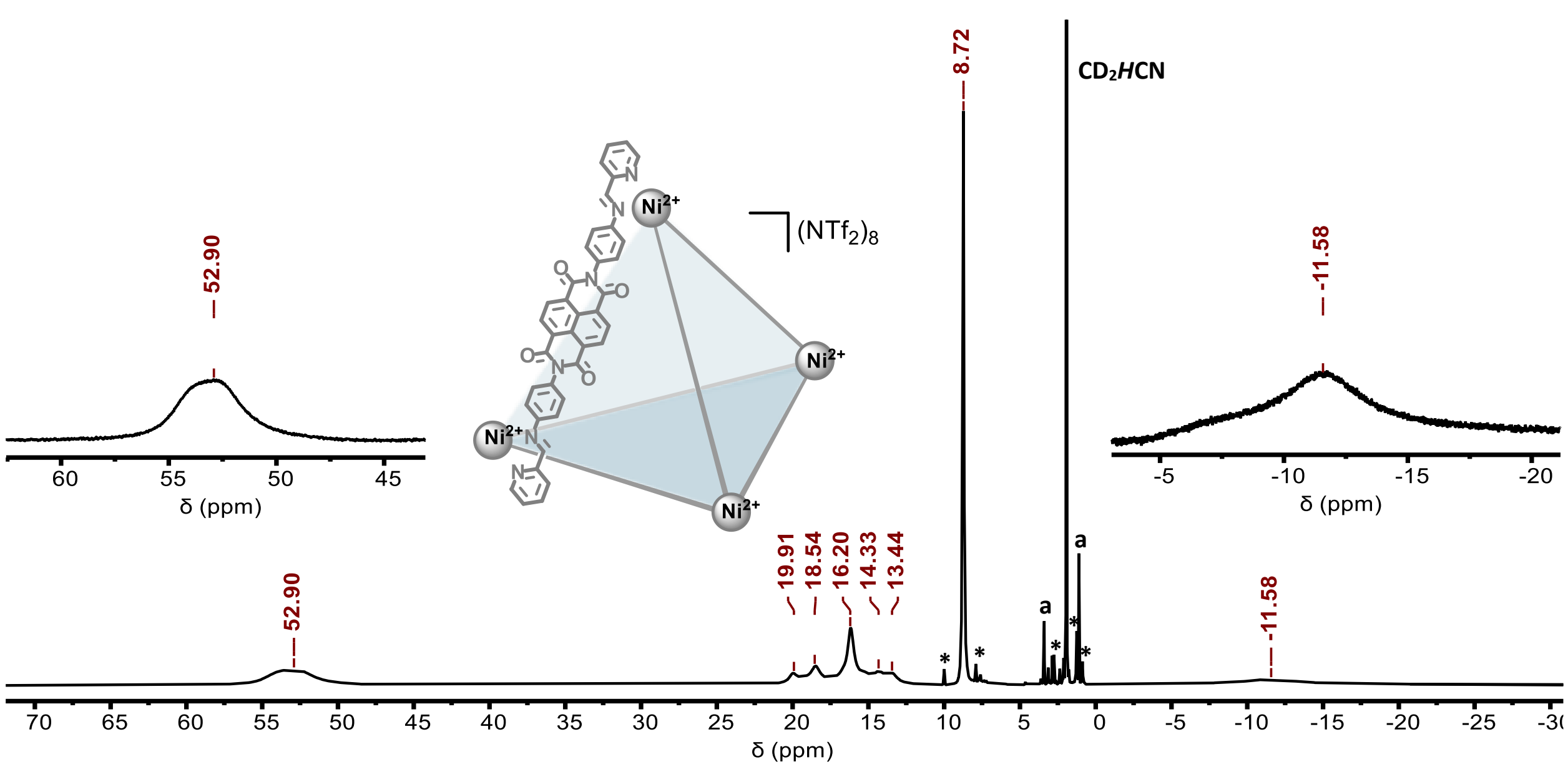

Figure S8. ^{1}H NMR spectrum (400 MHz, CD_3CN) of $\mathbf{[Ni_4L_6]}(NTf_2)_8$ (^{a} residual Et_2O; *unidentified impurities).

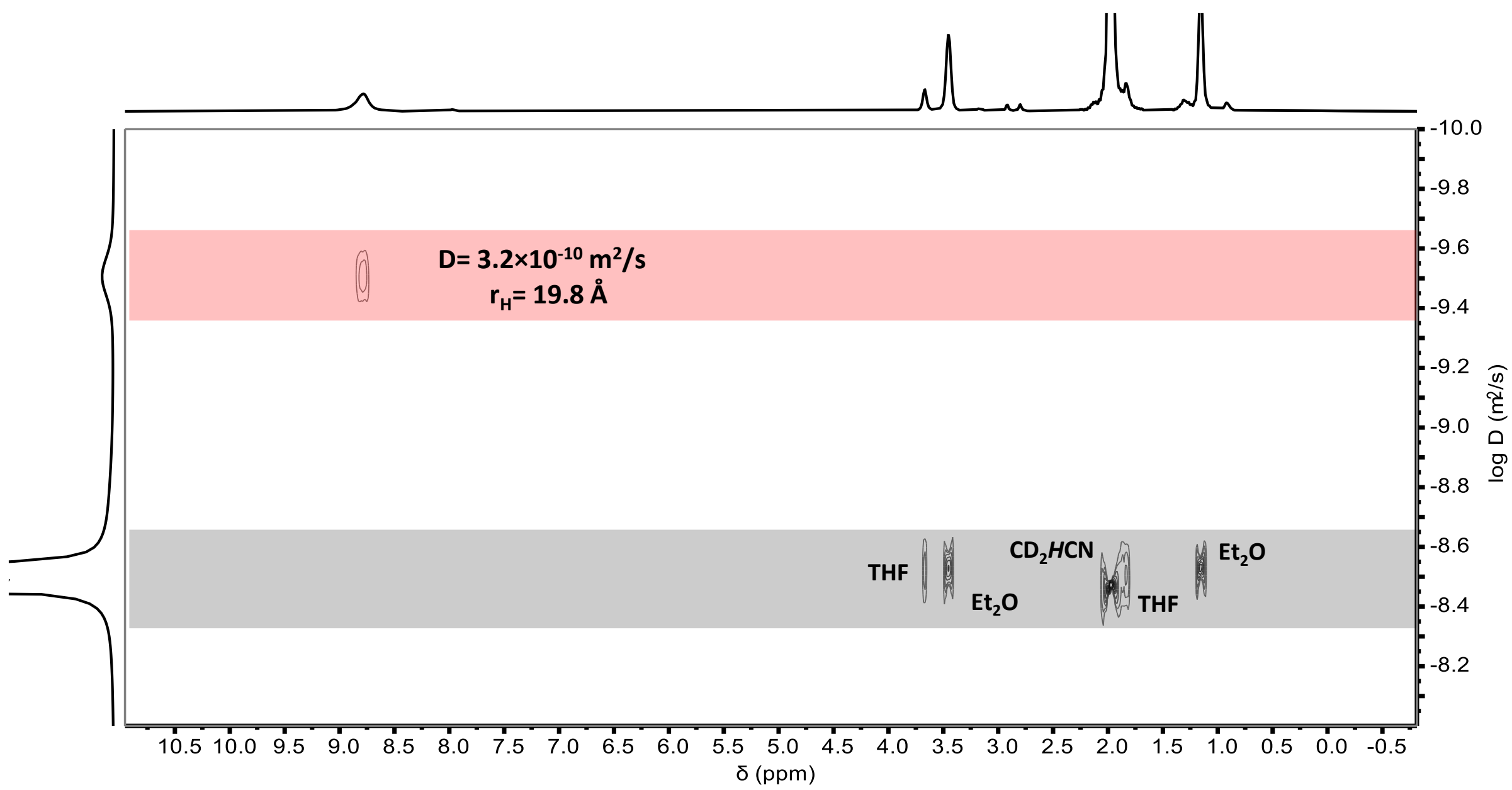

Figure S9. ^{1}H DOSY NMR spectrum (400 MHz, CD_3CN) of **$[Ni_4L_6]$**$(NTf_2)_8$ (diamagnetic region) showing a diffusion coefficient of 3.2×10^{-10} m^2/s (r_H = 19.8 Å) for the signal at 8.72 ppm. DOSY correlations to the other paramagnetic signals are absent due to the fast relaxation times and signal broadness, as observed previously for a mononuclear high-spin Fe(II) complex.[10]

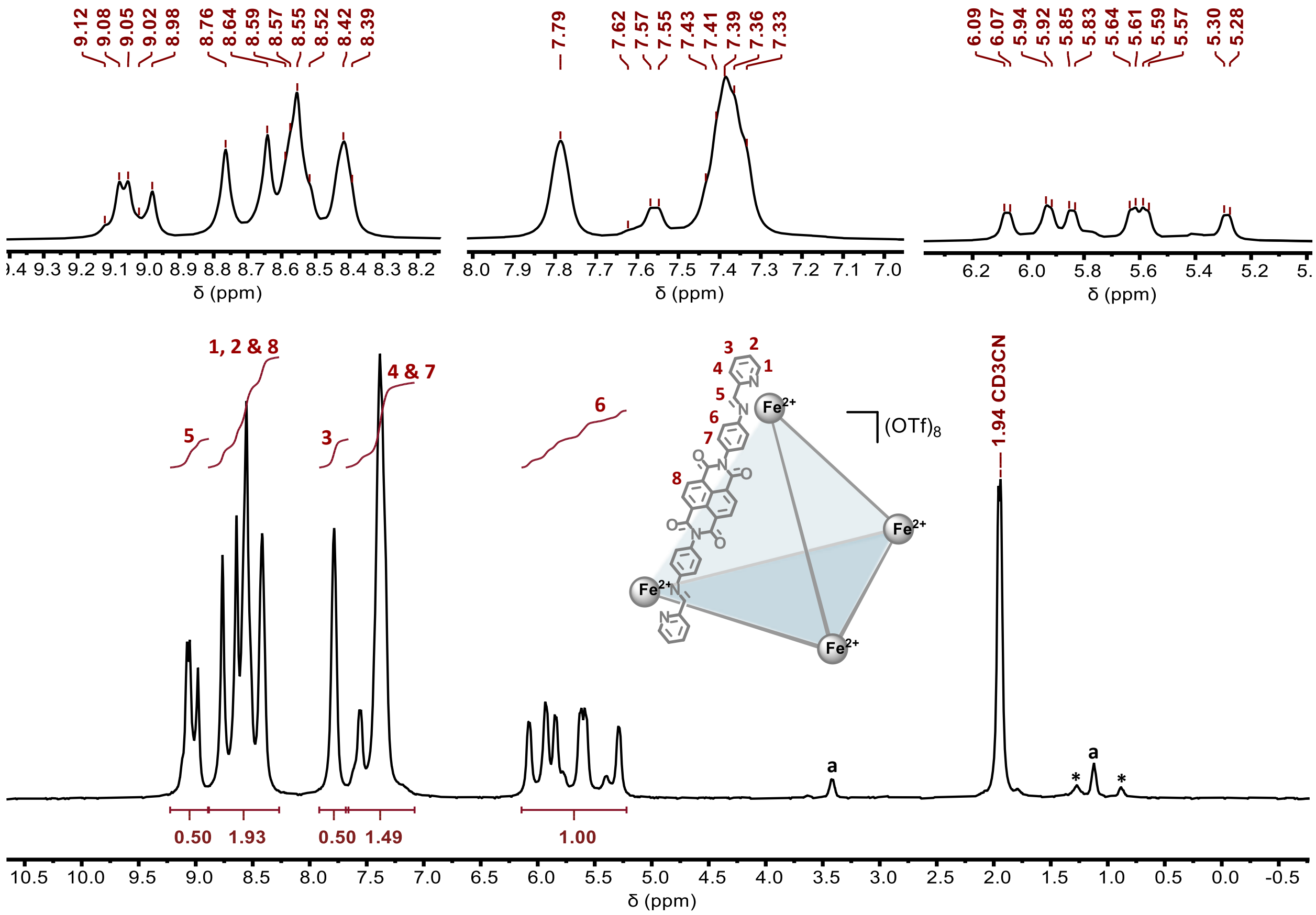

Figure S10. ^{1}H NMR spectrum (400 MHz, CD_3CN) of **[Fe$_4$L$_6$]**(OTf)$_8$ (^{a} residual Et_2O; *unidentified impurities).

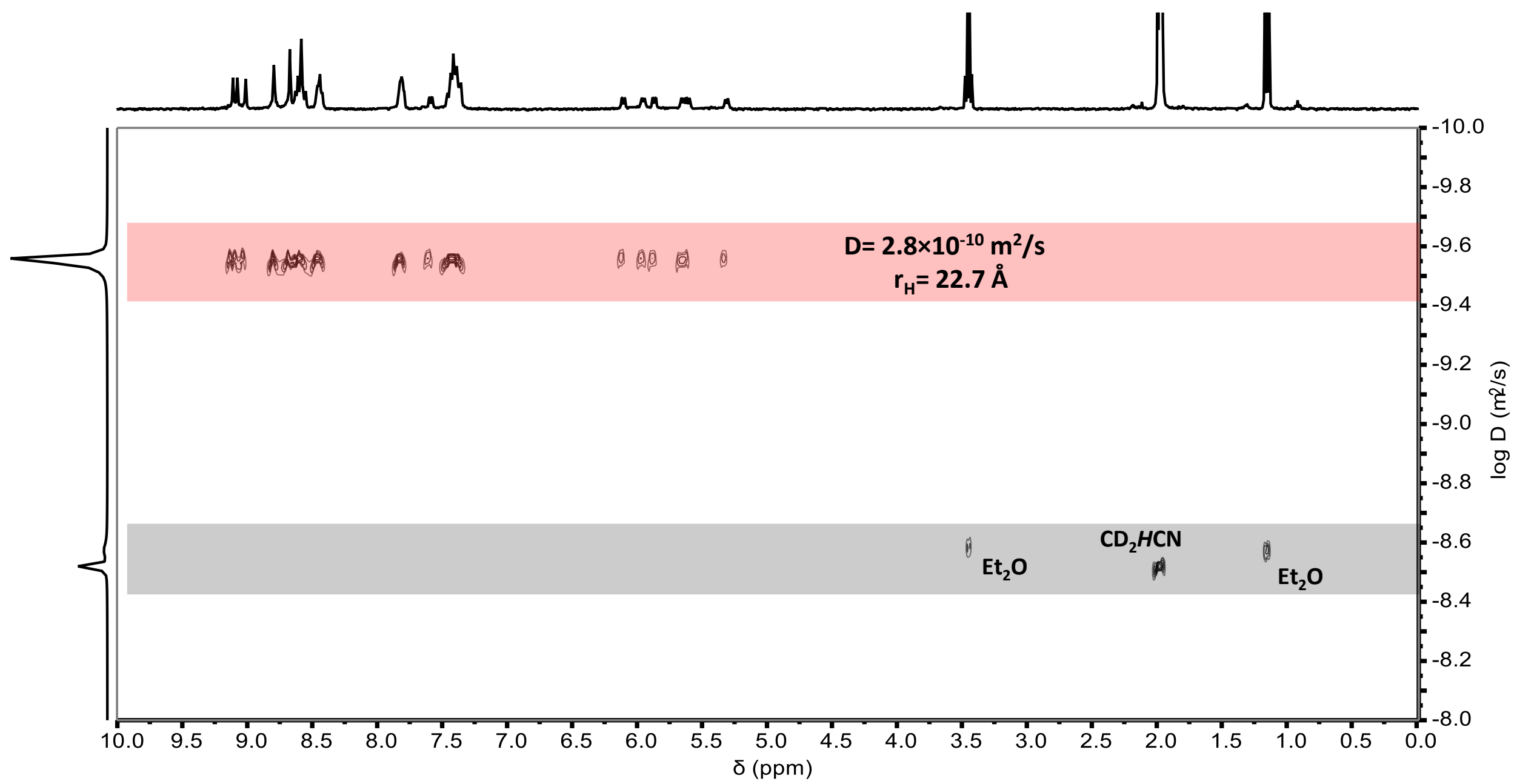

Figure S11. ^{1}H DOSY NMR spectrum (400 MHz, CD_3CN) of **[Fe_4L_6]**$(OTf)_8$ showing correlation of the cage signals to a diffusion coefficient of 2.8×10^{-10} m^2/s (r_H = 22.7 Å).

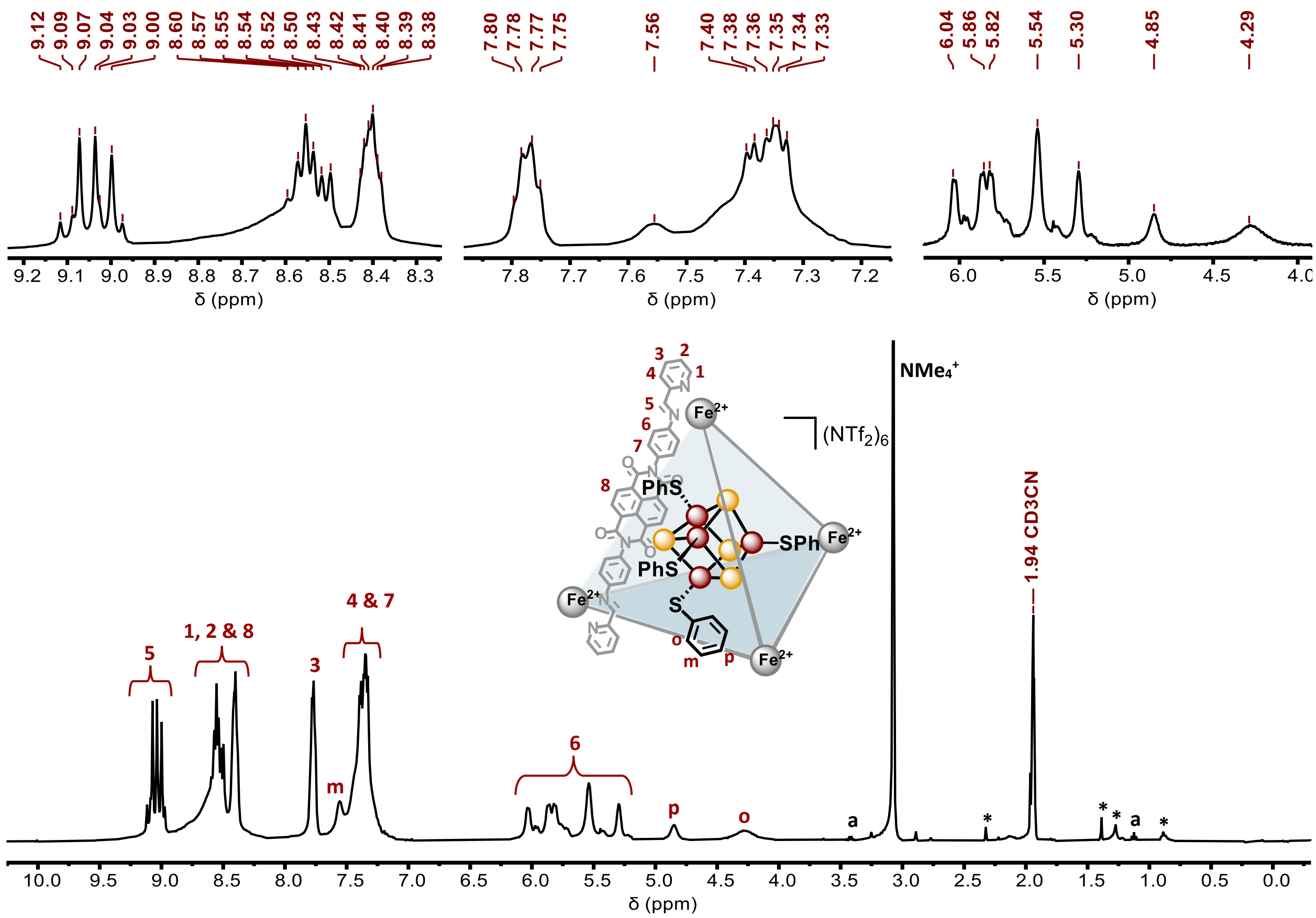

Figure S12. ^{1}H NMR spectrum (400 MHz, CD_3CN) of **1^{Ph}⊂[Fe_4L_6]**$(NTf_2)_6$ (^{a} residual Et_2O; *unidentified impurities).

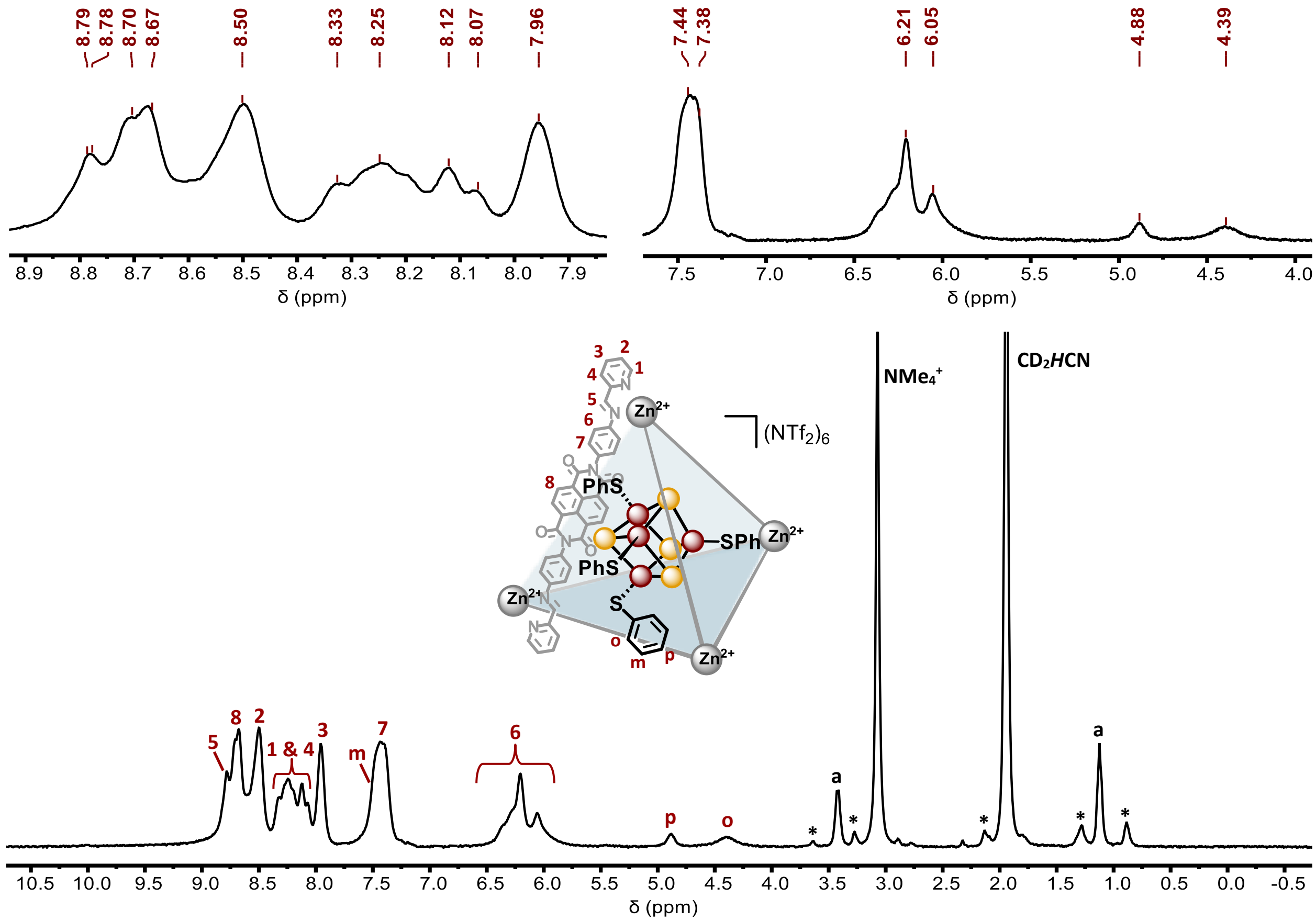

Figure S13. ^{1}H NMR spectrum (400 MHz, CD_3CN) of **1^{Ph}⊂[Zn$_4$L$_6$]**(NTf$_2$)$_6$ (^{a} residual Et_2O; *unidentified impurities).

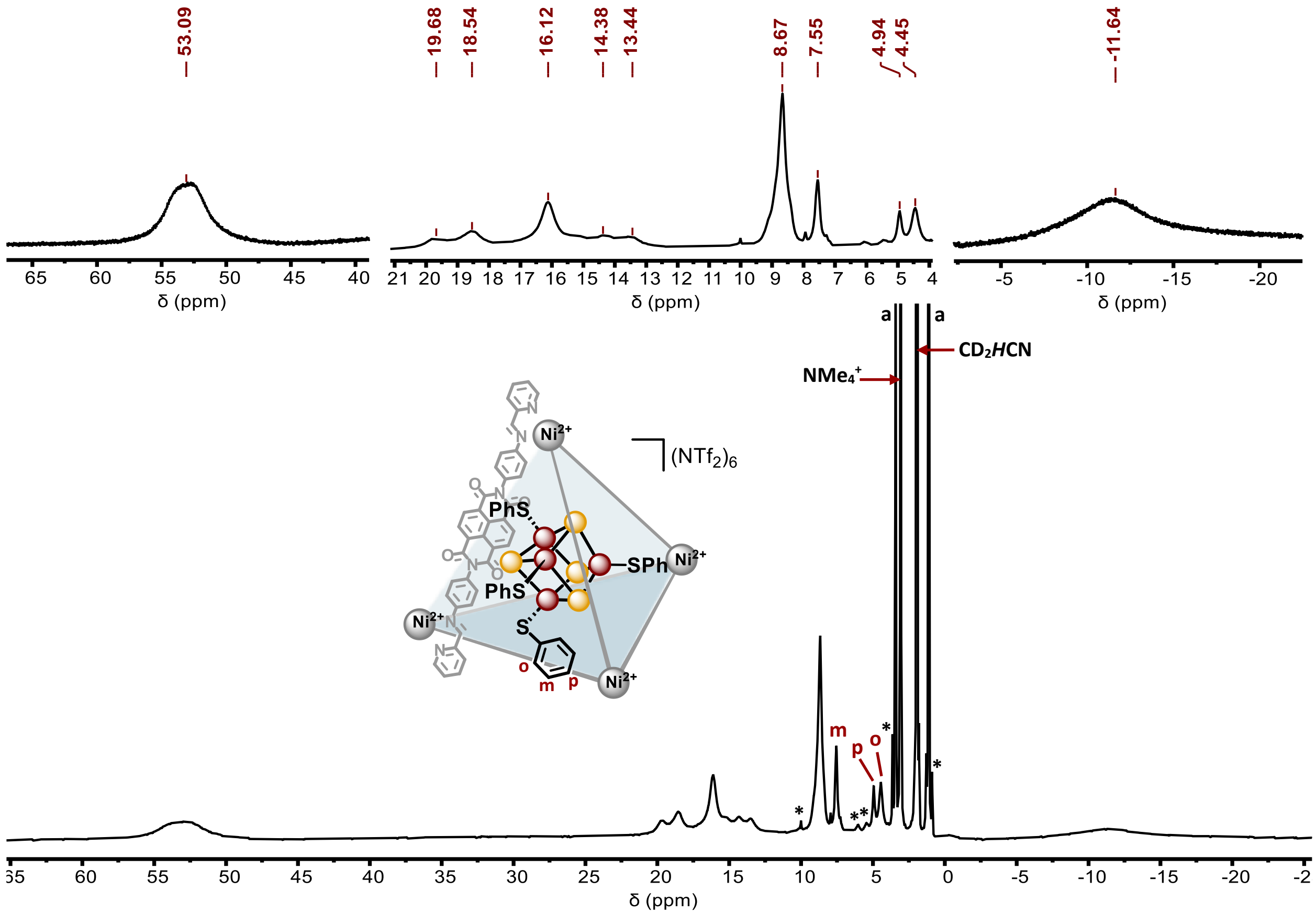

Figure S14. ^{1}H NMR spectrum (400 MHz, CD_3CN) of $\mathbf{1^{Ph}\subset[Ni_4L_6]}(NTf_2)_6$ ([a] residual Et_2O; *unidentified impurities).

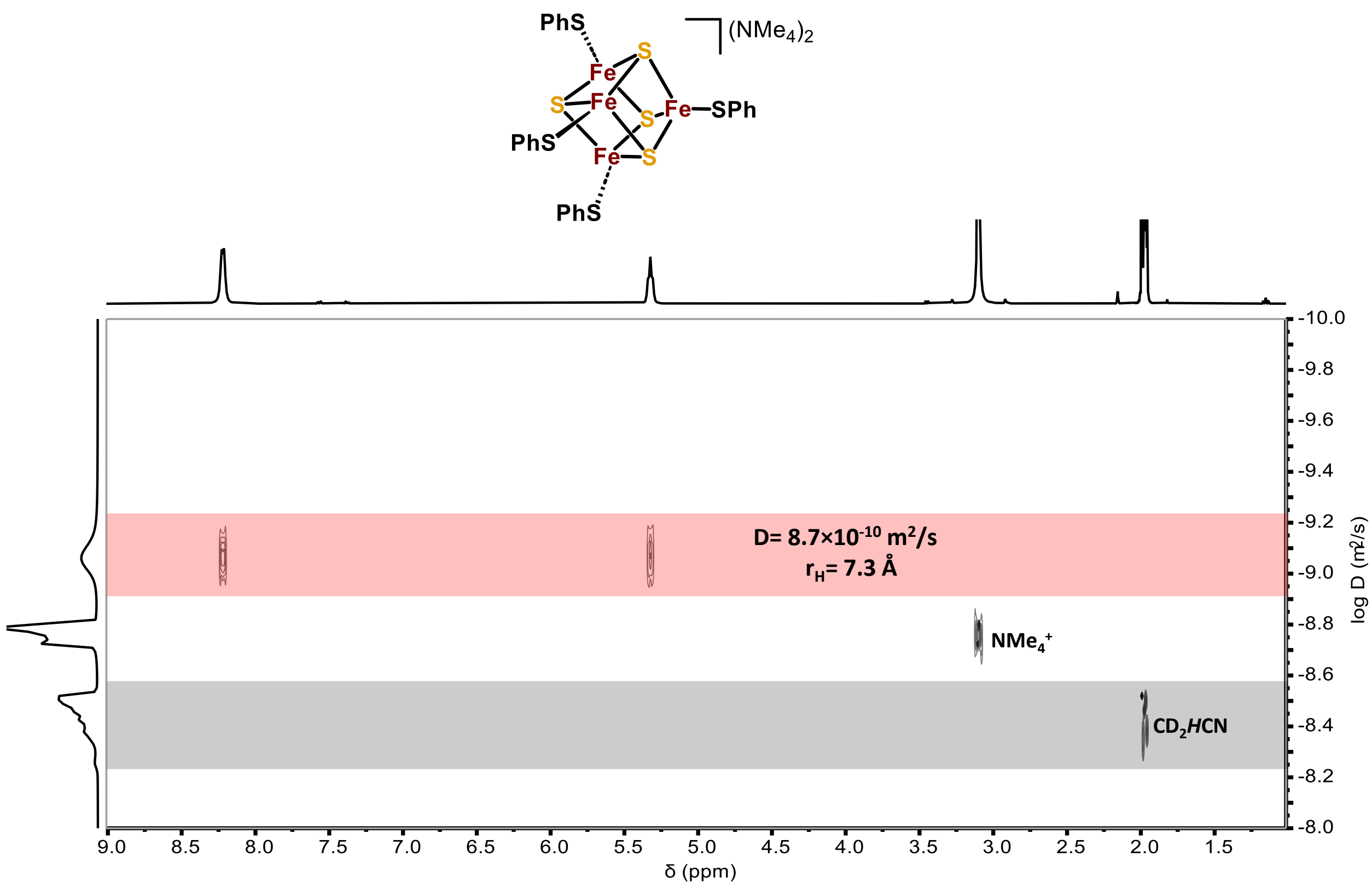

Figure S15. ^{1}H DOSY NMR spectrum (400 MHz, CD_3CN) of $(NMe_4)_2$**[1^{Ph}]**, showing a diffusion coefficient of 8.7×10^{-10} m^2/s (r_H = 7.3 Å).

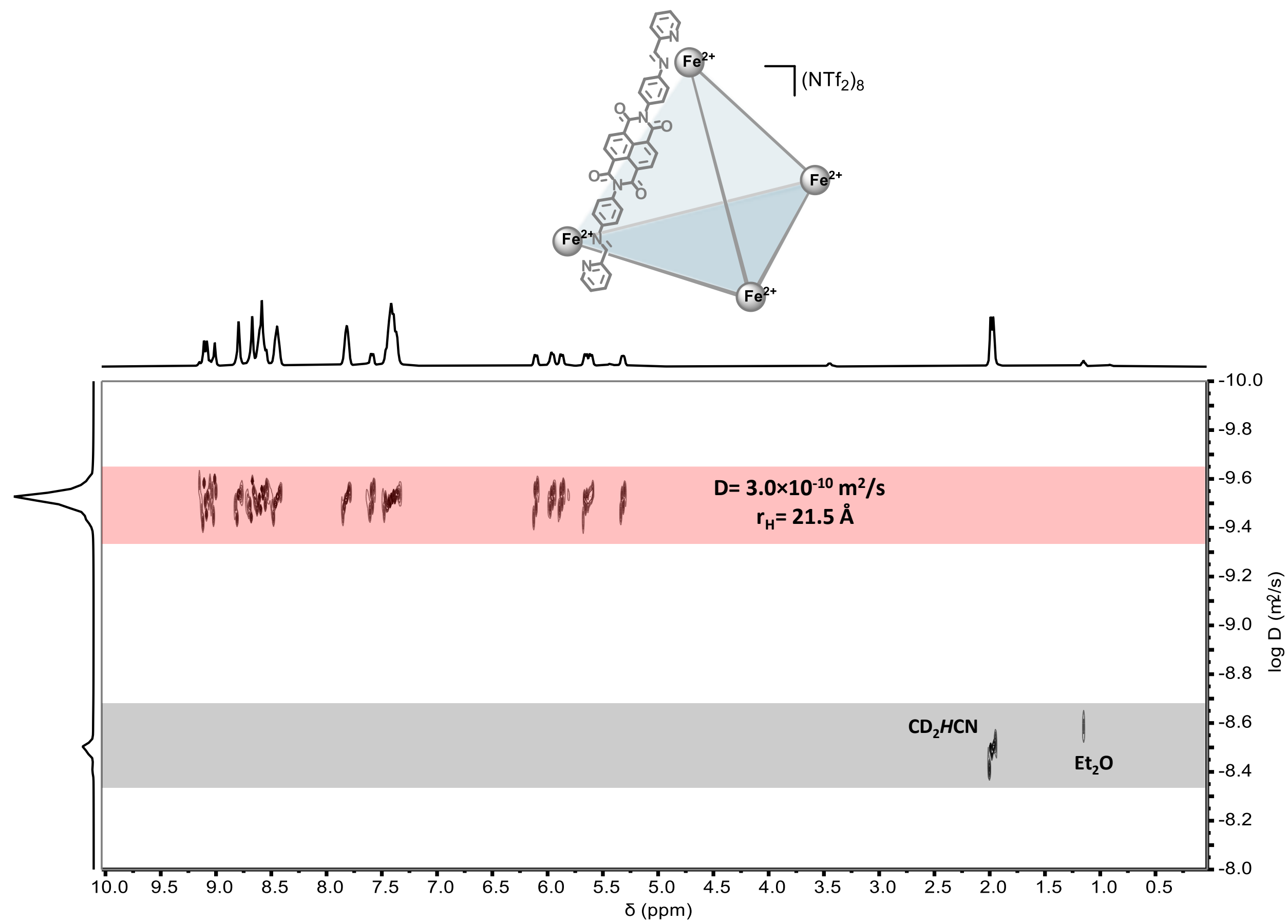

Figure S16. ^{1}H DOSY NMR spectrum (400 MHz, CD_3CN) of **[Fe_4L_6]**$(NTf_2)_8$, showing a diffusion coefficient of 3.0×10^{-10} m^2/s (r_H = 21.5 Å).

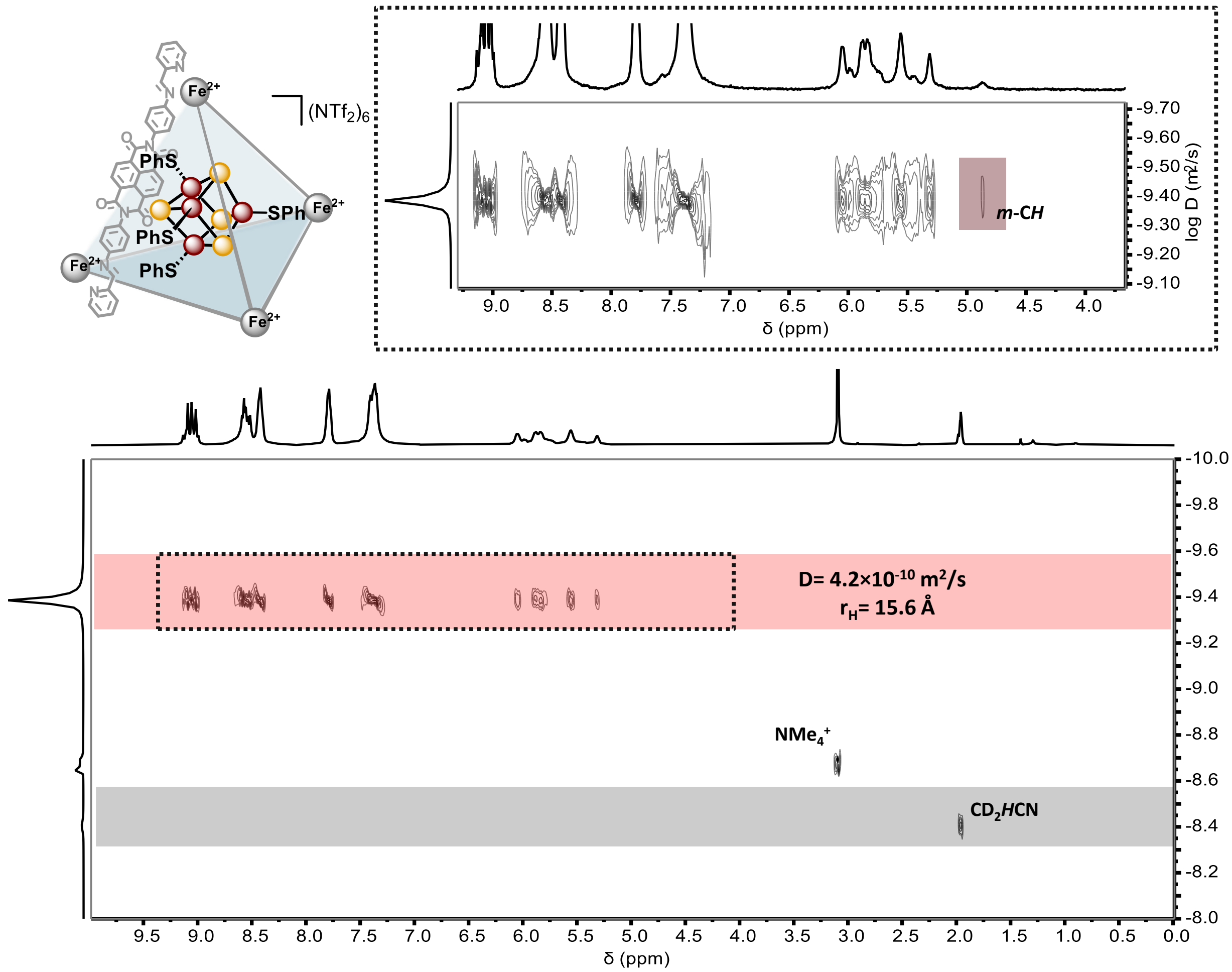

Figure S17. ^{1}H DOSY NMR spectrum (400 MHz, CD_3CN) of $\mathbf{1^{Ph}\subset[Fe_4L_6]}(NTf_2)_6$, showing a diffusion coefficient of 4.2×10^{-10} m^2/s (r_H = 15.6 Å).

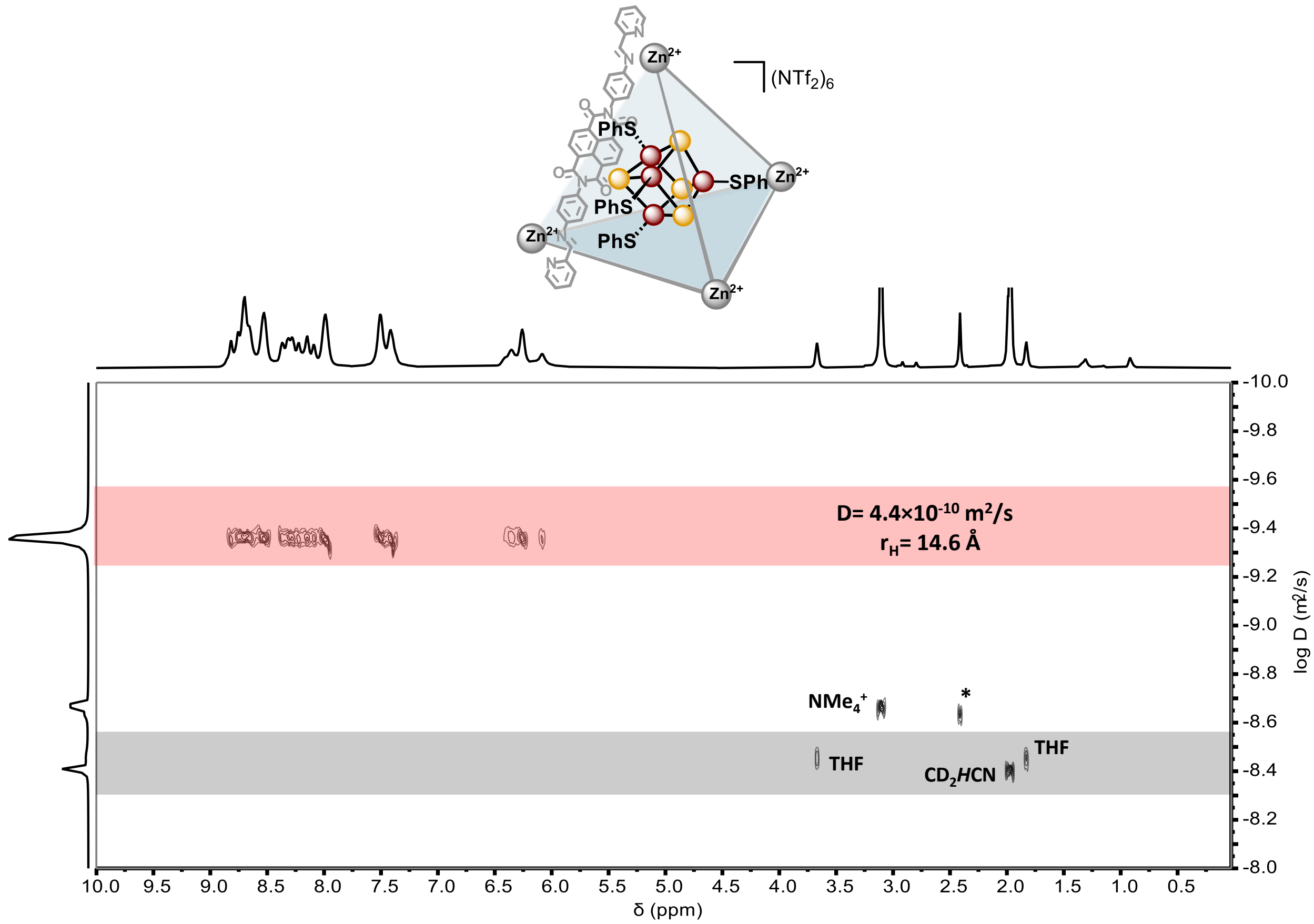

Figure S18. ^{1}H DOSY NMR spectrum (400 MHz, CD_3CN) of $\mathbf{1^{Ph}\subset[Zn_4L_6]}(NTf_2)_6$, showing a diffusion coefficient of 4.4×10^{-10} m^2/s (r_H = 14.6 Å) (*unidentified impurities).

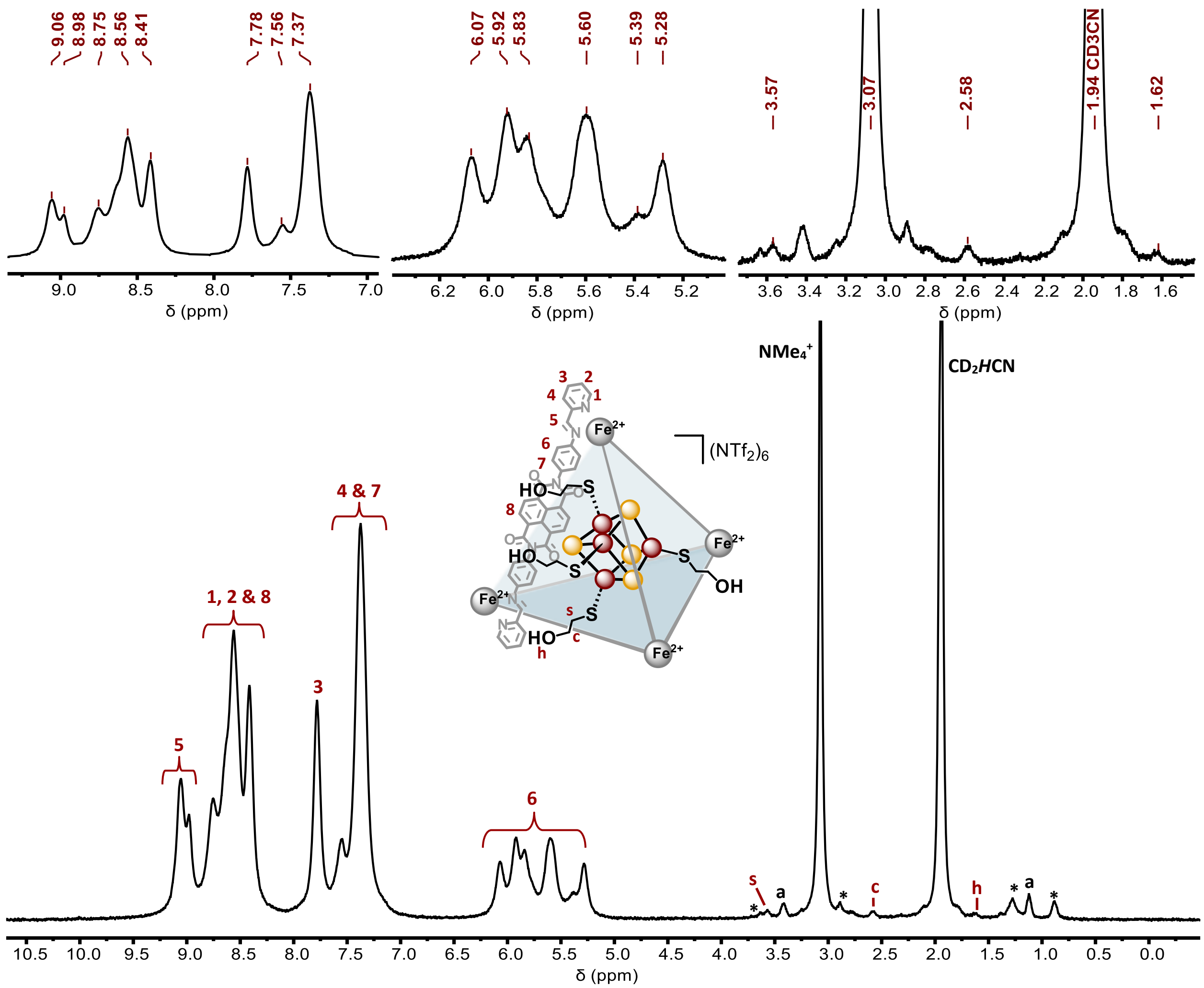

Figure S19. ^{1}H NMR spectrum (400 MHz, CD_3CN) of $\mathbf{1^{OH}{\subset}[Fe_4L_6]}(NTf_2)_6$ ([a] residual Et_2O; *unidentified impurities).

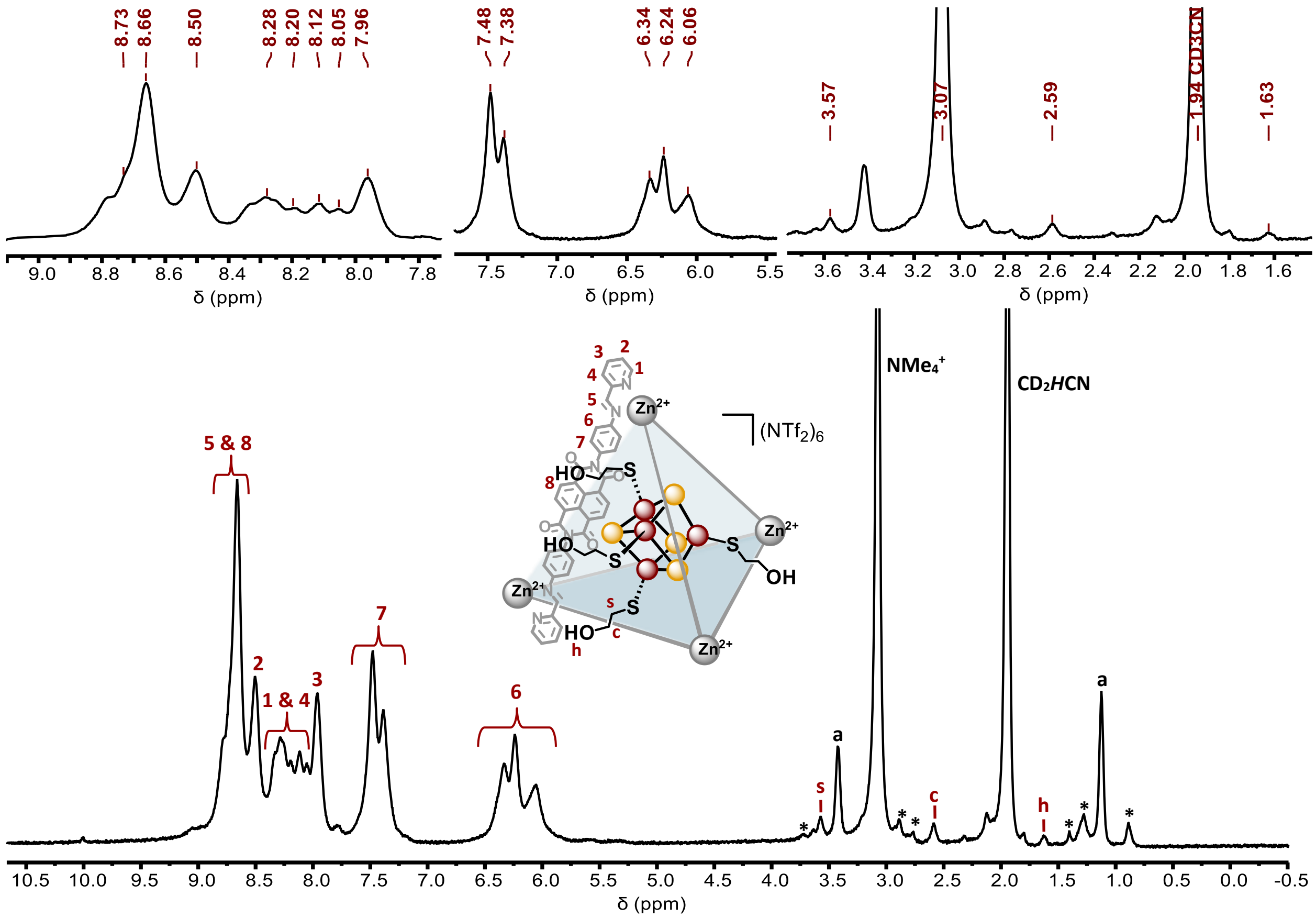

Figure S20. ^{1}H NMR spectrum (400 MHz, CD_3CN) of $\mathbf{1^{OH}{\subset}[Zn_4L_6]}(NTf_2)_6$ (^{a} residual Et_2O; *unidentified impurities).

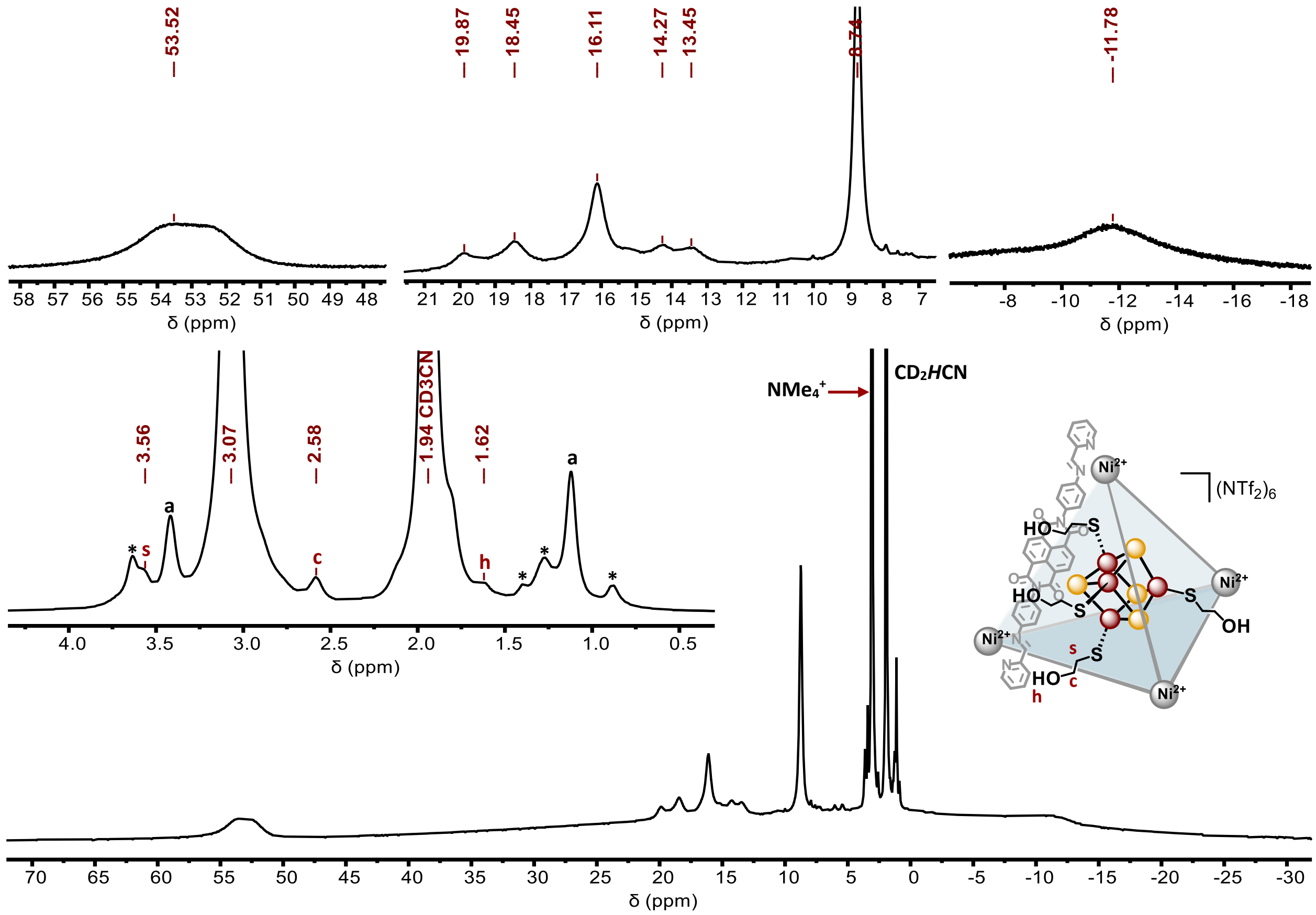

Figure S21. ^{1}H NMR spectrum (400 MHz, CD_3CN) of $\mathbf{1^{OH}\subset[Ni_4L_6]}(NTf_2)_6$ (^{a} residual Et_2O; *unidentified impurities).

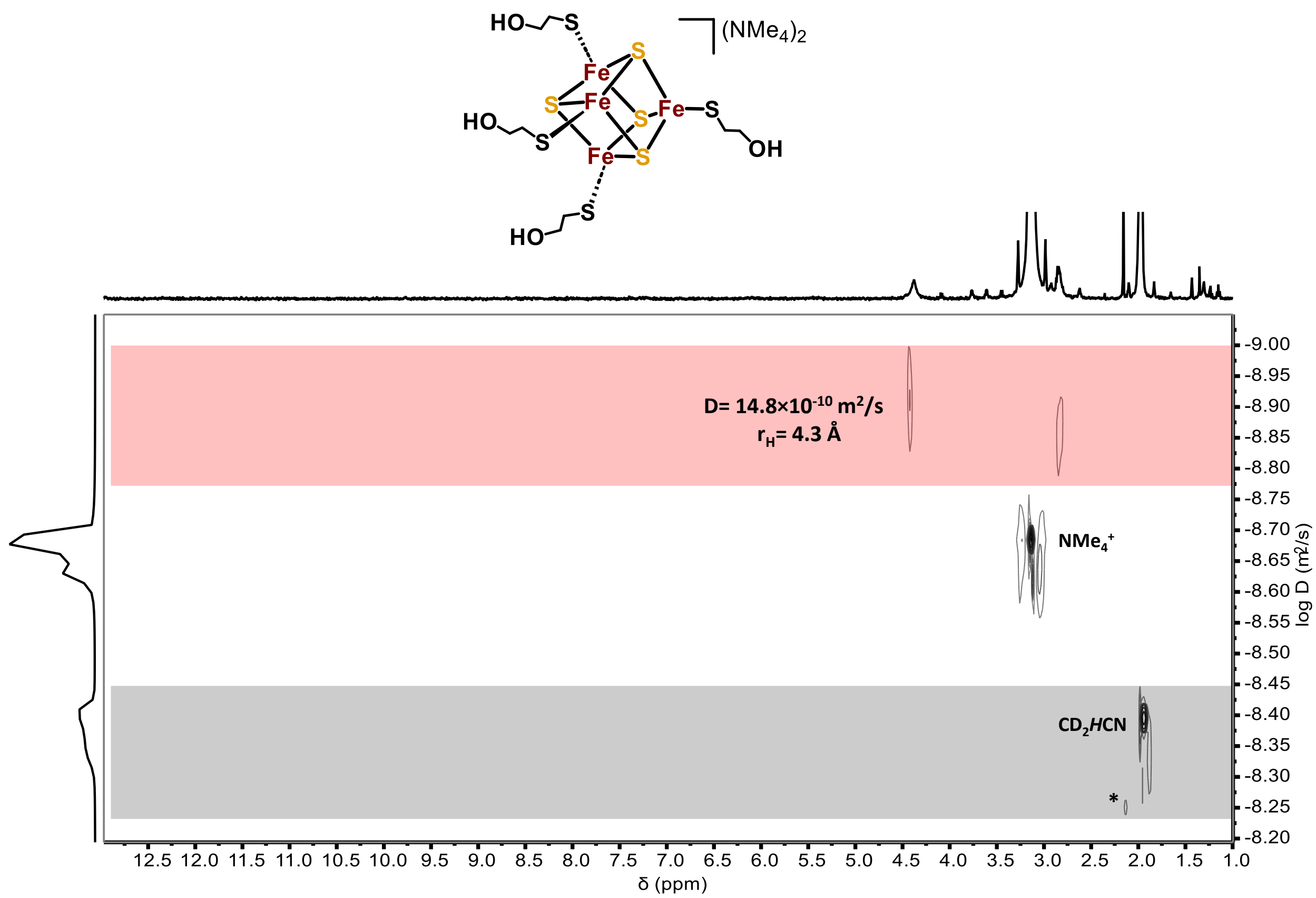

Figure S22. ^{1}H DOSY NMR spectrum (400 MHz, CD_3CN) of $(NMe_4)_2$**[1^{OH}]**, showing a diffusion coefficient of 14.8×10^{-10} m^2/s (r_H = 4.3 Å) (*unidentified impurities).

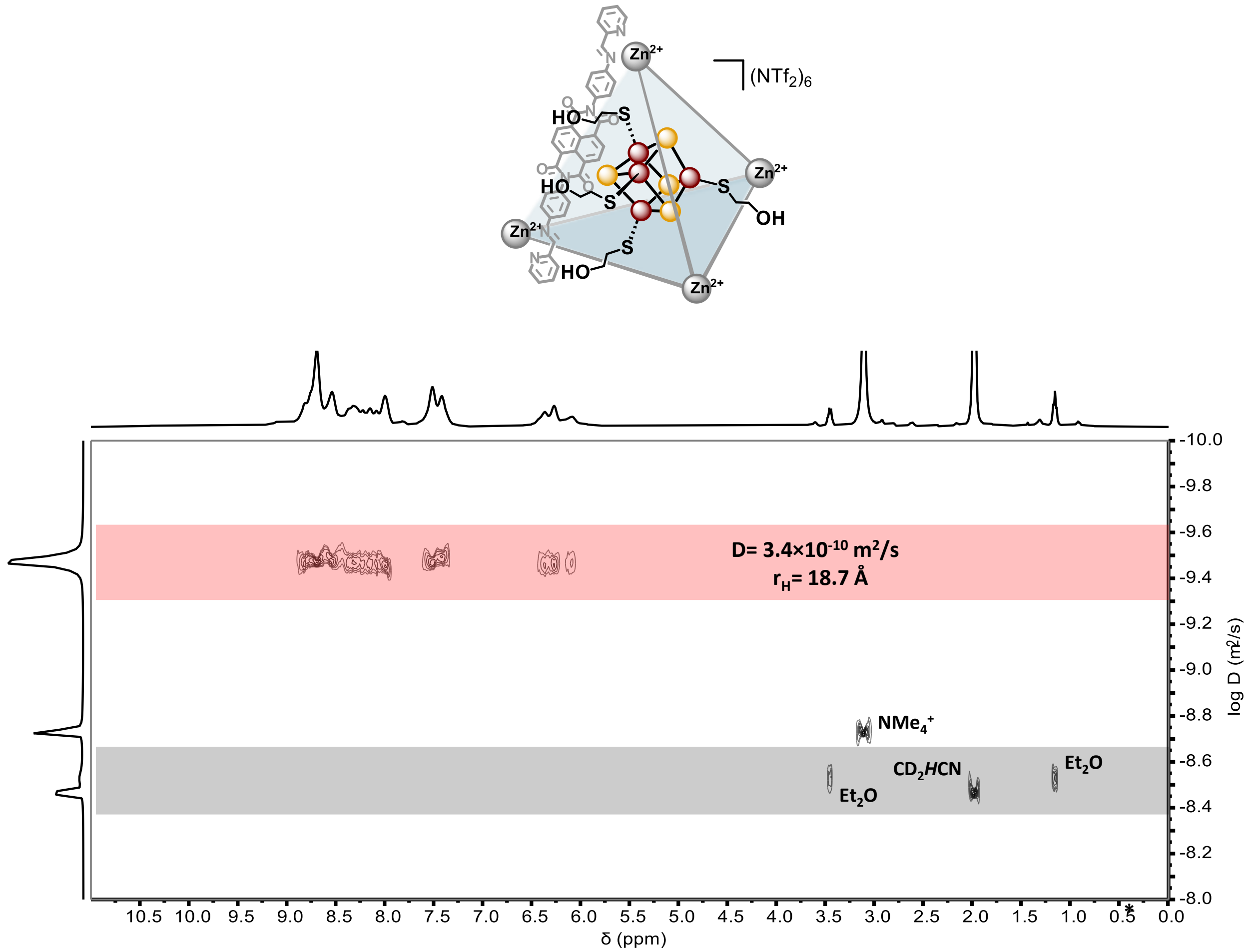

Figure S23. ^{1}H DOSY NMR spectrum (400 MHz, CD_3CN) of **1^{OH}⊂[Zn_4L_6]**(NTf_2)$_6$, showing a diffusion coefficient of 3.5×10^{-10} m^2/s (r_H = 20 Å).

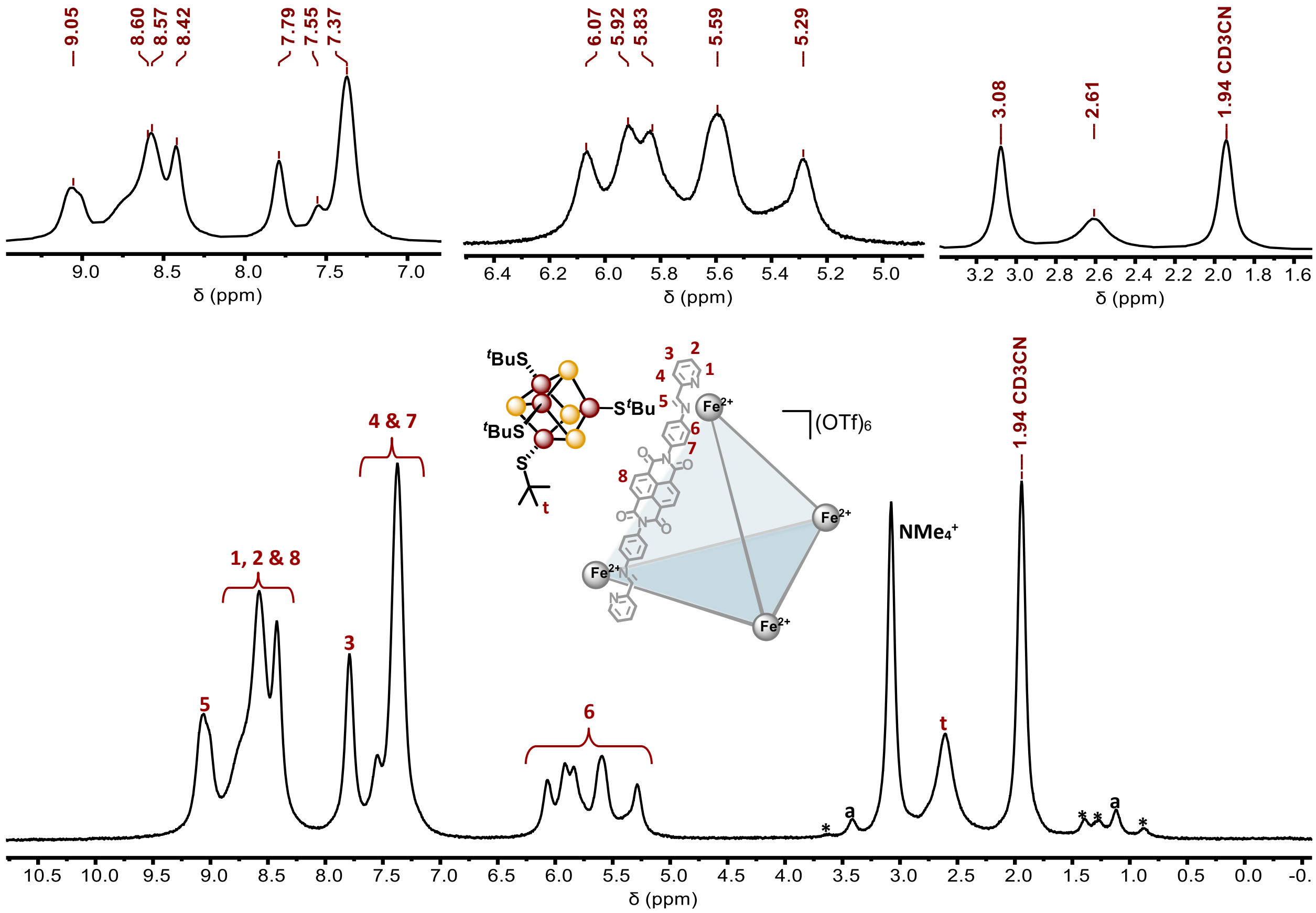

Figure S24. ^{1}H NMR spectrum (400 MHz, CD_3CN) of **[Fe$_4$L$_6$][1^{tBu}]**(OTf)$_6$ (^{a} residual Et_2O; *unidentified impurities).

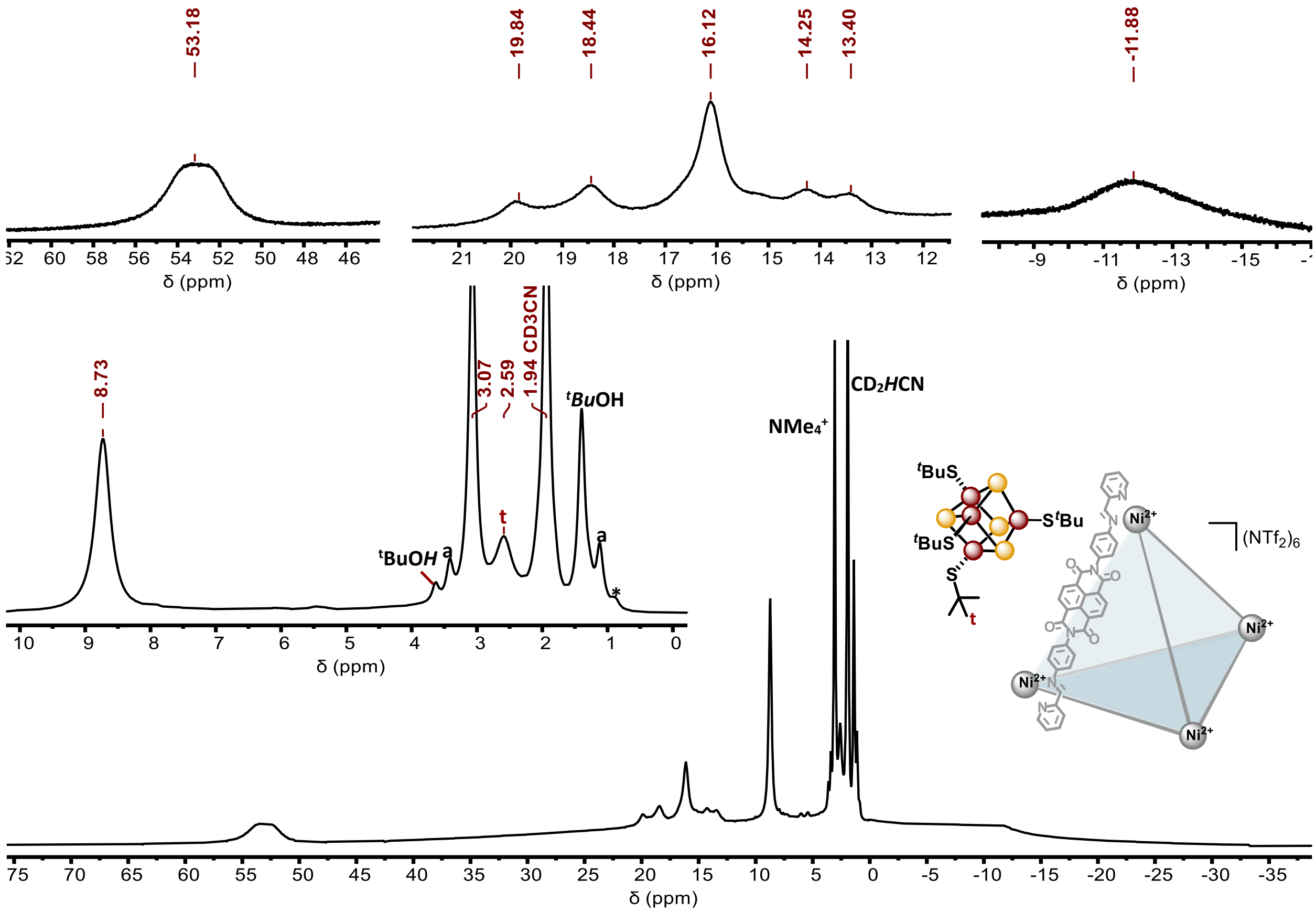

Figure S25. ^{1}H NMR spectrum (400 MHz, CD_3CN) of **$[Ni_4L_6][1^{tBu}]$**$(NTf_2)_6$ ([a] residual Et_2O; *unidentified impurities).

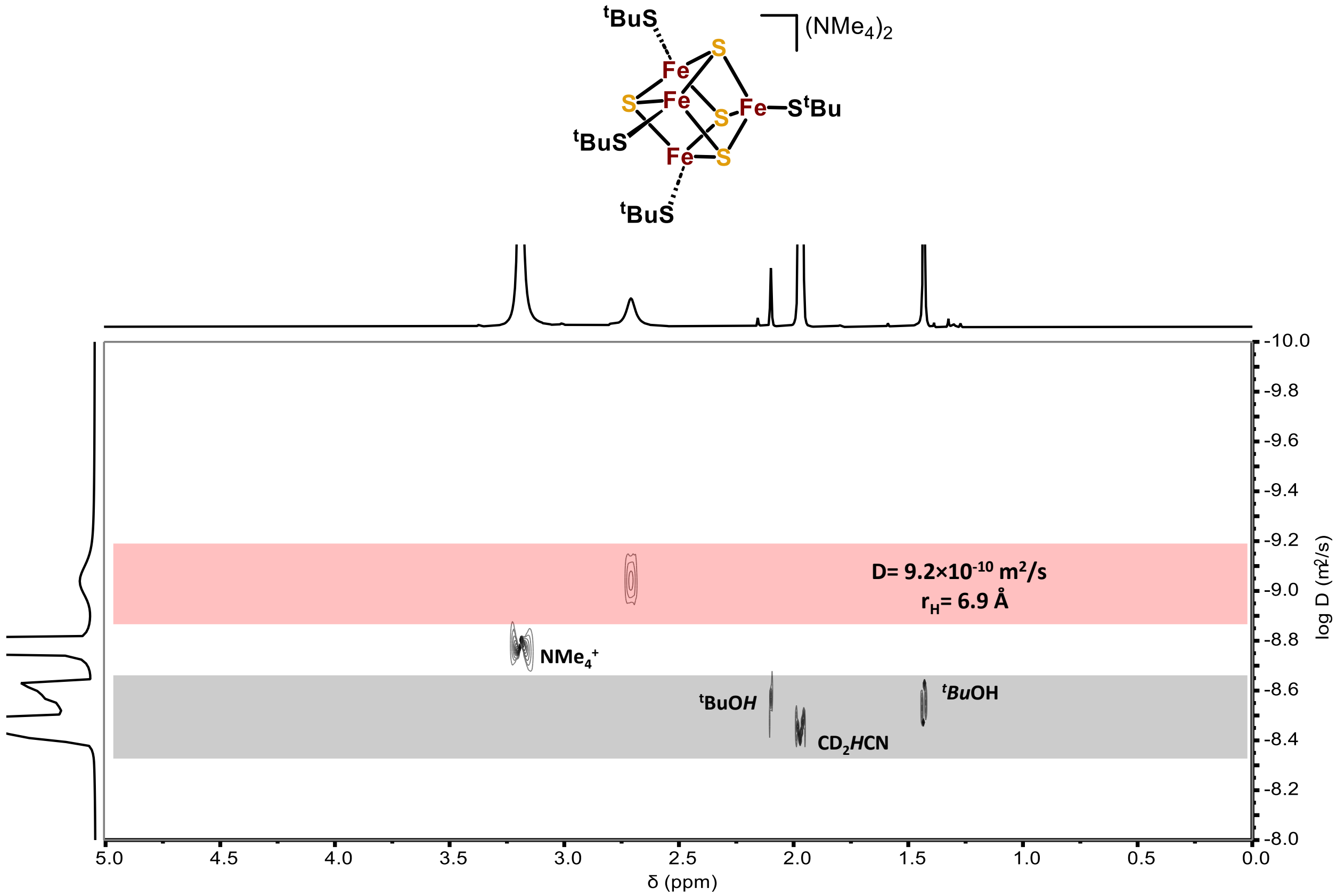

Figure S26. ^{1}H DOSY NMR spectrum (400 MHz, CD_3CN) of $(NMe_4)_2$**[1^{tBu}]**, showing a diffusion coefficient of 9.2×10^{-10} m^2/s (r_H = 6.9 Å).

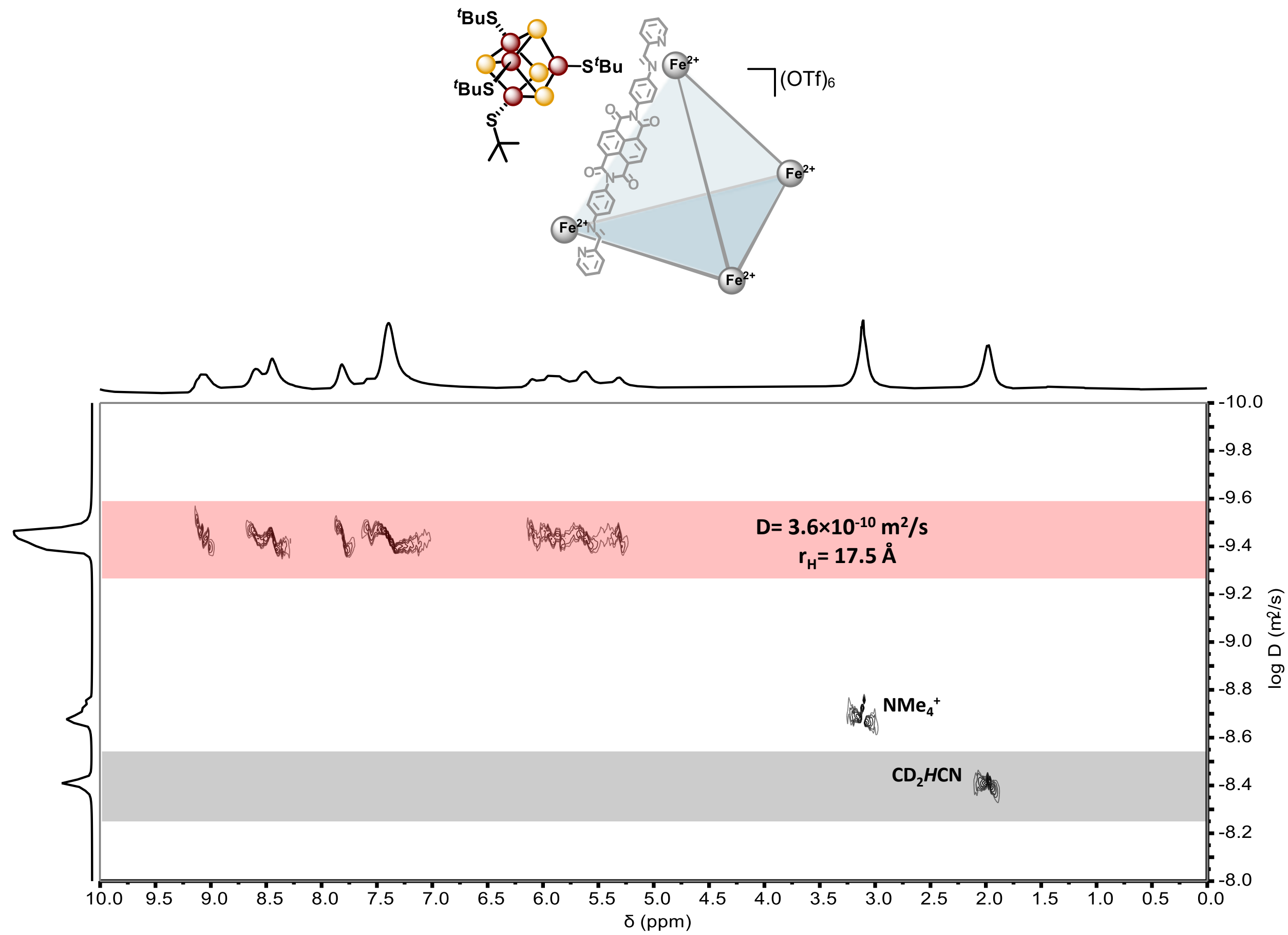

Figure S27. ^{1}H DOSY NMR (400 MHz, CD$_3$CN) of **[Fe$_4$L$_6$][1^{tBu}]**(OTf)$_6$, showing a diffusion coefficient of 3.6×10^{-10} m^2/s (r_H = 17.5 Å).

S5. MASS SPECTROMETRY

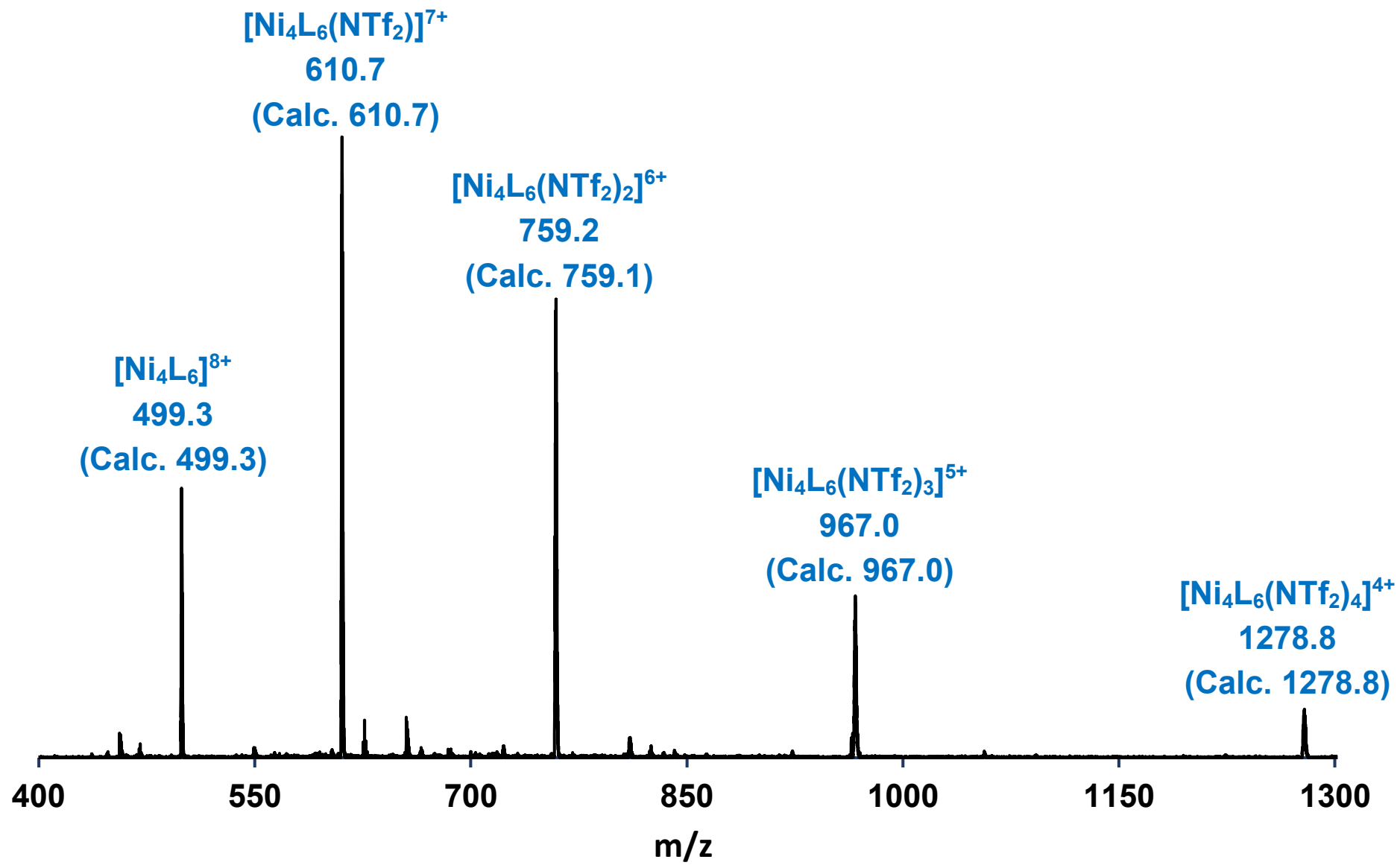

Figure S28. Low-resolution ESI mass spectrum of **[Ni_4L_6]**(NTf_2)$_8$.

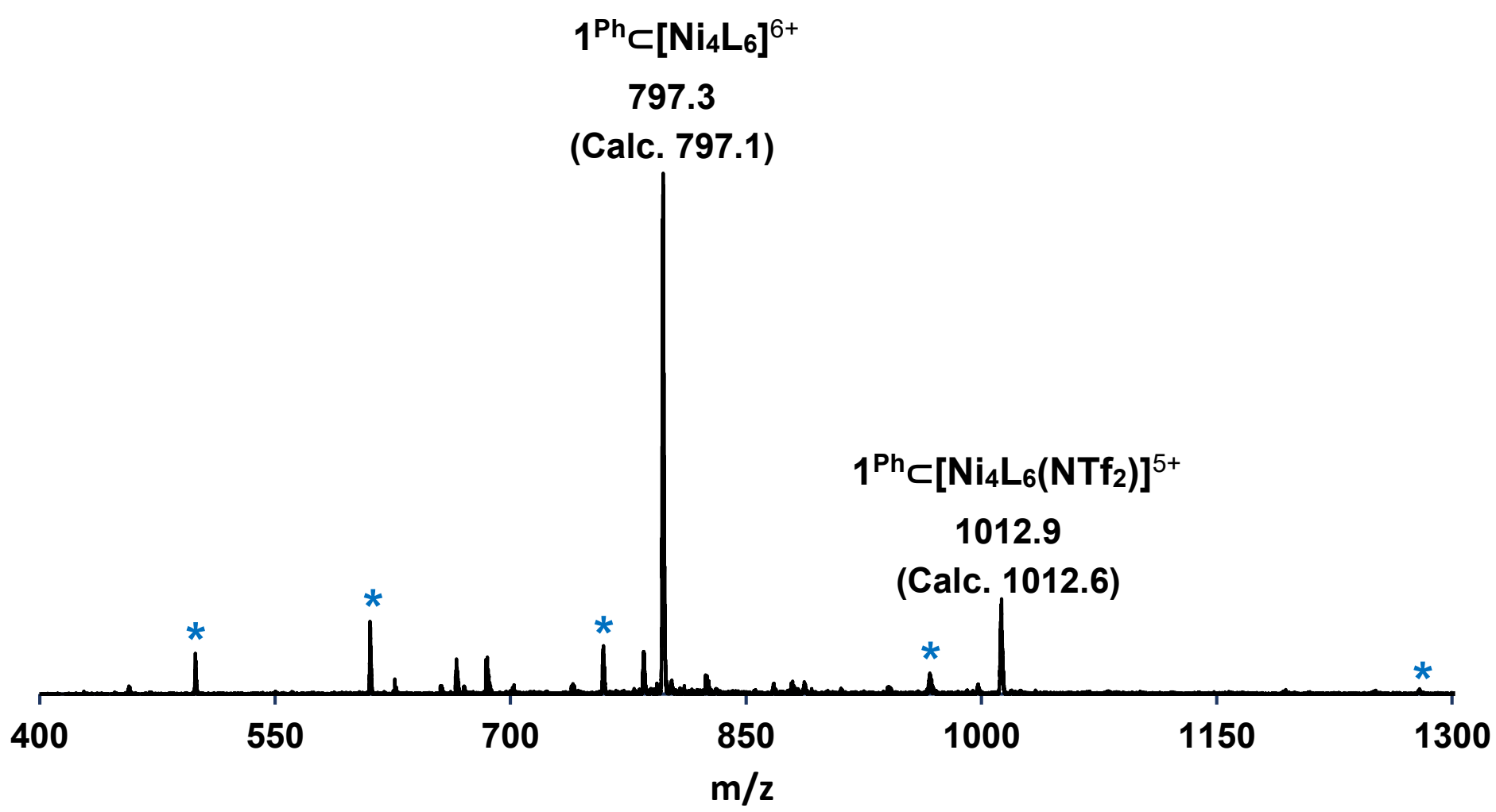

Figure S29. Low-resolution ESI mass spectrum of **1^{Ph}⊂[Ni_4L_6]**(NTf_2)$_6$. *represents the peaks from the empty cage.

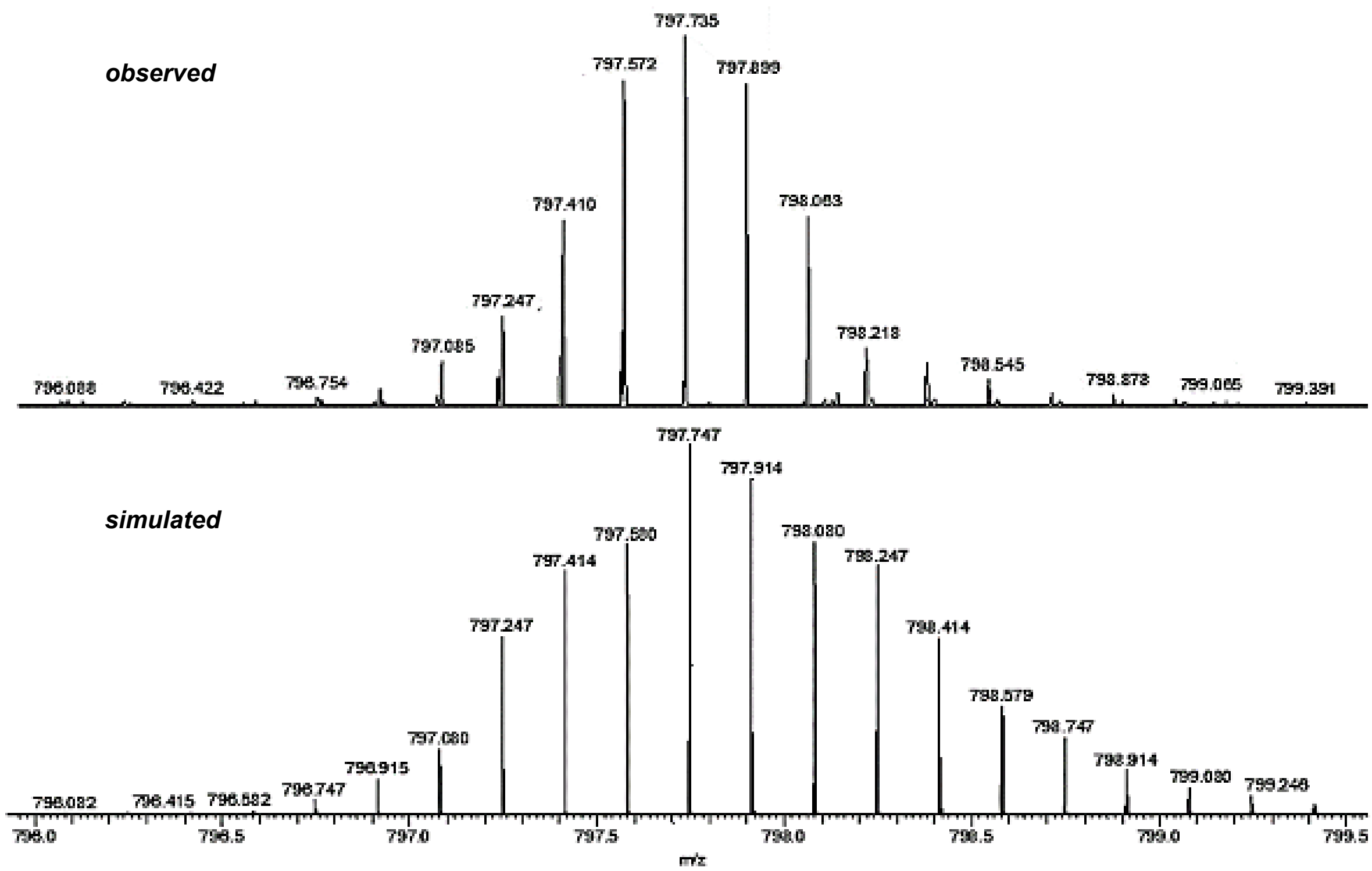

Figure S30. High-resolution (top) and simulated (bottom) ESI mass spectra of m/z 797.7 peaks assigned to $\mathbf{1^{Ph}{\subset}[Ni_4L_6]^{6+}}$ in $\mathbf{1^{Ph}{\subset}[Ni_4L_6]}(NTf_2)_8$.

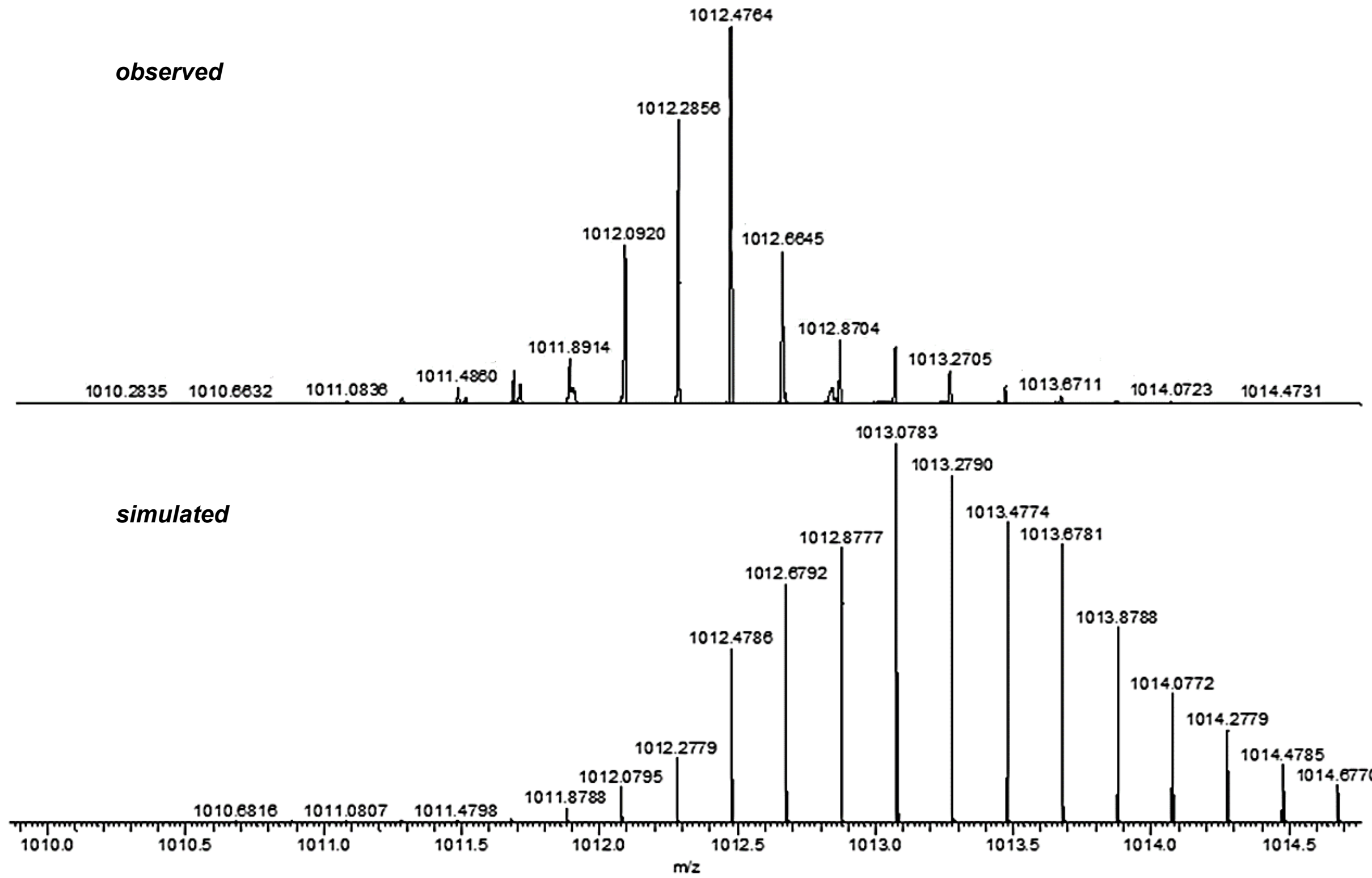

Figure S31. High-resolution ESI mass spectrum of in $\mathbf{1^{Ph}{\subset}[Ni_4L_6]}(NTf_2)_6$ showing magnified *m/z* 1012.5 pattern (top), with simulated pattern for $\mathbf{1^{Ph}{\subset}[Ni_4L_6(NTf_2)]^{5+}}$ at m/z 1013.08 (bottom) showing a structural mismatch. This contrasts with the low-resolution ESI-MS data (Figure S29), which shows a clear peak m/z 1012.9 matching the expected value for $\mathbf{1^{Ph}{\subset}[Ni_4L_6(NTf_2)]^{5+}}$.

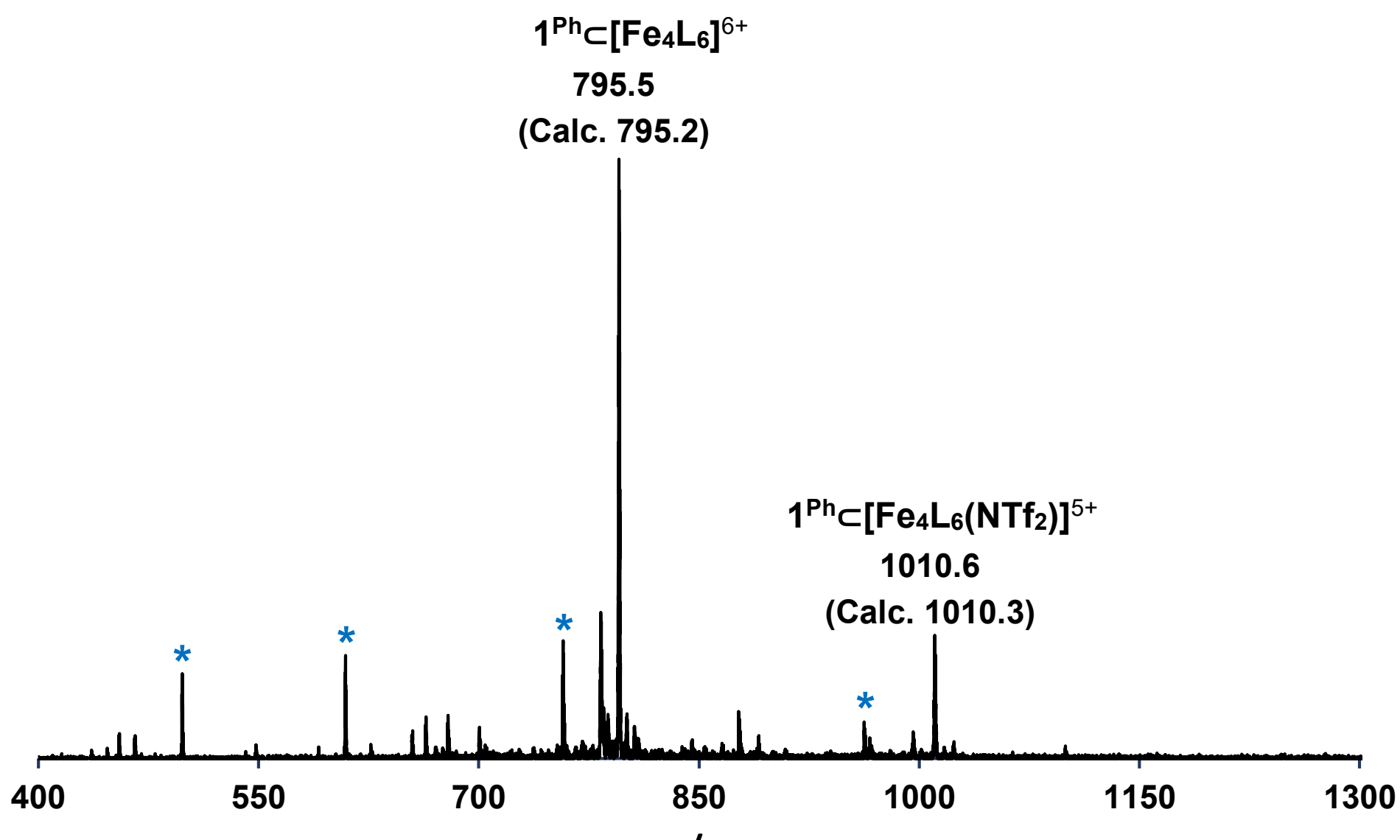

Figure S32. Low-resolution ESI mass spectrum of $\mathbf{1^{Ph}{\subset}[Fe_4L_6]}(NTf_2)_6$. *represents the peaks from the empty cage.

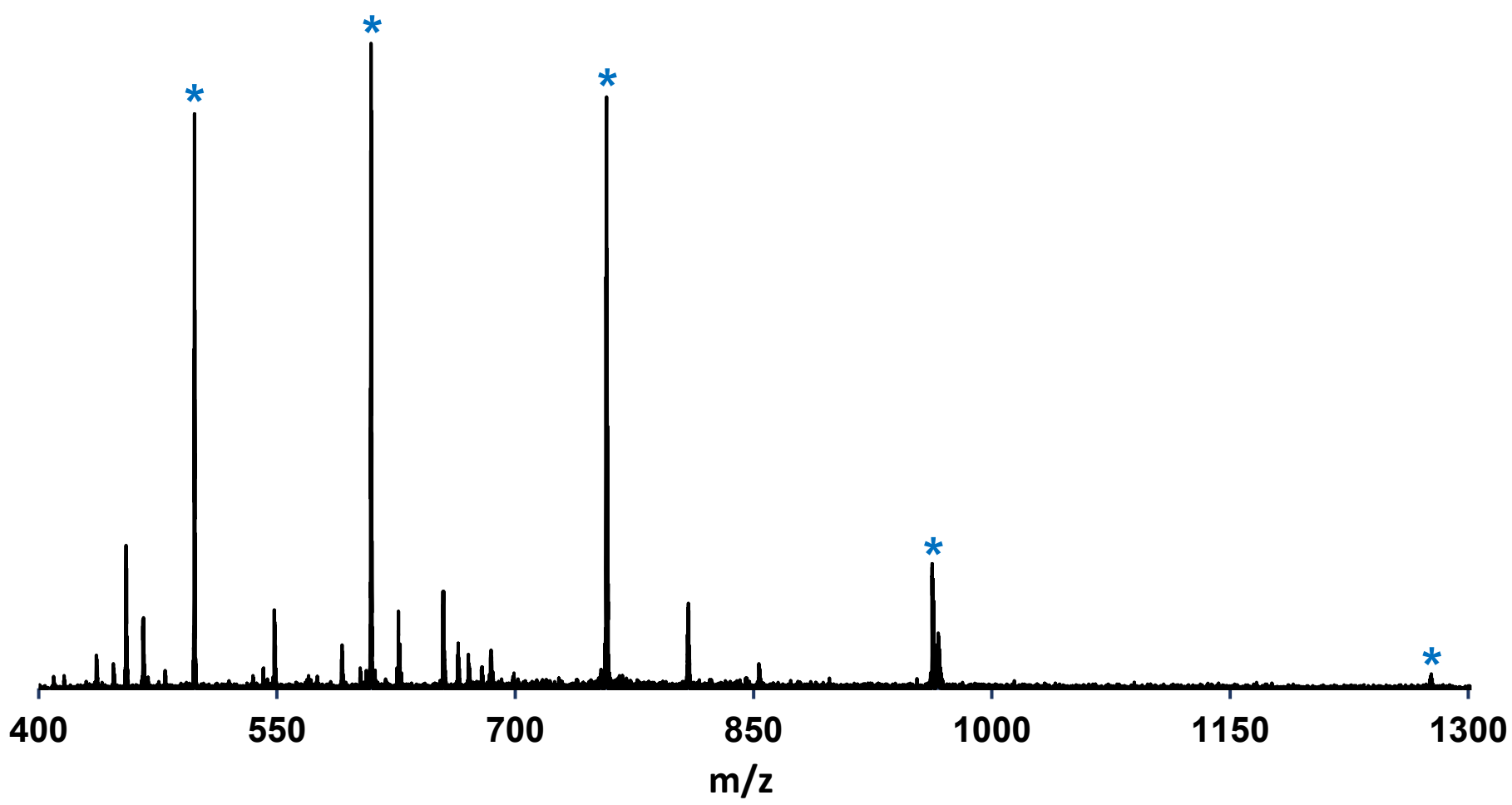

Figure S33. Low-resolution ESI mass spectrum of $\mathbf{1^{OH}\subset[Fe_4L_6]}(NTf_2)_6$. *indicates peaks assigned to the empty cage. We were unable to detect signals for the host-guest complex, suggesting lability under the conditions of ESI-MS due to its small size (packing coefficient = 0.36) and weak binding interactions.

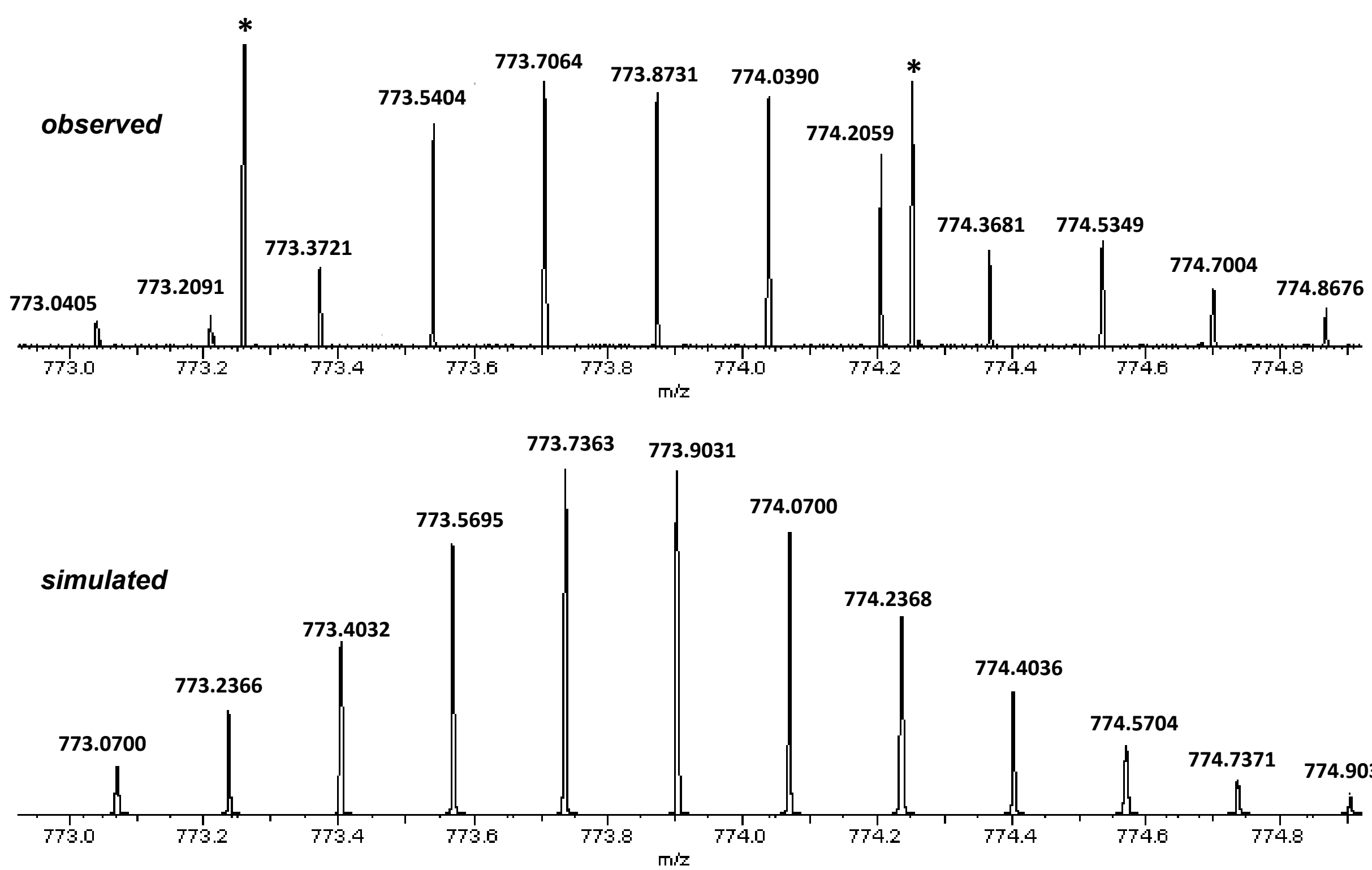

Figure S34. High-resolution (top) and simulated (bottom) ESI mass spectra of m/z 773.9 peaks assigned to $\mathbf{1^{OH}\subset[Fe_4L_6]}^{6+}$ in $\mathbf{1^{OH}\subset[Fe_4L_6]}(NTf_2)_6$. *represents unidentified peaks.

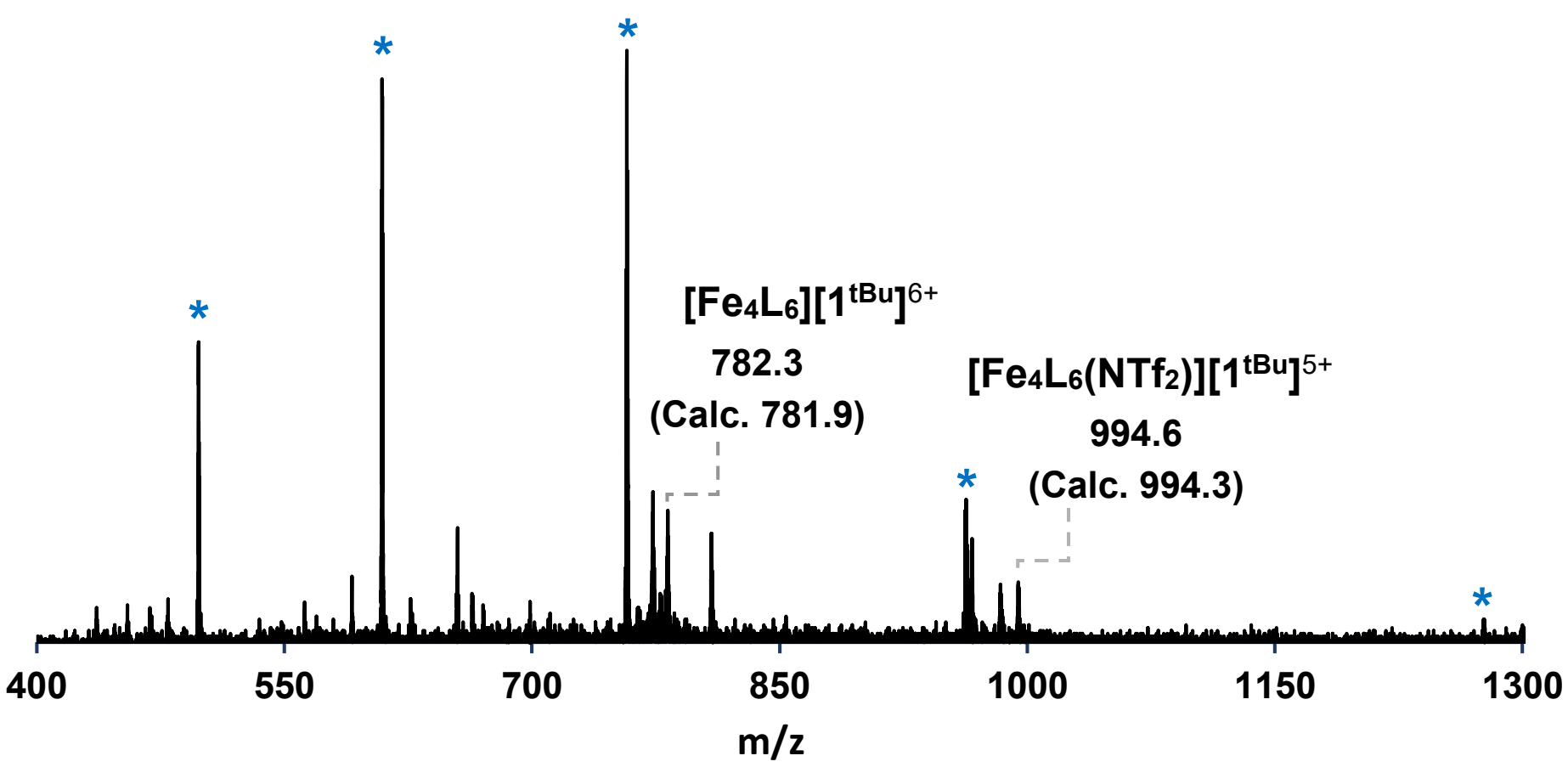

Figure S35. Low-resolution ESI mass spectrum of **[Fe_4L_6][1^{tBu}]**(NTf_2)$_6$. *represents the peaks from the empty cage.

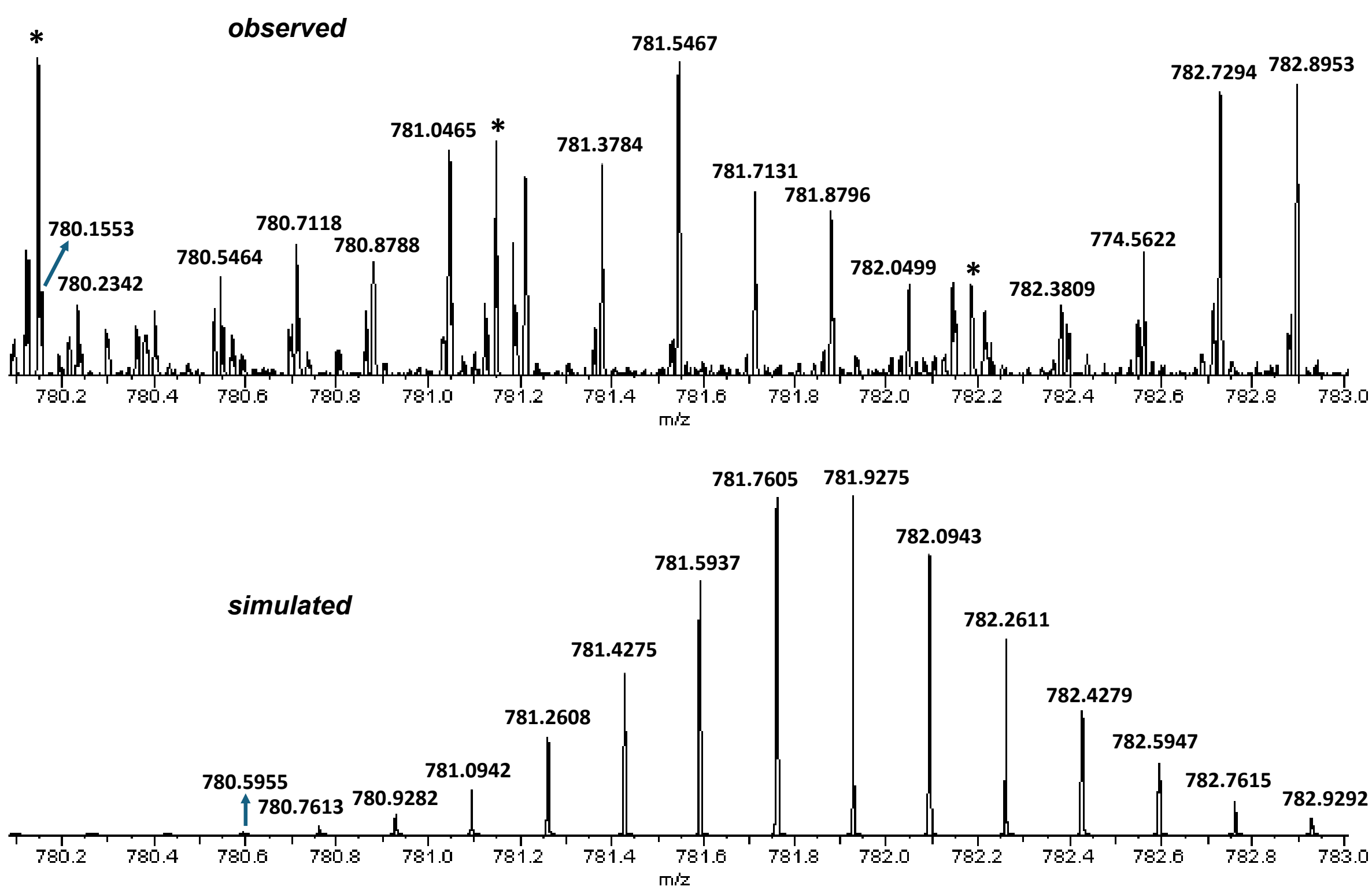

Figure S36. High-resolution ESI mass spectrum of m/z 781.5 peak (top), with simulated pattern for **[Fe_4L_6][1^{tBu}]$^{6+}$** in **[Fe_4L_6][1^{tBu}]**(NTf_2)$_6$ at m/z 781.9 (bottom) showing that while the masses are similar, the isotope patterns and precise mass clearly confirm a different structural assignment.

S6. CYCLIC VOLTAMMETRY

Electrochemical experiments were performed in an N_2-filled glovebox using a WaveNowXV potentiostat. Data processing was performed using the Aftermath software package (Pine Research). Experiments were performed using a three-electrode setup consisting of a 3 mm diameter glassy carbon disk working electrode (CH Instruments), a platinum wire counter electrode (CH Instruments), and a low-profile nonaqueous silver wire reference electrode housed in a capillary (Pine Research). The glassy carbon disk working electrode was polished with an alumina and water slurry (0.05 µm particle size, BASi Research Products), rinsed with methanol, and dried prior to use. The silver reference electrode was lightly polished using 600 grit sandpaper, rinsed with methanol, and dried before inserting into a capillary containing a solution of 100 mM $[N^nBu_4][PF_6]$ electrolyte and 10 mM $AgNO_3$ in acetonitrile. All working electrode potentials were measured versus the $Ag/AgNO_3$ redox couple and calibrated to the $Fc^{0/+}$ redox couple using an internal ferrocene reference. Analyte solutions were prepared from dry, degassed solvent containing 100 mM NMe_4PF_6 or $[N^nBu_4][PF_6]$ as the supporting electrolyte.

The Randles–Ševčík equation (Equation S2) was used to assess the electrochemical reversibility and determine the number of electrons (n) associated with redox events for the complexes **[M₄L₆]**$(NTf_2)_8$ (M= Fe, Zn, Ni) and $\mathbf{1^{Ph}}$**⊂[M₄L₆]**$(NTf_2)_6$ (M= Fe, Zn) according to equation S1:

$$i_p = 0.4463\, nFAC \left(\frac{nFvD}{RT}\right)^{1/2} \quad \textbf{(Equation S2)}$$

In this equation, i_p is the peak current measured at a given scan rate v; A is the surface area of the working electrode (0.071 cm^2); C is the analyte concentration (0.1 mM); D is the analyte diffusion coefficient at temperature T (298.15 K); F is Faraday's constant (96485 C mol^{-1}); and R is the ideal gas constant (8.3145 J mol^{-1} K^{-1}). The diffusion coefficients were determined by ^1H DOSY NMR analysis taken under identical conditions (CD_3CN at 25 °C), which is an approximation for the second and subsequent redox events since D can change substantially as a function of redox state.

Determination of *n*: the measured peak currents for the isolated events (i_{pc} and i_{pa} for the cathodic and anodic peak currents, respectively) were plotted as a function of the square root of the scan rate, $\sqrt{v}$, and Equation S2 was used to solve for n using the fitted slopes. The results for these measurements are summarized in **Table S1** along with the redox potentials and assignments for each event measured.

Determination of (electro)chemical reversibility: These studies focused on the first reduction event E_{red1}, as the analysis of subsequent events was complicated by overlapping signals and potential guest/host precipitation at highly negative potentials. To assess reversibility, we evaluated the ratio of the slopes from the Randles–Ševčík plots (i_{pc}/i_{pa}) and the peak-to-peak separation (ΔE_p).

For the first reduction peak (E_{red1}) at ~ –1.0 V, the ratio of the slopes was calculated to be 1.06 and 1.05 for **[Fe₄L₆]**$(NTf_2)_8$ and $\mathbf{1^{Ph}}$**⊂[Fe₄L₆]**$(NTf_2)_6$, respectively. These values, being near unity, indicate a high degree of chemical reversibility. However, the peak-to-peak separation (ΔE_p) increases from 60 and 100 mV at a scan rate of 0.1 V/s to 130 and 145 mV at 3 V/s for **[Fe₄L₆]**$(NTf_2)_8$ and $\mathbf{1^{Ph}}$**⊂[Fe₄L₆]**$(NTf_2)_6$, respectively. This trend is consistent with electrochemical quasireversibility, suggesting that the rate of heterogeneous electron transfer is slow relative to the voltage sweep rate at higher scan rates.

Table S1. Summary of reduction potentials, calculated number of electrons (n), and assignments for redox events in the cyclic voltammograms of $\mathbf{[M_4L_6]^{8+}}$ (M= Fe, Zn, Ni) and $\mathbf{1^{Ph}}$$\mathbf{\subset[M_4L_6]^{6+}}$ (M = Fe, Zn).[a]

		E_{red2} (n^b)		E_{red3}		
	E_{red1} (n)	E_{red2a}	E_{red2b}	E_{red3a}	E_{red3b}	E_{ox1}
**[Zn₄L₆]**$(NTf_2)_8$	–0.97 (5.9 e⁻)	–1.53 (11.3 e⁻)		–2.30		

**[Fe_4L_6]**(NTf_2)$_8$	–0.97 (5.6 e⁻)	–1.36	–1.59	–1.73	–2.28	
**[Ni_4L_6]**(NTf_2)$_8$	–0.96 (5.5 e⁻)	–1.37	–1.48		–2.34	
**1^{Ph}⊂[Zn_4L_6]**(NTf_2)$_6$	–1.03 (5.5 e⁻)	–1.57 (9.4 e⁻)		–2.37		–0.30
**1^{Ph}⊂[Fe_4L_6]**(NTf_2)$_6$	–1.00 (6.2 e⁻)	–1.61		–1.77	–2.31	–0.22
**1^{Ph}⊂[Ni_4L_6]**(NTf_2)$_6$	–0.98	–1.50		–2.37		–0.25
**Assignment**	6 x $[NDI]^{0/–}$	6 x $[NDI]^{0/–}$	imino-pyridine[b]	imino-pyridine[b]	imino-pyridine[b]	**$[1^{Ph}]^{2–}$**

[a]Conditions: 0.1 mM in MeCN; 0.1 M $N^nBu_4PF_6$; 1 V/s; applied potentials referenced to $Fc^{0/+}$.[b][M(imino-pyridine)]$^{n+}$ nodes. [b]It is not possible to confidently determine the electrons transferred in E_{red2} (n) are approximate due to the uncertainty associated with the diffusion coefficient D (Equation S2) combined with possible issues of precipitation. As such, the reported values are an approximation only. The first oxidation feature (E_{ox1}) of **$[1^{Ph}]^{2-}$** (Figure S38, S41 and S54) is shifted ~ 0.3V cathodically in **1^{Ph}⊂[M_4L_6]**(NTf_2)$_6$. This is attributed to the hydrophobic environment within the cage, similar to other reports.[11–14]

Table S2. Summary of literature-reported $E_{1/2}$ values for $[M(bpy)_3]^{2+}$ complexes, with M= Fe, Ni and Zn.[15–18] Conditions: 3 mM in MeCN; 0.1-0.2 M $[NEt_4][ClO_4]$ electrolyte; 100 mV/s.

	**$E_{1/2}$ (V)**			
	**2+/1+**	**1+/0**	**bpy dissociation**	**0/-**
**$[Fe(bpy)_3]^{2+}$**	-1.75	-1.94		-2.18
**$[Ni(bpy)_3]^{2+}$**	-1.66 (2e⁻)		-2.15	-2.36
**$[Zn(bpy)_3]^{2+}$**	-1.77 (2e⁻)			-2.25

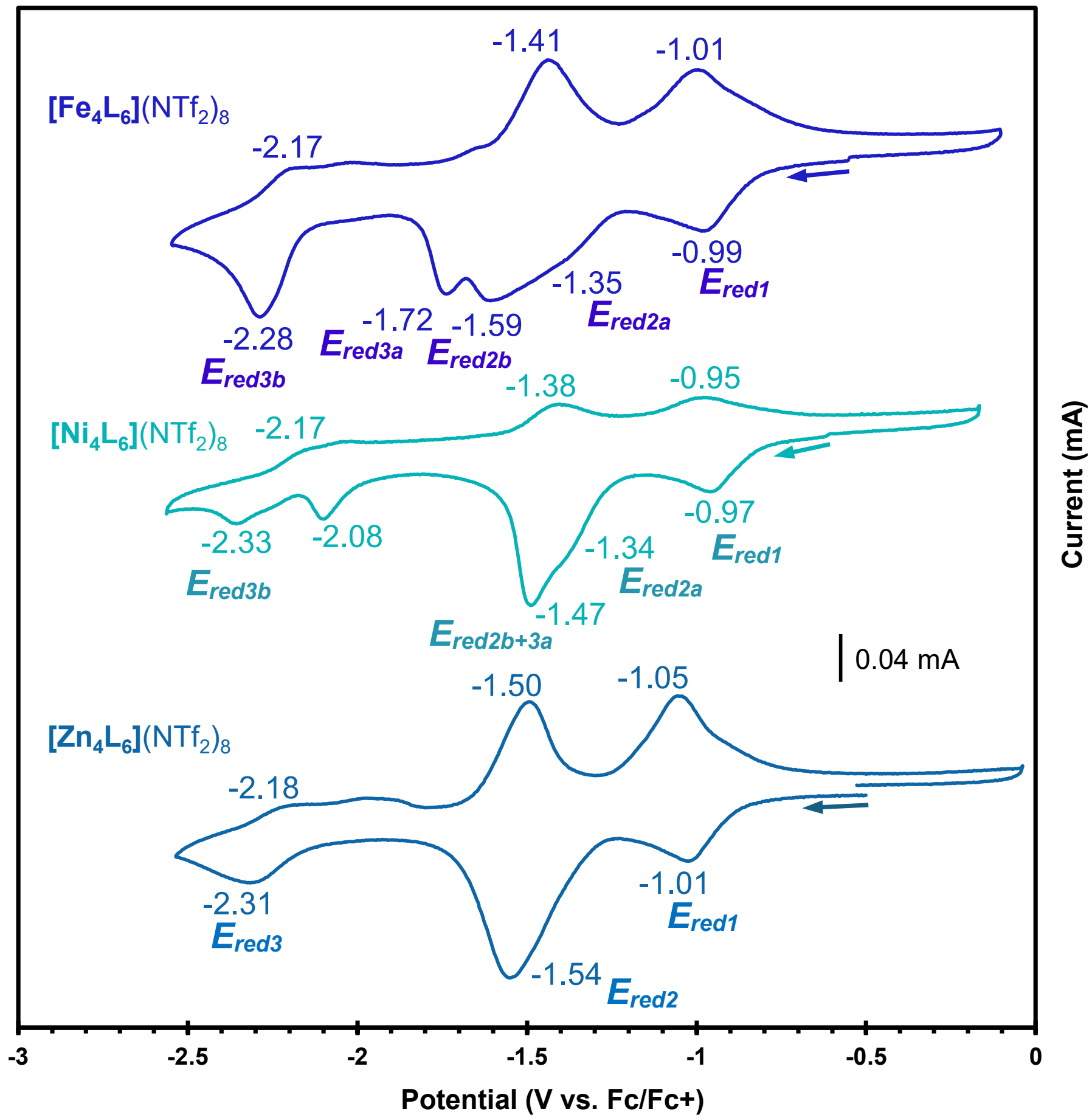

Figure 37. Cyclic voltammograms of **$[Fe_4L_6]$**$(NTf_2)_8$, **$[Ni_4L_6]$**$(NTf_2)_8$, and **$[Zn_4L_6]$**$(NTf_2)_8$. Arrows denote the scan directions. Conditions: 0.1 mM in MeCN; 0.1 M $[N^nBu_4][PF_6]$; 2 V/s. In **$[Ni_4L_6]^{8+}$**, evidence of speciation via iminopyridine dissociation was observed following the first reduction of the corner nodes of at -2.08 V indicating possible reductive lability.[18]

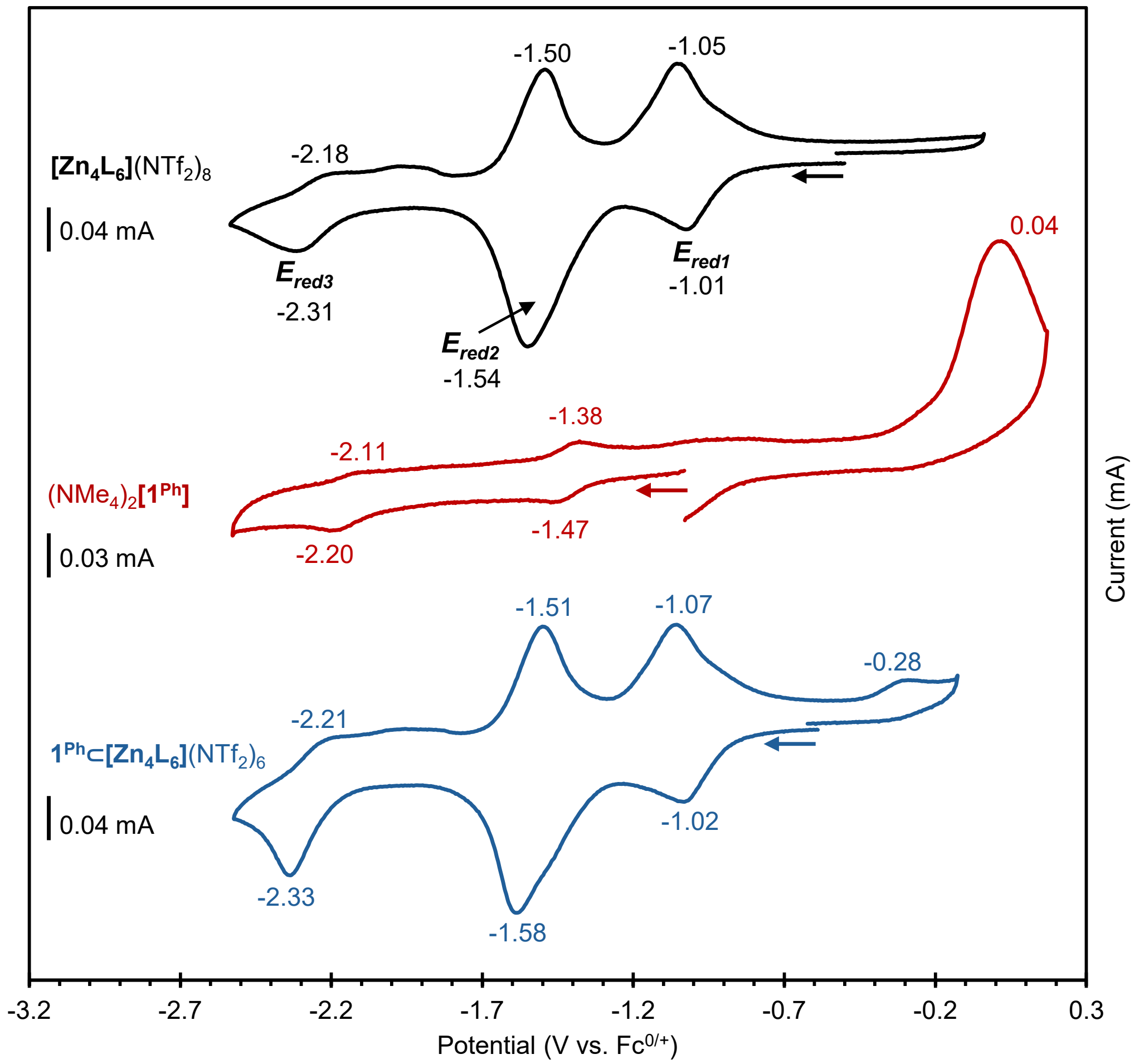

Figure S38. Cyclic voltammograms of **[Zn_4L_6]**(NTf_2)$_8$, (NMe_4)$_2$**[1^{Ph}]**, and **1^{Ph}⊂[Zn_4L_6]**(NTf_2)$_6$. Conditions: 0.1 mM in MeCN; 0.1 M $N^nBu_4PF_6$; 2 V/s. Scans were conducted starting from the measured open circuit potential of each species in the direction indicated by the arrows.

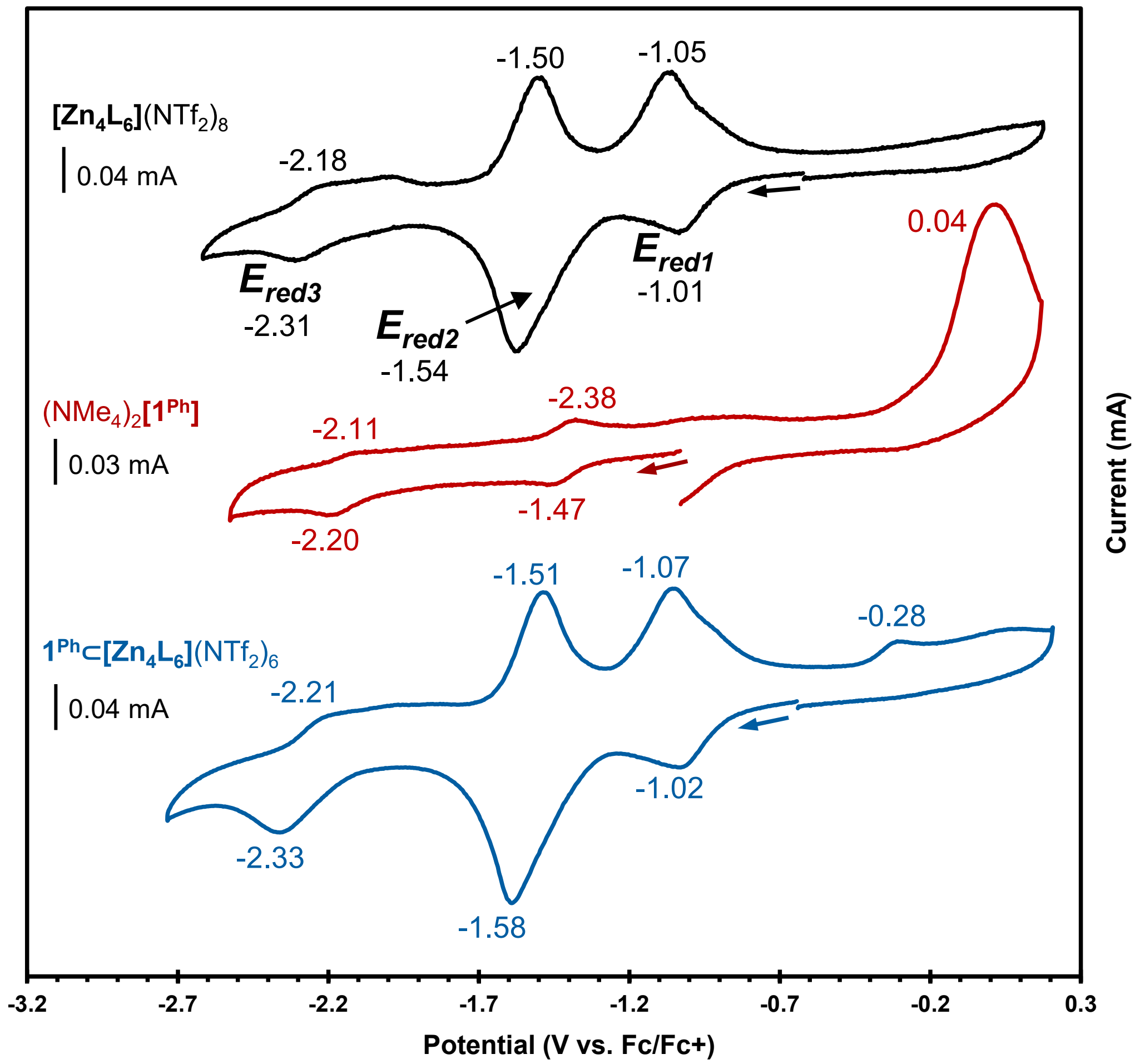

Figure S39. Cyclic voltammograms of **[Zn$_4$L$_6$]**(NTf$_2$)$_8$, (NMe$_4$)$_2$**[1^{Ph}]**, and **1^{Ph}⊂[Zn$_4$L$_6$]**(NTf$_2$)$_6$, including a wider anodic scan range for **1^{Ph}⊂[Zn$_4$L$_6$]**(NTf$_2$)$_6$. Conditions: 0.1 mM in MeCN; 0.1 M N^{n}Bu$_4$PF$_6$; 2 V/s. Scans were conducted starting from the measured open circuit potential of each species in the direction indicated by the arrows.

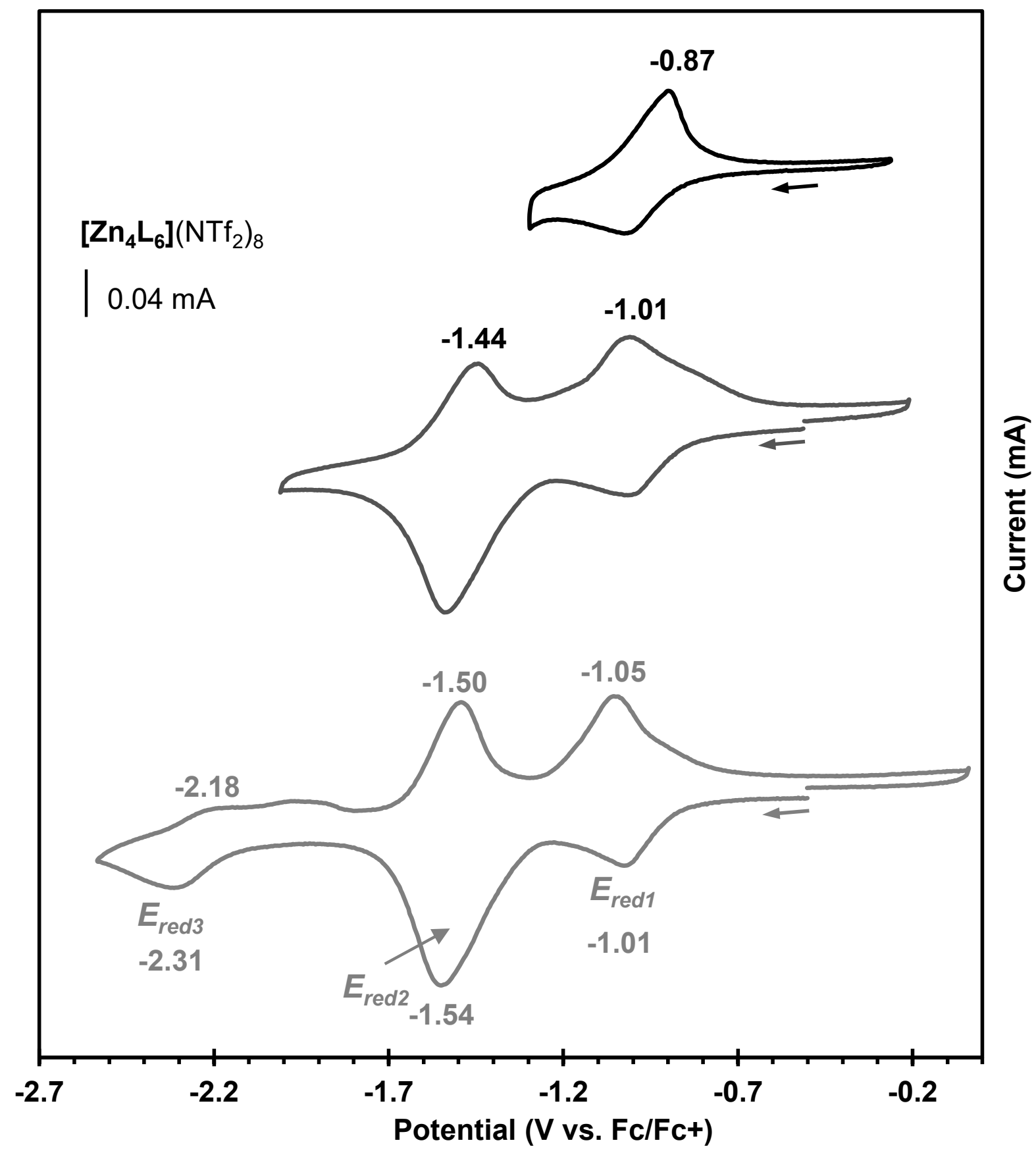

Figure S40. Cyclic voltammograms of **[Zn_4L_6]**$(NTf_2)_8$ with different windows of scan up to E_{red3}, E_{red2} and E_{red1} showing the increase in reversibility of E_{red2} and E_{red1} on moving from wider to narrower window range. Conditions: 0.1 mM in MeCN; 0.1 M $N^nBu_4PF_6$; 2 V/s. Scans were conducted starting from the measured open circuit potential of each species in the direction indicated by the arrows.

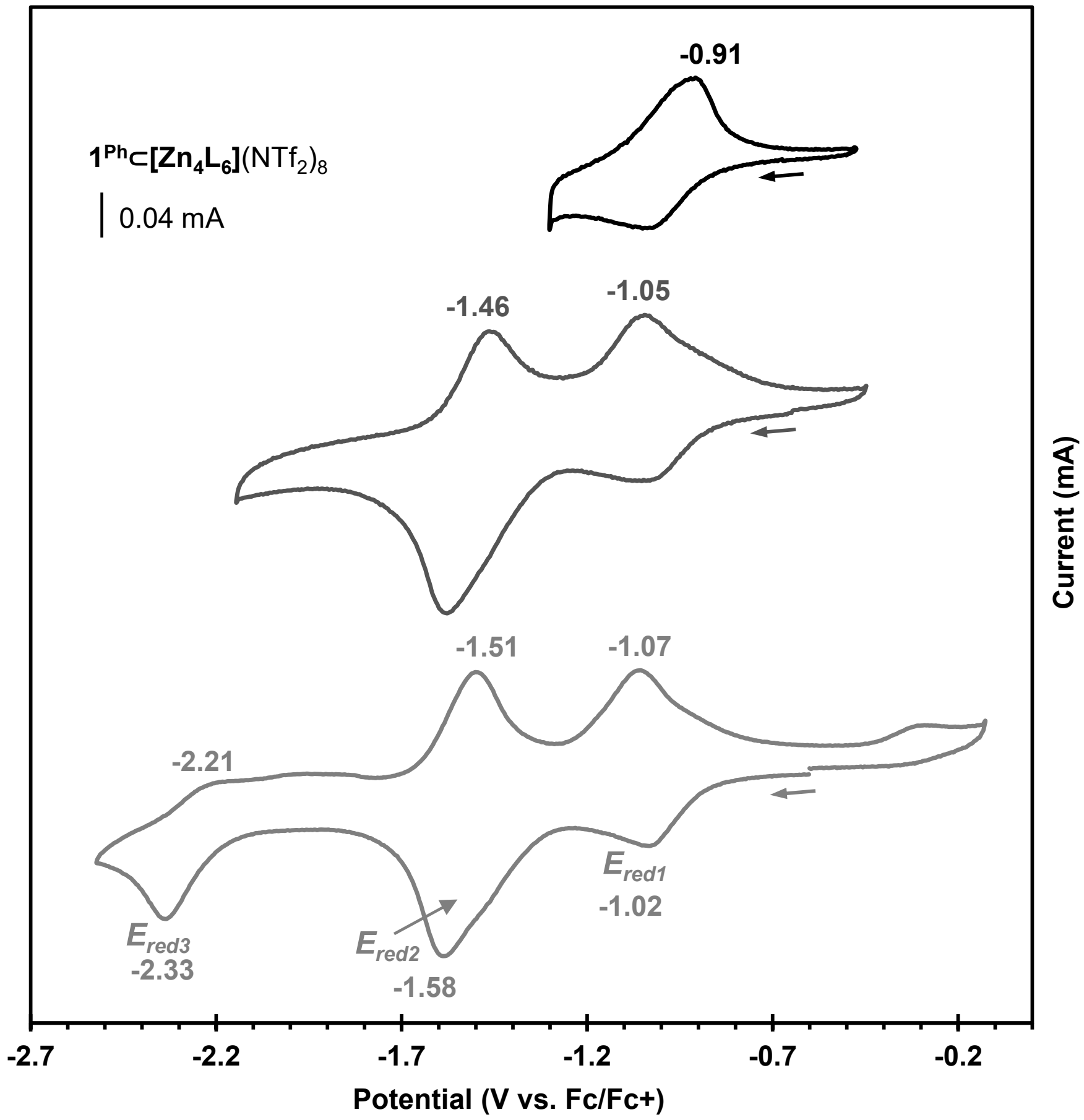

Figure S41. Cyclic voltammograms of $\mathbf{1^{Ph}\subset[Zn_4L_6]}(NTf_2)_8$ with different windows of scan up to E_{red3}, E_{red2} and E_{red1} showing the increase in reversibility of E_{red2} and E_{red1} on moving from wider to narrower window range. Conditions: 0.1 mM in MeCN; 0.1 M $N^{n}Bu_4PF_6$; 2 V/s. Scans were conducted starting from the measured open circuit potential of each species in the direction indicated by the arrows.

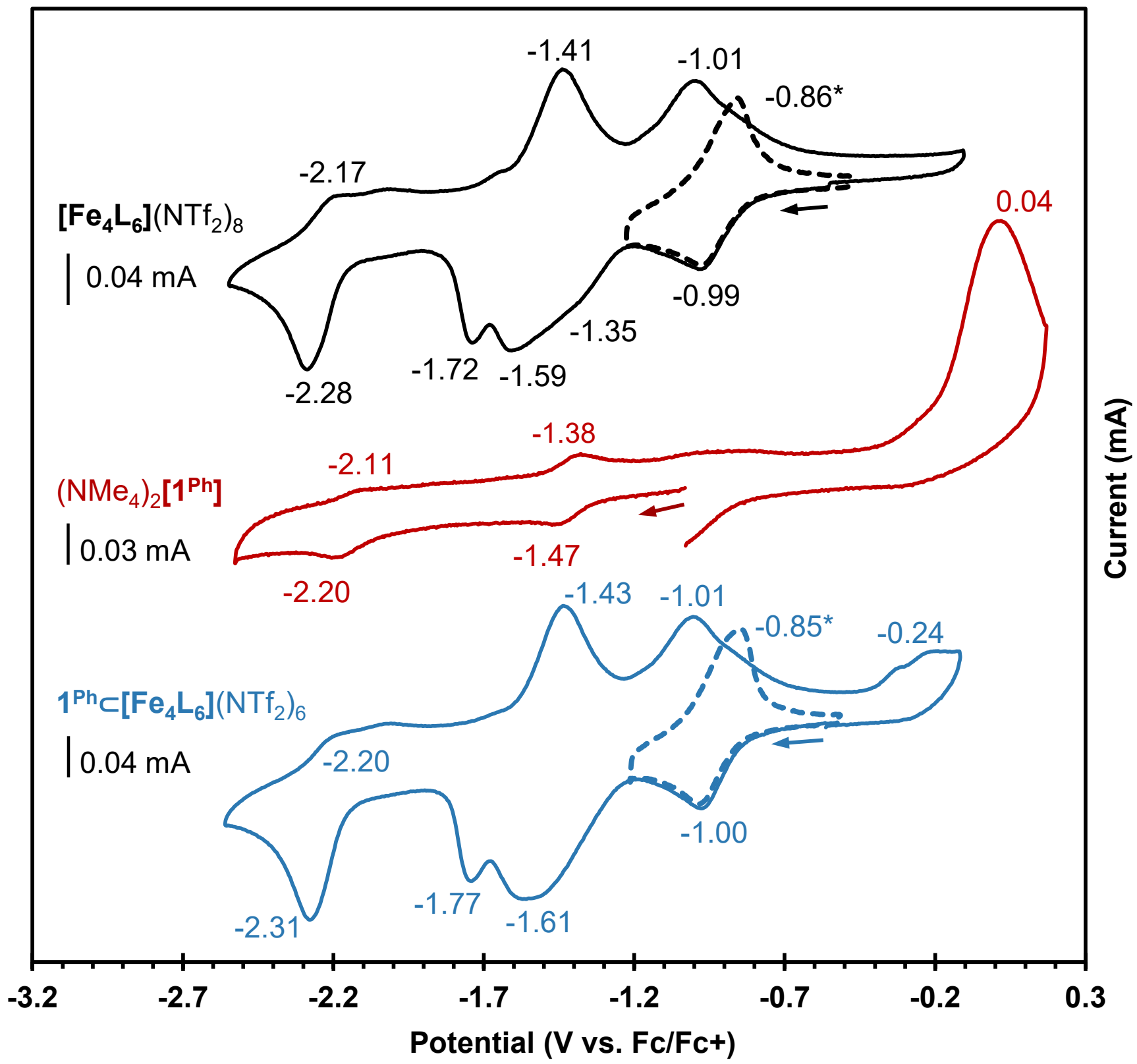

Figure S42. Cyclic voltammograms of **[Fe$_4$L$_6$]**(NTf$_2$)$_8$, (NMe$_4$)$_2$**[1^{Ph}]**, and **1^{Ph}⊂[Fe$_4$L$_6$]**(NTf$_2$)$_6$. Conditions: 0.1 mM in MeCN; 0.1 M N^{n}Bu$_4$PF$_6$; 2 V/s. Scans were conducted starting from the measured open circuit potential of each species in the direction indicated by the arrows. The first redox event for **[Fe$_4$L$_6$]**(NTf$_2$)$_8$ and **1^{Ph}⊂[Fe$_4$L$_6$]**(NTf$_2$)$_6$ is isolated and overlaid on the full-scan CV as dotted lines; the corresponding oxidation peaks are marked with asterisks.

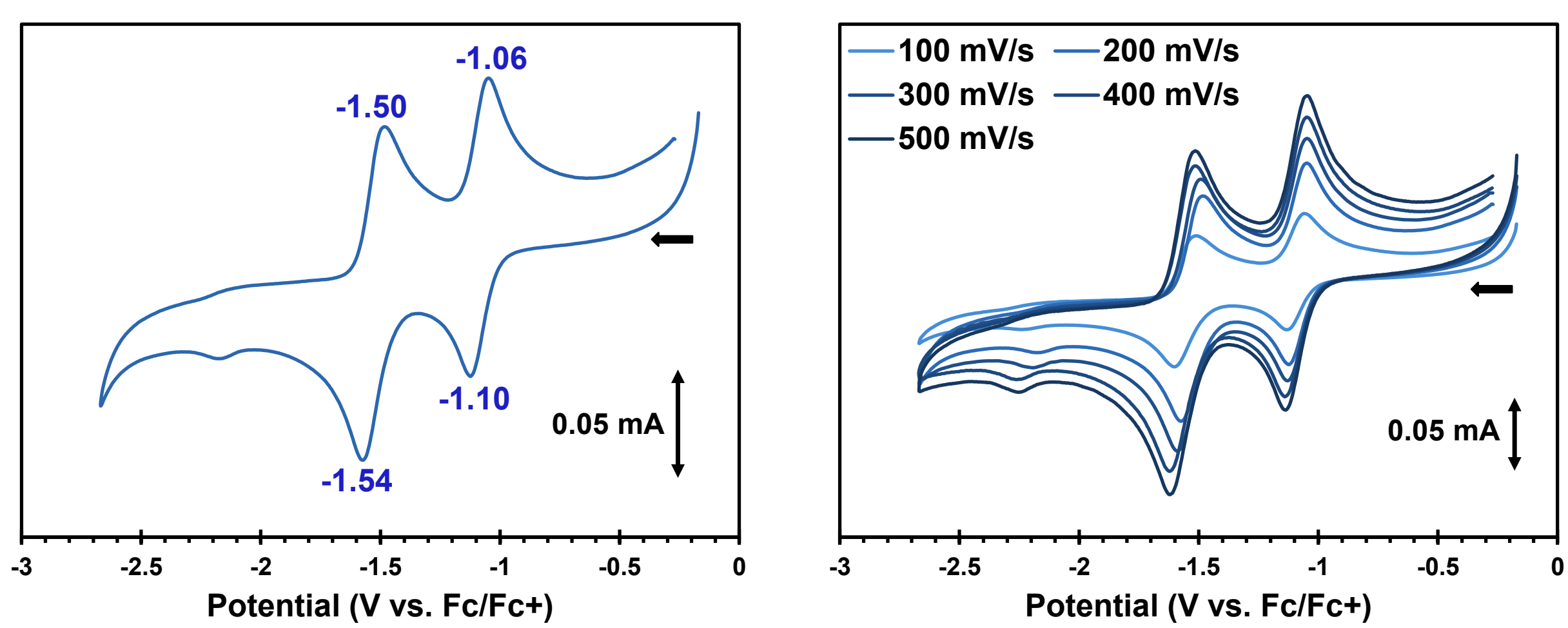

Figure S43. Cyclic voltammograms of diamine **A** (Scheme S1) recorded at 0.2 V/s (left), and at variable scan rates (right). The arrows denote the scan direction. Conditions: 1 mM in DMF; 0.1 M [NMe$_4$][PF$_6$].

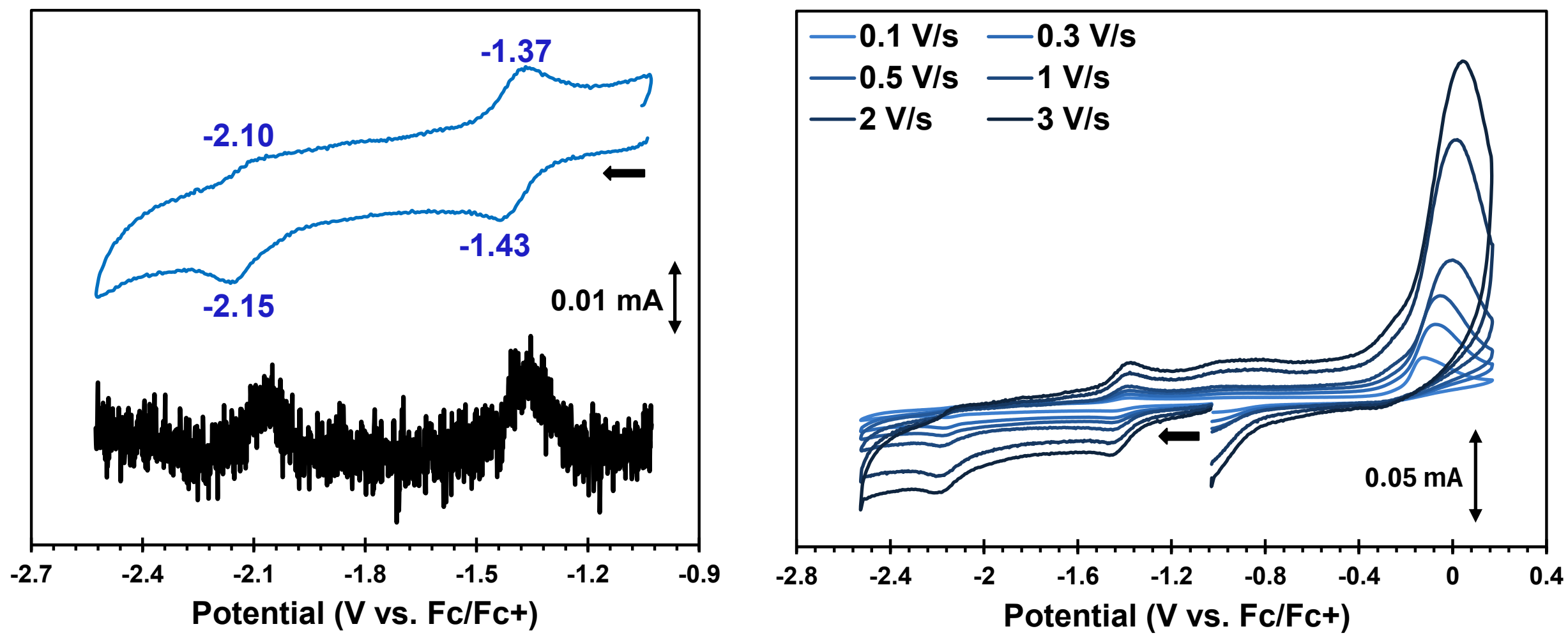

Figure S44. Cyclic voltammogram of $(NMe_4)_2$**[1^{Ph}]** at 1 V/s and (Right) Cyclic voltammogram at variable scan rates. The 1 V/s scan (left) is plotted above the differential pulse voltammogram. The arrows denotes the scan direction. Conditions: 0.1 mM in MeCN; 0.1 M $[N^nBu_4][PF_6]$.

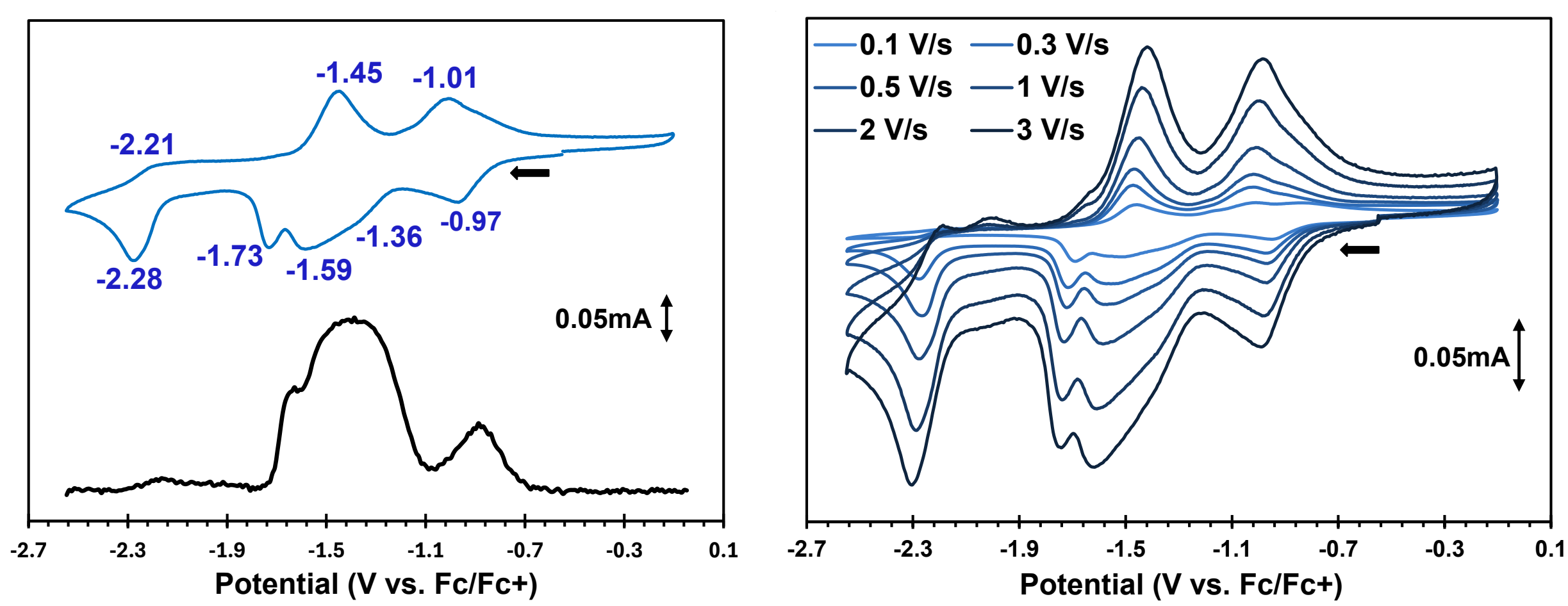

Figure S45. Cyclic voltammograms of **$[Fe_4L_6]$**$(NTf_2)_8$ recorded at 1 V/s (left) and at variable scan rates (right). The 1 V/s scan (left) is plotted above the differential pulse voltammogram. The arrows denote the scan direction. Conditions: 0.1 mM in MeCN; 0.1 M $[N^nBu_4][PF_6]$.

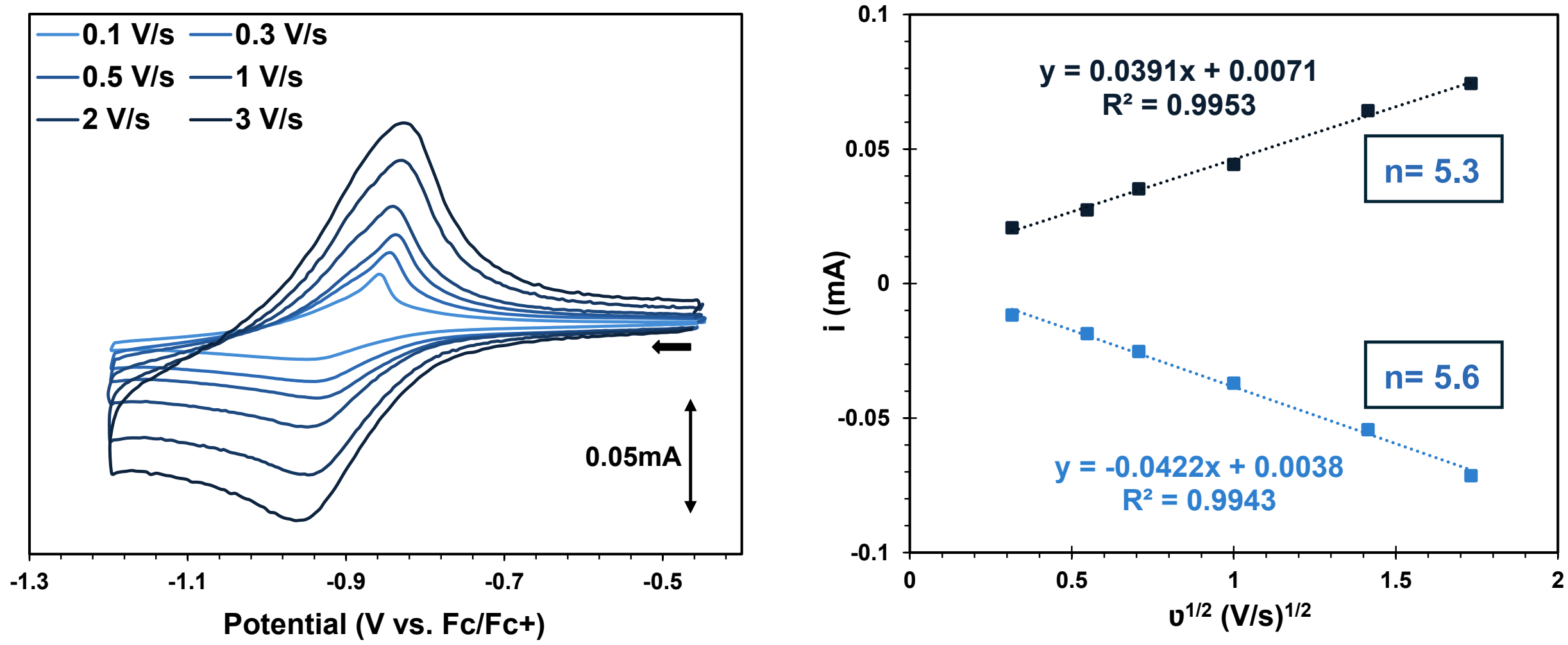

Figure S46. Left: Cyclic voltammogram of the first reduction event (E_{red1}) and corresponding oxidation event for **[Fe$_4$L$_6$]**(NTf$_2$)$_8$. The arrow denotes the scan direction. Conditions: 0.1 mM in MeCN; 0.1 M [N^{n}Bu$_4$][PF$_6$]. Right: Corresponding Randles Sevčik plot showing that approximately n = 6 electrons are transferred.

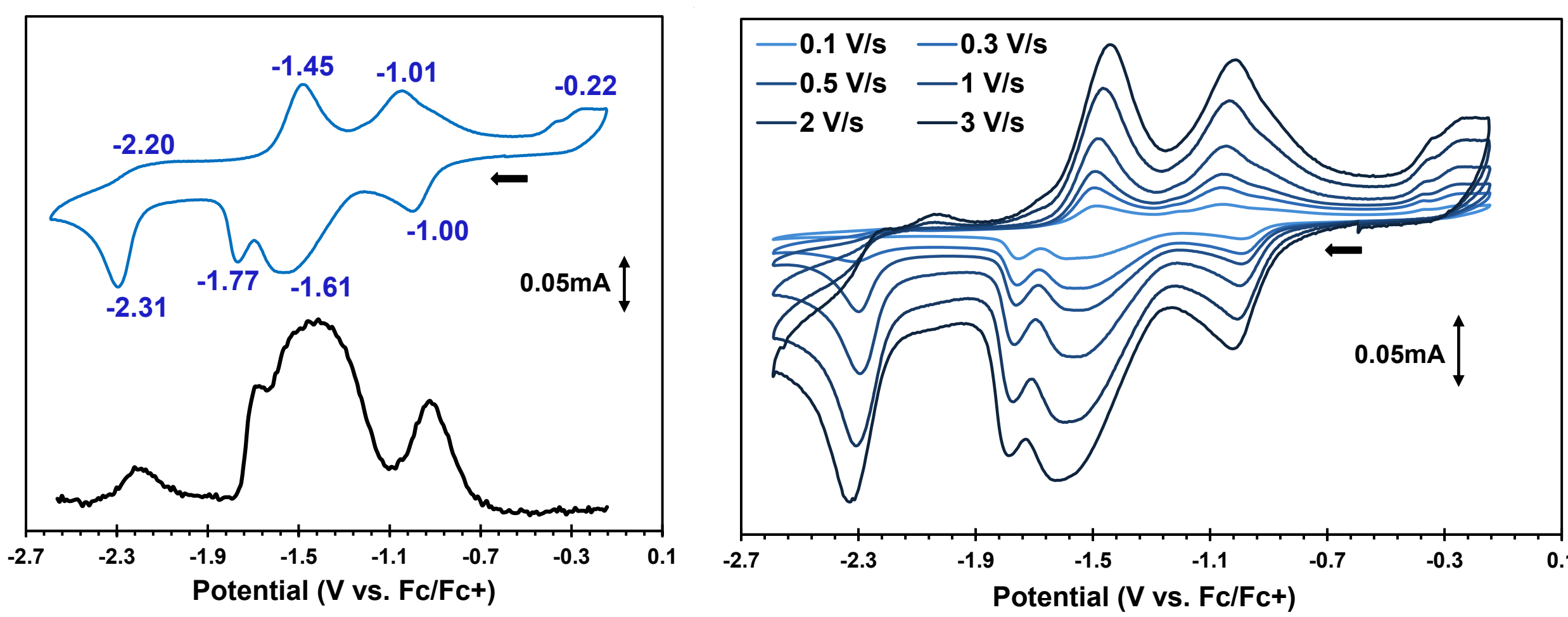

Figure S47. Cyclic voltammograms of **1^{Ph}⊂[Fe$_4$L$_6$]**(NTf$_2$)$_6$ recorded at 1 V/s (left) and at variable scan rates (right). The 1 V/s scan (left) is plotted above the differential pulse voltammogram. The arrows denote the scan direction. Conditions: 0.1mM in MeCN; 0.1 M [N^{n}Bu$_4$][PF$_6$].

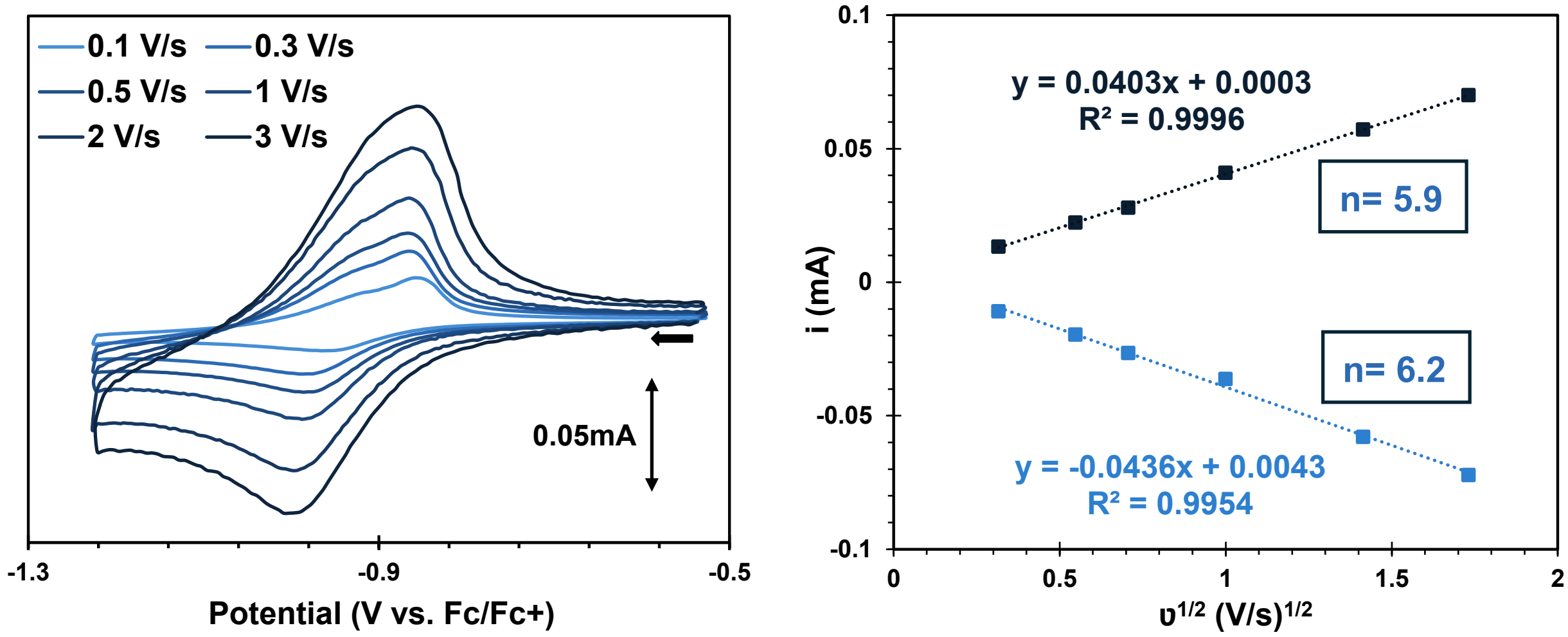

Figure S48. Left: Cyclic voltammogram of the first reduction event (E_{red1}) and corresponding oxidation event for $\mathbf{1^{Ph}}\subset\mathbf{[Fe_4L_6]}(NTf_2)_6$ (left). The arrow denotes the scan direction. Conditions: 0.1 mM in MeCN; 0.1 M $[N^nBu_4][PF_6]$. Right: Corresponding Randles Sevčik plot showing that approximately $n = 6$ electrons are transferred.

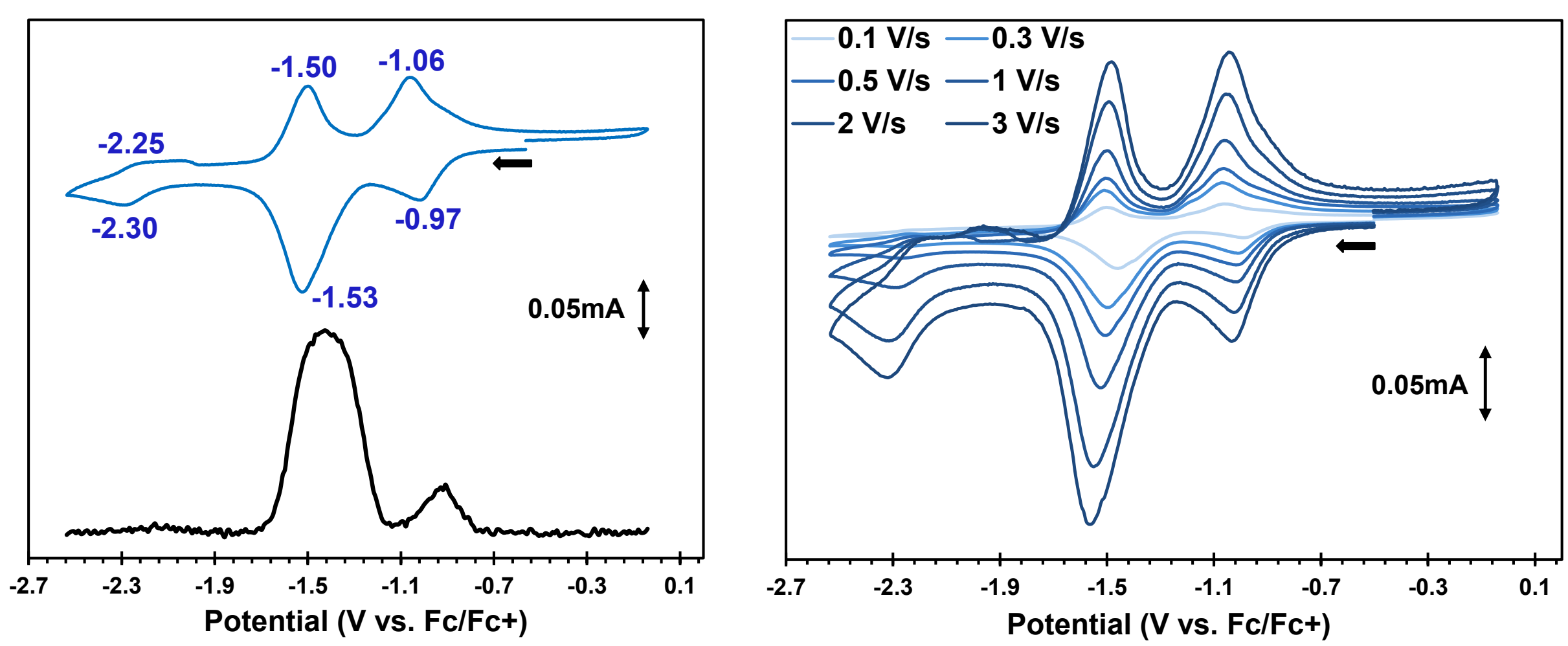

Figure S49. Cyclic voltammograms of $\mathbf{[Zn_4L_6]}(NTf_2)_8$ recorded at 1 V/s (left) and at variable scan rates (right). The 1 V/s scan (left) is plotted above the differential pulse voltammogram. The arrows denotes the scan direction. Conditions: 0.1mM in MeCN; 0.1 M $[N^nBu_4][PF_6]$.

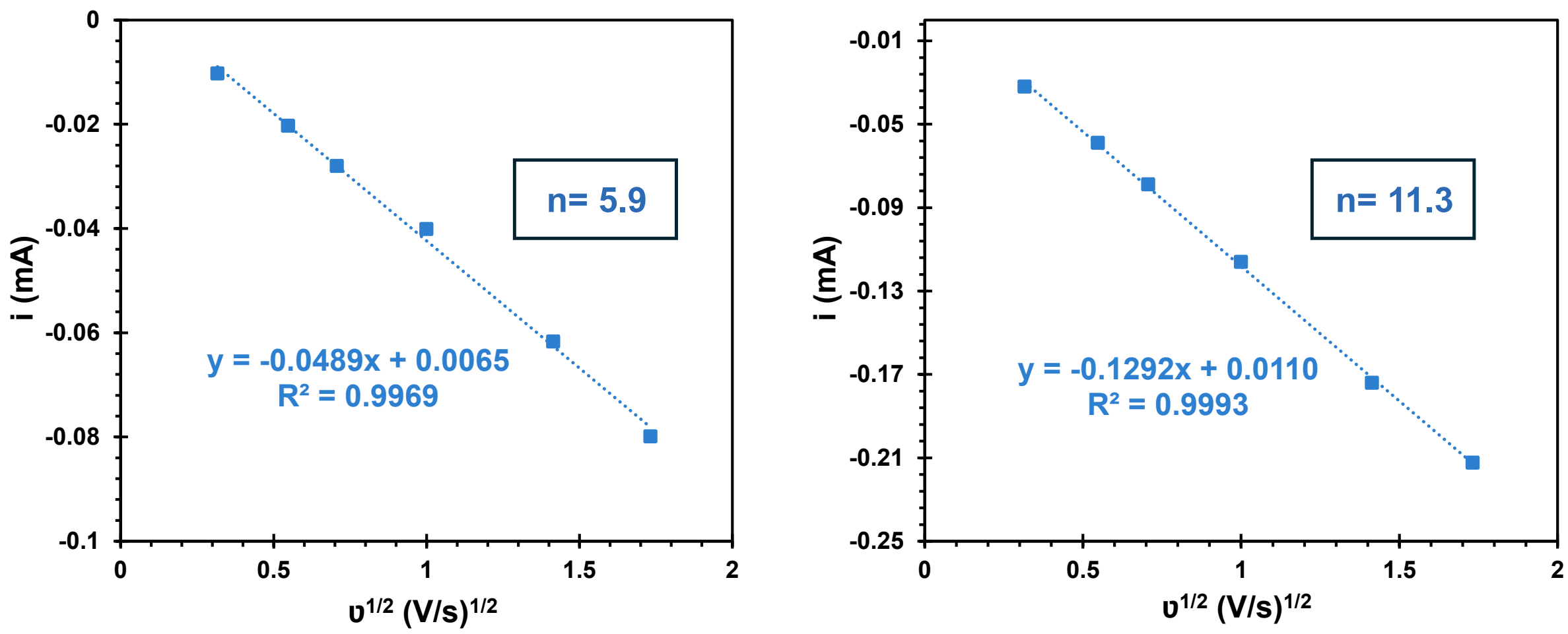

Figure S50. Randles-Sevčik plot for the first (E_{red1}, left) and second (E_{red2}, right) reduction event of **[Zn$_4$L$_6$]**(NTf_2)$_6$ showing a linear relationship and that approximately n = 6 and 11 electrons are transferred per event, respectively.

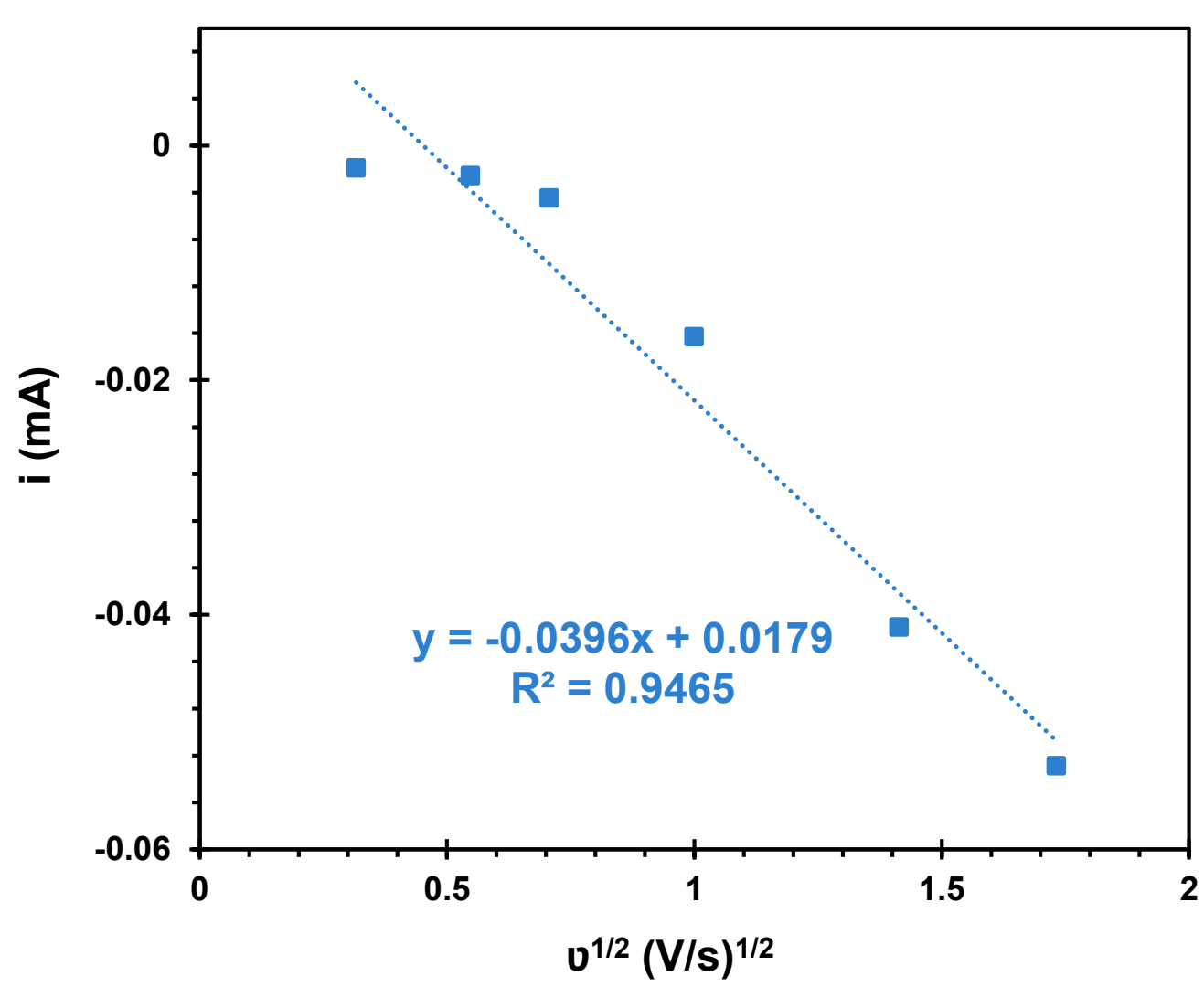

Figure S51. Randles-Sevčik plot for the third reduction event (E_{red3}) of **[Zn$_4$L$_6$]**(NTf_2)$_6$ showing a non-linear relationship consistent with competing precipitation at these low applied potentials.

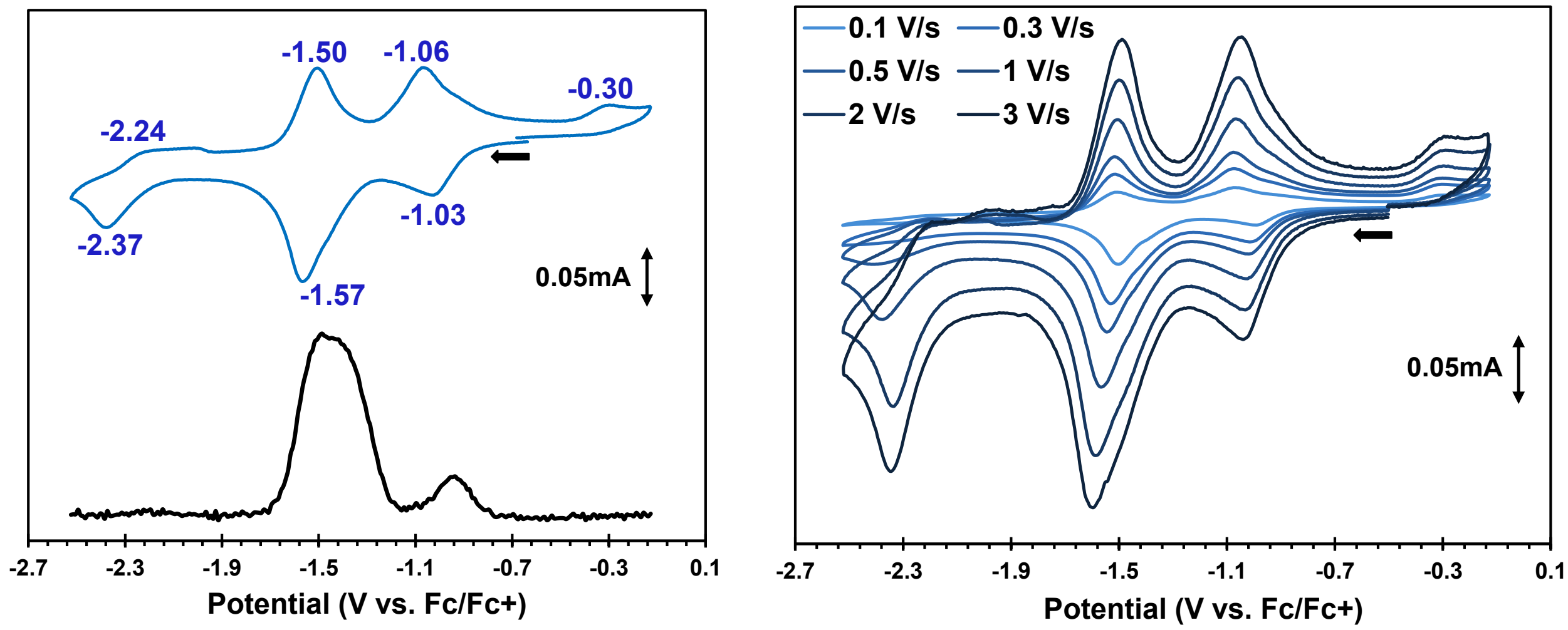

Figure S52. Cyclic voltammograms of **1^{Ph}⊂[Zn_4L_6]**(NTf_2)$_6$ recorded at 1 V/s (left) and at variable scan rates (right). The 1 V/s scan (left) is plotted above the differential pulse voltammogram. The arrows denotes the scan direction. Conditions: 0.1mM in MeCN; 0.1 M [N^nBu_4][PF_6].

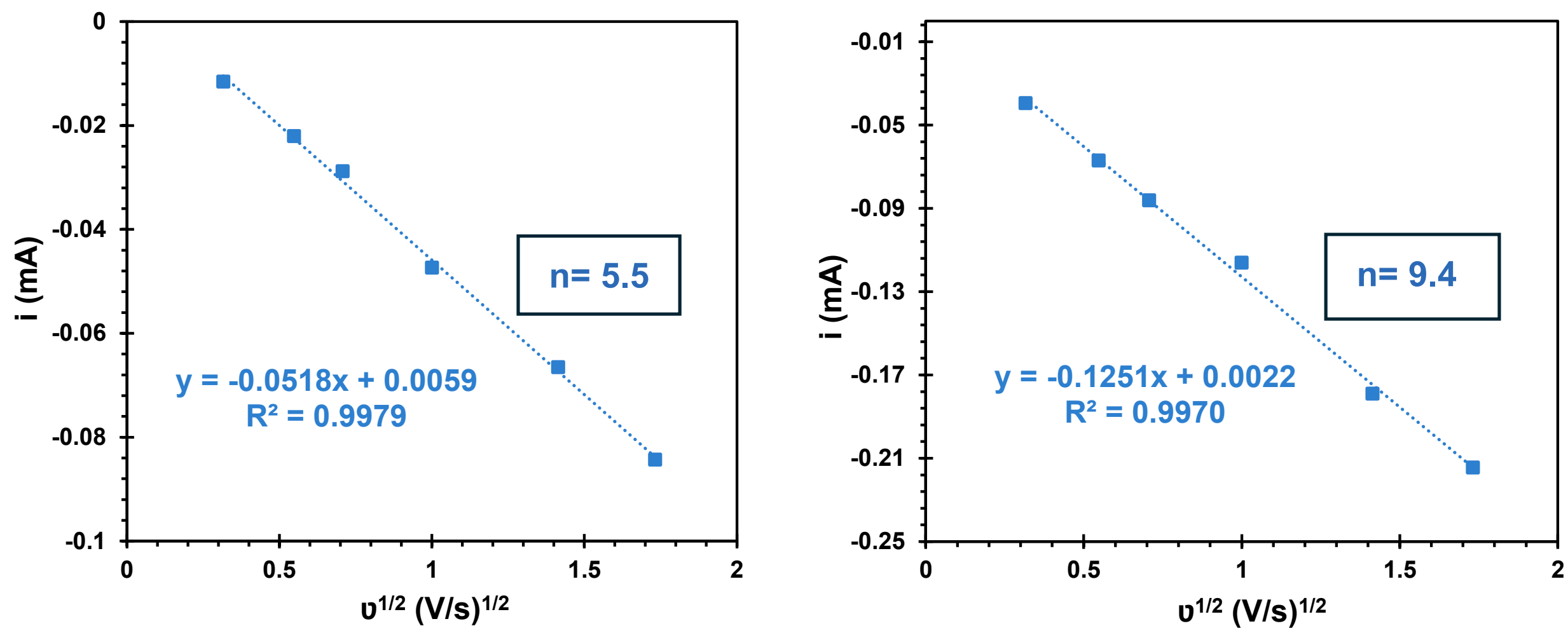

Figure S53. Randles-Sevčik plot for the first (E_{red1}, left) and second (E_{red2}, right) reduction event of **1^{Ph}⊂[Zn_4L_6]**(NTf_2)$_6$ showing a linear relationship and that approximately n = 6 and 9 electrons are transferred per event, respectively.

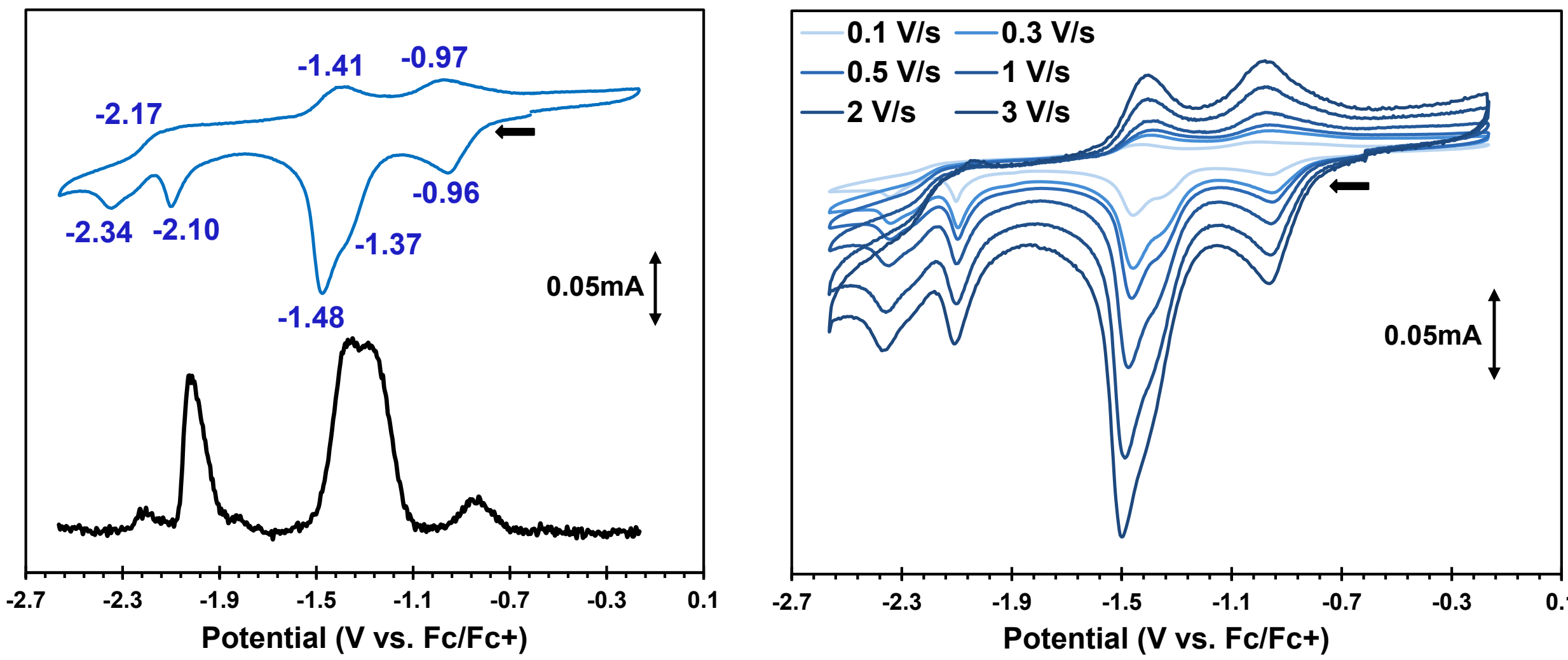

Figure S54. Cyclic voltammograms of **[Ni$_4$L$_6$]**(NTf$_2$)$_8$ recorded at 1 V/s (left) and at variable scan rates (right). The 1 V/s scan (left) is plotted above the differential pulse voltammogram. The arrows denotes the scan direction. Conditions: 0.1mM in MeCN; 0.1 M [N^{n}Bu$_4$][PF$_6$].

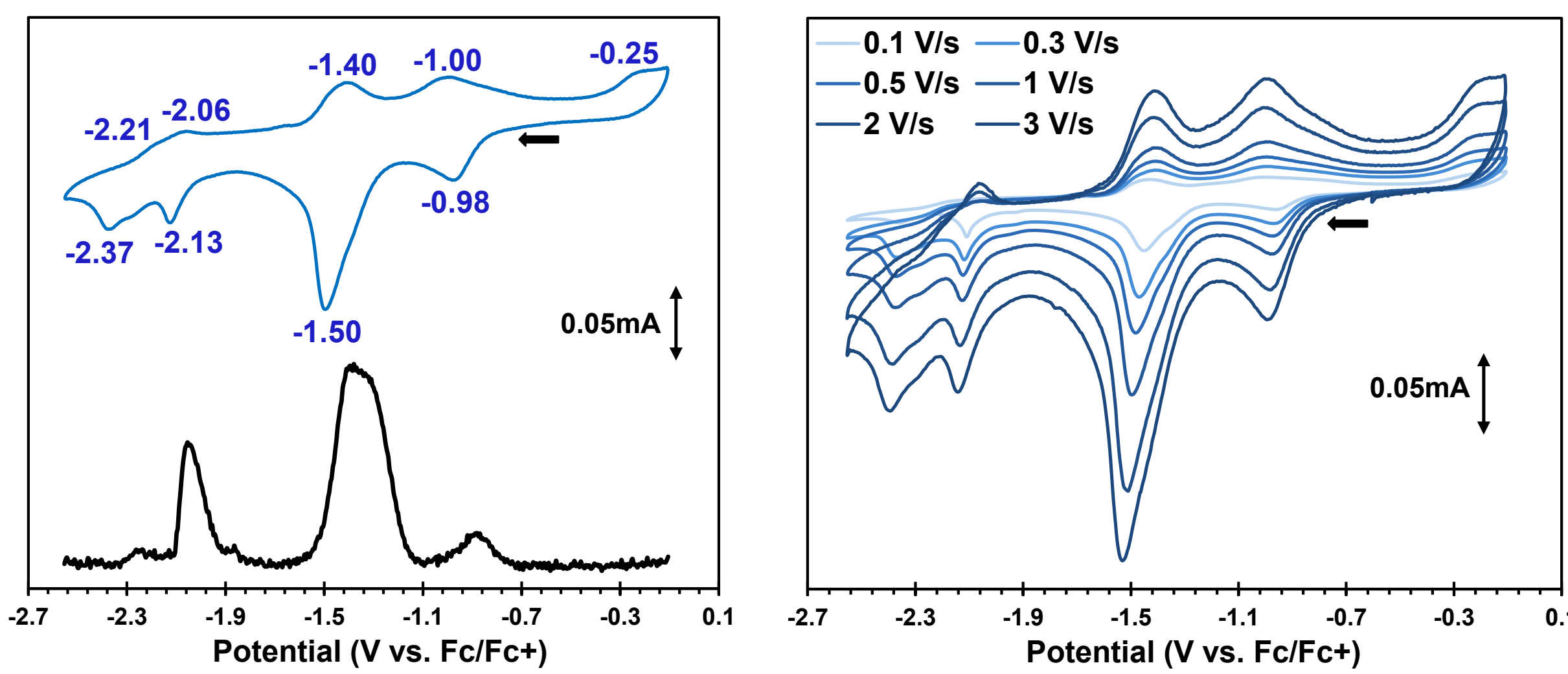

Figure S55. Cyclic voltammograms of **$1^{Ph}\subset$[Ni$_4$L$_6$]**(NTf$_2$)$_6$ recorded at 1 V/s (left) and at variable scan rates (right). The 1 V/s scan (left) is plotted above the differential pulse voltammogram. The arrows denotes the scan direction. Conditions: 0.1mM in MeCN; 0.1 M [N^{n}Bu$_4$][PF$_6$].

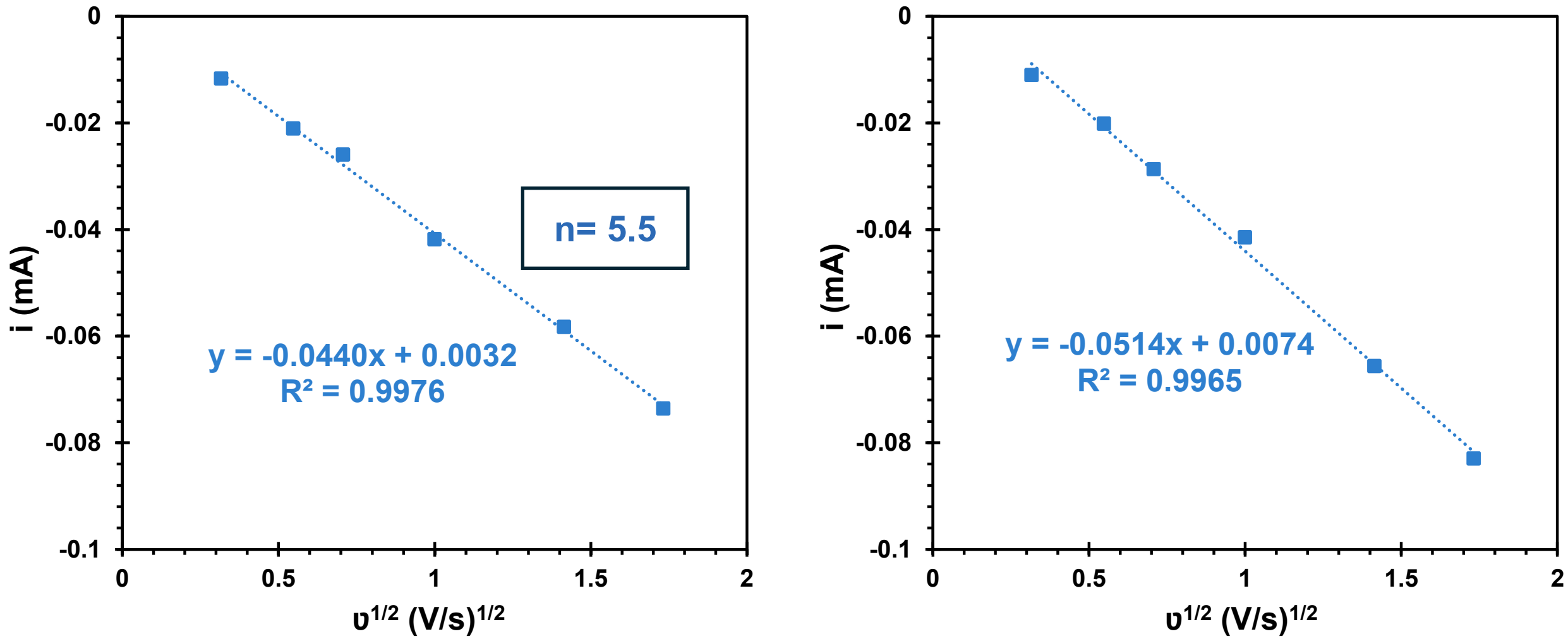

Figure S56. Randles-Sevčik plot for the first (E_{red1}) reduction event of **[Ni_4L_6]**$(NTf_2)_8$ (left) and **1^{Ph}⊂[Ni_4L_6]**$(NTf_2)_6$ (right) showing a linear relationship and that approximately n = 6 and 9 electrons are transferred per event, respectively.

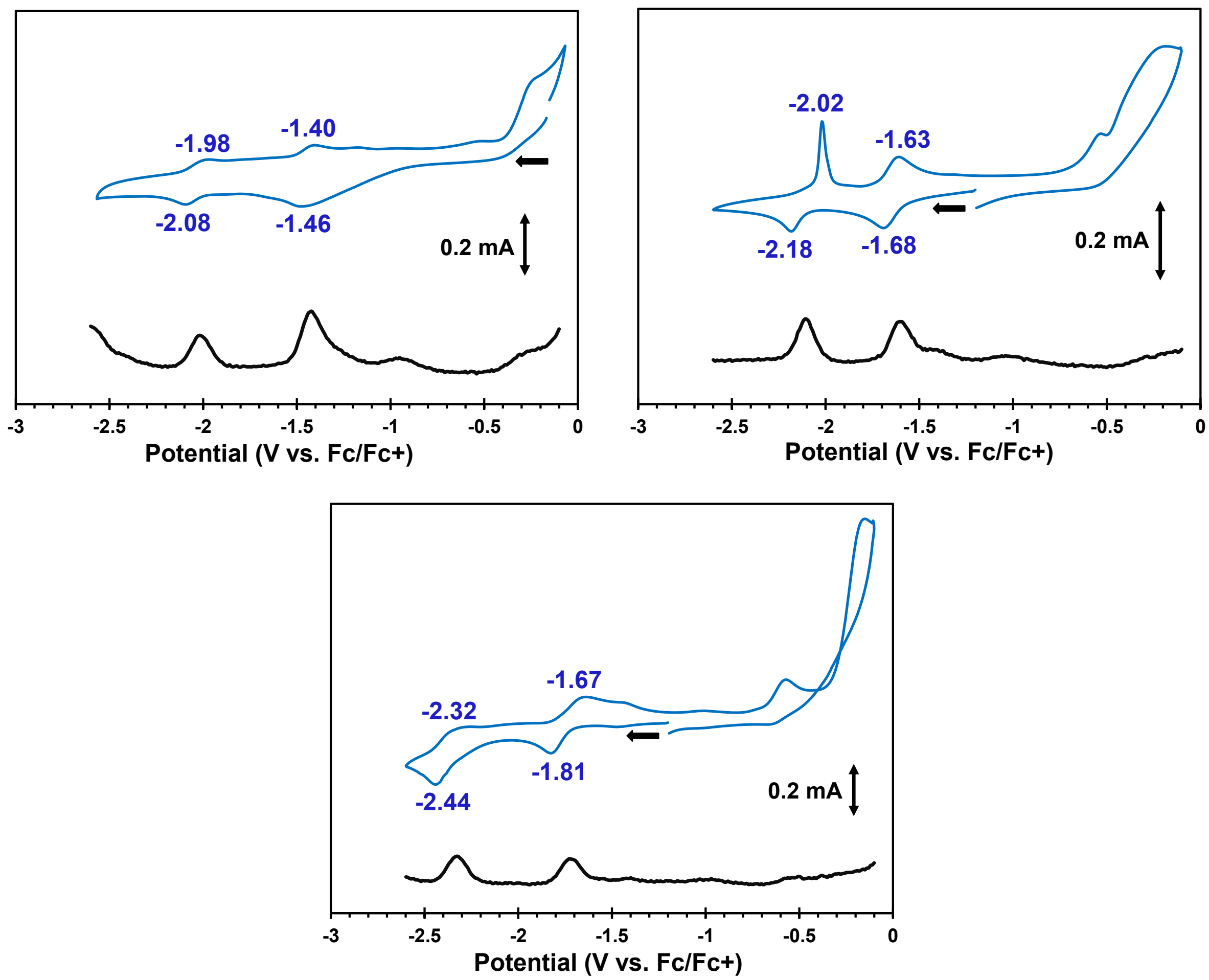

Figure S57. Cyclic voltammograms of $(NMe_4)_2$**[1^{Ph}]**, (top, left) $(NMe_4)_2$**[1^{OH}]** (top, right), and $(NMe_4)_2$**[1^{tBu}]** (bottom) at 0.2 V/s scan rate are plotted above the differential pulse voltammogram. The arrow denotes the scan direction. Conditions: 1mM in DMF; 0.1 M $[NMe_4][PF_6]$.

S7. REDOX TITRATIONS

General Procedure. These experiments were performed similarly to those reported in the literature for host-guest complexes synthesized using other NDI-based cages.[19,20] A CD_3CN solution of 3.3 mM **[Fe$_4$L$_6$]**$(NTf_2)_6$ or **1^R⊂[Fe$_4$L$_6$]**$(NTf_2)_6$ or **[Fe$_4$L$_6$][1^{tBu}]**$(NTf_2)_6$ (0.5 mL) was transferred into a J-Young NMR tube. Reductions were carried out via consecutive addition of aliquots from a $CoCp_2$ stock solution (59.4 mM in CD_3CN; 56 µL, 2 equiv), with vigorous shaking after each addition to ensure complete mixing prior to ^1H NMR analysis. Each addition was accompanied by a gradual change in color, from purple to dark brown for **[Fe$_4$L$_6$]**$(NTf_2)_6$ or magenta to brown for **1^R⊂[Fe$_4$L$_6$]**$(NTf_2)_6$ and **[Fe$_4$L$_6$][1^{tBu}]**$(NTf_2)_6$, together with the formation of a precipitate. ^1H NMR analysis revealed gradual broadening and disappearance of the starting analyte signals due to paramagnetic relaxation effects together with the appearance of the expected signal for cobaltocene (δ_H 5.7 ppm). The redox titration was continued until 6 equiv $CoCp_2$ were added, at which point the signal for cobaltocene broadened and shifted slightly upfield (4.8 ppm) indicating that a slight excess of $CoCp_2$ had been added. Subsequently, back-oxidation was performed similarly by successive addition of aliquots from a $AgNTf_2$ stock solution (59.4 mM in CD_3CN; 56 µL, 2 equiv), which improved the solubility of the complexes and led to the reappearance of ^1H NMR signals for the starting analyte. Some solids remained after the addition of 6 or more equiv $AgNTf_2$. To maximize the quality of the final ^1H NMR spectrum, the solutions were filtered prior to acquisition.

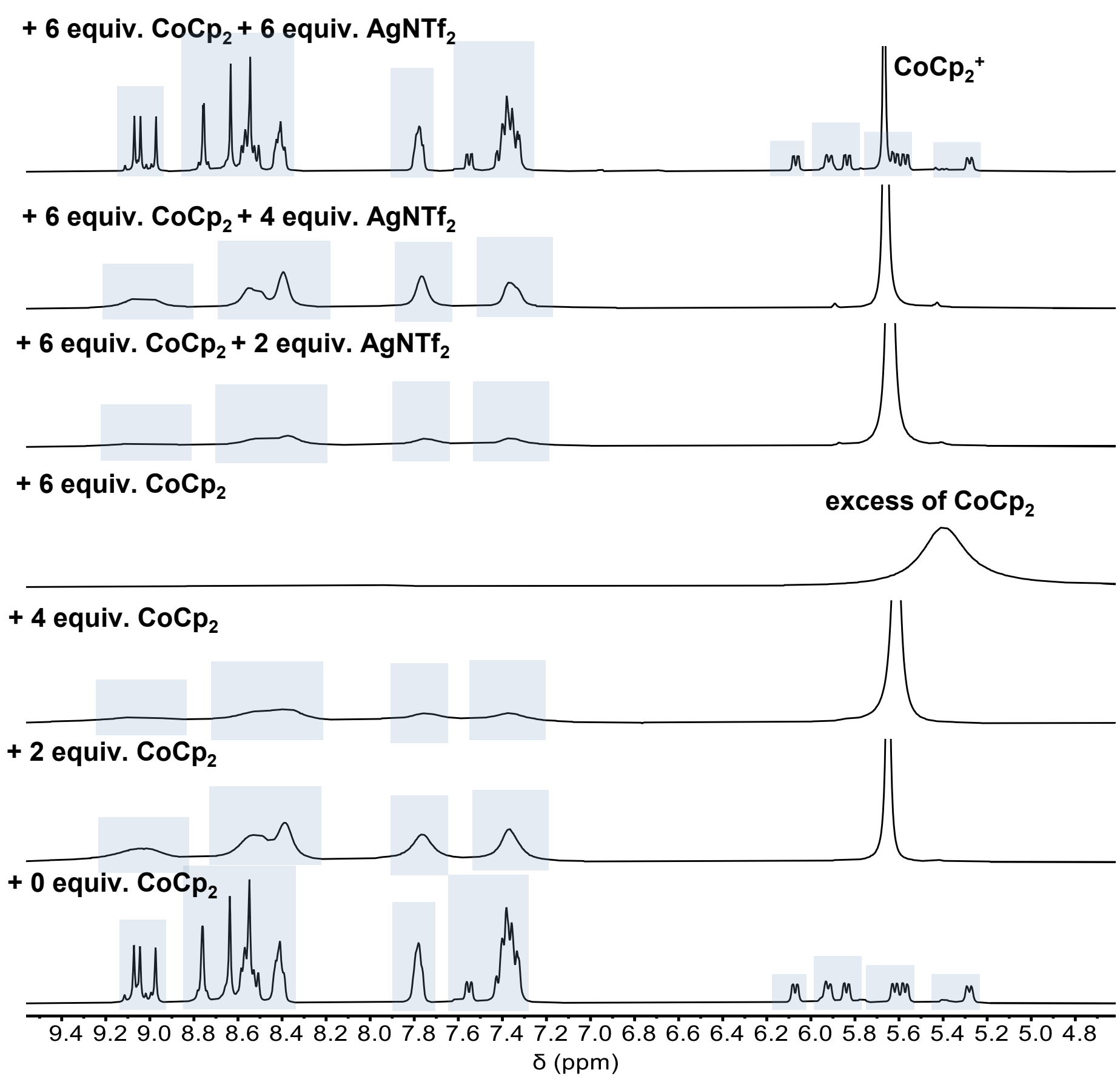

Figure S58. ^1H NMR spectra (400 MHz, CD_3CN) showing the results of a redox titration study in which **[Fe$_4$L$_6$]**$(NTf_2)_8$ was reduced with $CoCp_2$ (6 equiv) and then re-oxidized with $AgNTf_2$, leading to complete regeneration of the cage signals (highlighted in blue).

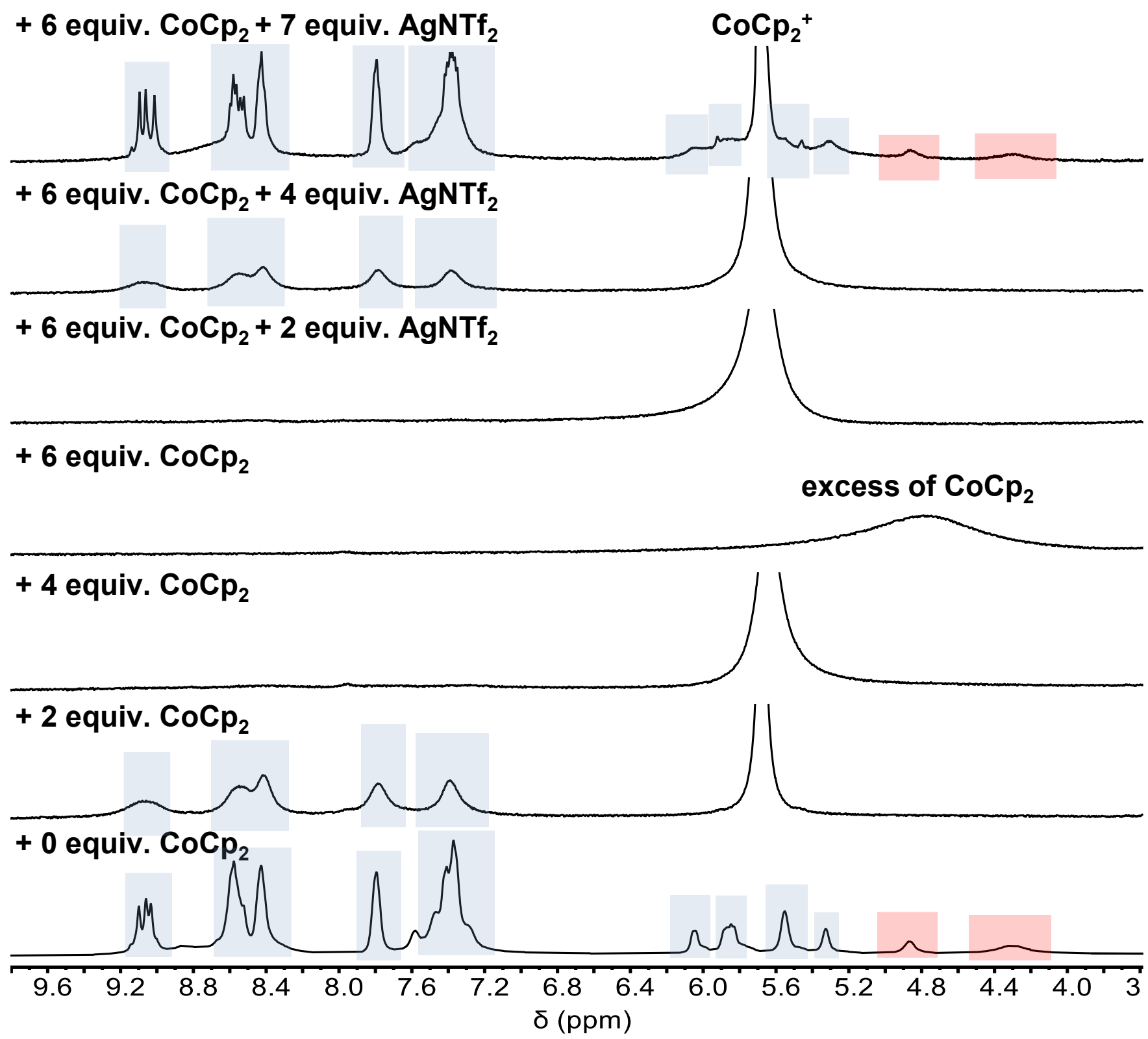

Figure S59. ^{1}H NMR spectra (400 MHz, CD_3CN) showing the results of a redox titration study in which $\mathbf{1^{Ph}}\subset\mathbf{[Fe_4L_6]}(NTf_2)_6$ was reduced with $CoCp_2$ (6 equiv) and then re-oxidized with $AgNTf_2$, leading to complete regeneration of the cage signals (highlighted in blue). Following reoxidation, ca. 94% of encapsulated $\mathbf{[1^{Ph}]^{2-}}$ remained as judged by integration of *p*-C*H* peak of $\mathbf{[1^{Ph}]^{2-}}$ at 4.85 ppm vs. the cage *py* C*H* peak at 7.79 ppm.[a] residual Et_2O; *unidentified impurities.

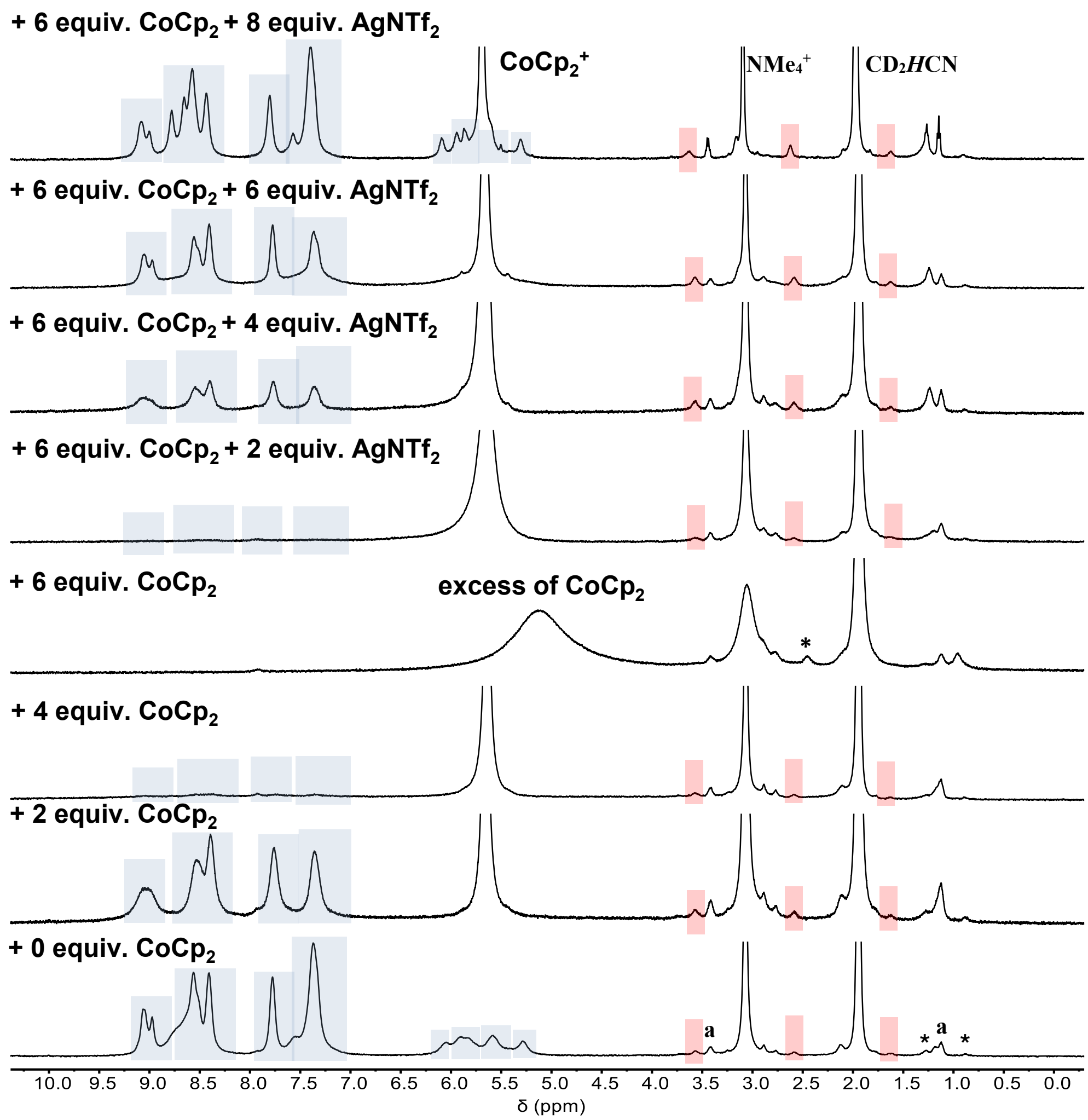

Figure S60. ^{1}H NMR spectra (400 MHz, CD_3CN) showing the results of a redox titration study in which $\mathbf{1^{OH}}$**⊂[Fe$_4$L$_6$]**$(NTf_2)_6$ was reduced with $CoCp_2$ (6 equiv) and then re-oxidized with $AgNTf_2$, leading to complete regeneration of the cage signals (highlighted in blue). Following reoxidation, ca. 123% of the original encapsulated $\mathbf{[1^{OH}]^{2-}}$ was present as judged by integration of SCH_2CH_2OH peak of $\mathbf{[1^{OH}]^{2-}}$ at 2.58 ppm vs. the cage *py* C*H* peak at 7.79 ppm, consistent with either cage degradation under the reaction conditions or an improvement in signal quality as a result of filtration. [a] residual Et_2O; *unidentified impurities.

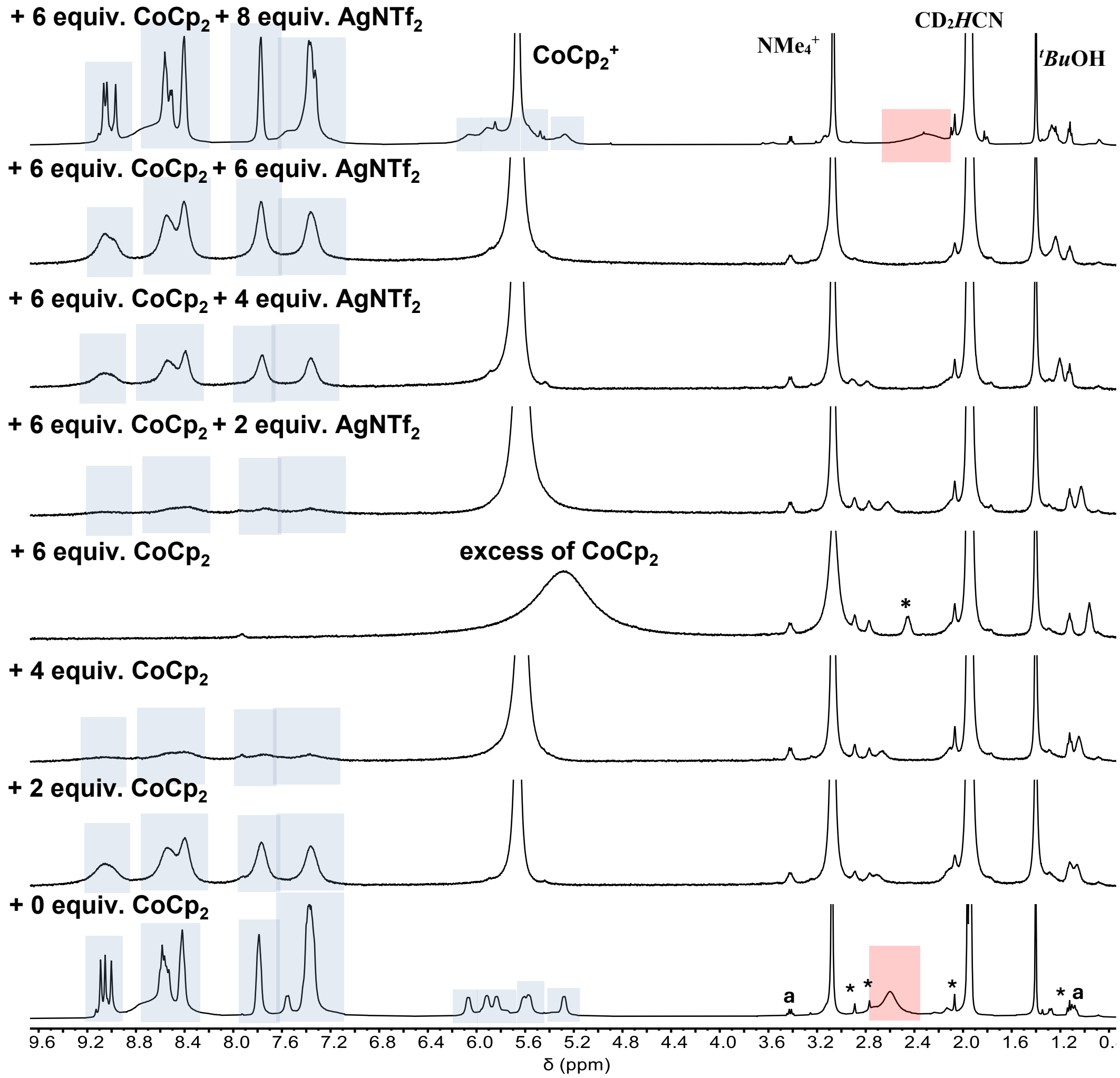

Figure S61. ^{1}H NMR spectra (400 MHz, CD_3CN) showing the results of a redox titration study in which **$[Fe_4L_6][1^{tBu}]$**$(NTf_2)_6$ was reduced with $CoCp_2$ (6 equiv) and then re-oxidized with $AgNTf_2$, leading to complete regeneration of the cage signals (highlighted in blue). Following reoxidation, ca. 70% of encapsulated **$[1^{tBu}]^{2-}$** remained as judged by integration of *p*-C*H* peak of **$[1^{tBu}]^{2-}$** at 2.61 ppm vs. the cage *py* C*H* peak at 7.79 ppm, consistent with either cluster degradation and/or peak broadening under these conditions. [a] residual Et_2O; *unidentified impurities.

S8. ^{57}Fe MÖSSBAUER SPECTROSCOPY

^{57}Fe Mössbauer spectra were recorded at 70 or 200 K using a SEE Co. MS6 Mössbauer spectrometer with a Janis SVT-400 Nitrogen-shielded liquid Helium cryostat. The data were collected without an external magnetic field and referenced to Fe metal. The samples were prepared in an N_2-filled glovebox by grinding polycrystalline samples of the analyte (20-50 mg) into a fine powder and diluting with SpectroBlend® powder (Chemplex; 1:5 volume ratio) using a mortar and pestle. The powder mixtures were then transferred to a polyethylene sample holder. Following transfer from the glovebox, prepared samples were immediately frozen in liquid N_2 and kept cold while loading into the spectrometer to minimize air oxidation. Inside the spectrometer, the samples were shielded from the atmosphere using a continuous supply of liquid nitrogen. Spectra were simulated using two pairs of symmetric quadrupole doublets with equal populations and Lorentzian lineshapes. The fits were refined via least squares optimization to a minimum root mean square deviation (σ) using custom software developed by the Institute for Rock Magnetism at UMN (mossferret_ferret.exe). Reasonable fits were obtained assuming either (i) two quadrupole doublets with similar isomer shift (δ) and different quandrupole splitting (ΔE_Q) or (ii) similar ΔE_Q and different δ. We favor the former on the basis of prior literature reports of synthetic and biological $[Fe_4S_4]$ clusters reporting similar δ for mixed-valence $[Fe^{2.5+}{}_2S_2]^+$ states.[21]

Sample preparation. The host-guest complexes and ion pair were isolated as crystalline solids on a 38.5 mmol scale. In a representative procedure, the complex $\mathbf{1^{Ph}{\subset}[Zn_4L_6]}(NTf_2)_6$ was prepared by mixing $\mathbf{[Zn_4L_6]}(NTf_2)_8$ (240 mg, 38.5 mmol, 1 equiv) and $(NMe_4)_2\mathbf{[1^{Ph}]}$ (36 mg, 38.5 mmol, 1 equiv) in MeCN (15 mL), which resulted in a color change from light to dark brown. After stirring for 2 h at RT, the reaction mixture was filtered through a celite plug and the product precipitated from the filtrate via the addition of Et_2O (1:2 v/v vs. MeCN) at 0 °C. Filtration afforded clean $\mathbf{1^{Ph}{\subset}[Zn_4L_6]}(NTf_2)_6$ as a brown solid.

$\mathbf{1^{Ph}{\subset}[Ni_4L_6]}(NTf_2)_6$, $\mathbf{1^{OH}{\subset}[Zn_4L_6]}(NTf_2)_6$, and $\mathbf{[Ni_4L_6][1^{tBu}]}(NTf_2)_6$ were prepared similarly. We were unable to prepare $\mathbf{[Zn_4L_6][1^{tBu}]}(NTf_2)_6$ due to challenges associated with characterization, as described in section S3 above. The reduced host-guest complexes $\mathbf{1^{Ph}{\subset}[Zn_4L_6]}$, $\mathbf{1^{OH}{\subset}[Zn_4L_6]}$, and ion pair $\mathbf{[Ni_4L_6][1^{tBu}]}$ were prepared following the procedure described in Section S3 on a 38.5 mmol scale.

Table S3. ^{57}Fe Mössbauer parameters of clusters $(NMe_4)_2\mathbf{[1^R]}$ and complexes $\mathbf{1^R{\subset}[M_4L_6]}(NTf_2)_6$, $\mathbf{1^R{\subset}[M_4L_6]}$, $\mathbf{[Ni_4L_6][1^{tBu}]}(NTf_2)_6$, $\mathbf{[Ni_4L_6][1^{tBu}]}$ (M = Zn, Ni) taken at zero magnetic field.

@200K	δ, mm/s		ΔE_Q, mm/s		σ,[a] mm/s (×10^{-3})
	Fe1	Fe2	Fe1	Fe2	
$(NMe_4)_2\mathbf{[1^{Ph}]}$	0.37	0.38	0.52	0.87	1.47
$\mathbf{1^{Ph}{\subset}[Zn_4L_6]}(NTf_2)_6$	0.35	0.39	0.66	1.03	0.69
$\mathbf{1^{Ph}{\subset}[Zn_4L_6]}$	0.44	0.53	0.82	1.26	1.11
$(NMe_4)_2\mathbf{[1^{OH}]}$	0.36	0.38	1.19	1.01	0.63
$\mathbf{1^{OH}{\subset}[Zn_4L_6]}(NTf_2)_6$	0.31	0.35	0.53	1.07	0.68
$\mathbf{1^{OH}{\subset}[Zn_4L_6]}$	0.40	0.47	0.80	1.27	1.56
$(NMe_4)_2\mathbf{[1^{tBu}]}$	0.37	0.37	0.86	0.71	0.37
$\mathbf{[Ni_4L_6][1^{tBu}]}(NTf_2)_6$	0.36	0.38	0.78	1.12	0.65
$\mathbf{[Ni_4L_6][1^{tBu}]}$	0.38	0.40	0.68	1.01	1.0
**@70 K**	**δ, mm/s**		**ΔE_Q, mm/s**		**σ,[a] mm/s (×10^{-3})**
	Fe1	Fe2	Fe1	Fe2	
$(NMe_4)_2\mathbf{[1^{Ph}]}$	0.44	0.45	0.77	1.18	3.0
$\mathbf{1^{Ph}{\subset}[Ni_4L_6]}(NTf_2)_6$	0.46	0.47	0.93	1.25	1.29

[a]goodness-of-fit parameter

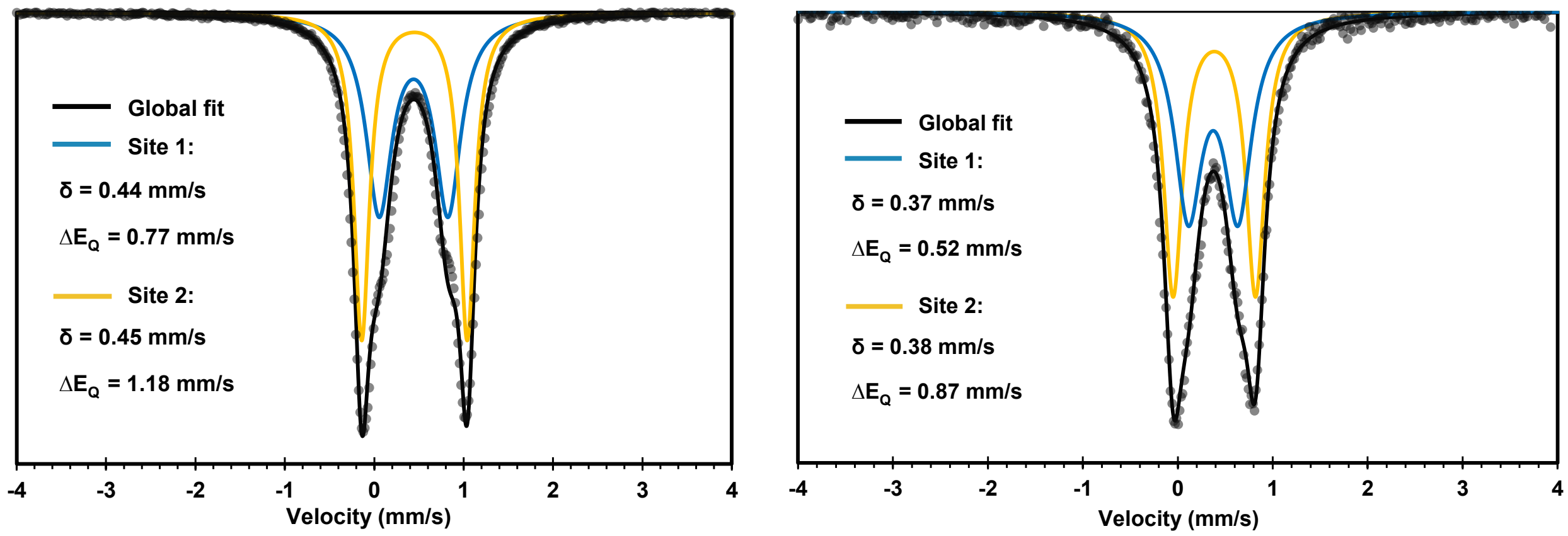

Figure S62. ^{57}Fe Mössbauer spectra of $(NMe_4)_2$**[1^{Ph}]** at 70 K (left) and 200 K (right).

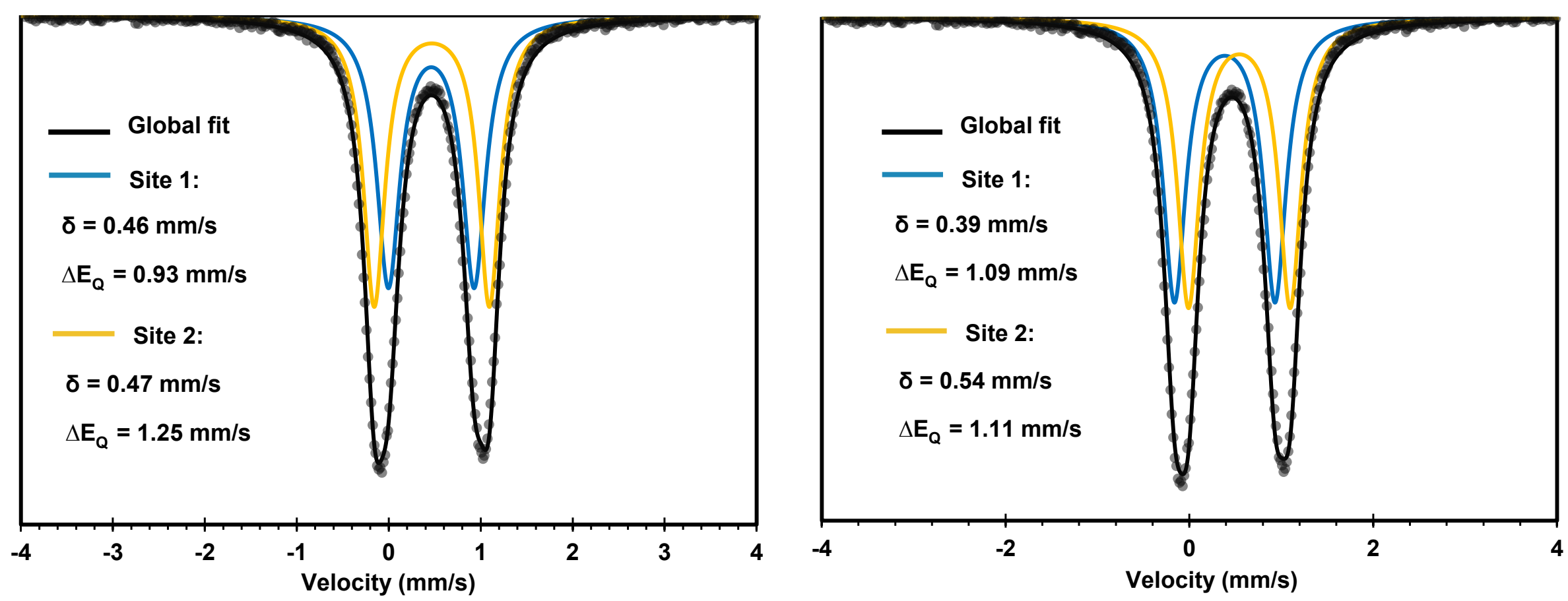

Figure S63. ^{57}Fe Mössbauer spectra of **1^{Ph}⊂[Ni$_4$L$_6$]**$(NTf_2)_6$ (70 K) with two alternate doublet fittings: similar δ (left) or similar ΔE_Q (right).

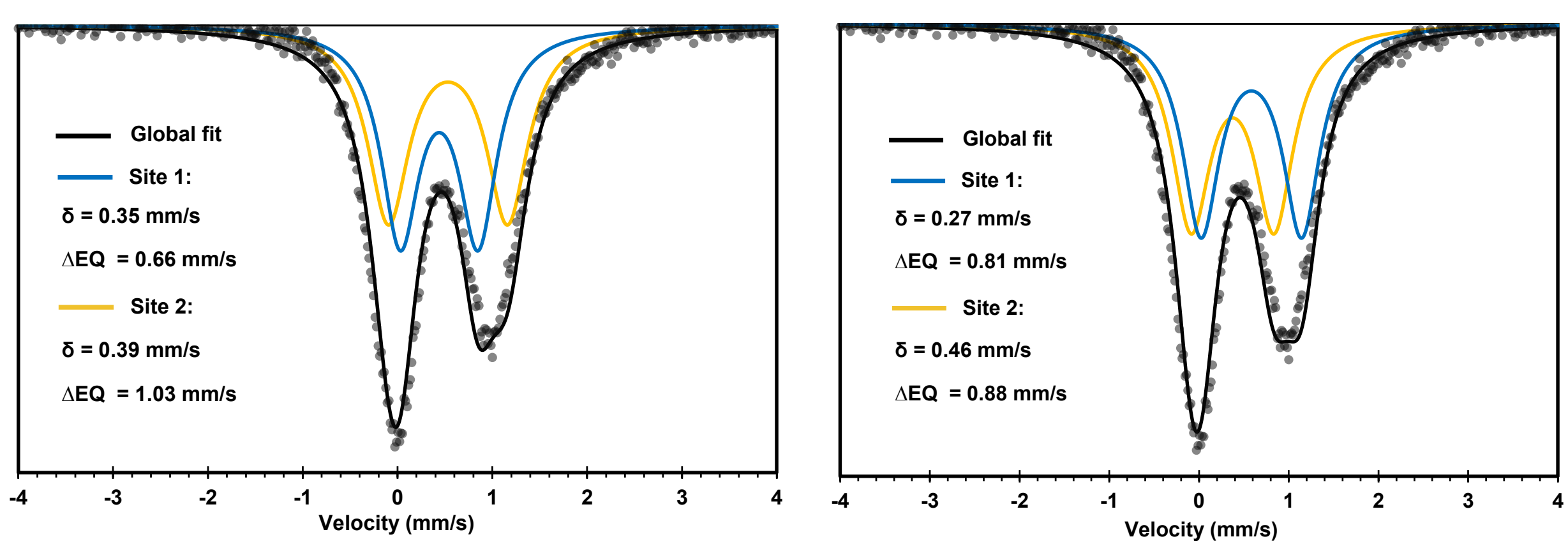

Figure S64. ^{57}Fe Mössbauer spectra of **1^{Ph}⊂[Zn$_4$L$_6$]**$(NTf_2)_6$ (200 K) with two alternate doublet fittings: similar δ (left) or similar ΔE_Q (right).

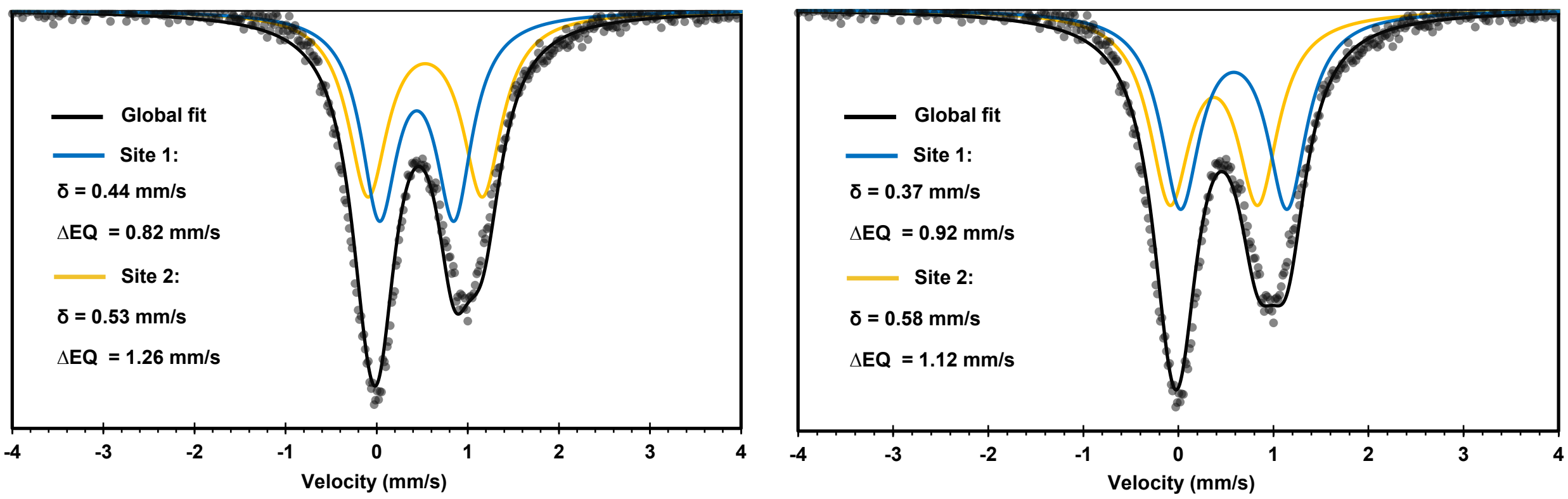

Figure S65. ^{57}Fe Mössbauer spectra of $\mathbf{1^{Ph}}\subset\mathbf{[Zn_4L_6]}$ (200 K) with two alternate doublet fittings: similar δ (left) or similar ΔE_Q (right).

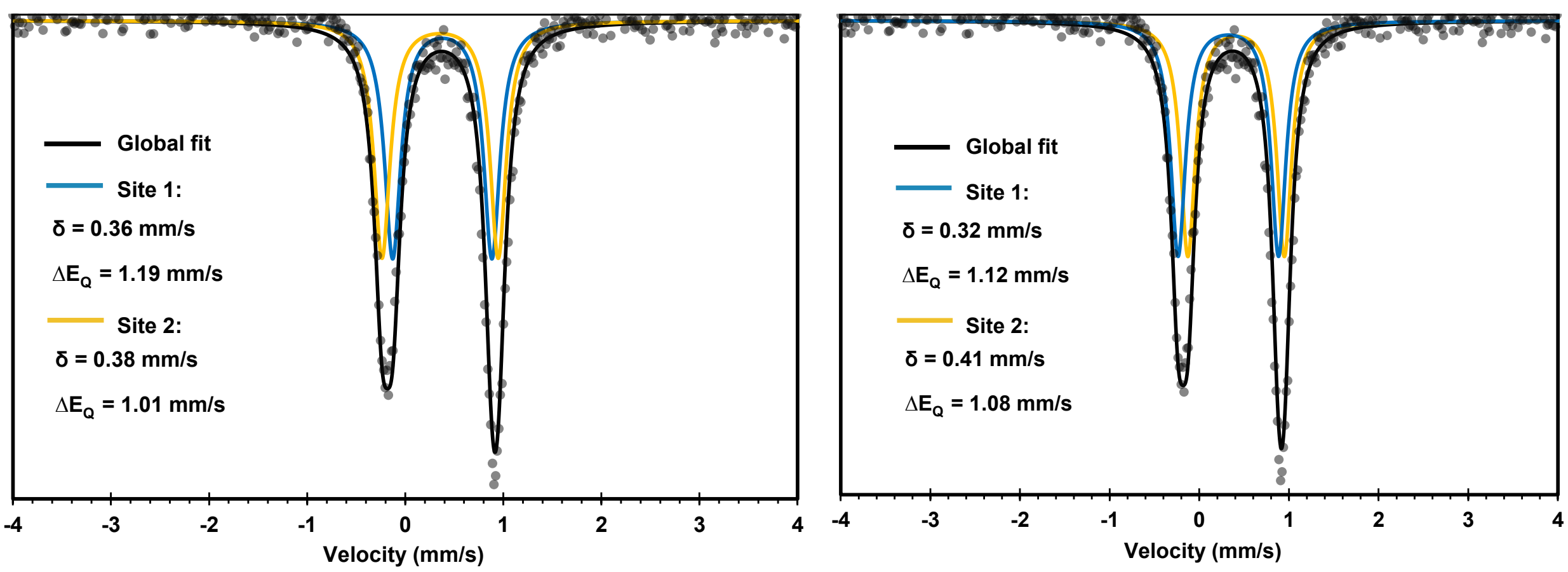

Figure S66. ^{57}Fe Mössbauer spectra of $(NMe_4)_2$$\mathbf{[1^{OH}]}$ (200 K) with two alternate doublet fittings: similar δ (left) or similar ΔE_Q (right).

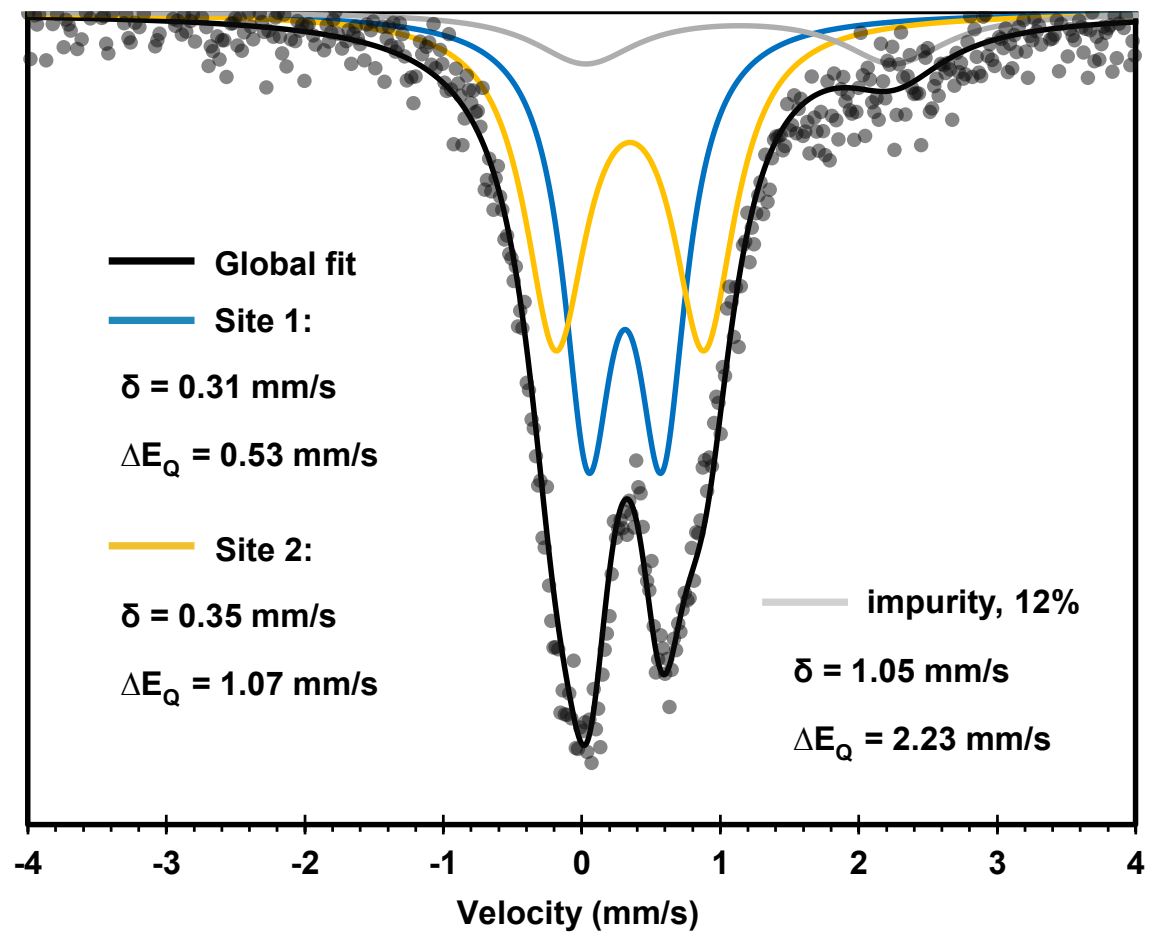

Figure S67. ^{57}Fe Mössbauer spectra of $\mathbf{1^{OH}{\subset}[Zn_4L_6]}(NTf_2)_6$ (200 K) with a single doublet fitting of similar δ. The feature with gray solid line represents an unidentified impurity.

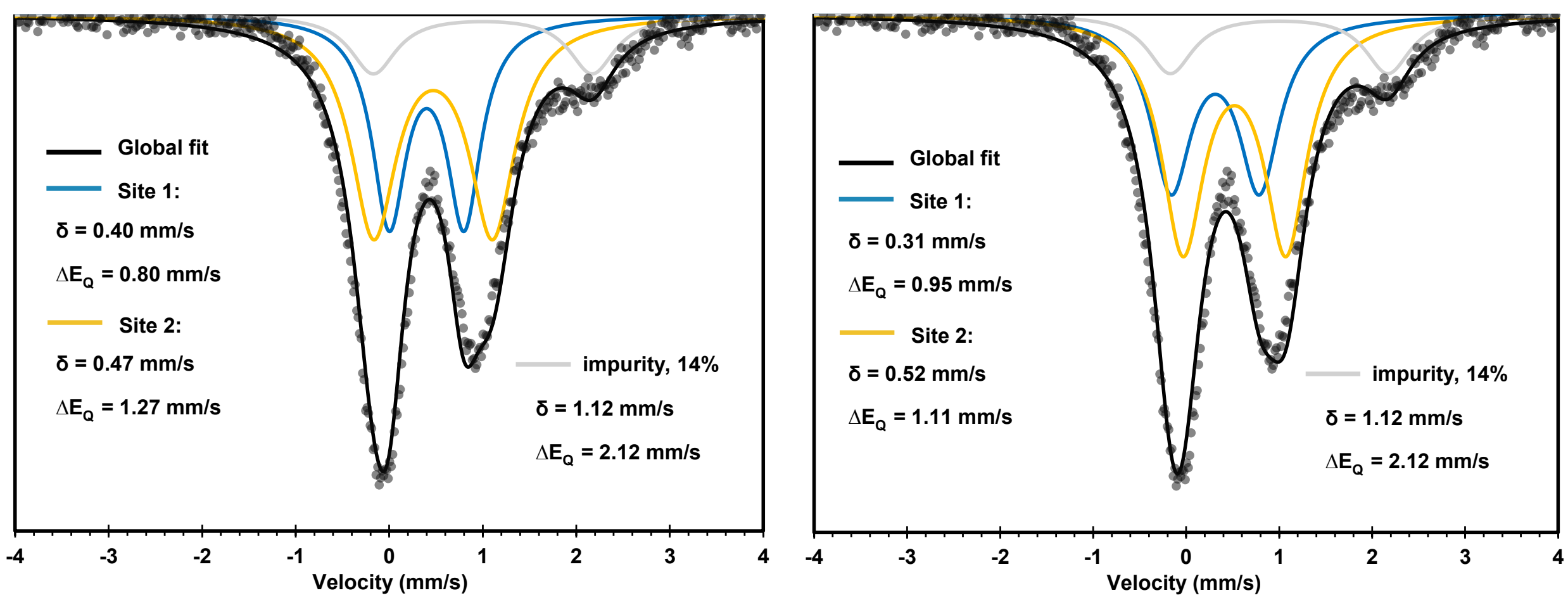

Figure S68. ^{57}Fe Mössbauer spectra of $\mathbf{1^{OH}{\subset}[Zn_4L_6]}$ (200 K) with two alternate doublet fittings: similar δ (left) or similar ΔE$_Q$ (right).

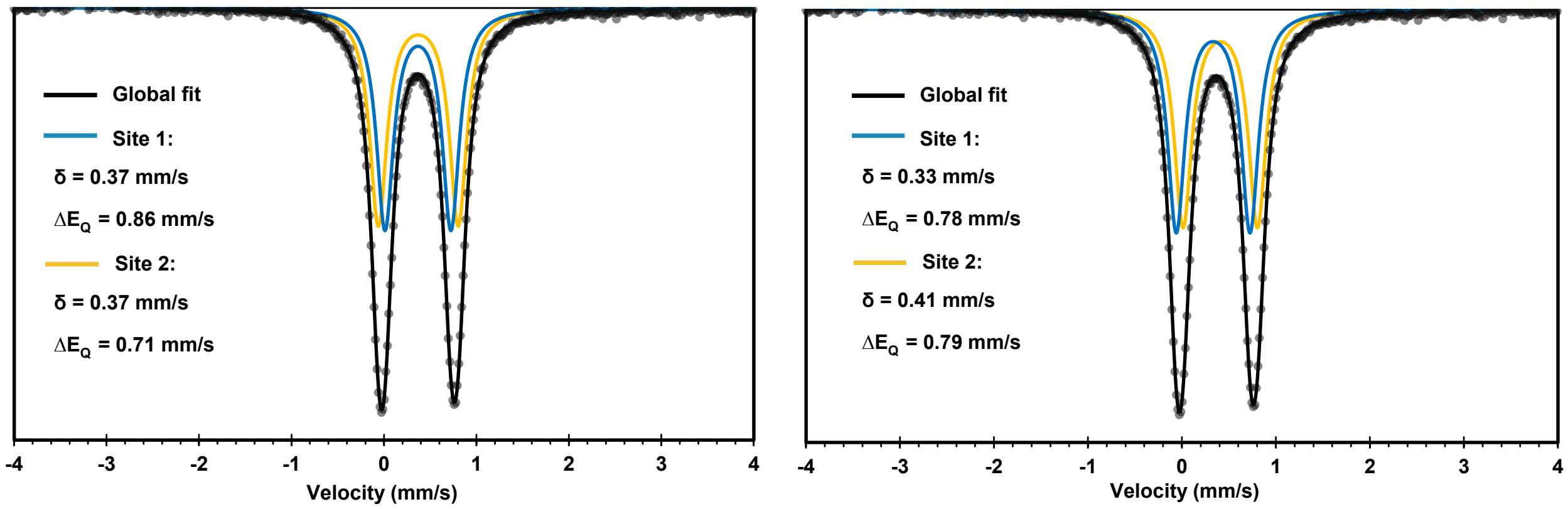

Figure S69. ^{57}Fe Mössbauer spectra of $(NMe_4)_2$**[1^{tBu}]** (200 K) with two alternate doublet fittings: similar δ (left) or similar ΔE_Q (right).

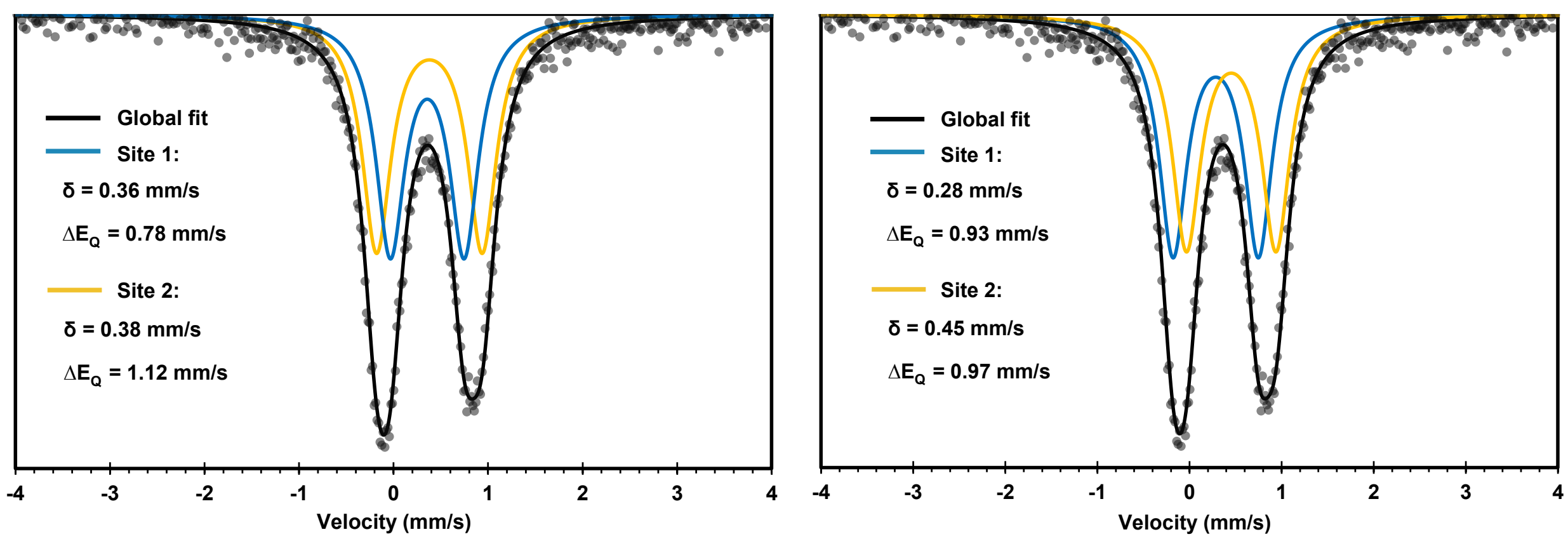

Figure S70. ^{57}Fe Mössbauer spectra of **[Ni$_4$L$_6$][1^{tBu}]**$(NTf_2)_6$ (200 K) with two alternate doublet fittings: similar δ (left) or similar ΔE_Q (right).

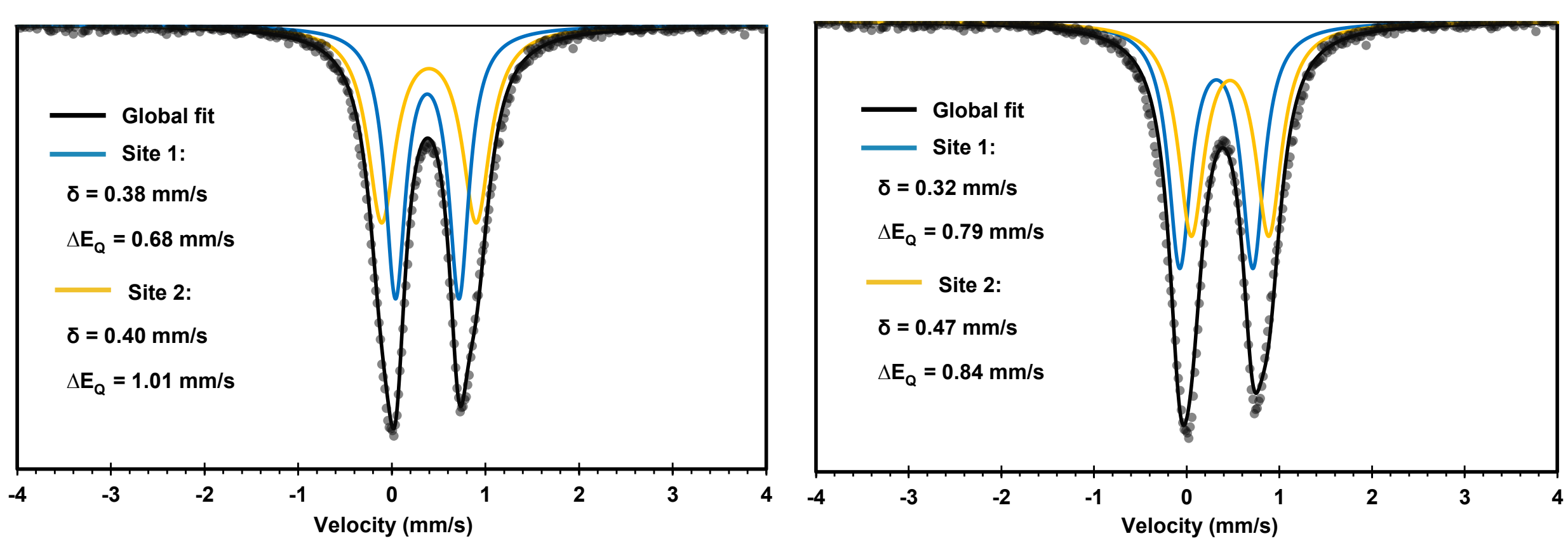

Figure S71. ^{57}Fe Mössbauer spectra of **[Ni$_4$L$_6$][1^{tBu}]** (200 K) with two alternate doublet fittings: similar δ (left) or similar ΔE_Q (right).

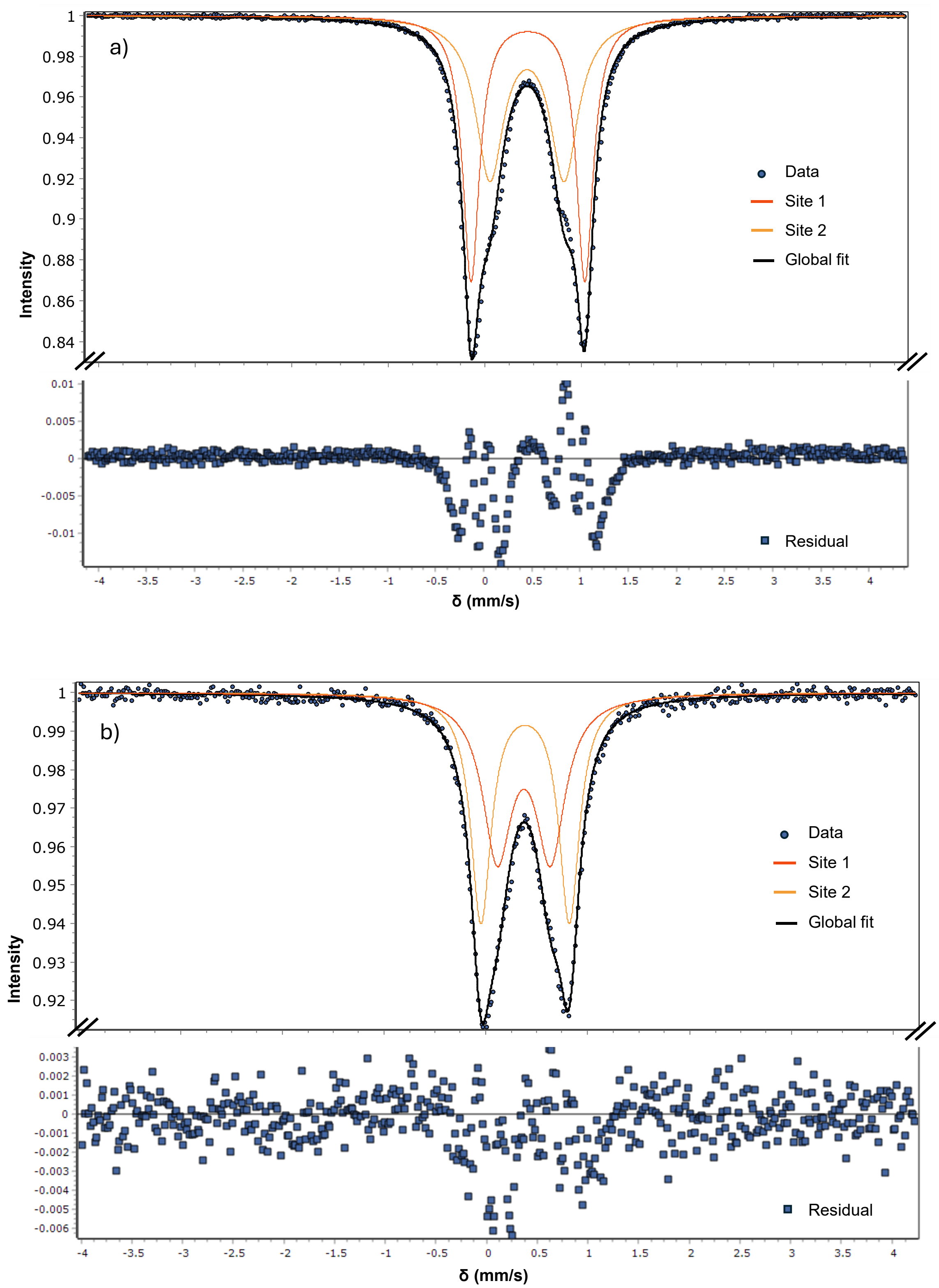

Figure S72. ^{57}Fe Mössbauer spectra of $(NMe_4)_2$**[1^{Ph}]** at a) 70 K and b) 200 K with doublet fittings of similar δ. Residuals (data – global fit) are plotted beneath each spectrum; the goodness-of-fit σ (RMS of residuals) are 3.0×10^{-3} mm/s and 1.47×10^{-3} mm/s, respectively.

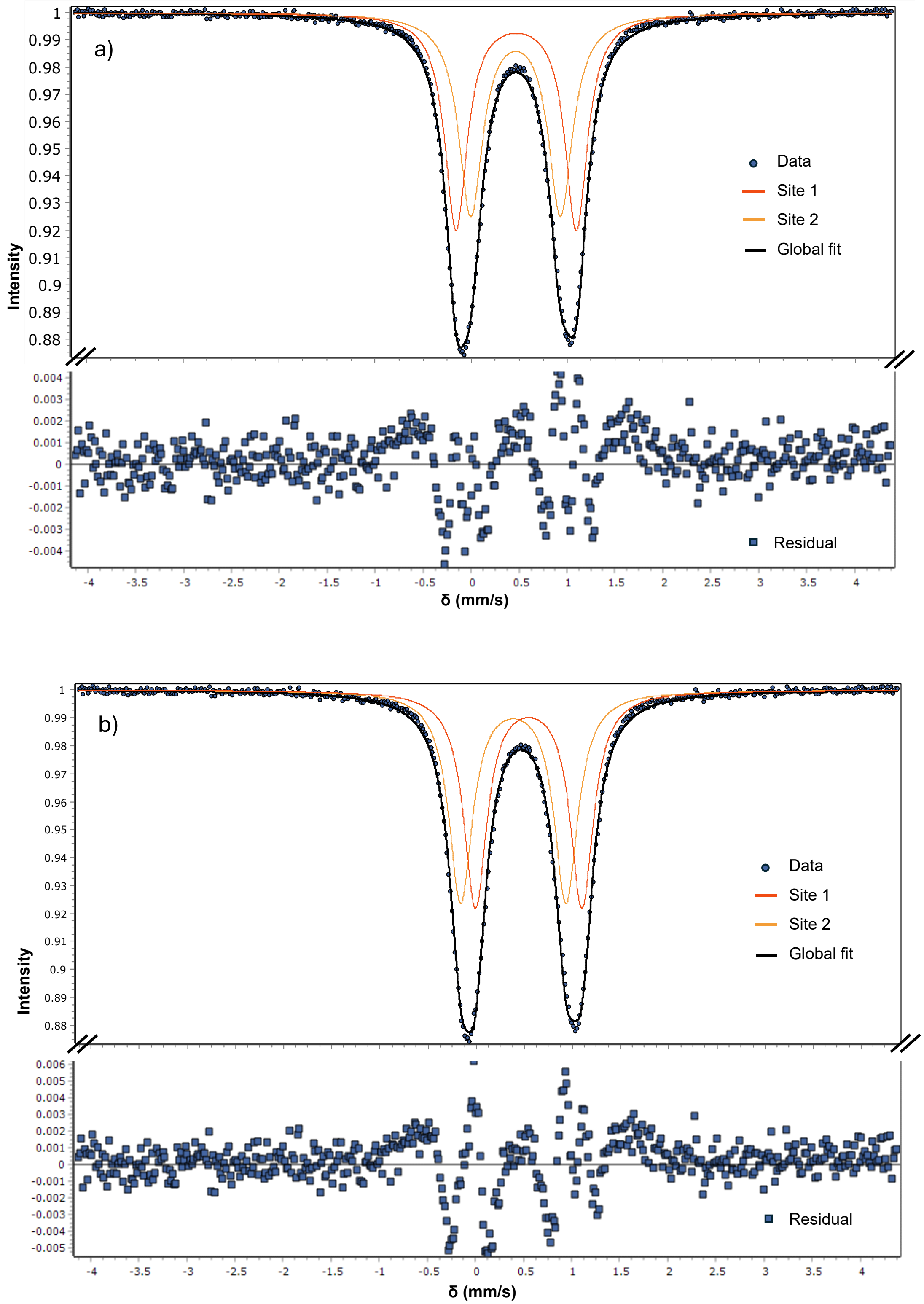

Figure S73. ^{57}Fe Mössbauer spectra of $\mathbf{1^{Ph}{\subset}[Ni_4L_6]}(NTf_2)_6$ (70 K) with two alternate doublet fittings: a) similar δ or b) similar ΔE_Q. Residuals (data – global fit) are plotted beneath each spectrum; the goodness-of-fit σ (RMS of residuals) are 1.29×10^{-3} mm/s and 1.11×10^{-3} mm/s, respectively.

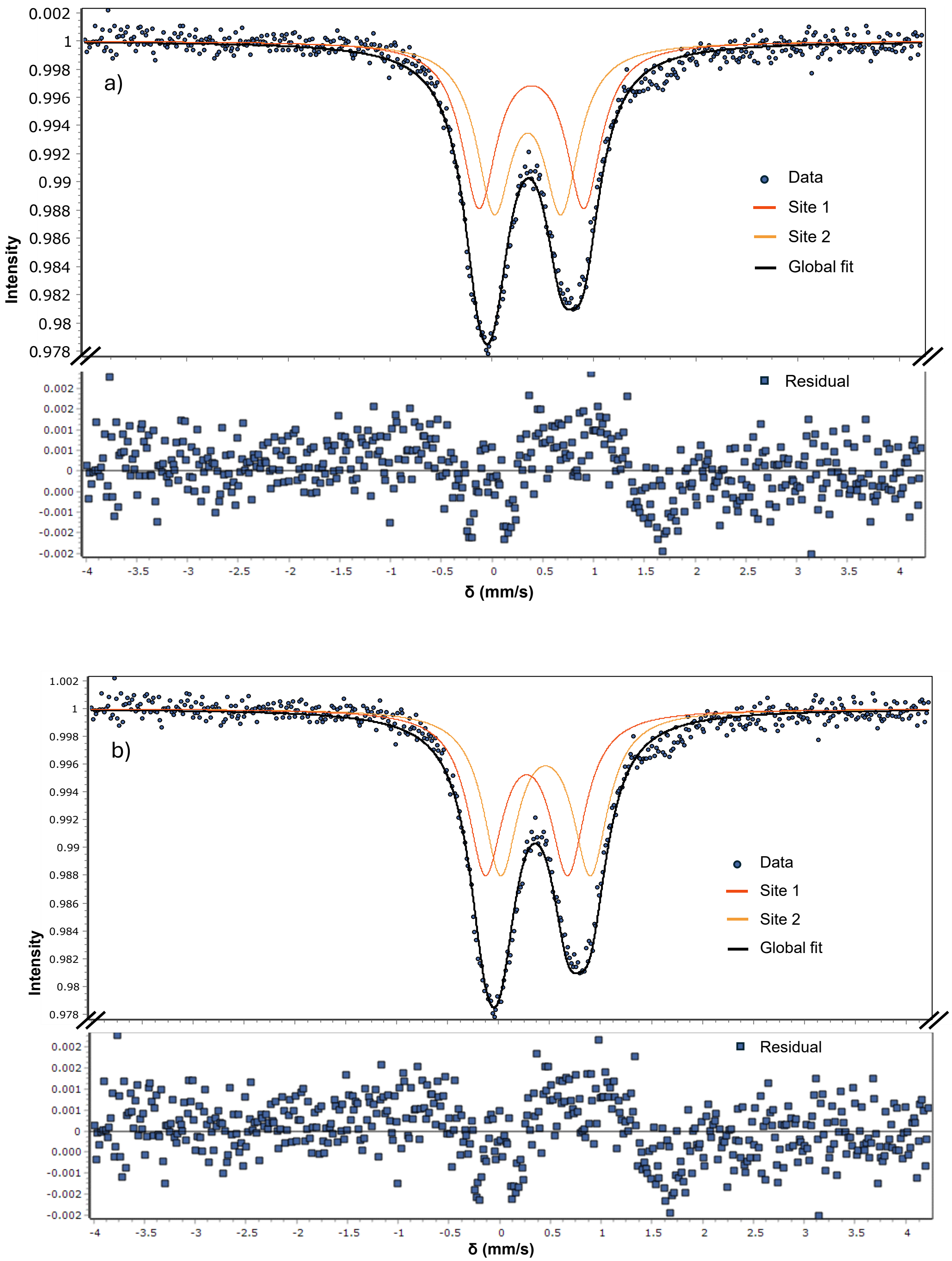

Figure S74. ^{57}Fe Mössbauer spectra of $\mathbf{1^{Ph}{\subset}[Zn_4L_6]}(NTf_2)_6$ (200 K) with two alternate doublet fittings: a) similar δ or b) similar ΔE_Q. Residuals (data – global fit) are plotted beneath each spectrum; the goodness-of-fit σ (RMS of residuals) are 0.69 × 10^{-3} mm/s and 0.69 × 10^{-3} mm/s, respectively.

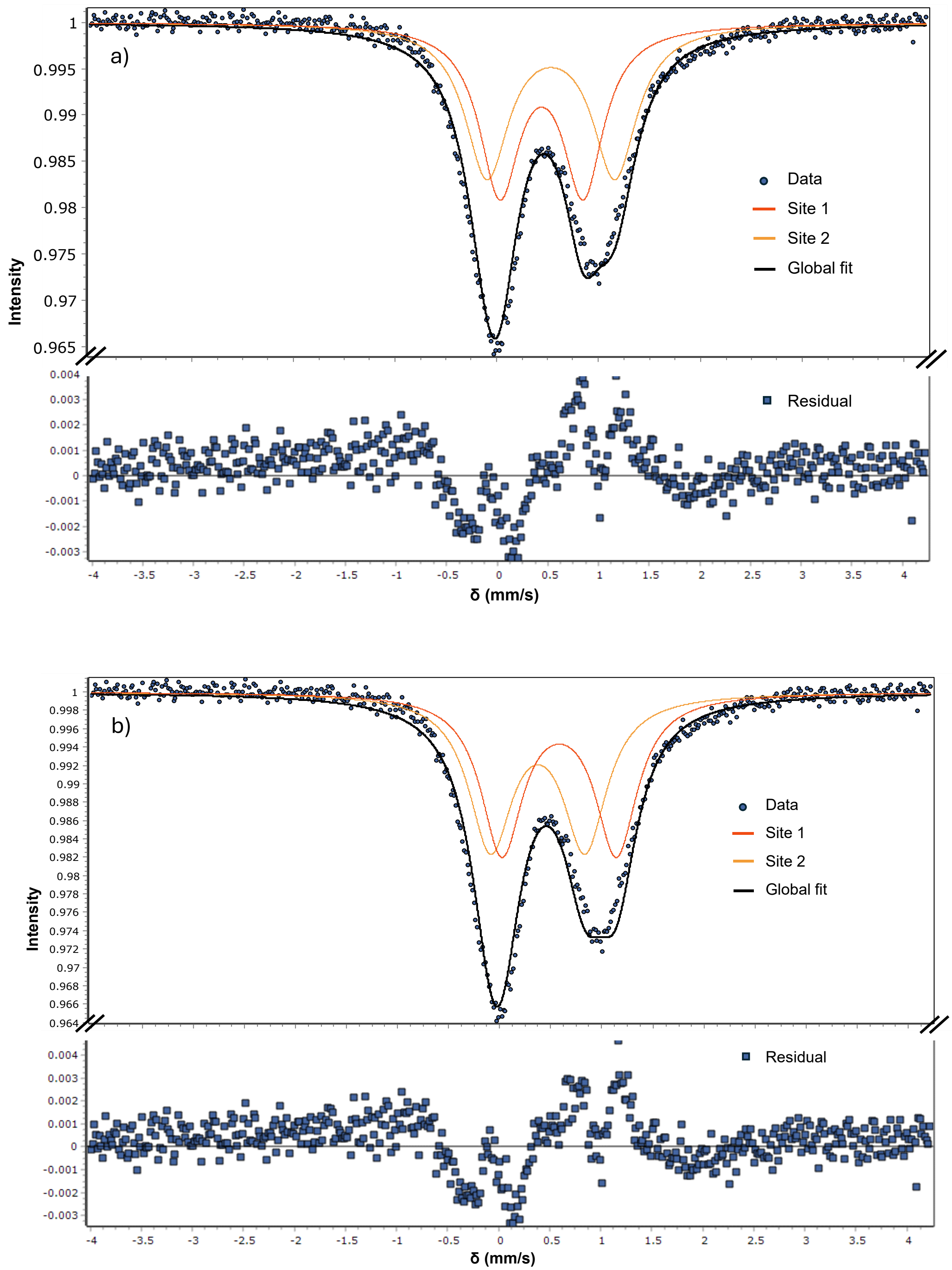

Figure S75. ^{57}Fe Mössbauer spectra of **1^{Ph}⊂[Zn$_4$L$_6$]** (200 K) with two alternate doublet fittings: a) similar δ or b) similar ΔE_Q. Residuals (data – global fit) are plotted beneath each spectrum; the goodness-of-fit σ (RMS of residuals) are 1.11×10^{-3} mm/s and 1.11×10^{-3} mm/s, respectively.

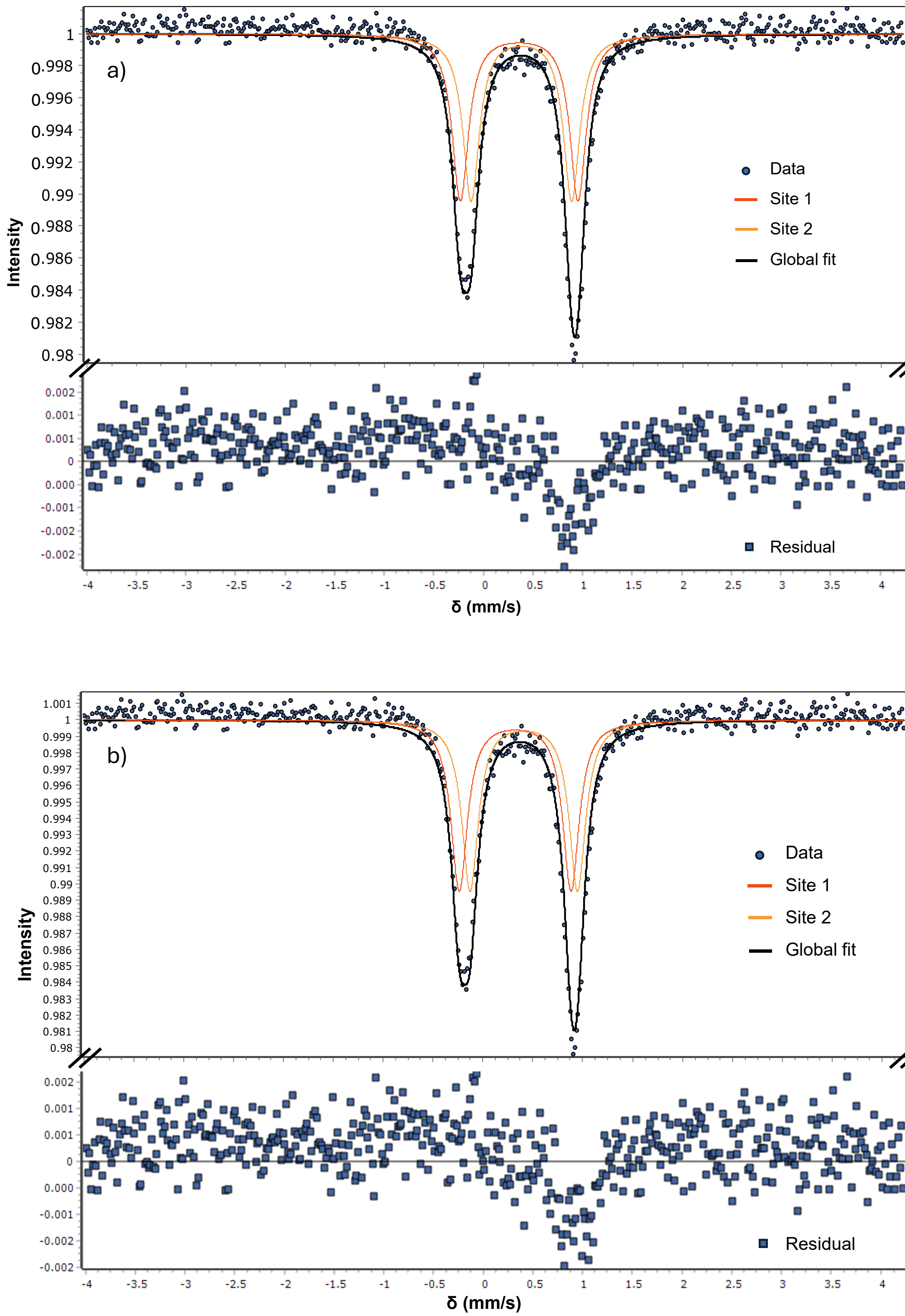

Figure S76. ^{57}Fe Mössbauer spectra of $(NMe_4)_2$[**1^{OH}**] (200 K) with two alternate doublet fittings: a) similar δ or b) similar ΔE_Q. Residuals (data – global fit) are plotted beneath each spectrum; the goodness-of-fit σ (RMS of residuals) are 0.63×10^{-3} mm/s and 0.63×10^{-3} mm/s, respectively.

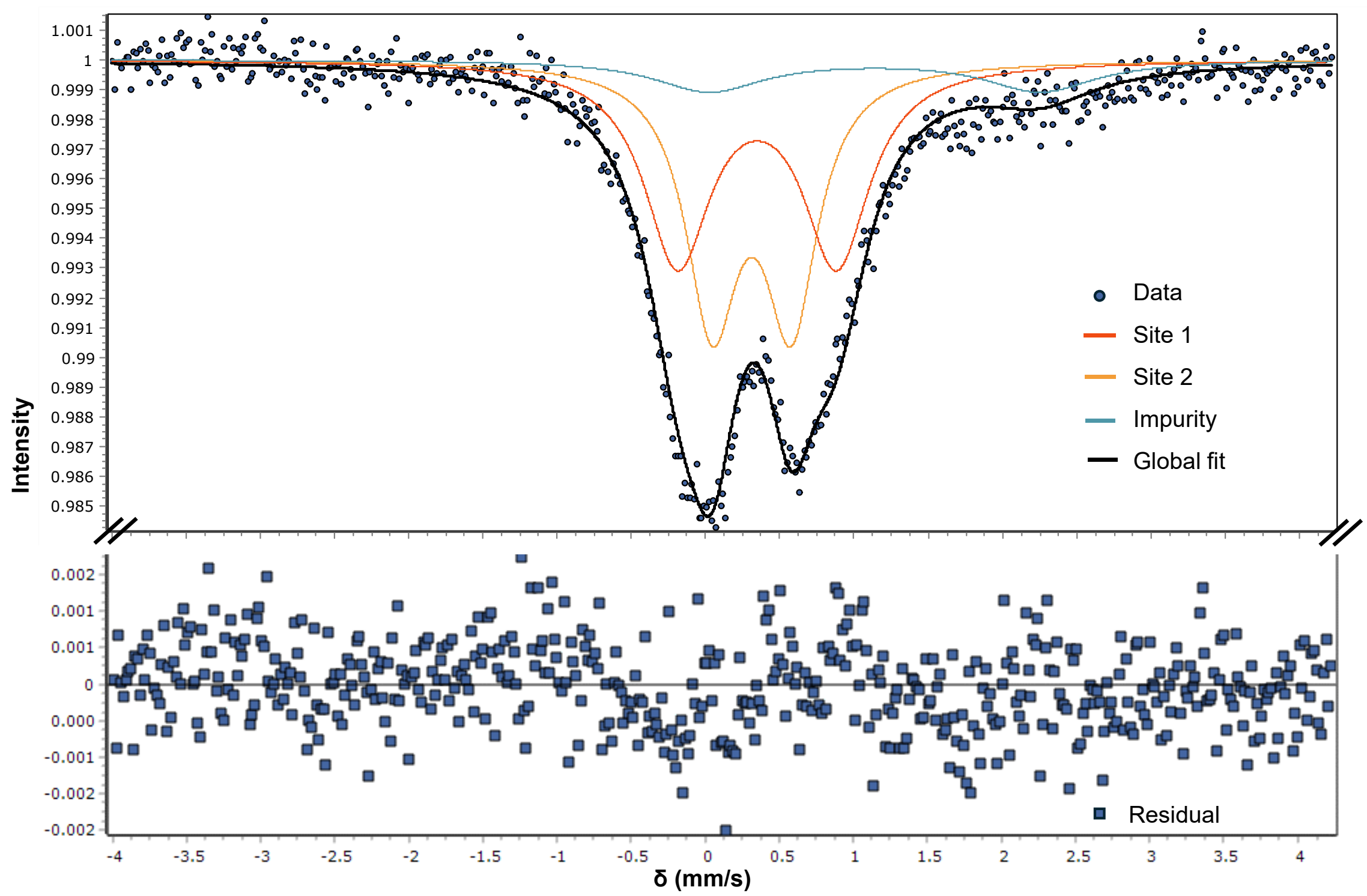

Figure S77. ^{57}Fe Mössbauer spectra of $\mathbf{1^{OH}\subset[Zn_4L_6]}(NTf_2)_6$ (200 K) with one doublet fitting of similar δ. Residual (data – global fit) is plotted beneath the spectrum; the goodness-of-fit σ (RMS of residual) is 0.56×10^{-3} mm/s.

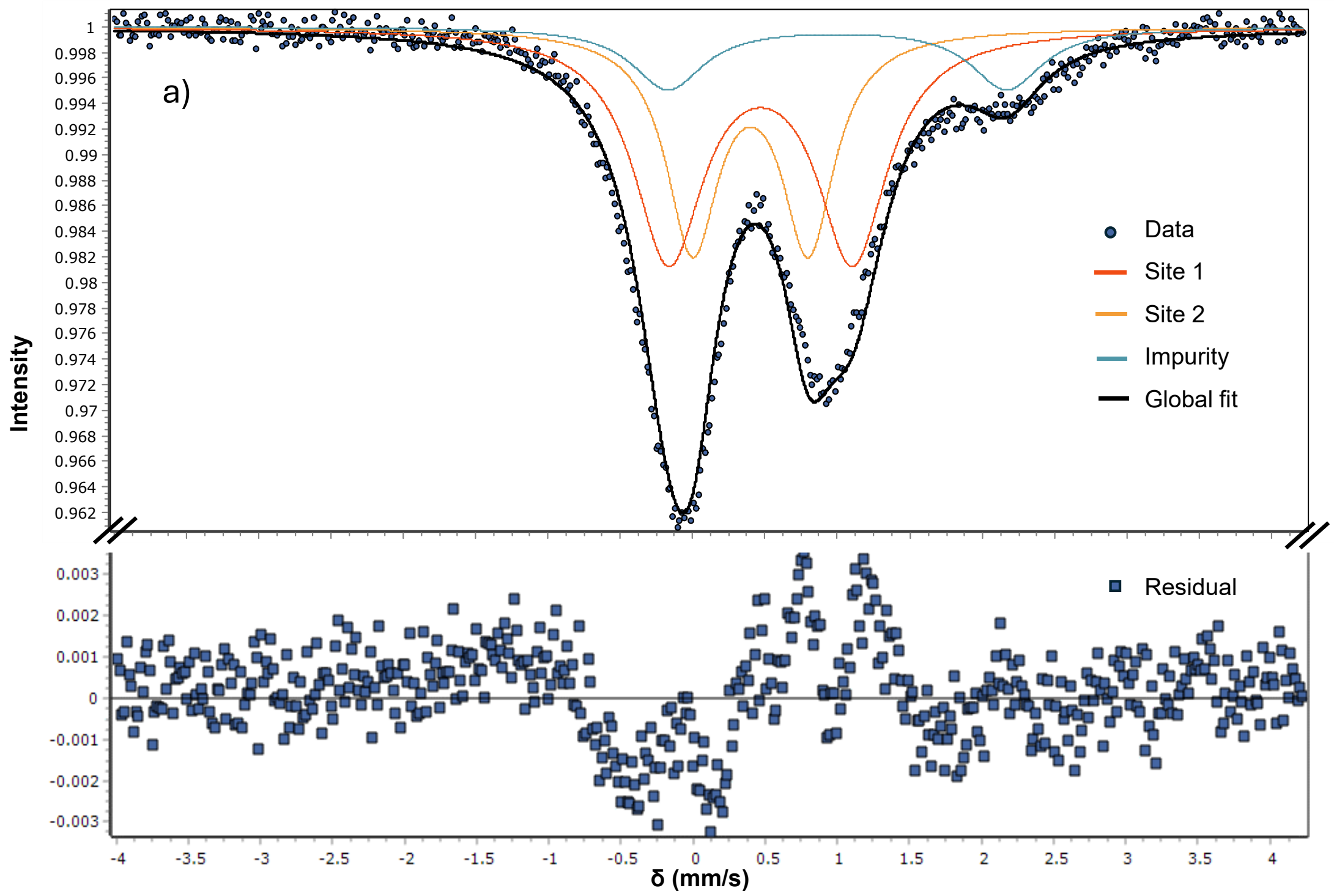

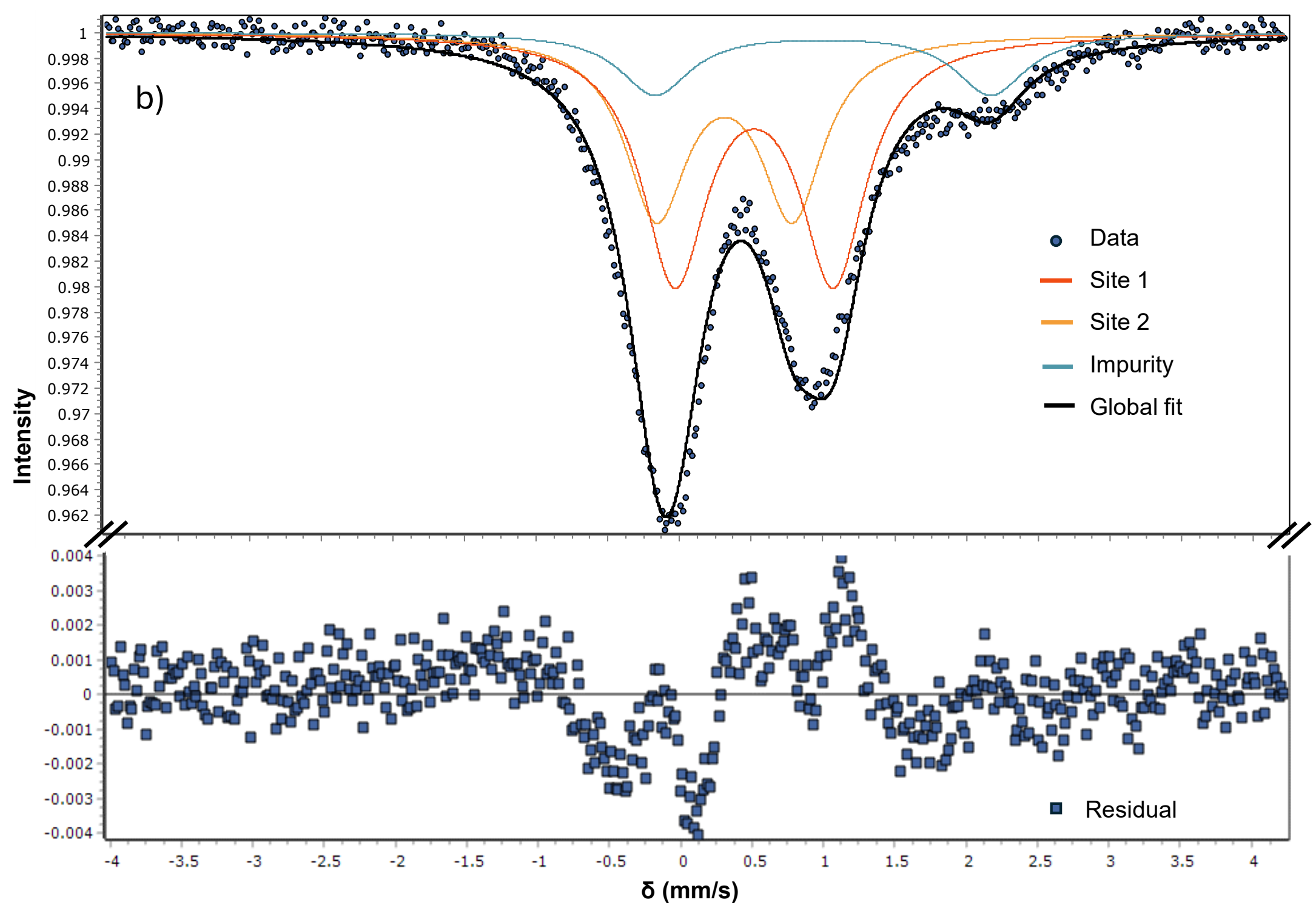

Figure S78. ^{57}Fe Mössbauer spectra of $\mathbf{1^{OH}{\subset}[Zn_4L_6]}$ (200 K) with two alternate doublet fittings: a) similar δ or b) similar ΔE_Q. Residuals (data – global fit) are plotted beneath each spectrum; the goodness-of-fit σ (RMS of residuals) are 1.13×10^{-3} mm/s and 1.19×10^{-3} mm/s, respectively.

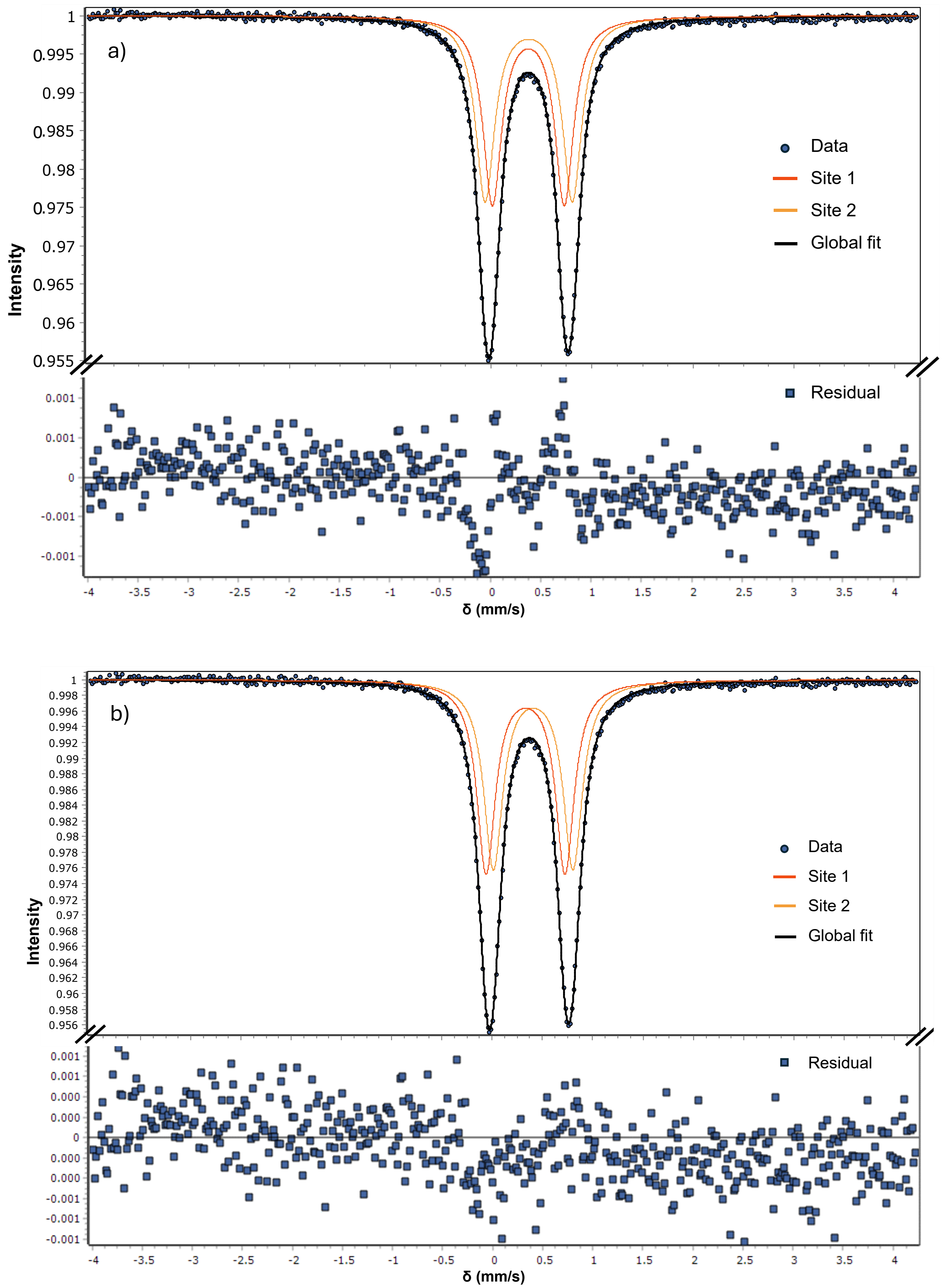

Figure S79. ^{57}Fe Mössbauer spectra of $(NMe_4)_2$**[1^{tBu}]** (200 K) with two alternate doublet fittings: a) similar δ or b) similar ΔE_Q. Residuals (data – global fit) are plotted beneath each spectrum; the goodness-of-fit σ (RMS of residuals) are 0.37×10^{-3} mm/s and 0.33×10^{-3} mm/s, respectively.

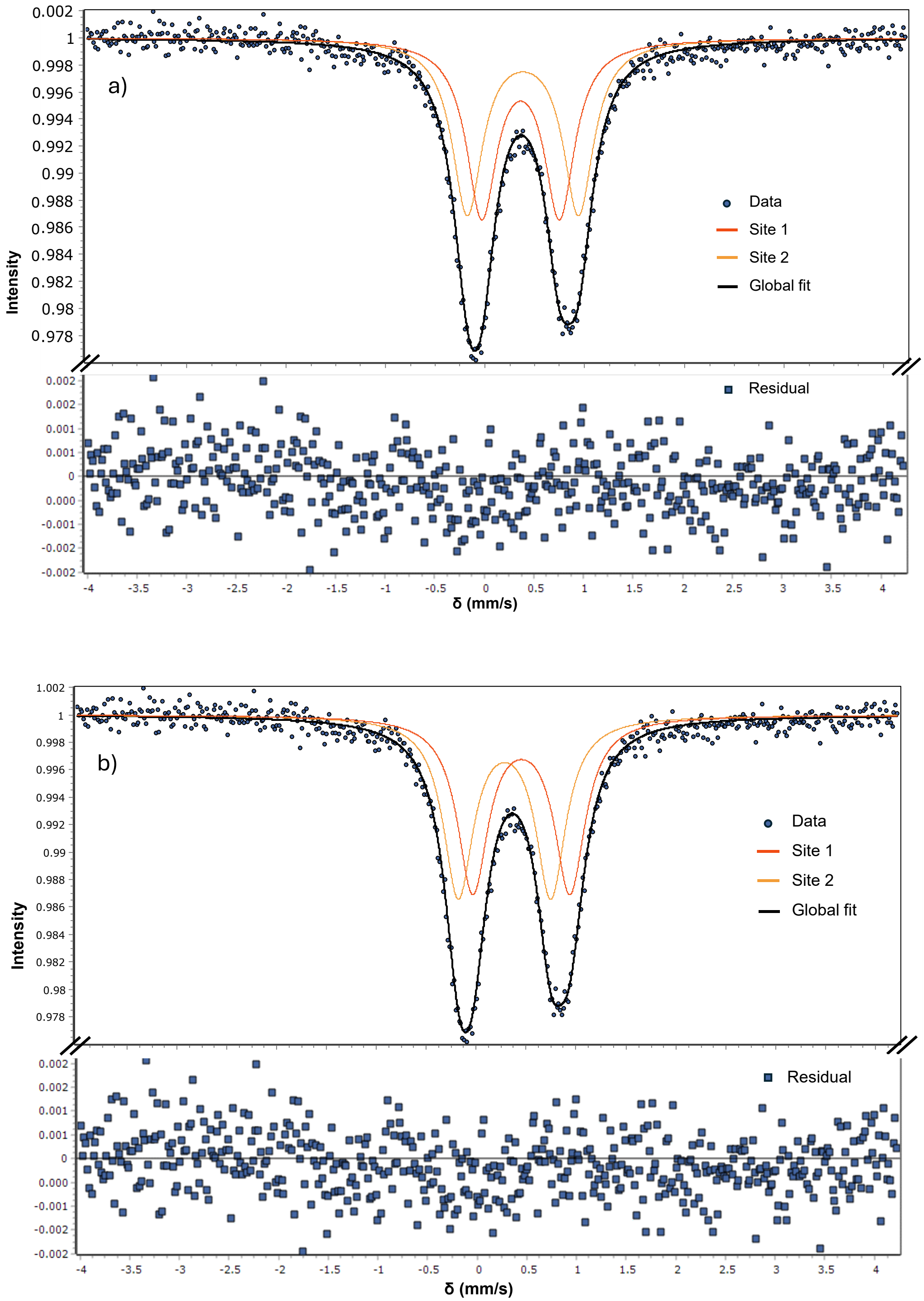

Figure S80. ^{57}Fe Mössbauer spectra of **$[Ni_4L_6][1^{tBu}]$**$(NTf_2)_6$ (200 K) with two alternate doublet fittings: a) similar δ or b) similar ΔE_Q. Residuals (data – global fit) are plotted beneath each spectrum; the goodness-of-fit σ (RMS of residuals) are 0.65×10^{-3} mm/s and 0.64×10^{-3} mm/s, respectively.

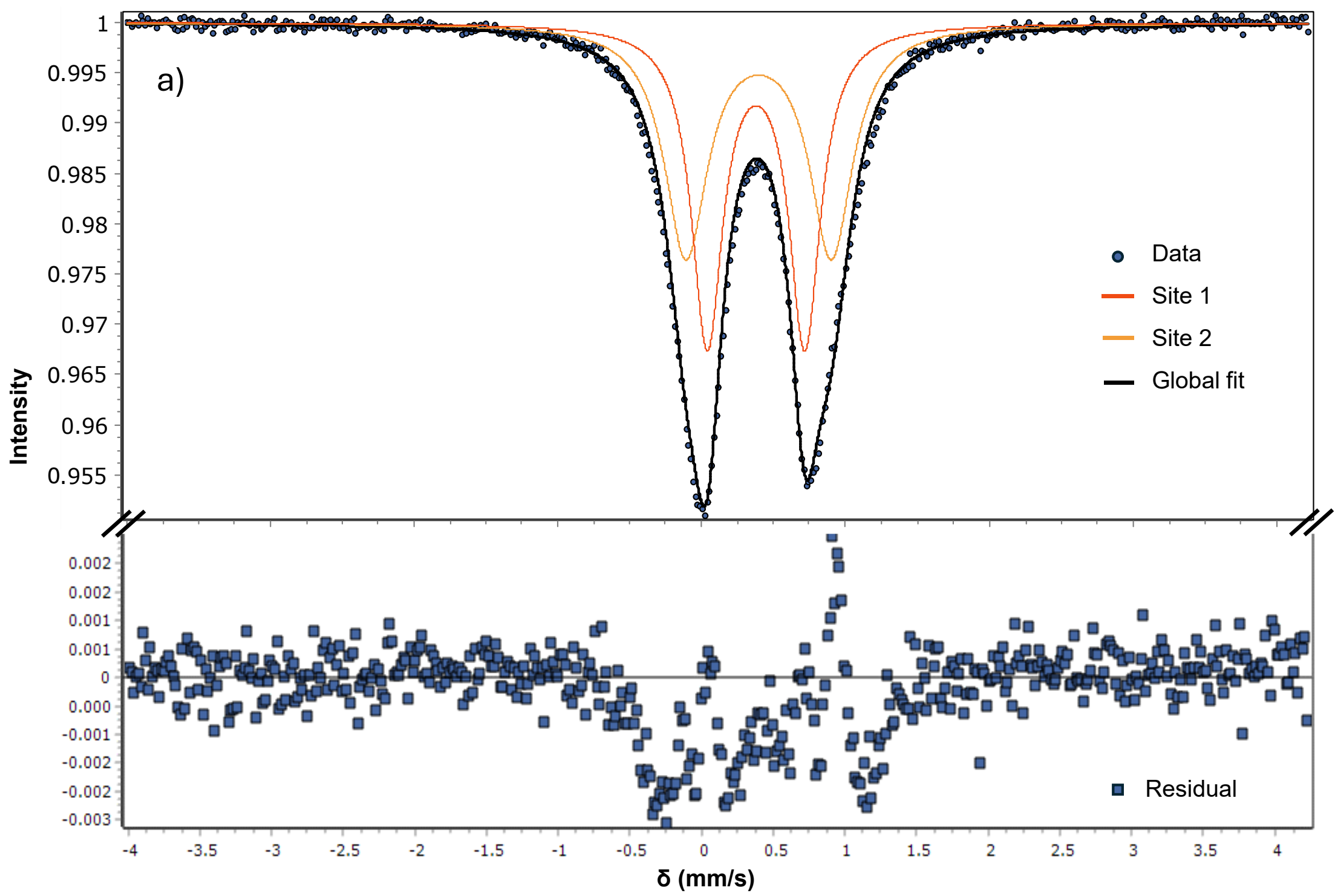

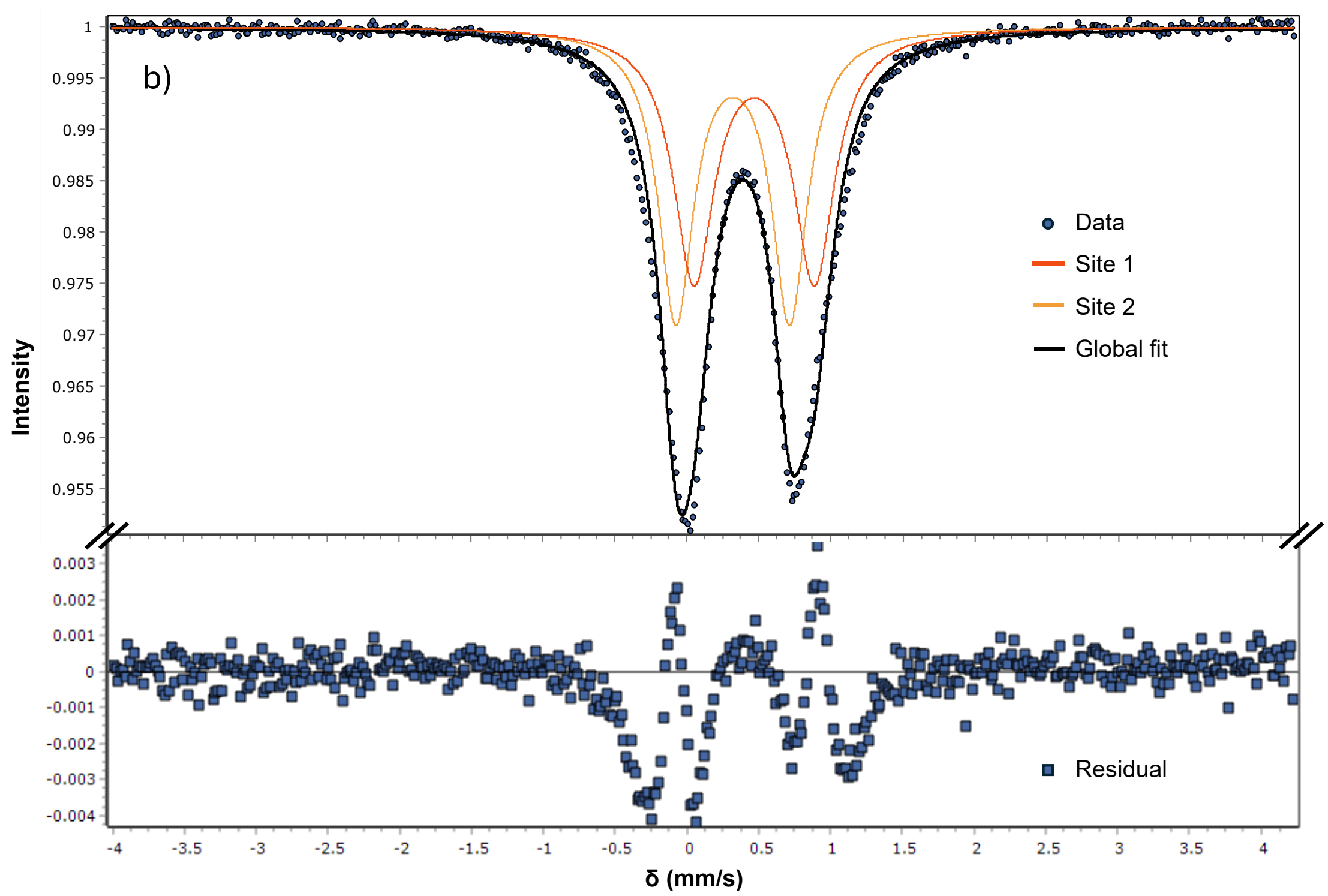

Figure S81. ^{57}Fe Mössbauer spectra of **[Ni$_4$L$_6$][1^{tBu}]** (200 K) with two alternate doublet fittings: a) similar δ or b) similar ΔE_Q. Residuals (data – global fit) are plotted beneath each spectrum; the goodness-of-fit σ (RMS of residuals) are 0.74×10^{-3} mm/s and 1.00×10^{-3} mm/s, respectively.

S9. X-RAY PHOTOELECTRON SPECTROSCOPY

X-ray photoelectron spectroscopy (XPS) measurements were performed using a PHI 5000 VersaProbe III photoelectron spectrometer equipped with a monochromatic Al Kα X-ray source (hν = 1486.6 eV). The X-ray beam was operated at a spot size of 200 µm for survey scans and 100 µm for high-resolution scans. The power settings were 50 W for survey scans and 15 W for high-resolution scans, with an accelerating voltage of 15 kV. Samples were prepared according to the procedure outlined in Section S3 and S8 in an N_2-filled glovebox and loaded into a sealed container to prevent air exposure during transport to the instrument. The pressure in the analysis chamber was maintained below 5.0×10^{-4} Pa throughout the measurements. High-resolution spectra were collected for Fe and S regions using a pass energy of 55 eV, a step size of 0.1 eV, and a dwell time of 20 ms. Charge neutralization was applied using a dual-beam charge compensation system. Spectra were calibrated to the Au $4f_{7/2}$ reference peak at 84.0 eV. Data processing and peak fitting were conducted using the MultiPak software package. Peak shapes in the high-resolution spectra were modeled using 80% Gaussian and 20% Lorentzian functions, and the area ratio of $2p_{3/2}$ to $2p_{1/2}$ peaks was constrained to 2:1. Satellite peaks were included in the Fe 2*p* region to improve the fit quality and assess electronic relaxation (see below). Background correction was performed to achieve the lowest possible χ^2 value, which represents the goodness-of-fit parameter, with lower values indicating a better match between the experimental data and the fitted model.

Determination of E_{rlx}. The valence band configuration interaction (VBCI) model developed by Kennepohl and co-workers provides a framework for estimating the covalency of transition metal complexes via analysis of their XPS spectra.[22,23] Core-to-vacuum Fe 2*p* XPS spectra commonly exhibit an intense main peak (M) and a satellite feature (S) located at higher binding energy. The satellite features result from shake-up ionization processes in which the core-to-vacuum transition of a 2*p* electron is coupled with a valence electron excitation. These processes are formally two-electron transitions and thus forbidden; however, the allowedness increases with the electronic relaxation energy (E_{rlx}), which reflects the stabilization energy gained through subsequent electron redistribution from the ligands to the metal center. The extent of ligand-to-metal electron redistribution is positively correlated with covalency and thus can be used to get a relative sense of covalency in structurally related complexes. According to the VBCI model, E_{rlx} can be quantified using the following expression:

$$E_{rlx} = W_f \left(\frac{I_S/I_M}{1+(I_S/I_M)}\right) \quad \textbf{(Equation S3)}$$

where E_{rlx} is the electronic relaxation energy in eV, W_f is the energy difference between the main $2p_{3/2}$ peak and its corresponding satellite, and I_S/I_M is the area ratio of the satellite peak (I_S) to the main peak (I_M), extracted from the simulated XPS spectra. This approach allows for a semi-quantitative assessment of metal-ligand covalency, as higher E_{rlx} values indicate a greater extent of ligand-to-metal redistribution during relaxation and therefore greater electronic delocalization (covalency).

Table S4. XPS parameters for Fe 2*p* transitions of the clusters $(NMe_4)_2$**[1^R]** and host-guest complexes **1^R⊂[Zn$_4$L$_6$]**^{x} and ion pairs **[Ni$_4$L$_6$][1^{tBu}]**^{x} (x = +6, 0) taken at RT with pressure < 5.0 × 10^{-4} Pa. Energies are given in eV and referenced to the Au $4f_{7/2}$ peak of gold foil.

Complex	2$p_{3/2}$ BE (eV)^a	Satellite^b (eV)	I_S/I_P	W_f (eV)	E_{rlx} (eV)	2$p_{1/2}$ BE (eV)^a	χ^{2c}
$(NMe_4)_2$**[1^{Ph}]**	708.84	715.14	0.14	6.3	0.77	722.14	1.98
**1^{Ph}⊂[Zn$_4$NDI$_6$]**$(NTf_2)_6$	709.91	715.91	0.48	6.0	1.95	722.01	2.15
**1^{Ph}⊂[Zn$_4$NDI$_6$]**	709.69	716.49	0.36	6.8	1.80	722.29	1.27
$(NMe_4)_2$**[1^{OH}]**	708.58	714.28	0.19	5.7	0.91	721.88	1.68
**1^{OH}⊂[Zn$_4$NDI$_6$]**$(NTf_2)_6$	709.11	715.91	0.52	6.8	2.33	722.01	1.62
**1^{OH}⊂[Zn$_4$NDI$_6$]**	709.52	715.92	0.42	6.4	1.89	722.52	1.21
$(NMe_4)_2$**[1^{tBu}]**	709.46	714.66	0.25	5.2	1.04	722.86	2.90
**[Ni$_4$NDI$_6$][1^{tBu}]**$(NTf_2)_6$	710.00	716.01	0.58	6.0	2.20	723.10	1.62
**[Ni$_4$NDI$_6$][1^{tBu}]**	708.89	716.19	0.37	7.3	1.97	721.59	1.55

^{a}Binding energy = BE. ^{b}Energy of the shake-up satellite transition associated with the Fe 2$p_{3/2}$ core to valence transition. ^{c}goodness-of-fit parameter.

Table S5. XPS parameters for S 2*p* transitions of the clusters $(NMe_4)_2$**[1^R]** and host-guest complexes **1^R⊂[Zn$_4$L$_6$]**^{x} and ion pairs **[Ni$_4$L$_6$][1^{tBu}]**^{x} (x = +6, 0) taken at RT with pressure < 5.0 × 10^{-4} Pa. Energies are given in eV and referenced to the Au $4f_{7/2}$ peak of gold foil.

Complex	2$p_{3/2}$ BE (eV)^a	2$p_{1/2}$ BE (eV)^a	2$p_{3/2}$ Area^b [1^R]$^{2-}$:SO_2	Calculated^c [1^R]$^{2-}$:[M_4L_6]$^{8+}$	χ^{2c}
$(NMe_4)_2$**[1^{Ph}]**	162.23	163.44	-	-	1.86
**1^{Ph}⊂[Zn$_4$L$_6$]**$(NTf_2)_6$	162.11	163.31	2.3 : 3	1.2 : 1	3.90
**1^{Ph}⊂[Zn$_4$L$_6$]**	162.09	163.29	-	-	1.85
$(NMe_4)_2$**[1^{OH}]**	161.88	163.18	-	-	1.02
**1^{OH}⊂[Zn$_4$L$_6$]**$(NTf_2)_6$	161.77	162.91	3.0 : 3	1.5 : 1	2.10
**1^{OH}⊂[Zn$_4$L$_6$]wh**	161.82	163.12	-	-	1.73
$(NMe_4)_2$**[1^{tBu}]**	161.76	163.06	-	-	1.79
**[Ni$_4$L$_6$][1^{tBu}]**$(NTf_2)_6$	162.30	163.50	2.3 : 3	1.2 : 1	3.88
**[Ni$_4$L$_6$][1^{tBu}]**	161.99	163.29	-	-	1.52

^{a}Binding energy = BE. ^{b}Relative integrated areas of 2$p_{3/2}$ peaks assigned to encapsulated **[1^R]**$^{2-}$ and triflimide groups. The ideal ratio based on a 1:1 host-guest and ion pair stoichiometry is 2:3 (one cluster containing 8 sulfur atoms and 6 triflimide groups containing 2 sulfur atoms each). ^{c}Calculated ratio of **[1^R]**$^{2-}$ to cage as judged by the S 2$p_{3/2}$ peak integrations for the **[1^R]**$^{2-}$ and triflimide peaks. ^{c}goodness-of-fit parameter.

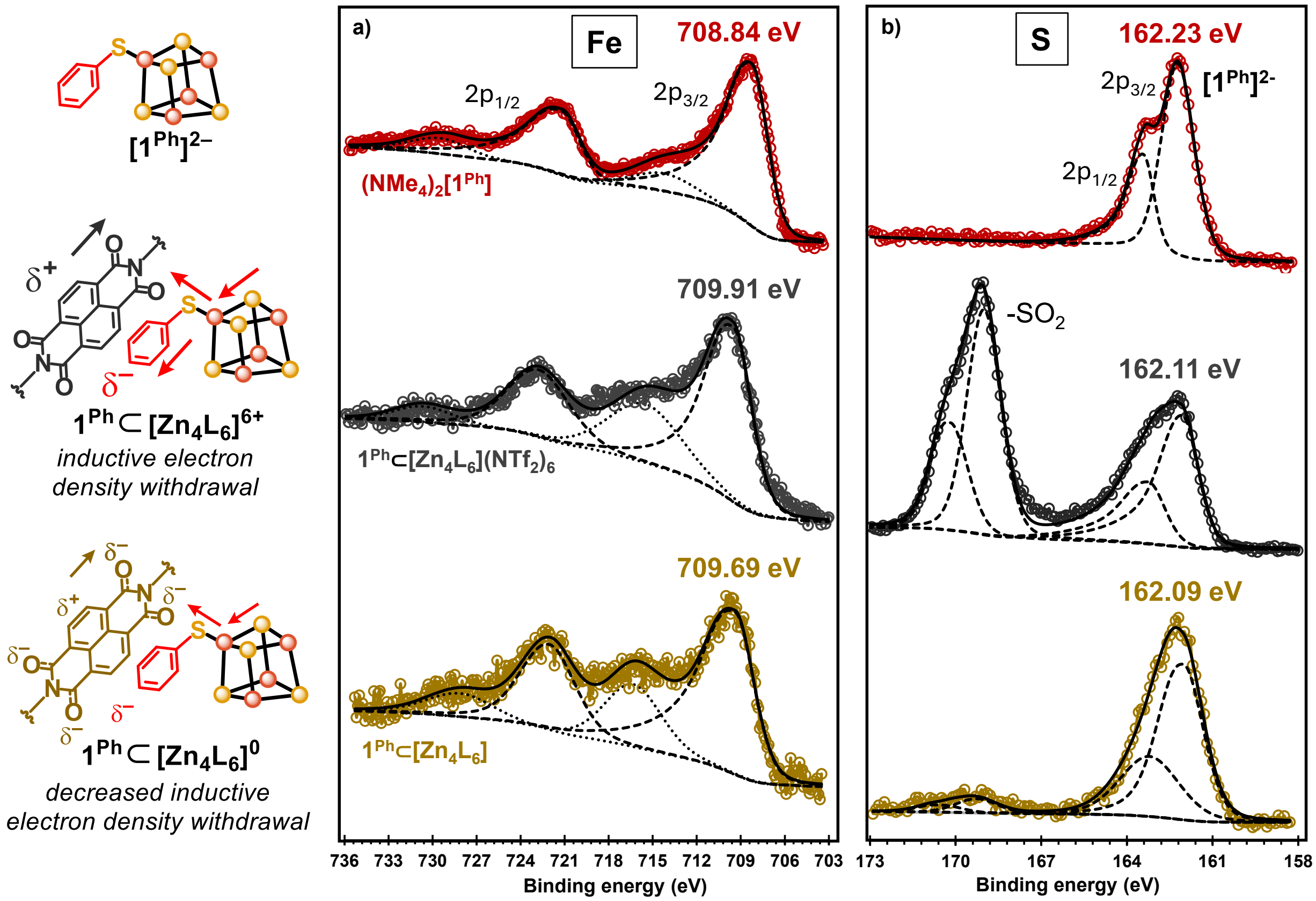

Figure S82. Experimental (colored dots) and simulated (black lines) high-resolution XPS spectra of $(NMe_4)_2$**[1^Ph]**, **1^{Ph}⊂[Zn_4L_6]**$(NTf_2)_6$, and **1^{Ph}⊂[Zn_4L_6]**: (a) Fe 2*p* region, with satellite peaks highlighted in black dotted lines; and (b) S 2*p* region (including cluster and -SO_2 groups). The fittings for the $2p_{1/2}$ and $2p_{3/2}$ peaks are shown with black dashed lines.

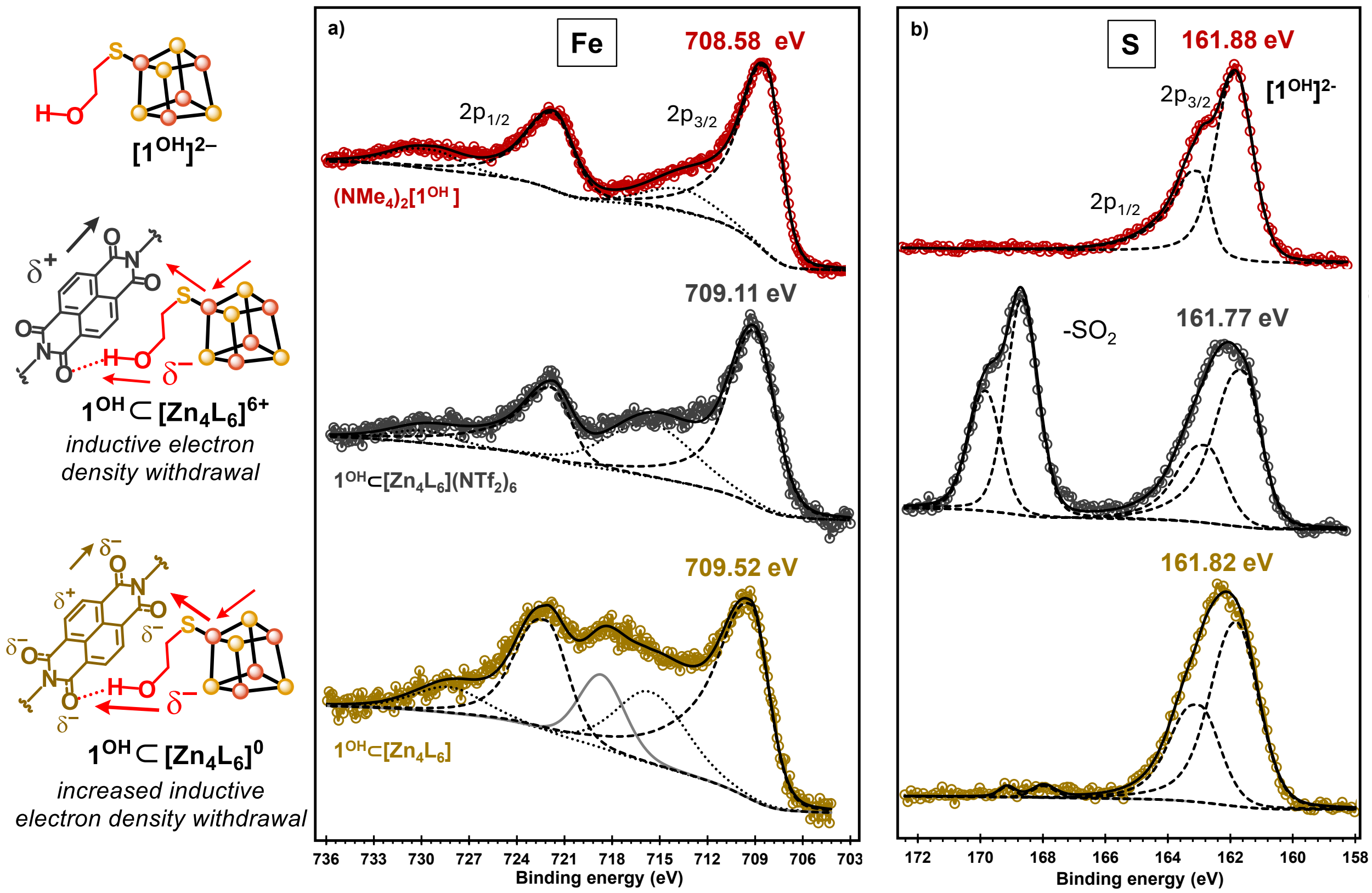

Figure S83. Experimental (colored dots) and simulated (black lines) high-resolution XPS spectra of $(NMe_4)_2$**[1^{OH}]**, **1^{OH}⊂[Zn$_4$L$_6$]**$(NTf_2)_6$, and **1^{OH}⊂[Zn$_4$L$_6$]**: (a) Fe 2*p* region, with satellite peaks highlighted in black dotted lines, Co LMM Auger peak is highlighted in gray; and (b) S 2*p* region (including cluster and $-SO_2$ groups). The fittings for the $2p_{1/2}$ and $2p_{3/2}$ peaks are shown with black dashed lines.

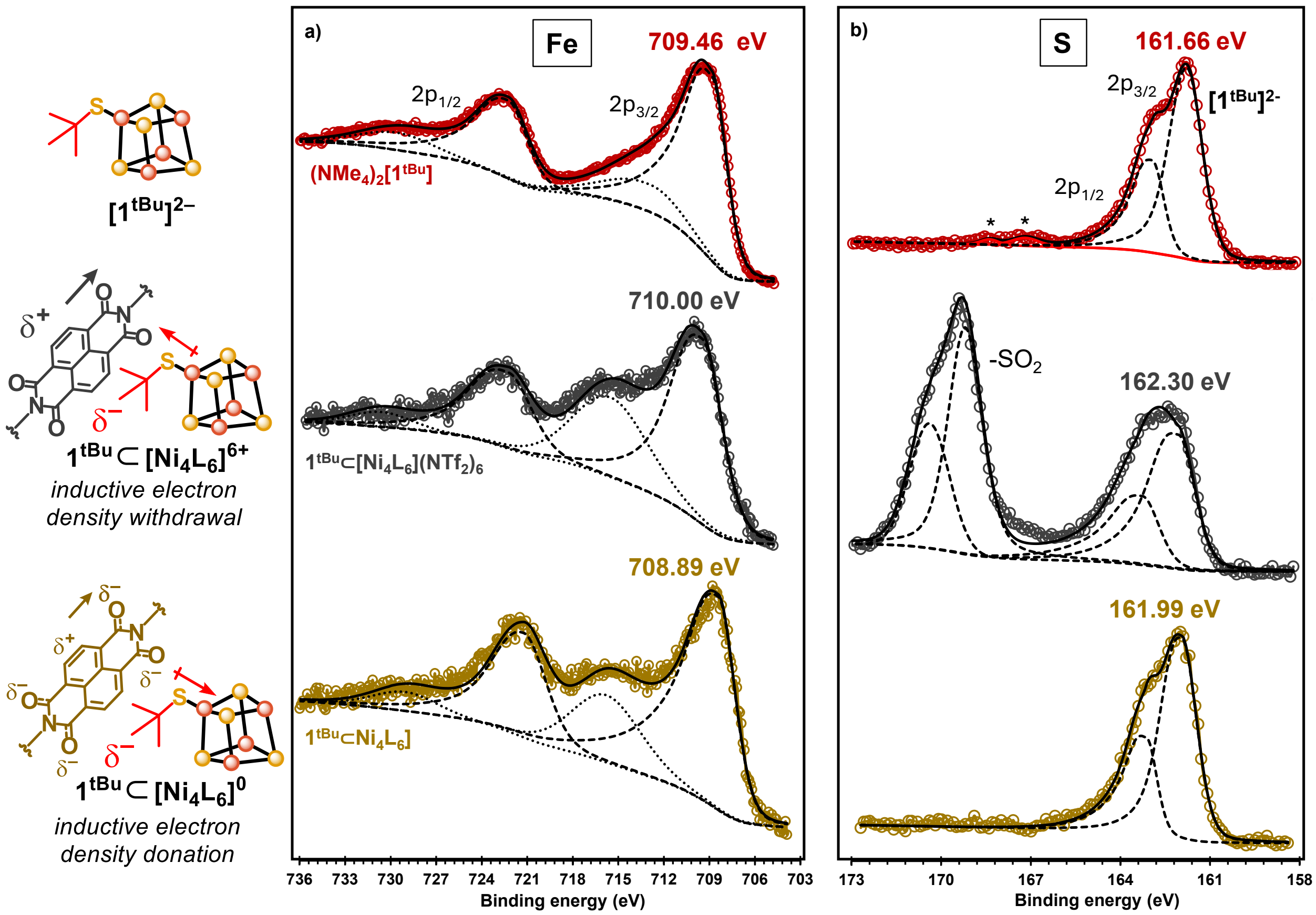

Figure S84. Experimental (colored dots) and simulated (black lines) high-resolution XPS spectra of $(NMe_4)_2$**[1^{tBu}]**, **[Ni$_4$L$_6$][1^{tBu}]**$(NTf_2)_6$, and **[Ni$_4$L$_6$][1^{tBu}]**: (a) Fe 2*p* region, with satellite peaks highlighted in black dotted lines; and(b) S 2*p* region (including cluster and $-SO_2$ groups). The fittings for the $2p_{1/2}$ and $2p_{3/2}$ peaks are shown with black dashed lines. *unidentified impurities.

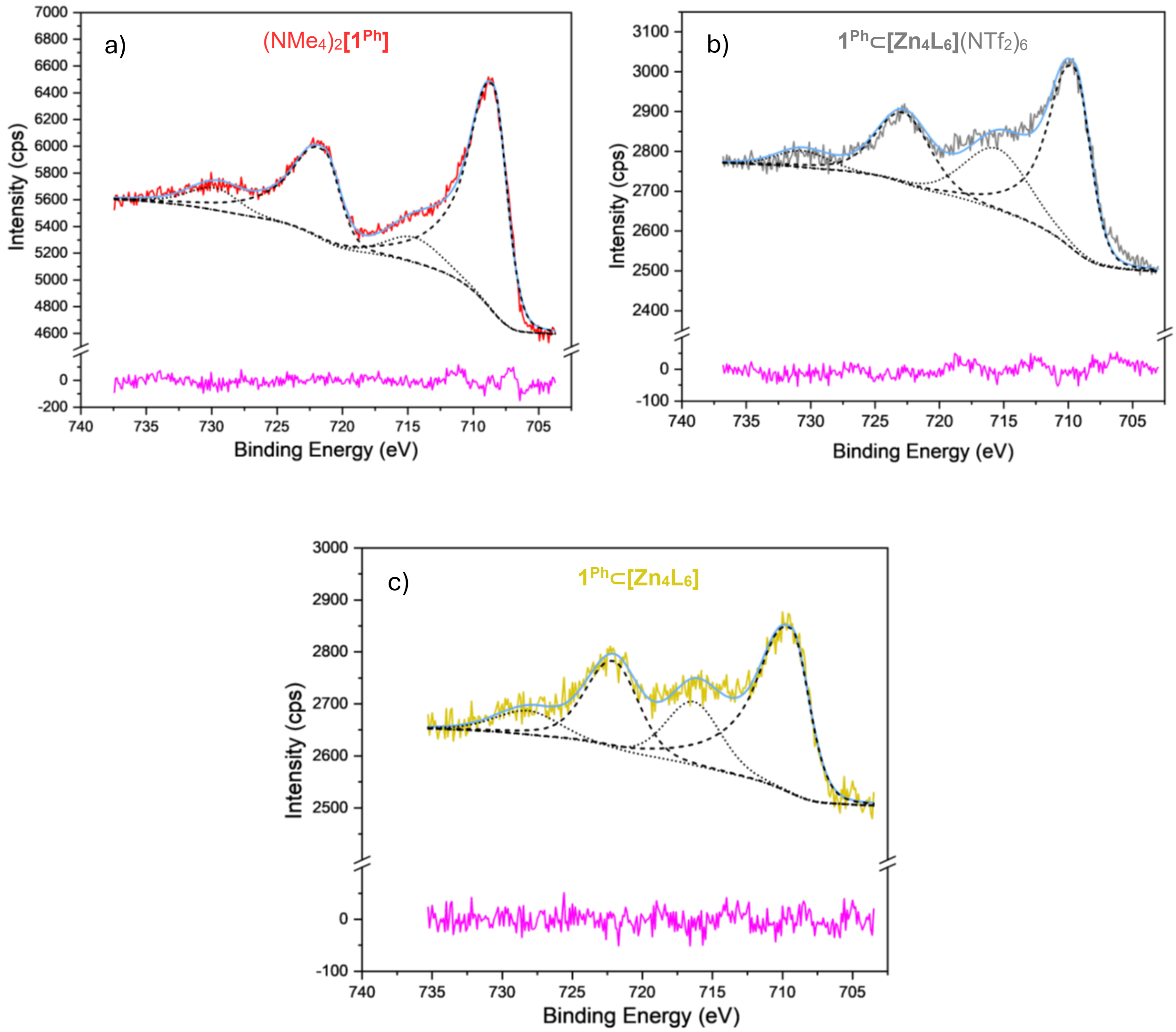

Figure S85. Experimental (colored lines) and simulated (blue lines) Fe *2p* high-resolution XPS spectra of (a) $(NMe_4)_2$**[1^{Ph}]**, (b) **1^{Ph}⊂[Zn$_4$L$_6$]**$(NTf_2)_6$, and (c) **1^{Ph}⊂[Zn$_4$L$_6$]** with satellite peaks highlighted in black dotted lines and the fittings for the 2$p_{1/2}$ and 2$p_{3/2}$ peaks are shown with black dashed lines. Residuals (data – global fit, pink line) are plotted beneath each spectrum; the goodness-of-fit χ^2 are 1.98, 2.15 and 1.27 for (a)–(c), respectively.

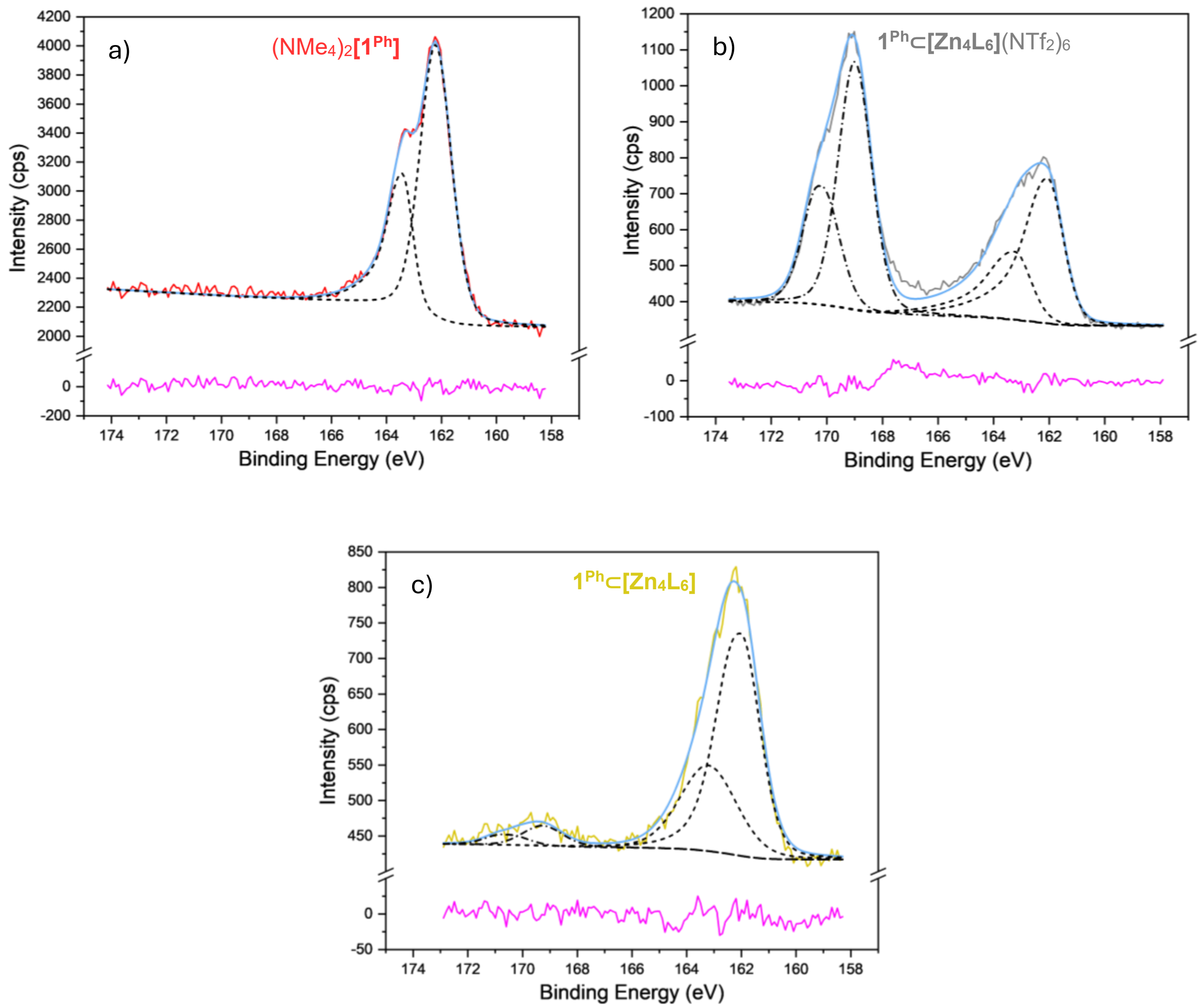

Figure S86. Experimental (colored lines) and simulated (blue lines) S *2p* high-resolution XPS spectra of (a) $(NMe_4)_2$**[1^{Ph}]**, (b) **1^{Ph}⊂[Zn_4L_6]**$(NTf_2)_6$, and (c) **1^{Ph}⊂[Zn_4L_6]** and the fittings for the $2p_{1/2}$ and $2p_{3/2}$ peaks of $-SO_2$ are shown in black dot-dashed lines and -SPh are shown with black dashed lines. Residuals (data – global fit, pink line) are plotted beneath each spectrum; the goodness-of-fit χ^2 are 1.86, 3.90 and 1.85 for (a)–(c), respectively.

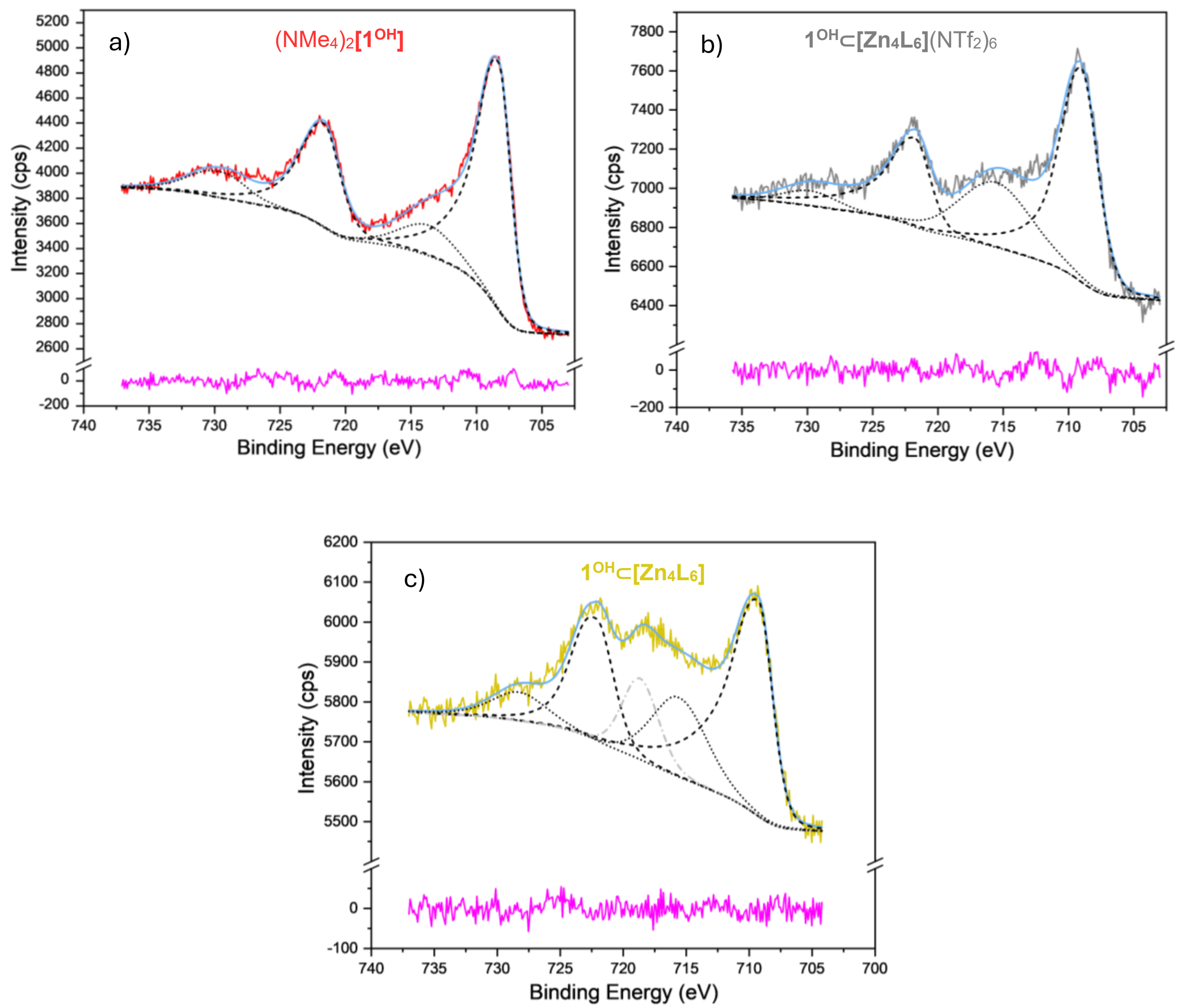

Figure S87. Experimental (colored lines) and simulated (blue lines) Fe *2p* high-resolution XPS spectra of (a) $(NMe_4)_2$**[1^{OH}]**, (b) **1^{OH}⊂[Zn_4L_6]**$(NTf_2)_6$, and (c) **1^{OH}⊂[Zn_4L_6]** with satellite peaks highlighted in black dotted lines and the fittings for the $2p_{1/2}$ and $2p_{3/2}$ peaks are shown with black dashed lines. Co LMM Auger peak is highlighted in gray dash dot line. Residuals (data – global fit, pink line) are plotted beneath each spectrum; the goodness-of-fit χ^2 are 2.90, 1.62 and 1.55 for (a)–(c), respectively.

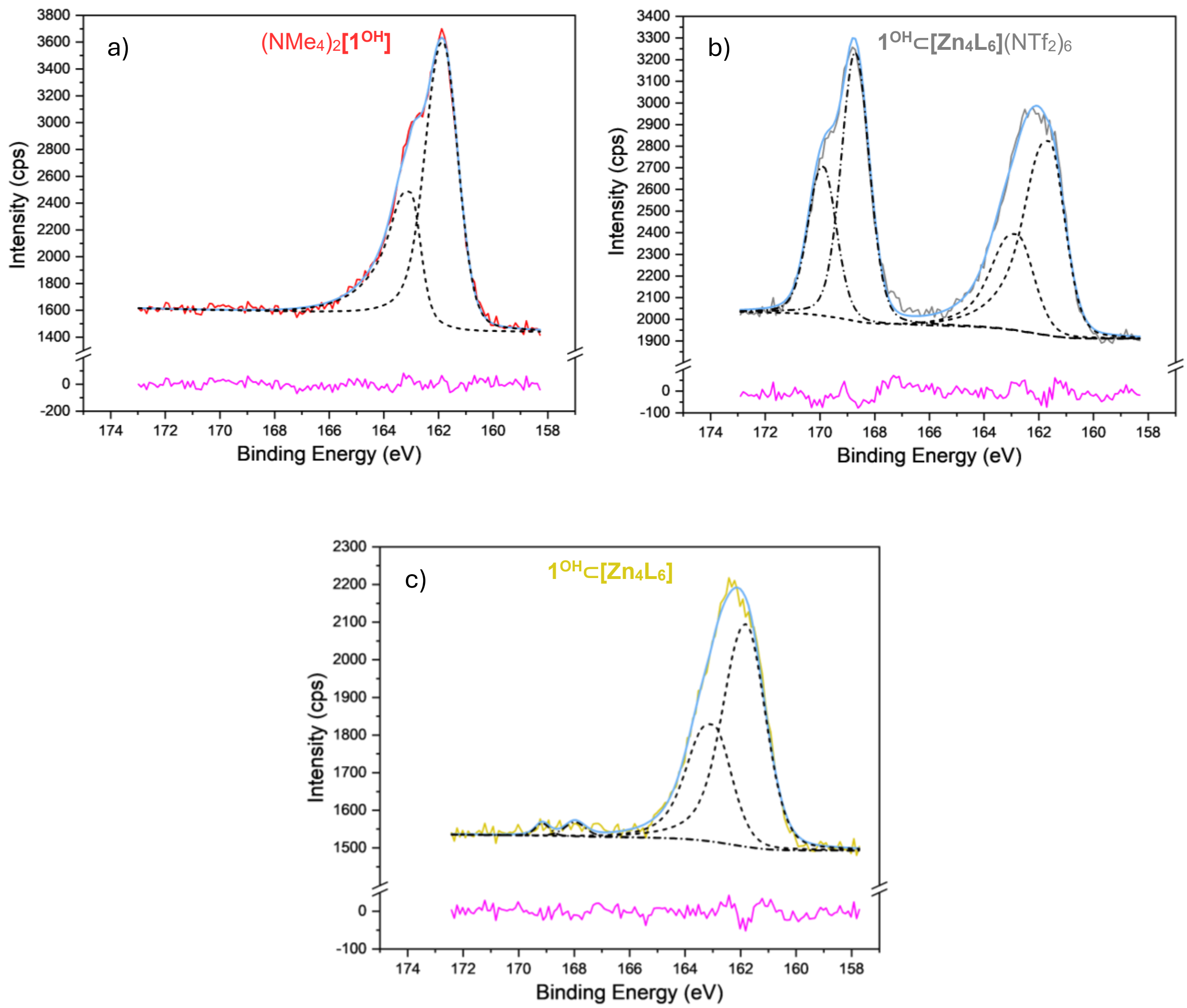

Figure S88. Experimental (colored lines) and simulated (blue lines) S *2p* high-resolution XPS spectra of (a) $(NMe_4)_2$**[1^{OH}]**, (b) **1^{OH}⊂[Zn_4L_6]**$(NTf_2)_6$, and (c) **1^{OH}⊂[Zn_4L_6]** and the fittings for the $2p_{1/2}$ and $2p_{3/2}$ peaks of -SO_2 are shown in black dot-dashed lines and -SPh are shown with black dashed lines. Residuals (data – global fit, pink line) are plotted beneath each spectrum; the goodness-of-fit χ^2 are 1.02, 2.10 and 1.73 for (a)–(c), respectively.

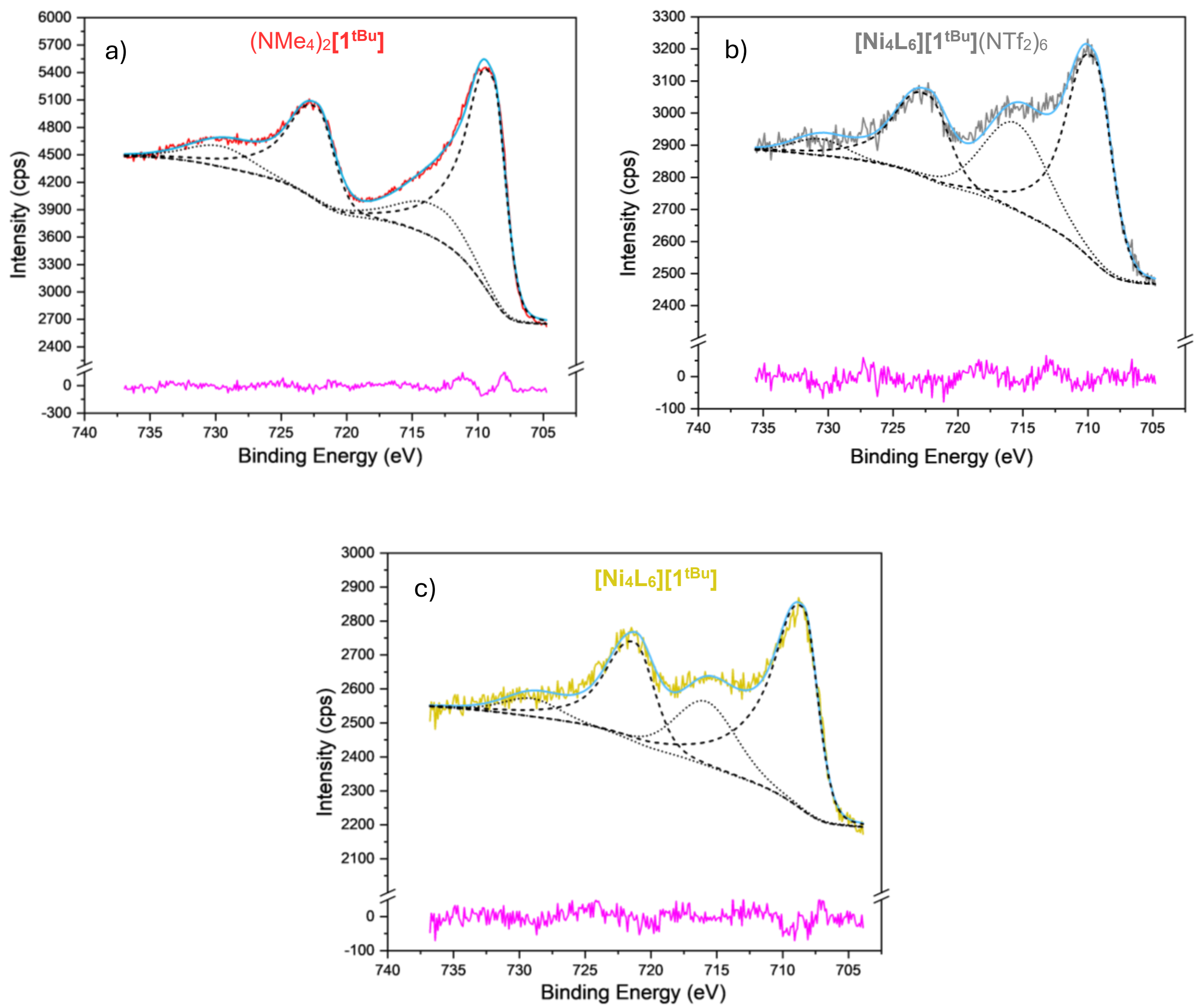

Figure S89. Experimental (colored lines) and simulated (blue lines) Fe *2p* high-resolution XPS spectra of (a) $(NMe_4)_2$**[1^{tBu}]**, (b) **[Ni_4L_6][1^{tBu}]**$(NTf_2)_6$, and (c) **[Ni_4L_6][1^{tBu}]** with satellite peaks highlighted in black dotted lines and the fittings for the $2p_{1/2}$ and $2p_{3/2}$ peaks are shown with black dashed lines. Residuals (data – global fit, pink line) are plotted beneath each spectrum; the goodness-of-fit χ^2 are 2.9, 1.62 and 1.55 for (a)–(c), respectively.

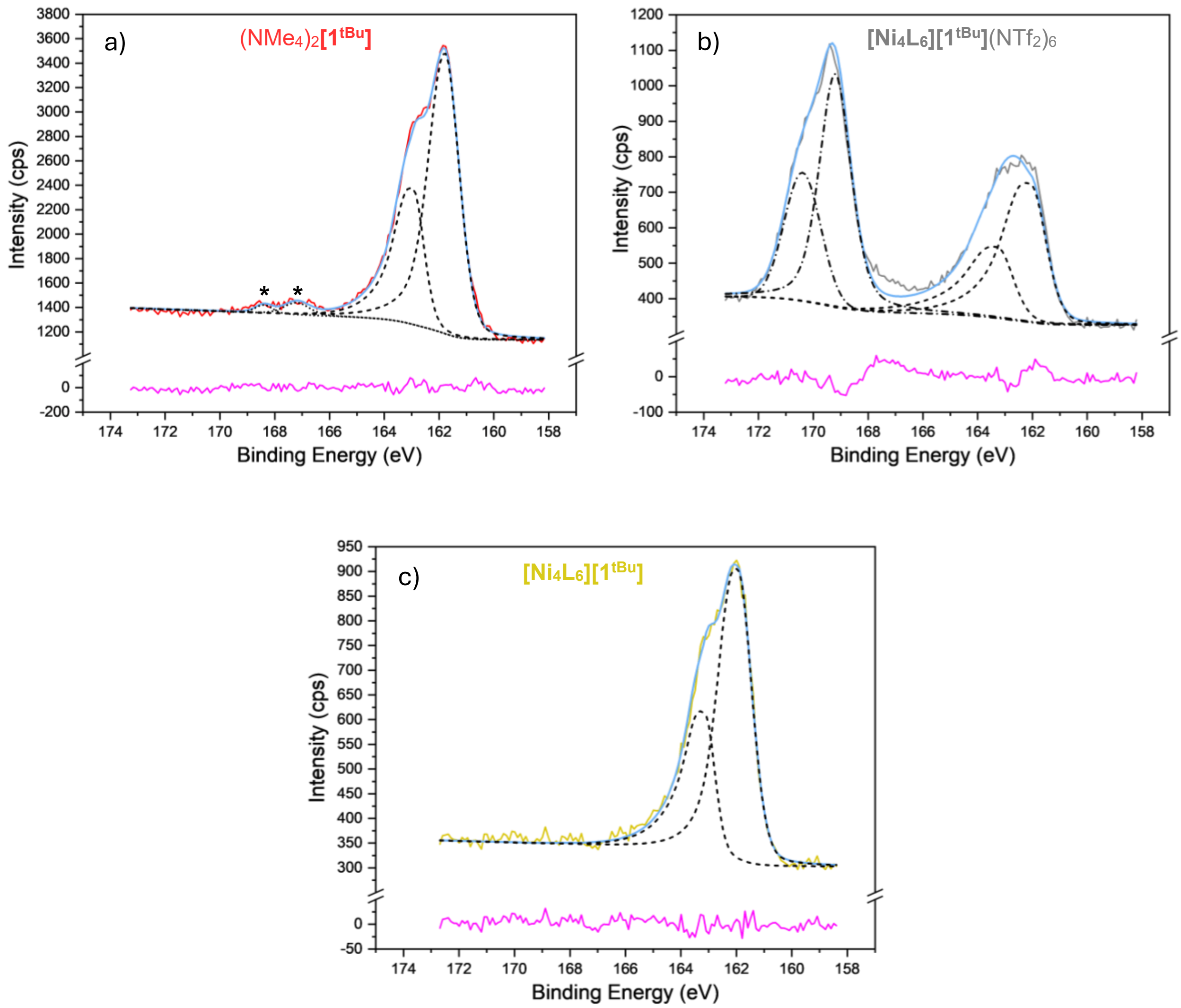

Figure S90. Experimental (colored lines) and simulated (blue lines) S *2p* high-resolution XPS spectra of (a) $(NMe_4)_2$**[1^{tBu}]**, (b) **[Ni_4L_6][1^{tBu}]**$(NTf_2)_6$, and (c) **[Ni_4L_6][1^{tBu}]** and the fittings for the $2p_{1/2}$ and $2p_{3/2}$ peaks of -SO_2 are shown in black dot-dashed lines and -SPh are shown with black dashed lines. Residuals (data – global fit, pink line) are plotted beneath each spectrum; the goodness-of-fit χ^2 are 1.79, 3.88 and 1.52 for (a)–(c), respectively. *unidentified impurities.

S10. UV-VIS AND IR SPECTROSCOPY

Analysis of the UV-vis spectra of **[1^R]$^{2-}$** and **1^R⊂[M$_4$L$_6$]**(NTf$_2$)$_6$ revealed a consistent red shift in the characteristic S→Fe LMCT band[24] consistent with the accepting Fe orbitals becoming more electron-deficient on encapsulation, while we see negligible change for ion pair **[M$_4$L$_6$][1^{tBu}]**(NTf$_2$)$_6$ (Table S6; Figures S92-94). This shift is most apparent on encapsulation for **1^R⊂[M$_4$L$_6$]**(NTf$_2$) (M = Ni, Zn), since the Fe cage **[M$_4$L$_6$]**(NTf$_2$)$_8$ exhibits overlapping transitions in the visible region between 450-600 nm. By contrast, negligible differences are observed in the cage charge-transfer bands on encapsulation (Figures S91 and S96), similar to prior reports of perylenebisimide (PBI)-panelled cage reported by Mahata *et al*.[25]

Table S6. UV-vis spectral parameters for (NMe$_4$)$_2$**[1^R]** and **1^R⊂[M$_4$L$_6$]**(NTf$_2$)$_6$ (M = Fe, Zn, Ni) and ion pair **[Ni$_4$L$_6$][1^{tBu}]**(NTf$_2$)$_6$ taken at RT in MeCN on mixing 1:1 equiv of (NMe$_4$)$_2$**[1^R]** and **[M$_4$L$_6$]**(NTf$_2$)$_8$ to obtain a solution of 5 × 10^{-6} M.

complex	S→Fe LMCT transition energy	
	nm	eV
(NMe$_4$)$_2$**[1^{Ph}]**	452	2.74
**1^{Ph}⊂[Ni$_4$L$_6$]**(NTf$_2$)$_6$	471 (+19)	2.63 (–0.11)
**1^{Ph}⊂[Zn$_4$L$_6$]**(NTf$_2$)$_6$	472 (+20)	2.63 (–0.11)
(NMe$_4$)$_2$**[1^{OH}]**	410	3.02
**1^{OH}⊂[Ni$_4$L$_6$]**(NTf$_2$)$_6$	436 (+26)	2.84 (–0.18)
**1^{OH}⊂[Zn$_4$L$_6$]**(NTf$_2$)$_6$	435 (+25)	2.85 (–0.17)
(NMe$_4$)$_2$**[1^{tBu}]**	426	2.91
**[Ni$_4$L$_6$][1^{tBu}]**(NTf$_2$)$_6$	428 (+2)	2.90 (–0.01)

Relatedly, analysis of the IR spectra of **[1^R]$^{2-}$**, **1^R⊂[M$_4$L$_6$]**(NTf$_2$)$_6$ and ion pair **[Ni$_4$L$_6$][1^{tBu}]**(NTf$_2$)$_6$ revealed a slight decrease in the C=O and C=C stretching bands of the cage (–0-1.5 cm^{-1}; Table S7; Figures S98, S99 and S101). The C=C and C=O NDI stretching bands of the cage were assigned by comparison to the literature values for the NDI groups.[26] At the same time, the C=C stretching bands of **[1^R]$^{2-}$** are observed to increase slightly. These observations could be due to inductive electron density withdrawal to/from the thiolate ligands/NDI group due to strong electrostatic interactions between the electron-poor NDI and electron-rich thiolate ligands. This effect is explained in more detail in the main text.

Table S7. IR spectral parameters for clusters (NMe$_4$)$_2$**[1^R]** and host-guest complexes **1^R⊂[M$_4$L$_6$]**(NTf$_2$)$_6$ (M = Zn, Ni) and ion pair **[Ni$_4$L$_6$][1^{tBu}]**(NTf$_2$)$_6$ taken at RT in solid state. Samples were prepared according to procedures discussed in Section S3 and S8.

	Cage peaks	
complex	C=O (cm^{-1})	C=C (cm^{-1})
**[Zn$_4$L$_6$]**(NTf$_2$)$_8$	1672.9<br>1715.4	1498.9<br>1582.8<br>1597.3
**1^{Ph}⊂[Zn$_4$L$_6$]**(NTf$_2$)$_6$	1672.9 (+0.0)<br>1714.8 (–0.4)	1498.4 (–0.5)<br>1581.3 (–1.5)<br>1596.3 (–1.0)
**1^{OH}⊂[Zn$_4$L$_6$]**(NTf$_2$)$_6$	1670.0 (–2.9)<br>1714.4 (–1.0)	1498.9 (+0.0)<br>1581.8 (–1.0)<br>1596.8 (–0.5)
**[Ni$_4$L$_6$]**(NTf$_2$)$_8$	1673.9<br>1715.9	1498.9<br>1583.3

		1597.3
$[Ni_4L_6][1^{tBu}](NTf_2)_6$	1673.9 (+0.0)<br>1715.9 (+0.0)	1498.9 (+0.0)<br>1582.8 (–0.5)<br>1596.3 (–1.0)
**Cluster functional group peaks**		
compound	O-H (cm^{-1})	C=C (cm^{-1})
$(NMe_4)_2[\mathbf{1^{Ph}}]$		1473.8
$\mathbf{1^{Ph}}\subset[Zn_4L_6](NTf_2)_6$		1476.2 (+2.4)
$(NMe_4)_2[\mathbf{1^{OH}}]$	3273.6	
$\mathbf{1^{OH}}\subset[Zn_4L_6](NTf_2)_6$	3300.1 (+26.5)	

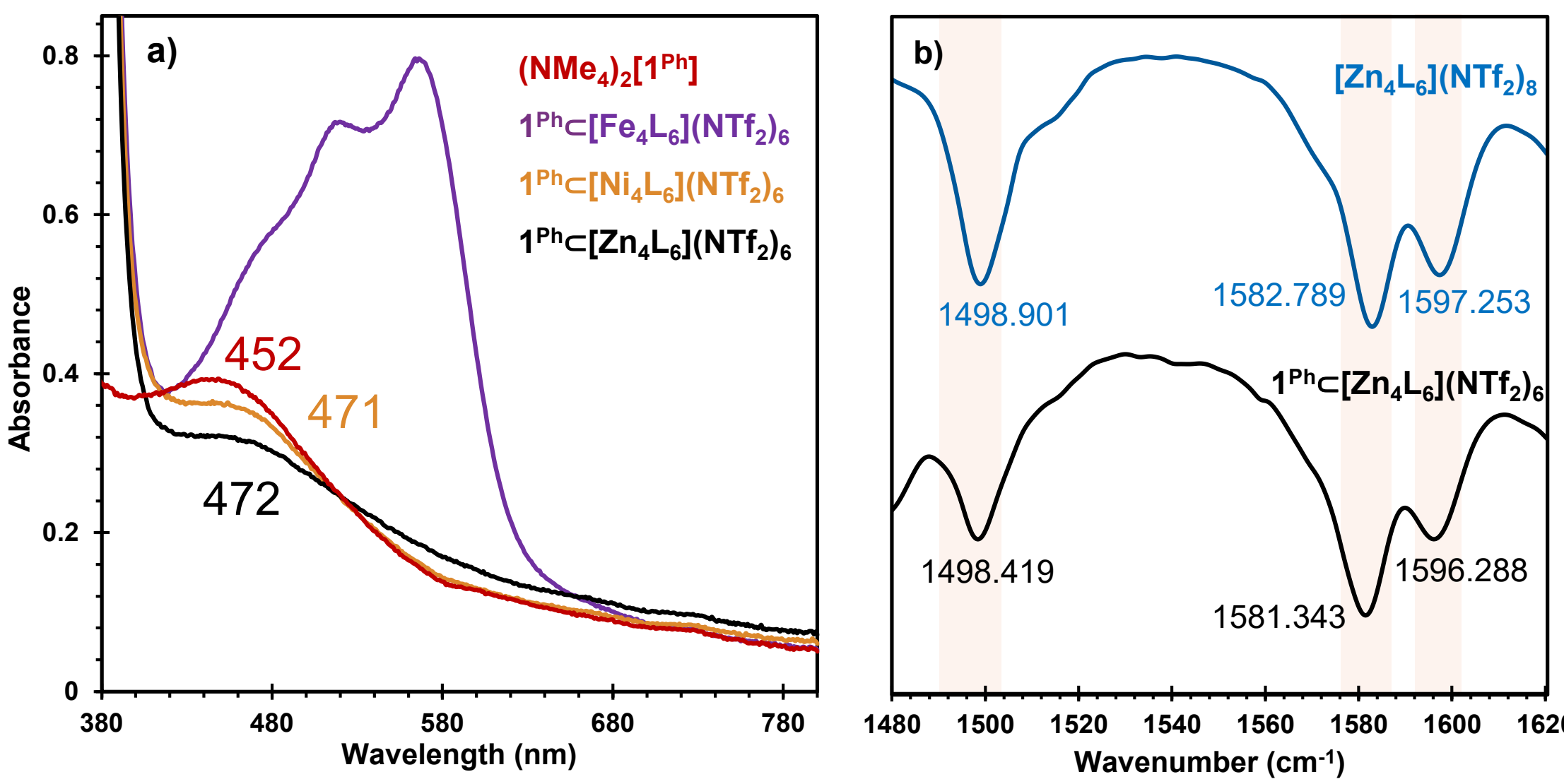

Figure S91. (a) UV-Visible spectra of $(NMe_4)_2[\mathbf{1^{Ph}}]$ and $\mathbf{1^{Ph}}\subset[\mathbf{M_4L_6}](NTf_2)_6$ (M = Fe, Ni, Zn); (b) magnetified IR spectra of $[\mathbf{Zn_4L_6}](NTf_2)_8$ and $\mathbf{1^{Ph}}\subset[\mathbf{Zn_4L_6}](NTf_2)_6$ showing the IR stretching frequencies for C=C bond of the linker.

UV-vis spectra

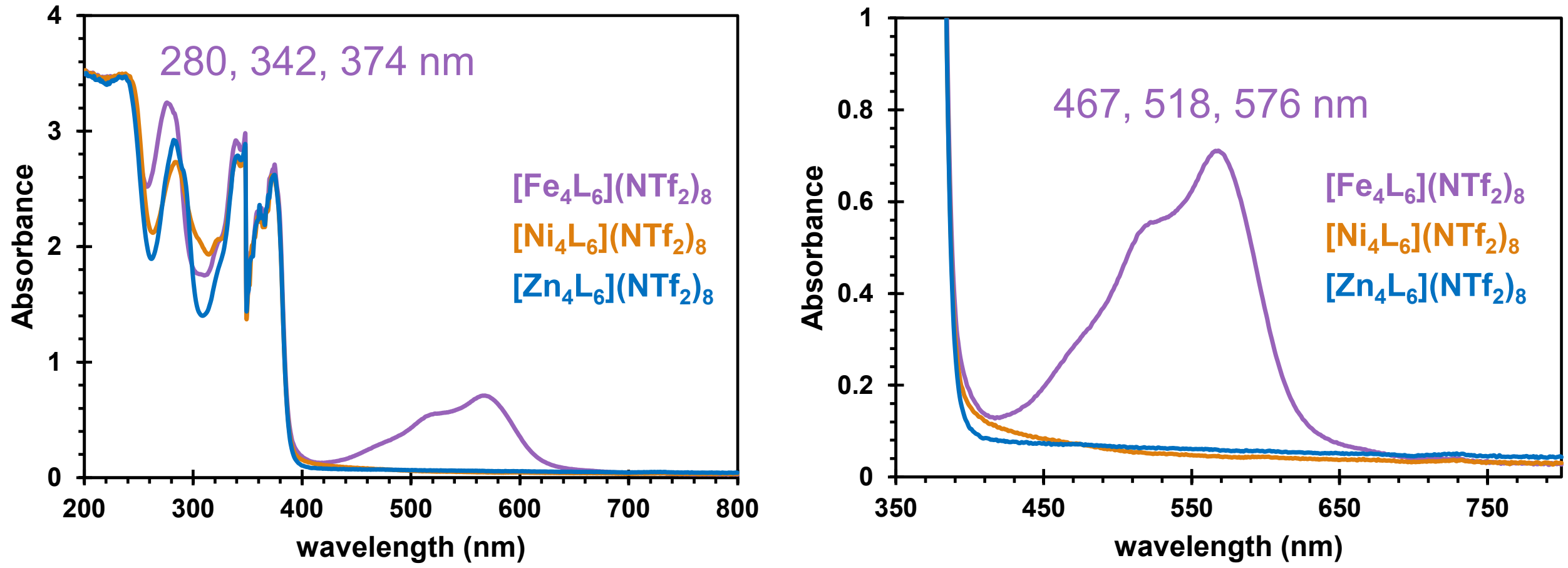

Figure S92. Stacked UV-vis spectra of **[M_4L_6]**$(NTf_2)_8$ (M= Fe, Ni, Zn). (Left) full spectrum highlighting π-π* transitions assigned to the NDI ring for **[Fe_4L_6]**$(NTf_2)_8$ as an exemplar in region 250-400 nm. (Right) Magnified view of the region 350-800 nm. Conditions: 5×10^{-6} M in MeCN. There is a break in the spectrum at 350 nm due to a change in the lamp used 200 to 800 nm.

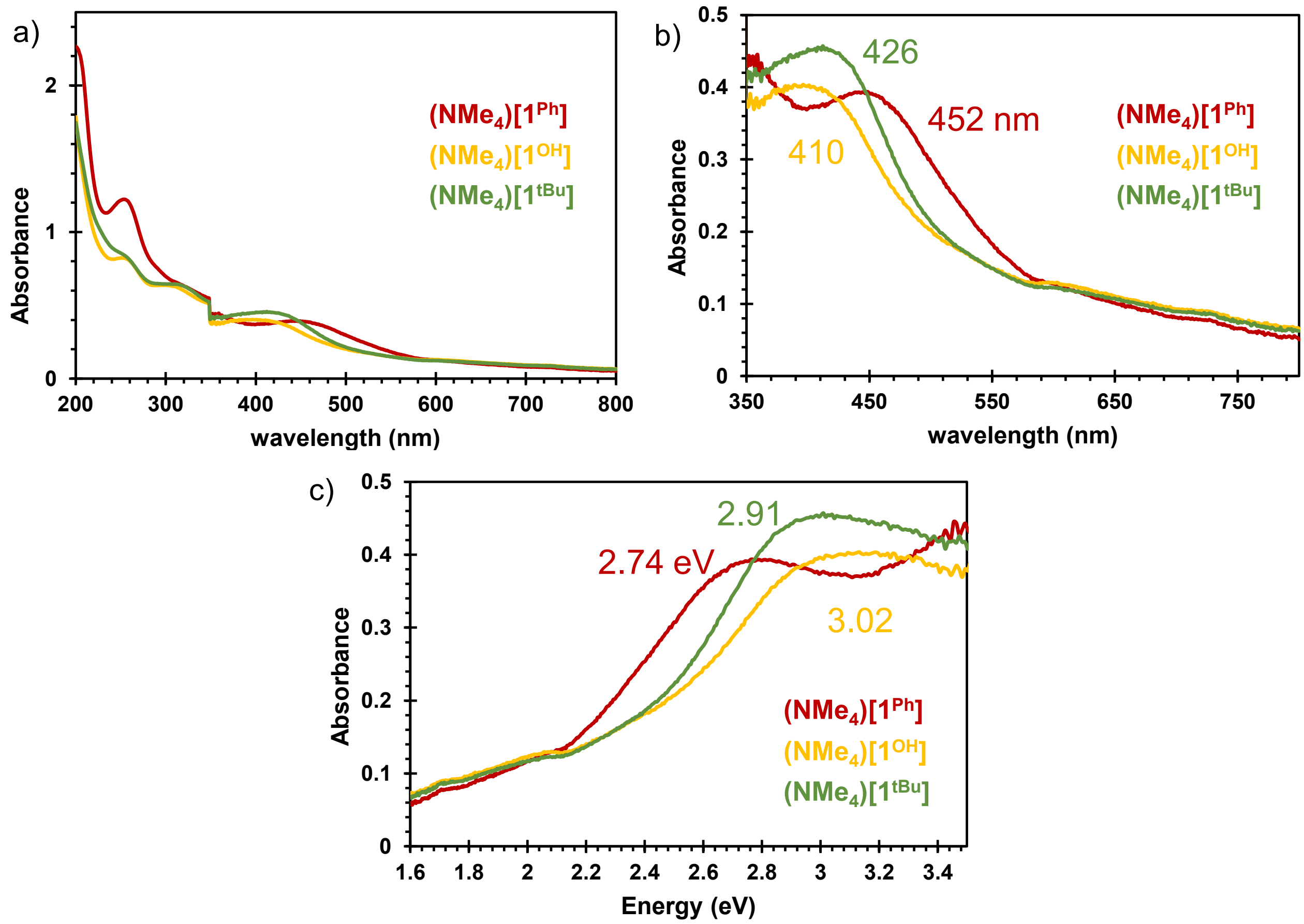

Figure S93. Stacked UV-vis spectra of $(NMe_4)_2$**[1^R]**. a) Full spectrum of the region 200-800 nm. Magnified view of the region b) 350-800 nm and c) 1.6-3.5 eV. Conditions: 5×10^{-6} M in MeCN. There is a break in the spectrum at 350 nm due to a change in the lamp used 200 to 800 nm.

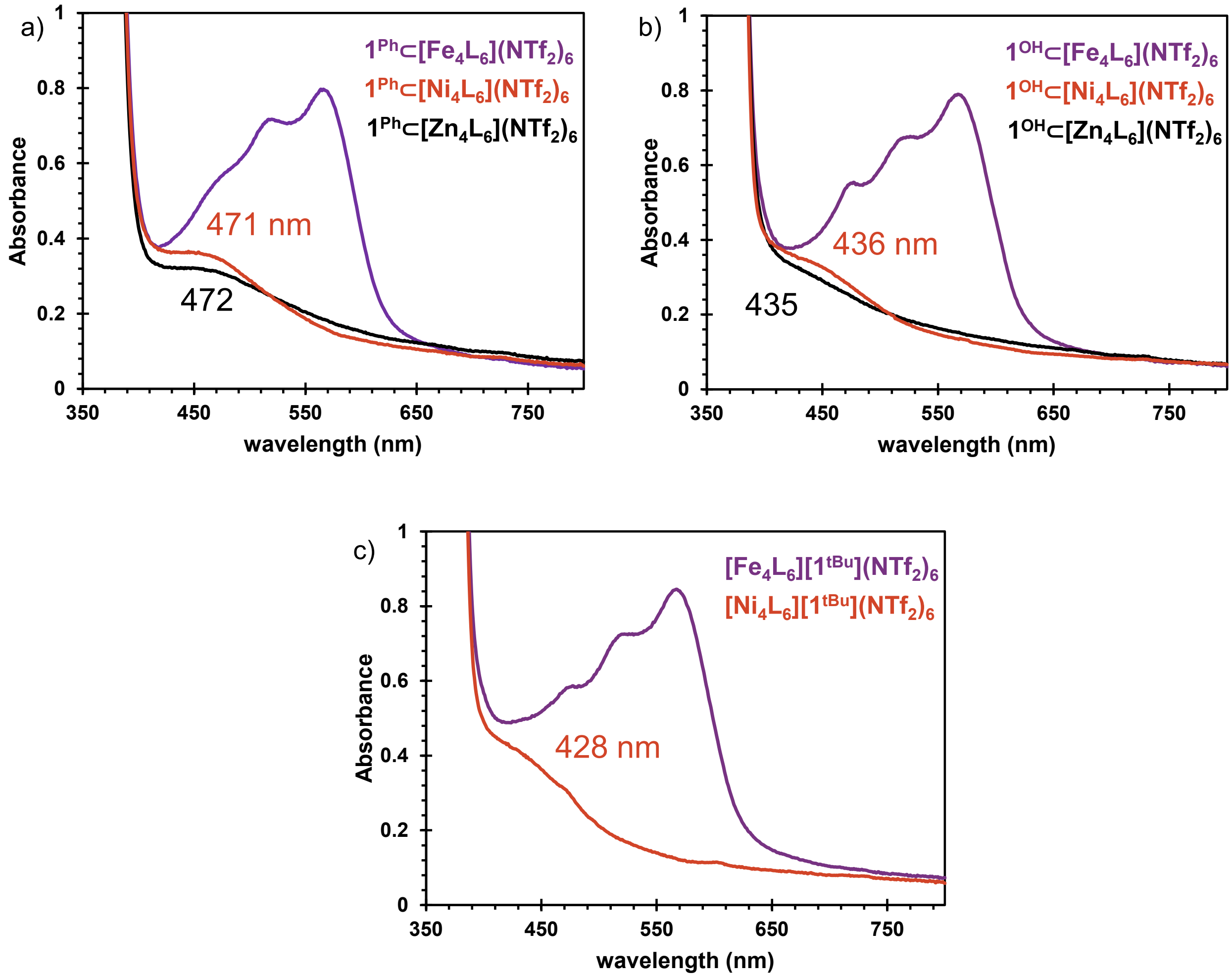

Figure S94. Stacked UV-vis spectra of a) $\mathbf{1^{Ph}{\subset}[M_4L_6]}(NTf_2)_6$, b) $\mathbf{1^{OH}{\subset}[M_4L_6]}(NTf_2)_6$, and c) $\mathbf{[M_4L_6][1^{tBu}]}(NTf_2)_6$ (M= Fe, Ni and Zn) highlighting the S→Fe LMCT band in the region 350-800 nm. Conditions: 5×10^{-6} M in MeCN.

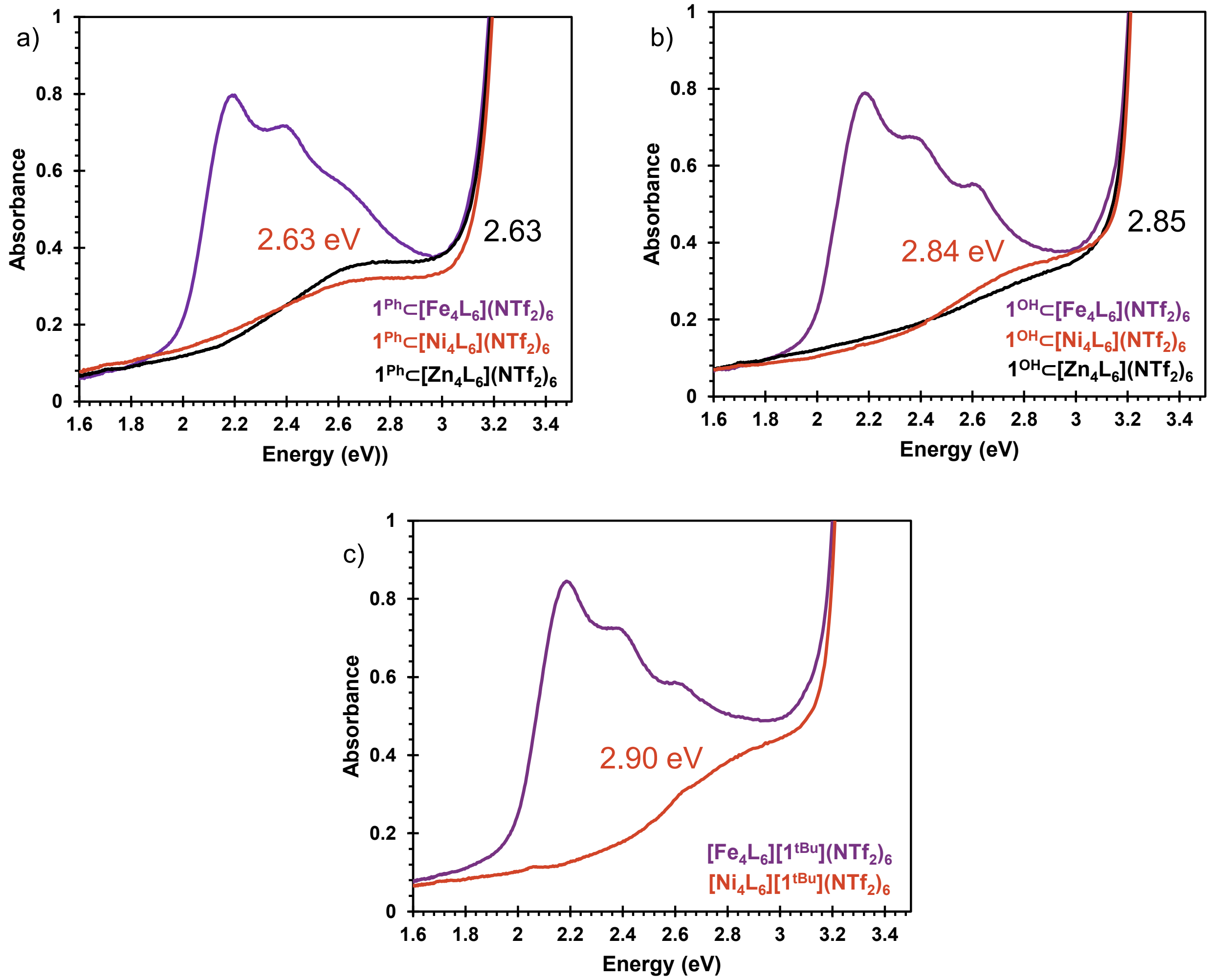

Figure S95. Stacked UV-vis spectra of a) $\mathbf{1^{Ph}\subset[M_4L_6]}(NTf_2)_6$, b) $\mathbf{1^{OH}\subset[M_4L_6]}(NTf_2)_6$, and c) $\mathbf{[M_4L_6][1^{tBu}]}(NTf_2)_6$ (M= Fe, Ni and Zn) highlighting the S→Fe LMCT band in the region 1.6-3.5 eV. Conditions: 5×10^{-6} M in MeCN.

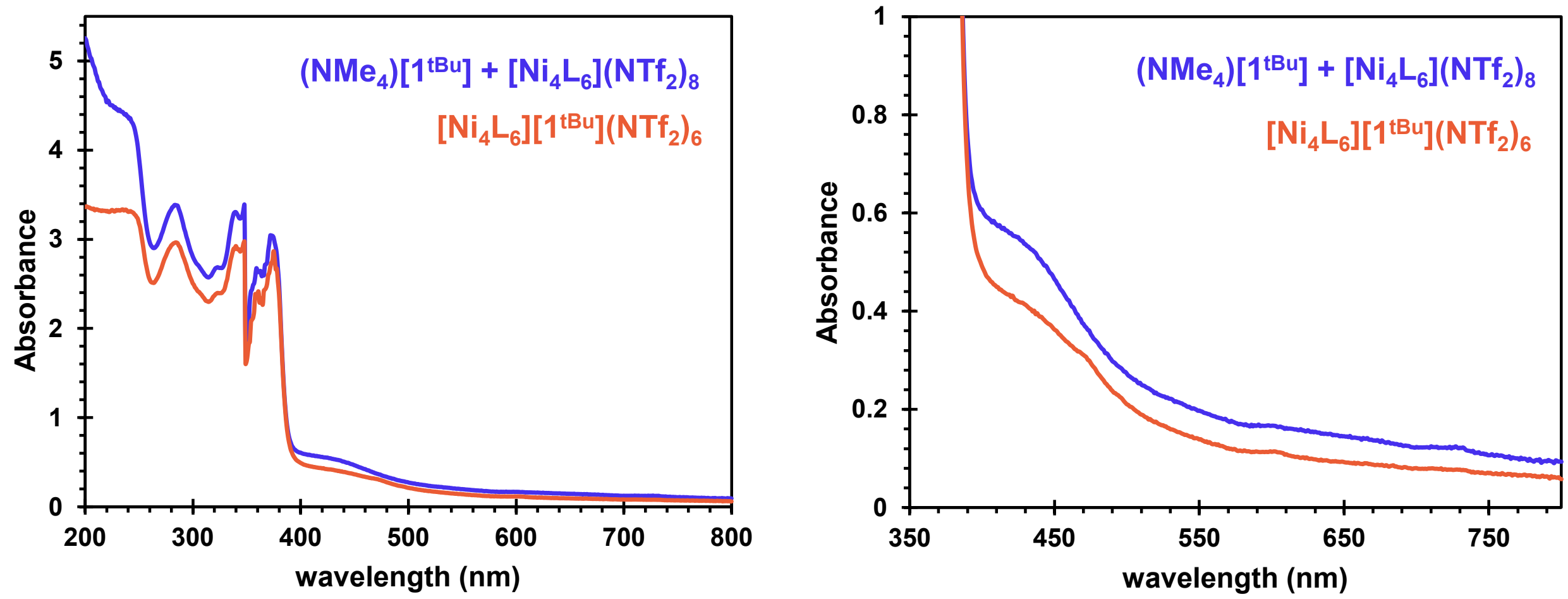

Figure S96. Stacked UV-vis spectra of $(NMe_4)_2$**[1^{tBu}] + [Ni$_4$L$_6$]**$(NTf_2)_8$ and **[Ni$_4$L$_6$][1^{tBu}]**$(NTf_2)_6$. (Left) full spectrum in region 250-400 nm. (Right) Magnified view of the region 350-800 nm. Conditions: 5×10^{-6} M in MeCN. There is a break in the spectrum at 350 nm due to a change in the lamp used 200 to 800 nm.

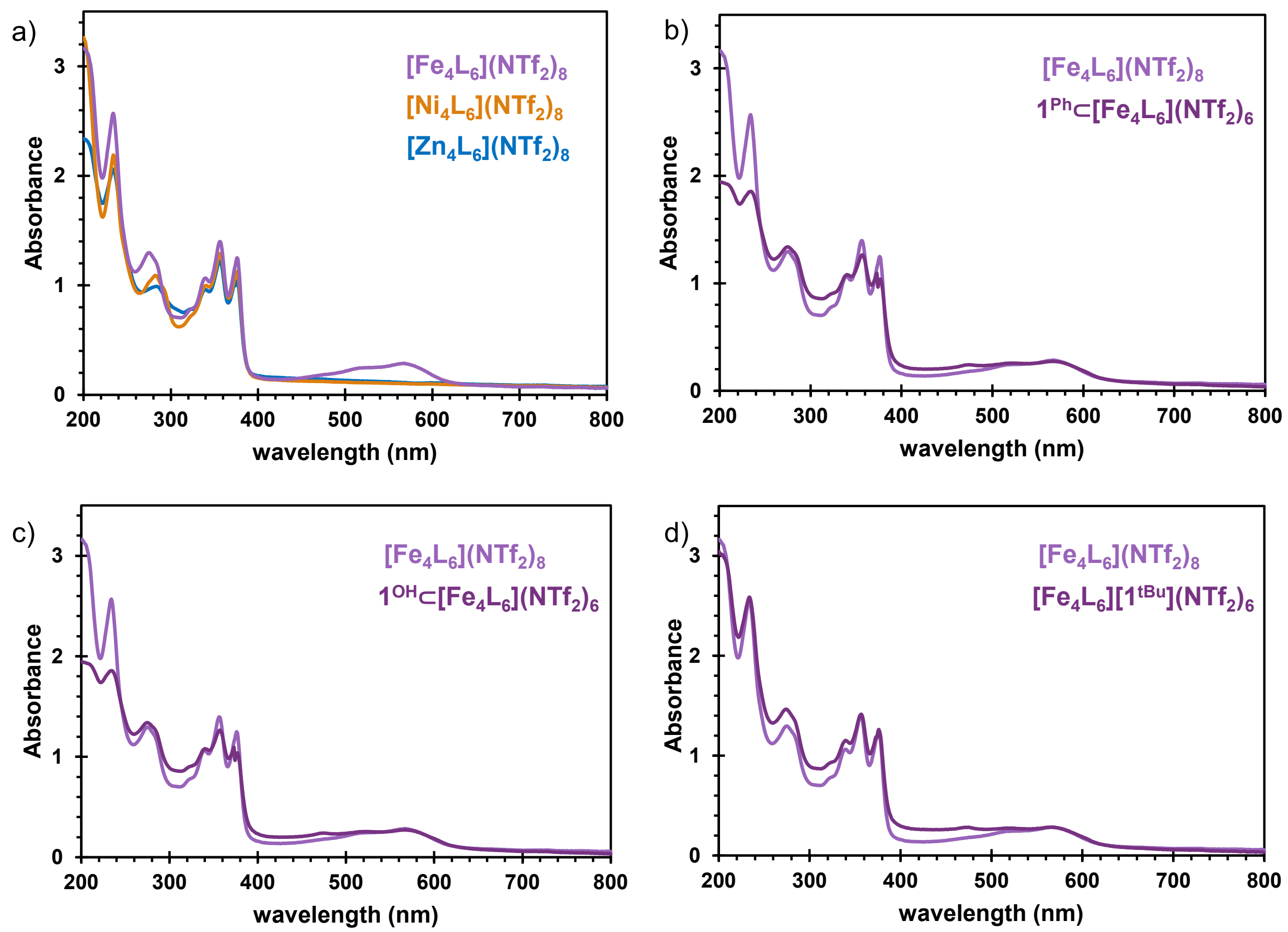

Figure S97. Stacked UV-vis spectra of a) **[M$_4$L$_6$]**$(NTf_2)_8$, b) **1^{Ph}⊂[M$_4$L$_6$]**$(NTf_2)_6$, and c) **1^{OH}⊂[M$_4$L$_6$]**$(NTf_2)_6$, and **[M$_4$L$_6$][1^{tBu}]**$(NTf_2)_6$ in the full region 200-800 nm. Conditions: 2.5×10^{-6} M in MeCN. There is a break in the spectrum at 375 nm due to a change in the lamp used 200 to 800 nm.

IR Spectra

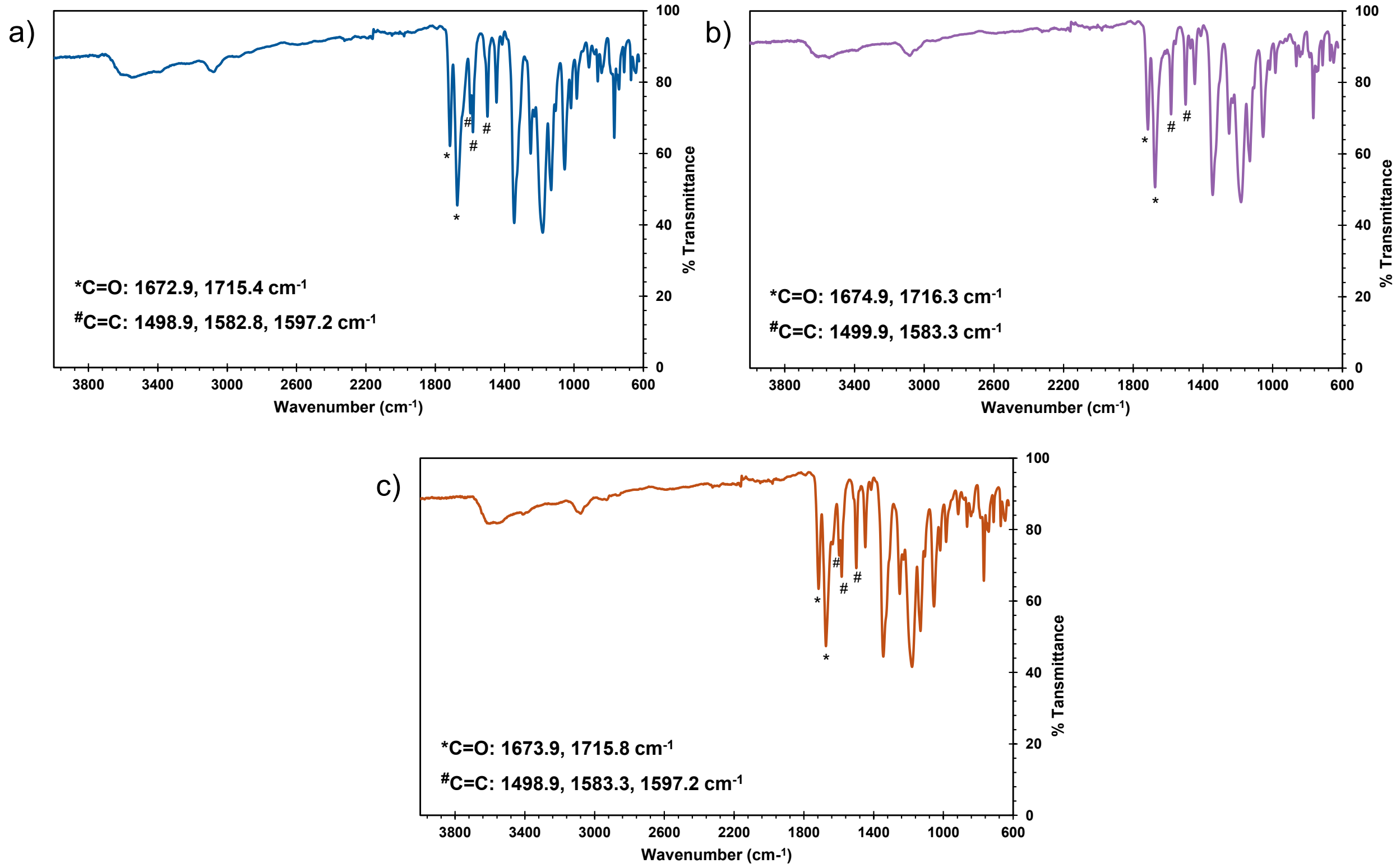

Figure S98. ATIR spectra of (a) **[Zn$_4$L$_6$]**(NTf$_2$)$_8$, (b) **[Fe$_4$L$_6$]**(NTf$_2$)$_8$, and (c) **[Ni$_4$L$_6$]**(NTf$_2$)$_8$ highlighting peaks assigned to the C=C and C=O stretching bands.

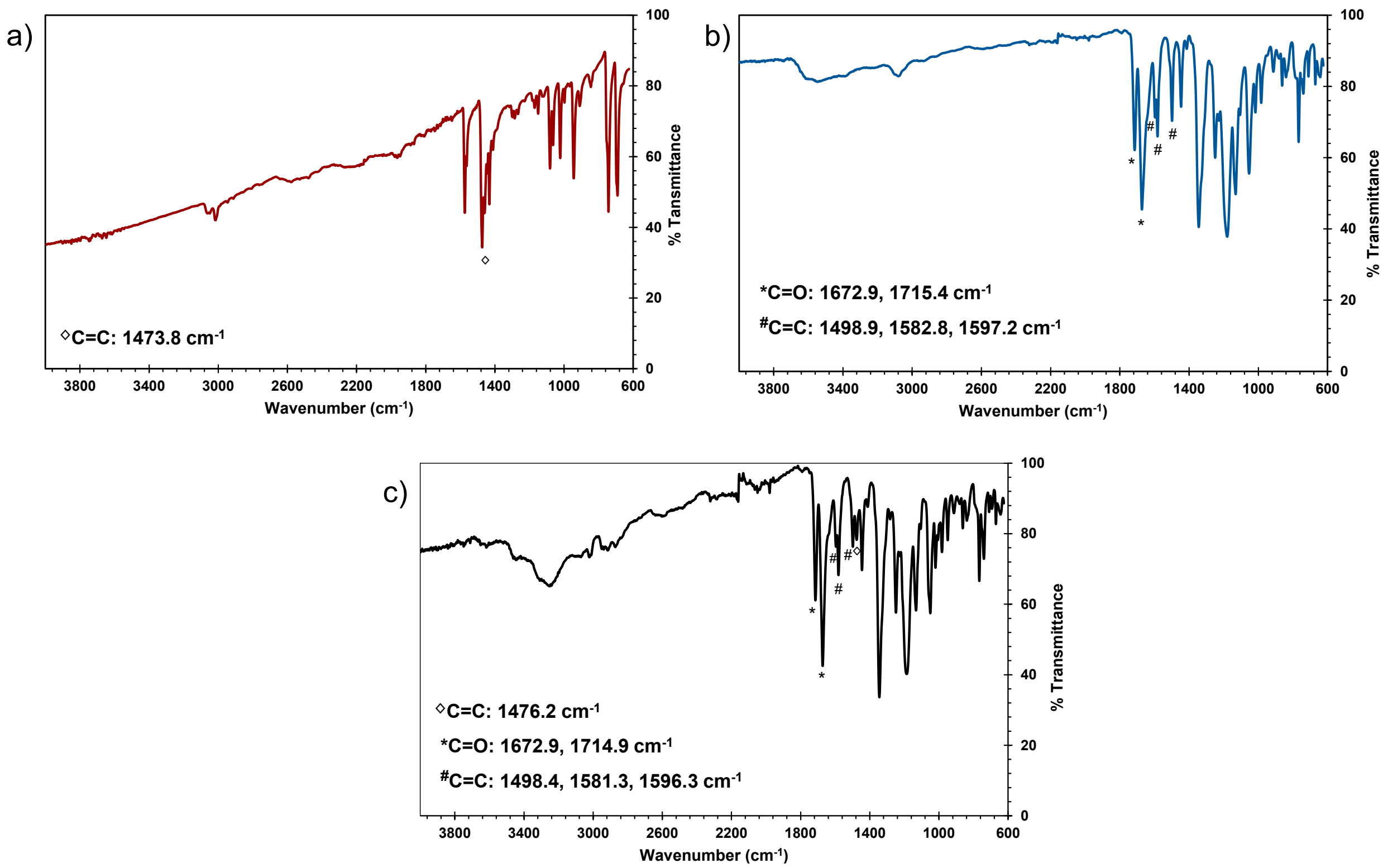

Figure S99. ATIR spectra of a) $(NMe_4)_2$**[1^{Ph}]**, b) **[Zn_4L_6]**$(NTf_2)_8$, and c) **1^{Ph}⊂[Zn_4L_6]**$(NTf_2)_6$ with the high-lighting peaks assigned to the C=C and C=O stretching bands.

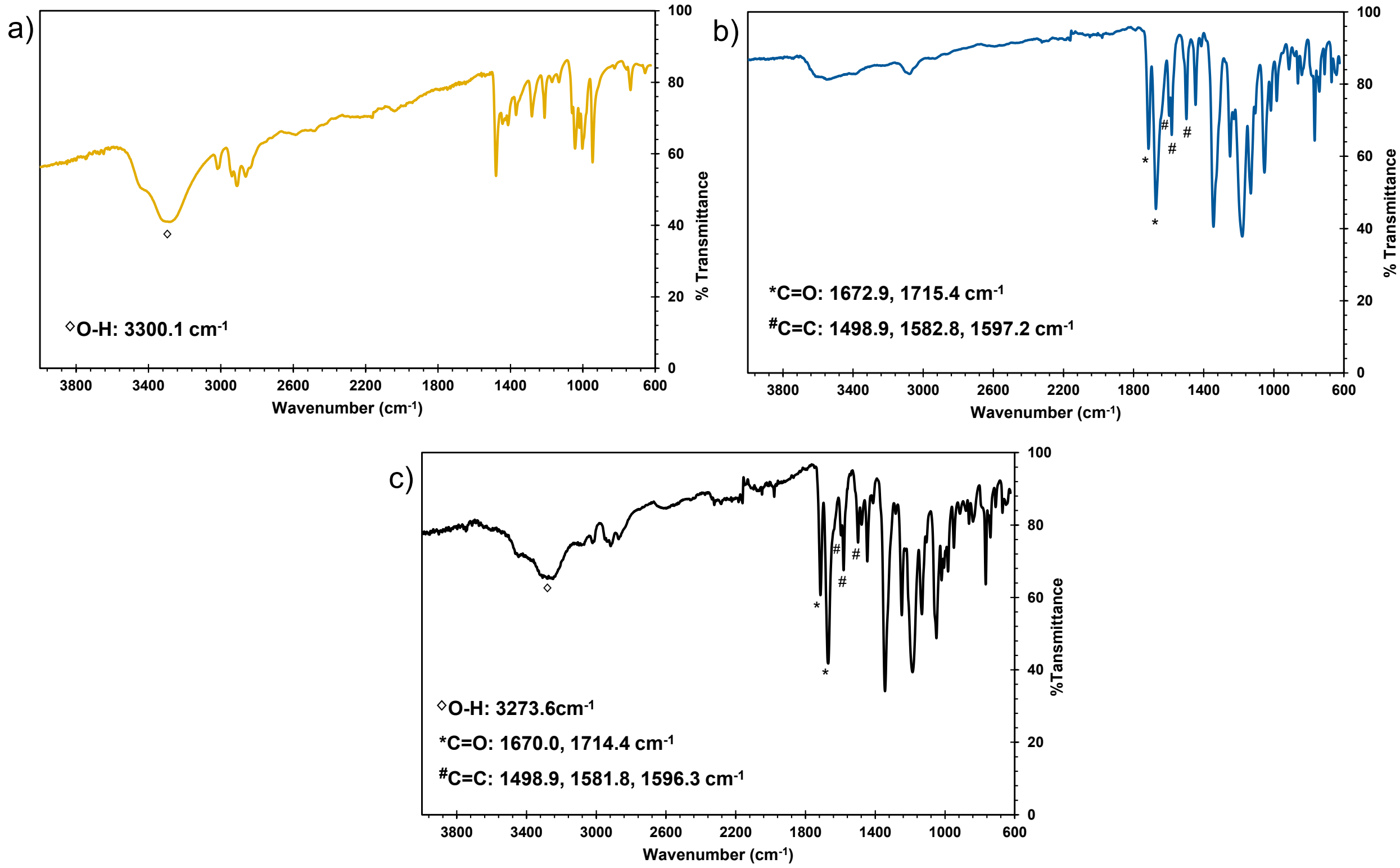

Figure S100. ATIR spectra of a) $(NMe_4)_2$**[1^{OH}]**, b) **[Zn_4L_6]**$(NTf_2)_8$, and c) **1^{OH}⊂[Zn_4L_6]**$(NTf_2)_6$ highlighting peaks assigned to the C=C, C=O and O-H stretching bands.

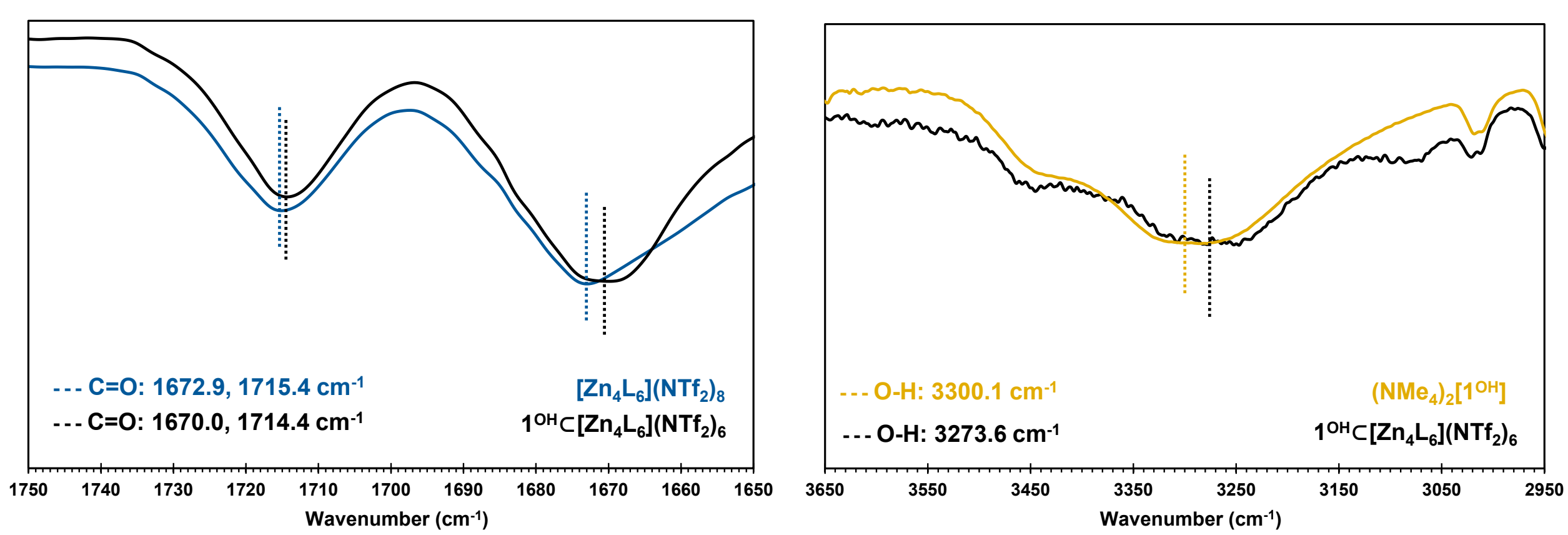

Figure S101. Stacked ATIR spectra of **[Zn_4L_6]**$(NTf_2)_8$ and **1^{OH}⊂[Zn_4L_6]**$(NTf_2)_6$ (left) highlighting peaks assigned to the C=O stretching bands, and $(NMe_4)_2$**[1^{OH}]** and **1^{OH}⊂[Zn_4L_6]**$(NTf_2)_6$ (right) highlighting peaks assigned to the O-H stretching band.

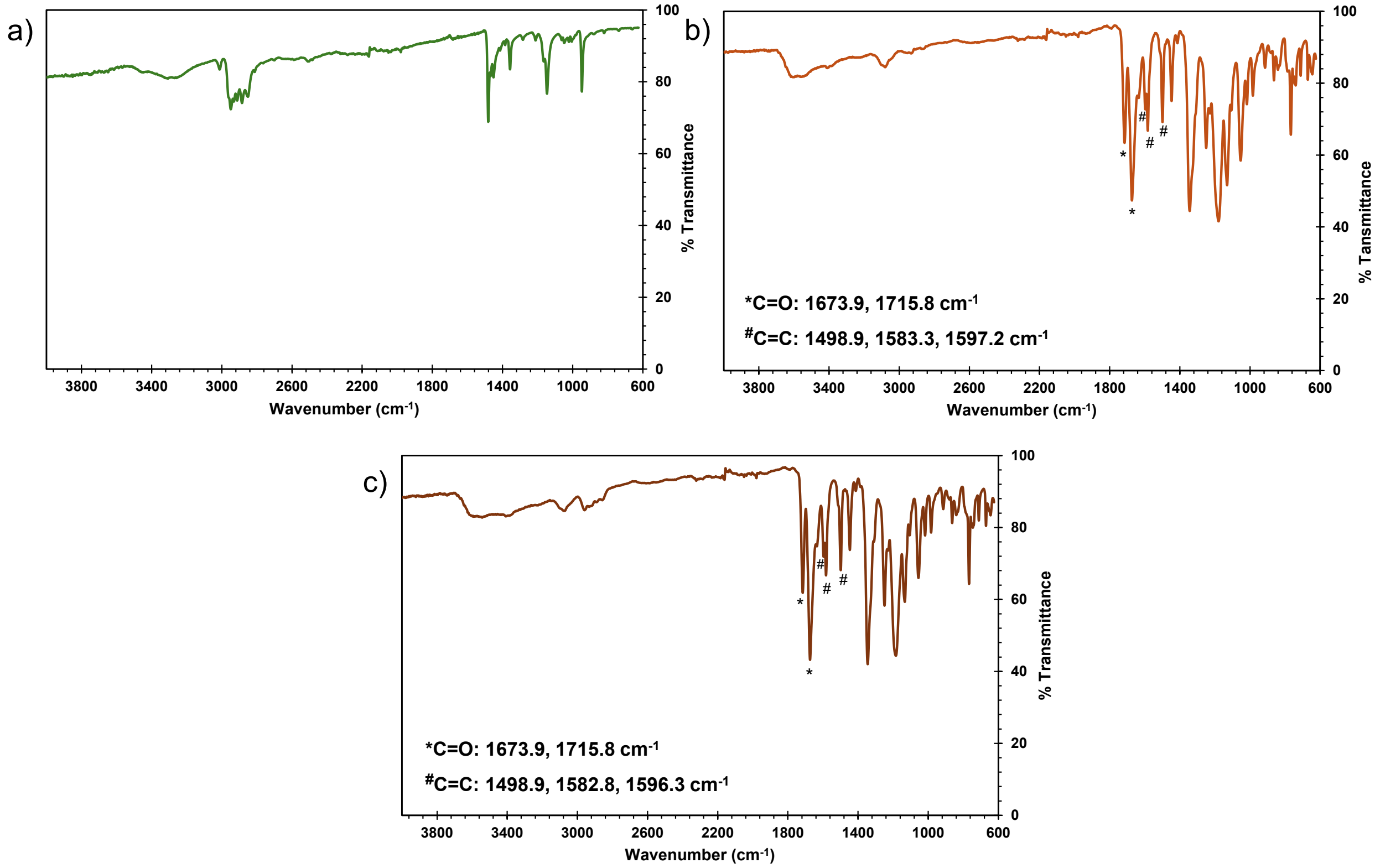

Figure S102. ATIR spectra of a) $(NMe_4)_2$**[1^{tBu}]**, b) **[Ni_4L_6]**$(NTf_2)_8$, and c) **[Ni_4L_6][1^{tBu}]**$(NTf_2)_6$ highlighting peaks assigned to the C=C and C=O stretching bands.

S11. BINDING TITRATION EXPERIMENTS

For 1:1 binding systems, the lower bound of the association constants is calculated by the method described by Thordarson.[27]

$$H + G \underset{}{\overset{K_a}{\rightleftharpoons}} HG$$

$$K_a = \frac{[HG]}{[H][G]}$$

$$p = \frac{[HG]}{[G_0]} \qquad \text{for } [H_0] \geq [G_0]$$

$$K_a = \frac{x}{[H_0-x][G_0-x]}$$

then solve for x.

where [H] = concentration of host species **[Fe_4L_6]**(NTf_2)$_8$

[G] = concentration of guest species (NMe_4)$_2$**[1^R]**

[HG] = x = concentration of host-guest species **1^R⊂[Fe_4L_6]**(NTf_2)$_6$

[H_0] = initial concentration of host species **[Fe_4L_6]**(NTf_2)$_8$

[G_0] = initial concentration of guest species (NMe_4)$_2$**[1^R]**

K_a = association constant

p = probability of binding

Table S8. Summary of ^{1}H NMR chemical shift values of cluster (NMe_4)$_2$**[1^R]** in CD_3CN at RT.

cluster	^{1}H NMR Chemical shift ± 0.01 (ppm)
(NMe_4)$_2$**[1^{Ph}]**	8.20, 5.91, 5.30
(NMe_4)$_2$**[1^{OH}]**	12.25, 4.34, 2.82
(NMe_4)$_2$**[1^{tBu}]**	2.66

Table S9. Summary of ^{1}H NMR chemical shift values of encapsulated cluster **[1^R]$^{2-}$** in the complexes **1^R⊂[Fe_4L_6]**(NTf_2)$_6$ and **[Fe_4L_6][1^{tBu}]**(NTf_2)$_6$ in CD_3CN at RT.

cluster	^{1}H NMR Chemical shift ± 0.01 (ppm)
**1:1 H_0-G_0 titration, [H_0] = [G_0] = 5 × 10^{-5} M**	
**1^{Ph}⊂[Fe_4L_6]**(NTf_2)$_6$	7.47, 4.82, 4.24
**1^{OH}⊂[Fe_4L_6]**(NTf_2)$_6$	3.57, 2.59, 1.63

**$[Fe_4L_6][1^{tBu}]$**$(NTf_2)_6$	2.57
**10:1 H_0-G_0 titration, $[H_0] = 5 \times 10^{-4}$ M, $[G_0] = 5 \times 10^{-5}$ M**	
**$1^{Ph}\subset[Fe_4L_6]$**$(NTf_2)_6$	N/A, 4.82, 4.24
**$1^{OH}\subset[Fe_4L_6]$**$(NTf_2)_6$	3.57, 2.59, 1.63
**$[Fe_4L_6][1^{tBu}]$**$(NTf_2)_6$	2.56

Table S10. Summary of association constant, [HG] value and probability of binding of encapsulated cluster **$[1^R]^{2-}$** in the complexes **$1^R\subset[Fe_4L_6]$**$(NTf_2)_6$ and **$[Fe_4L_6][1^{tBu}]$**$(NTf_2)_6$ in CD_3CN at RT.

	**lower bound of K_a (M^{-1})**	**[HG] (M)**	***p***
**$1^{Ph}\subset[Fe_4L_6]$**$(NTf_2)_6$	$>4.8 \times 10^7$	4.89×10^{-5}	0.98
**$1^{OH}\subset[Fe_4L_6]$**$(NTf_2)_6$	$>1.9 \times 10^8$	4.94×10^{-5}	0.99
**$[Fe_4L_6][1^{tBu}]$**$(NTf_2)_6$	-	-	-

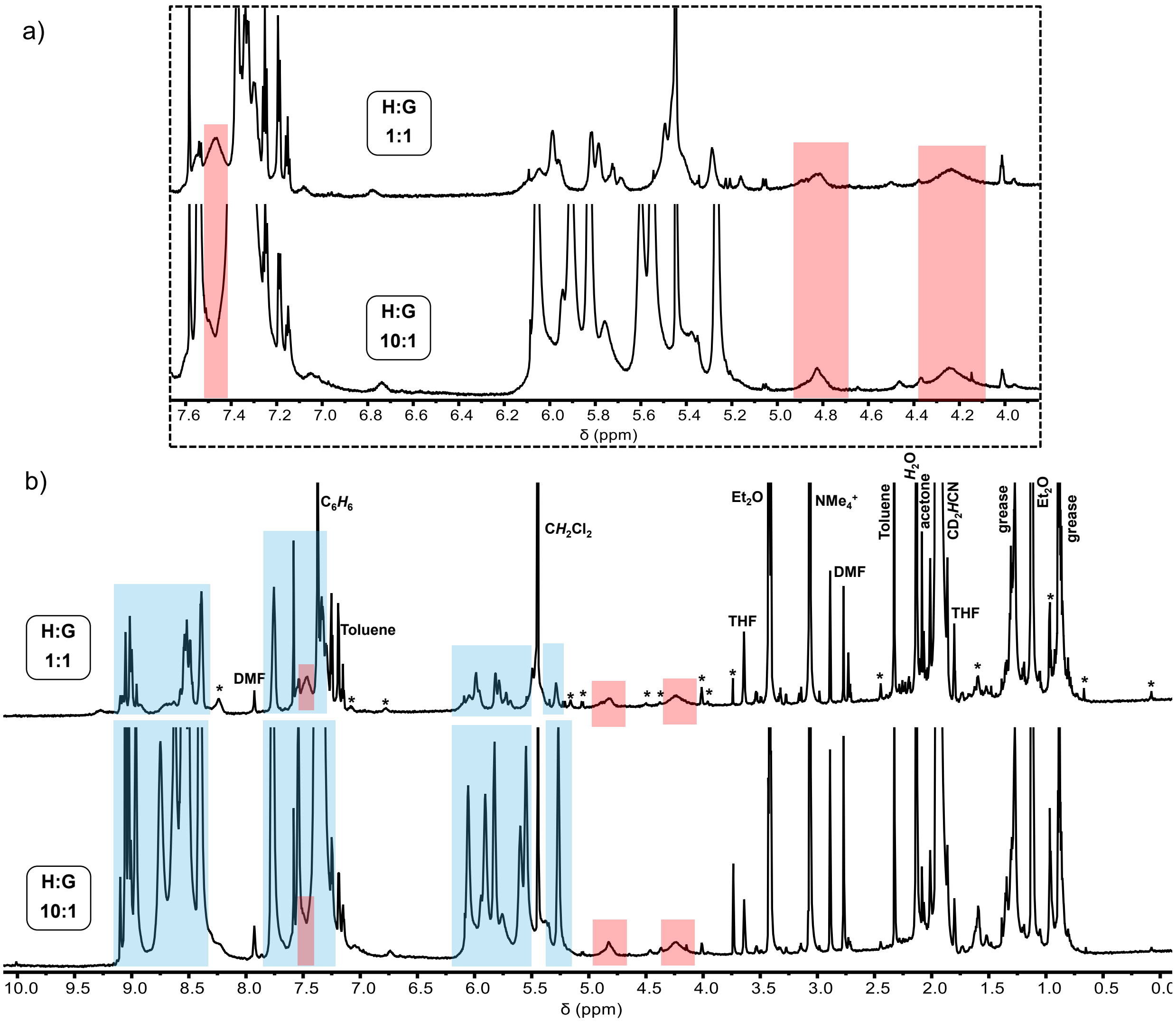

Figure S103. ^{1}H NMR spectra (900 MHz, CD_3CN) of 1:1 and 10:1 mixture of **[Fe_4L_6]**$(NTf_2)_8$ (H) and $(NMe_4)_2$**[1^{Ph}]** (G), respectively. The peaks of encapsulated **[1^{Ph}]**$^{2-}$ are highlighted in pink and the cage is highlighted in blue. a) Magnified view of region 3.9-7.6 ppm to highlight the cluster peaks. b) Full spectrum of region 0-10 ppm. (*unidentified impurities).

a)

b)

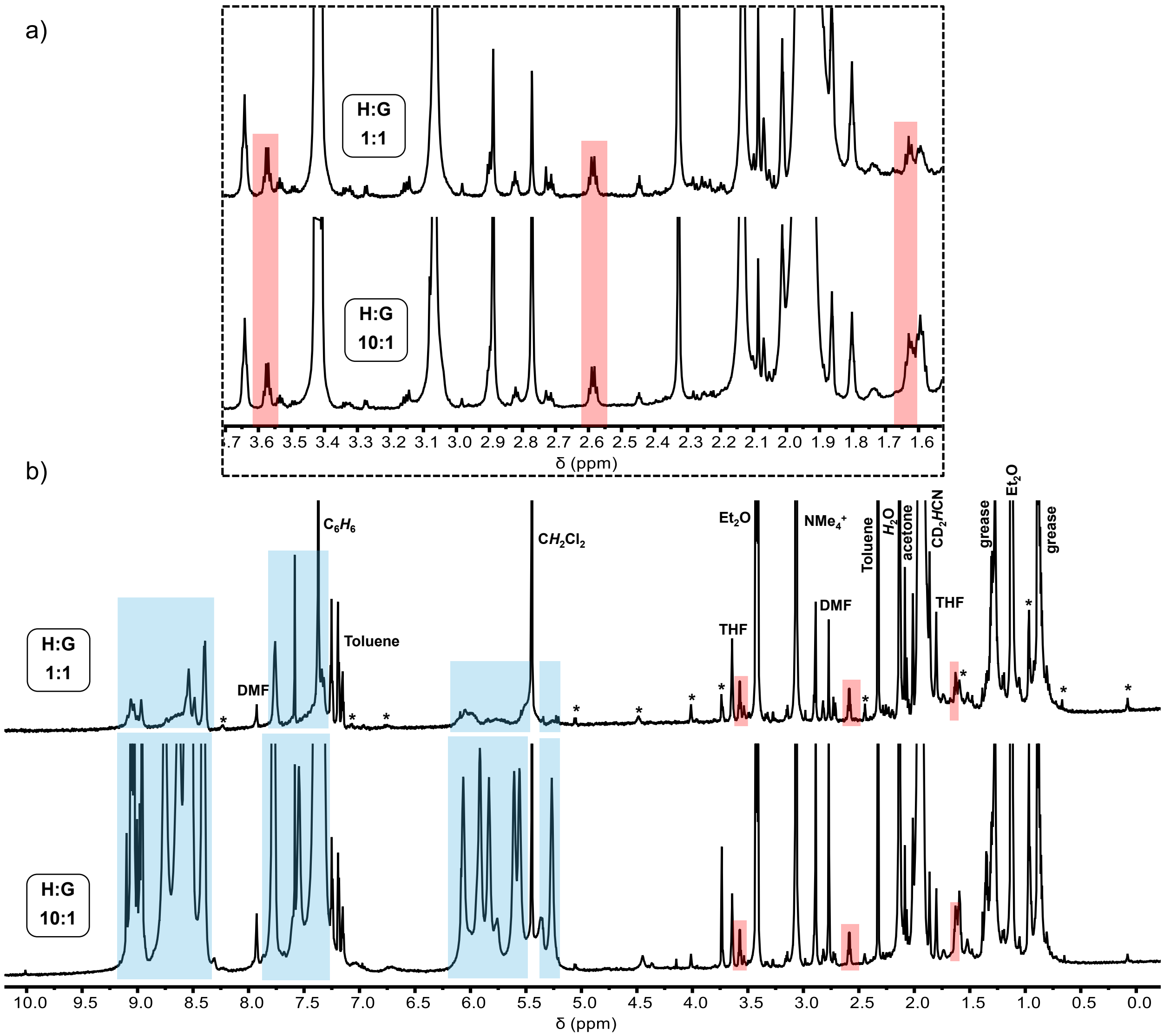

Figure S104. ^{1}H NMR spectra (900 MHz, CD_3CN) of 1:1 and 10:1 mixture of **[Fe$_4$L$_6$]**$(NTf_2)_8$ (H) and $(NMe_4)_2$**[1^{OH}]** (G), respectively. The peaks of encapsulated **[1^{OH}]**$^{2-}$ are highlighted in pink and the cage is highlighted in blue. a) Magnified view of region 1.55-3.70 ppm to highlight the cluster peaks. b) Full spectrum of region 0-10 ppm. (*unidentified impurities).

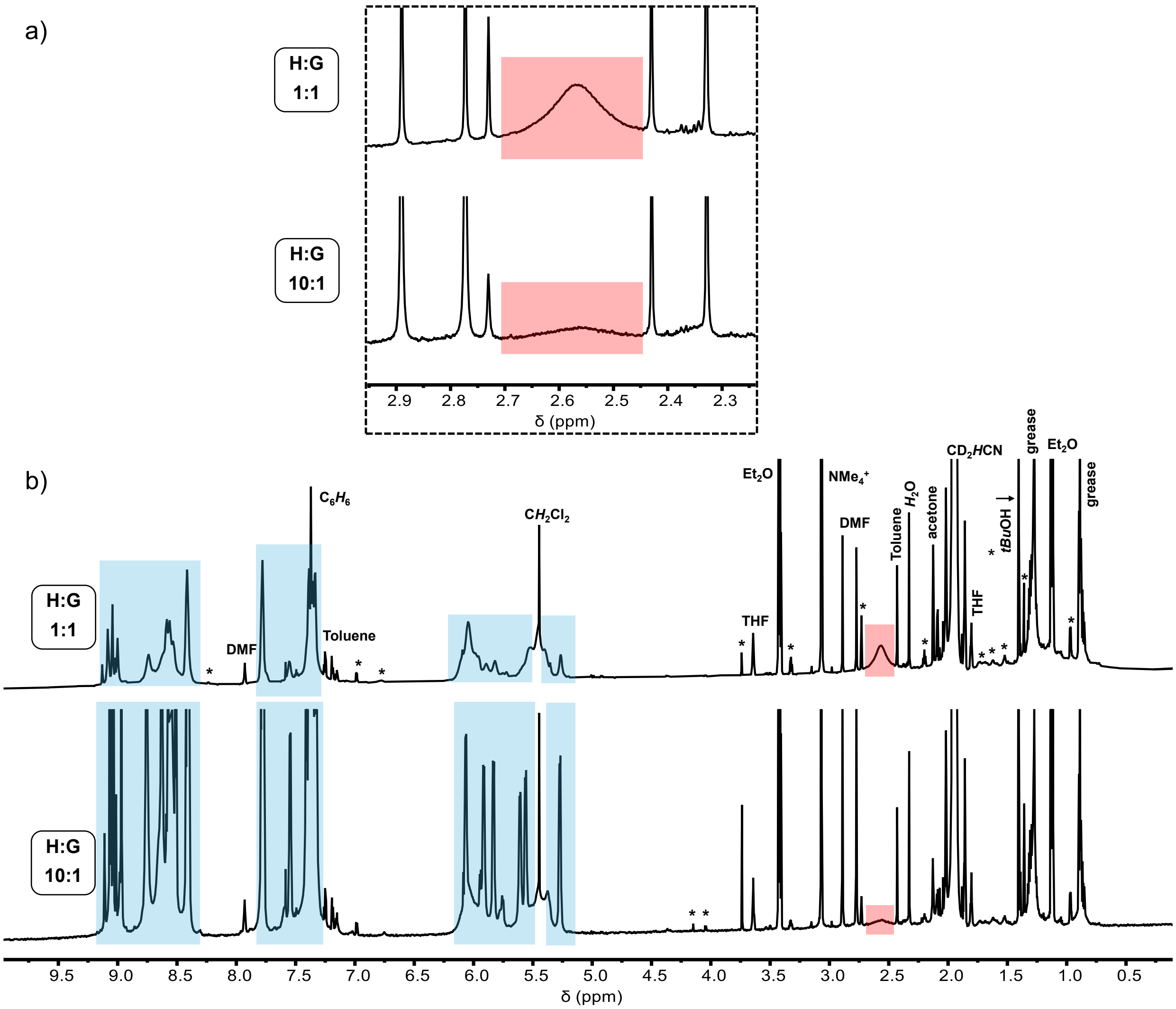

Figure S105. ^{1}H NMR spectra (850 MHz, CD_3CN) of 1:1 and 10:1 mixture of **[Fe$_4$L$_6$]**(NTf_2)$_8$ (H) and (NMe_4)$_2$**[1^{tBu}]** (G), respectively. The peaks of encapsulated **[1^{tBu}]**$^{2-}$ are highlighted in pink and the cage is highlighted in blue. a) Magnified view of region 2.25-2.95 ppm to highlight the cluster peaks. b) Full spectrum of region 0-10 ppm. (*unidentified impurities).

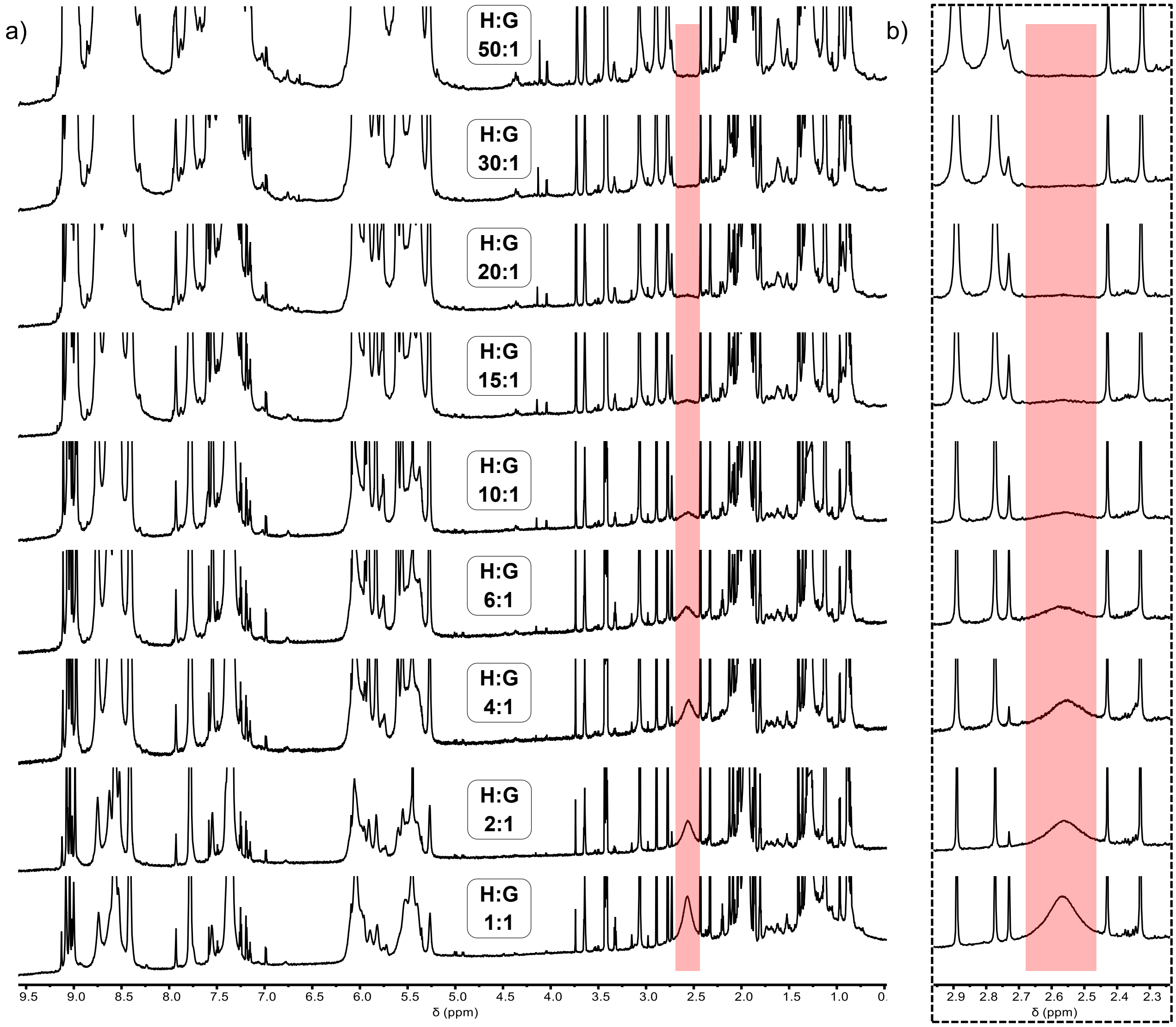

Figure S106. ^{1}H NMR spectra (850 MHz, CD_3CN) of mixtures of **$[Fe_4L_6]$**$(NTf_2)_8$ (H) and $(NMe_4)_2$**$[1^{tBu}]$** (G) at increasing host concentrations, corresponding to host:guest ratios from 1:1 to 50:1. The peaks of encapsulated **$[1^{tBu}]^{2-}$** are highlighted in pink. Progressive broadening of the cluster resonances is observed with increasing host concentration, and these signals disappear beyond a 20:1 host:guest ratio. a) Full spectrum of region 0.5 - 9.5 ppm. b) Magnified view of region 2.25-2.95 ppm highlighting the cluster peaks.

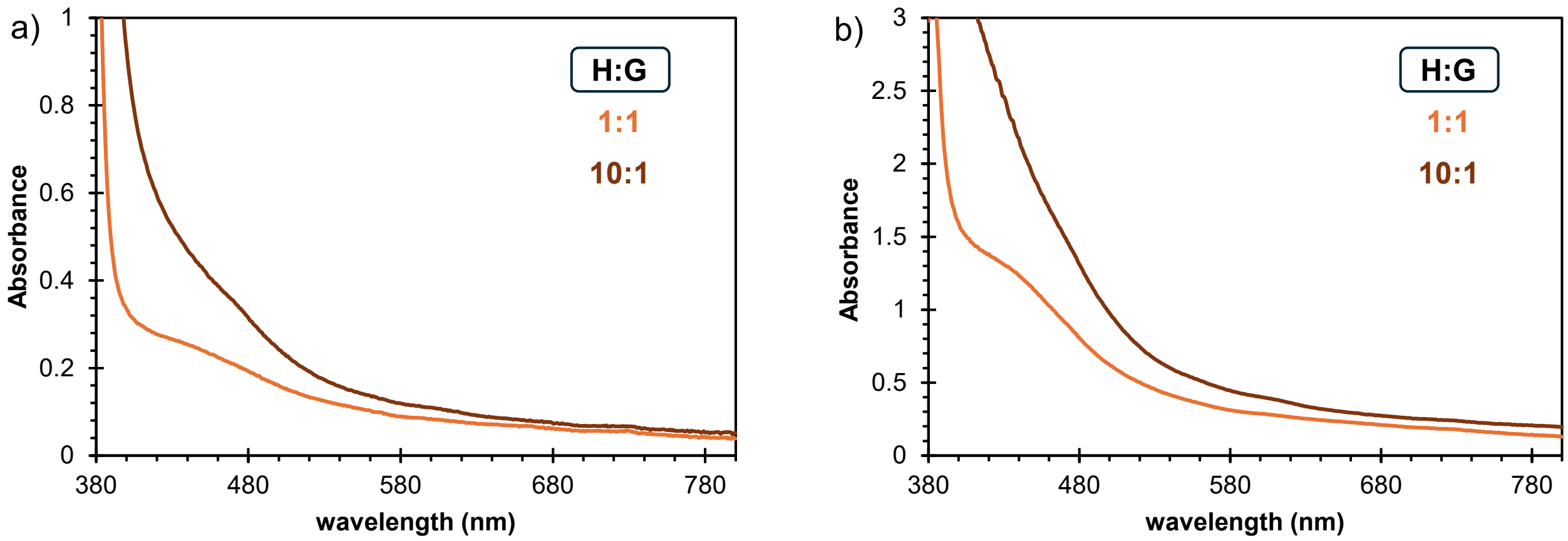

Figure S107. UV-vis absorption spectra of mixtures of **[Ni_4L_6]**(NTf_2)$_8$ (H) and (NMe_4)$_2$**[1^{tBu}]** (G) at host:guest ratios of 1:1 and 10:1. Increasing host concentration leads to obscuring of the **[1^{tBu}]$^{2-}$** absorption band, limiting cluster signal identification beyond a 10:1 host:guest ratio. a) Spectra with [G] = 2.5×10^{-6} M. b) Spectra with [G] = 1.25×10^{-5} M.

S12. COMPUTATIONAL METHOD

Simulations of the $\mathbf{1^{Ph}}\subset[\mathbf{Zn_4L_6}]^{6+}$ and $\mathbf{1^{OH}}\subset[\mathbf{Zn_4L_6}]^{6+}$ assemblies, alongside the hypothetical $[\mathbf{Zn_4L_6}][\mathbf{1^{tBu}}]^{6+}$ complex, were carried out using unrestricted Kohn-Sham density functional theory (DFT) as implemented in ORCA 6.0.1.[28] Geometry optimizations were initiated from the crystallographic coordinates of the $[\mathbf{1^R}]^{2-}$ clusters[7,29,30] and the $[\mathbf{Zn_4L_6}]^{8+}$ framework,[31] utilizing the TPSS[32] functional with the def2-SVP[33] basis set, complemented by Grimme's D3 dispersion correction with Becke-Johnson damping.[34] Subsequent frequency analyses were conducted at the TPSS-D3BJ/MINIX[35] level of theory. All systems were treated as singlets, with initial guesses employed in the UKS procedure to allow for spin-symmetry breaking and capture of strong correlation effects in the electronic structure of the $[Fe_4S_4]$ clusters.[36]

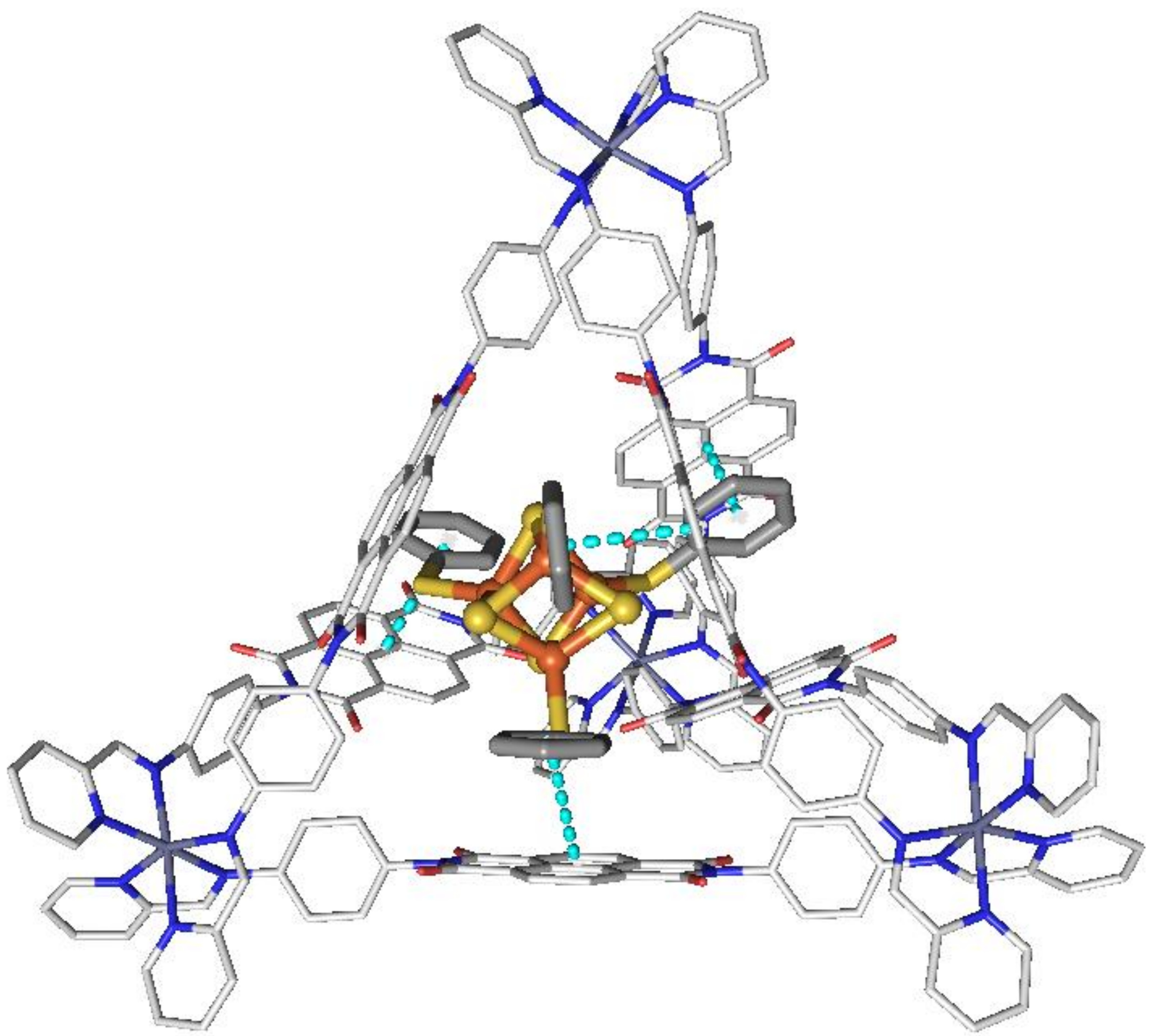

Figure S108. DFT-optimized structure of $\mathbf{1^{Ph}}\subset[\mathbf{Zn_4L_6}]^{6+}$. Level of theory: TPSS/D3BJ/def2-SVP for geometry optimizations and TPSS/D3BJ/MINIX for single-point and frequency calculations. These calculations revealed Gibbs free energies of –7.0 kcal/mol for encapsulation of $[\mathbf{1^{Ph}}]^{2-}$. Cyan dashes represent p-p stacking between -*Ph* groups of cluster and *NDI* panels of cage. Color Scheme: blue, Nitrogen; red, Oxygen; grey, Carbon (dark, cluster; light, cage); yellow, Sulfur; orange, Iron; indigo, Zinc.

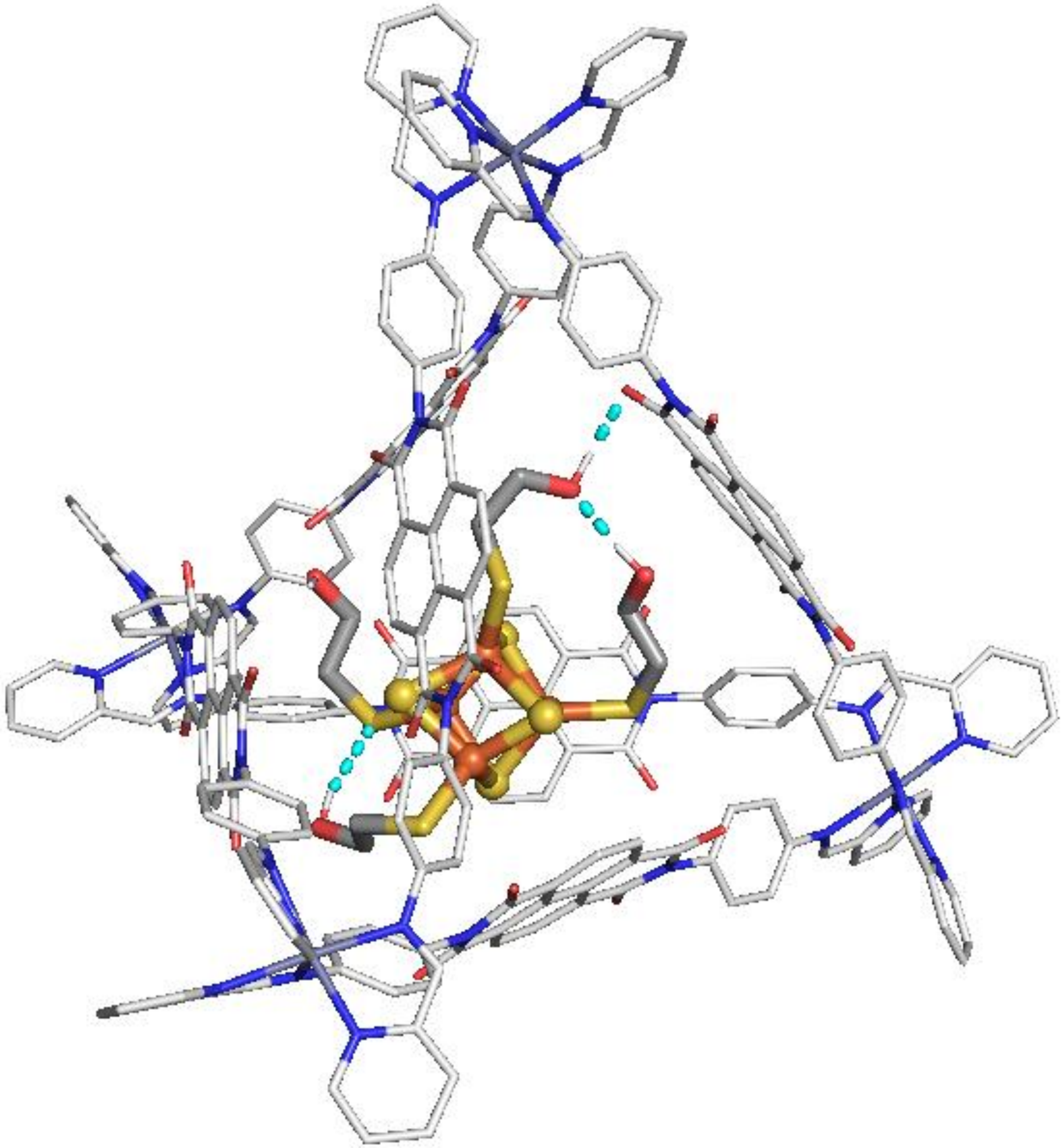

Figure S109. DFT-optimized structure of $\mathbf{1^{OH}{\subset}[Zn_4L_6]^{6+}}$. Level of theory: TPSS/D3BJ/def2-SVP for geometry optimizations and TPSS/D3BJ/MINIX for single-point and frequency calculations. Cyan dashes represent H-bonding between mercaptoethanol groups and mercaptoethanol groups of cluster and *NDI* panels of cage. Color Scheme: blue, Nitrogen; red, Oxygen; grey, Carbon (dark, cluster; light, cage); yellow, Sulfur; orange, Iron; indigo, Zinc.

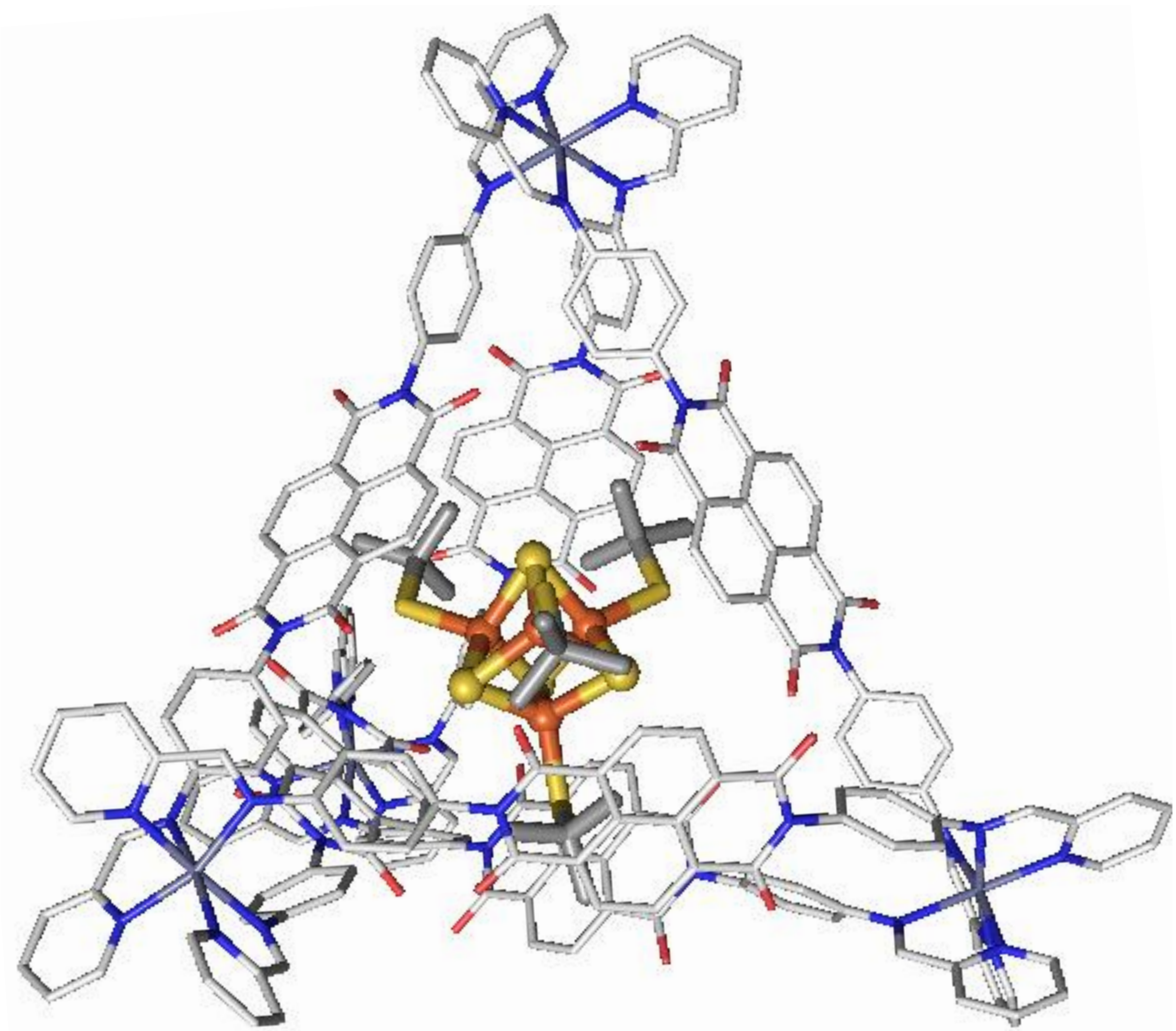

Figure S110. DFT-optimized structure of $\mathbf{Zn_4L_6][1^{tBu}]^{6+}}$. Level of theory: TPSS/D3BJ/def2-SVP for geometry optimizations and TPSS/D3BJ/MINIX for single-point and frequency calculations. These calculations revealed Gibbs free energies of +3.7 kcal/mol for encapsulation of $\mathbf{[1^{tBu}]^{2-}}$. Color Scheme: blue, Nitrogen; red, Oxygen; grey, Carbon (dark, cluster; light, cage); yellow, Sulfur; orange, Iron; indigo, Zinc.

S13. REFERENCES

(1) Roberts, D. A.; Pilgrim, B. S.; Sirvinskaite, G.; Ronson, T. K.; Nitschke, J. R. Covalent Post-Assembly Modification Triggers Multiple Structural Transformations of a Tetrazine-Edged Fe_4L_6 Tetrahedron. *J. Am. Chem. Soc.* **2018**, *140* (30), 9616–9623. https://doi.org/10.1021/jacs.8b05082.

(2) Black, S. P.; Wood, D. M.; Schwarz, F. B.; Ronson, T. K.; Holstein, J. J.; Stefankiewicz, A. R.; Schalley, C. A.; Sanders, Jeremy. K. M.; Nitschke, J. R. Catenation and Encapsulation Induce Distinct Reconstitutions within a Dynamic Library of Mixed-Ligand Zn_4L_6 Cages. *Chem. Sci.* **2016**, *7* (4), 2614–2620. https://doi.org/10.1039/c5sc04906g.

(3) Ronson, T. K.; Roberts, D. A.; Black, S. P.; Nitschke, J. R. Stacking Interactions Drive Selective Self-Assembly and Self-Sorting of Pyrene-Based $M^{II}{}_4L_6$ Architectures. *J. Am. Chem. Soc.* **2015**, *137* (45), 14502–14512. https://doi.org/10.1021/jacs.5b09920.

(4) Maglic, J. B.; Lavendomme, R. *MoloVol*: An Easy-to-Use Program for Analyzing Cavities, Volumes and Surface Areas of Chemical Structures. *J. Appl. Crystallogr.* **2022**, *55* (4), 1033–1044. https://doi.org/10.1107/s1600576722004988.

(5) Que, L.; Bobrik, M. A.; Ibers, J. A.; Holm, R. H. Synthetic Analogs of the Active Sites of Iron-Sulfur Proteins. VII. Ligand Substitution Reactions of the Tetranuclear Clusters $[Fe_4S_4(SR)_4]^{2-}$ and the Structure of $[(CH_3)_4N]_2[Fe_4S_4(SC_6H_5)_4]$. *J. Am. Chem. Soc.* **1974**, *96* (13), 4168–4178. https://doi.org/10.1021/ja00820a018.

(6) Mascharak, P. K.; Hagen, K. S.; Spence, J. T.; Holm, R. H. Structural Distortions of the $[Fe_4S_4]^{2+}$ Core of $[Fe_4S_4(S\text{-}t\text{-}C_4H_9)_4]^{2-}$ in Different Crystalline Environments and Detection and Instability of Oxidized ($[Fe_4S_4]^{3+}$) Clusters. *Inorg. Chim. Acta.* **1983**, *80*, 157–170. https://doi.org/10.1016/s0020-1693(00)91277-5.

(7) Barclay, J. E.; Davies, S. C.; Evans, D. J.; Hughes, D. L.; Longhurst, S. Lattice Effects in the Mössbauer Spectra of Salts of $[Fe_4S_4\{S(CH_2)_nOH\}_4]^{2-}$. Crystal Structures of $[PPh_4]_2[Fe_4S_4\{S(CH_4)_{2n}OH\}_4]$ (N=2, 3 and 4). *Inorg. Chim. Acta.* **1999**, *291* (1–2), 101–108. https://doi.org/10.1016/s0020-1693(99)00098-5.

(8) Horwitz, N. E.; Xie, J.; Filatov, A. S.; Papoular, R. J.; Shepard, W. E.; Zee, D. Z.; Grahn, M. P.; Gilder, C.; Anderson, J. S. Redox-Active 1D Coordination Polymers of Iron–Sulfur Clusters. *J. Am. Chem. Soc.* **2019**, *141* (9), 3940–3951. https://doi.org/10.1021/jacs.8b12339.

(9) Christou, G.; Garner, C. D. A Convenient Synthesis of Tetrakis[Thiolato-μ^3-Sulphido-Iron]$^{(2-)}$ Clusters. *J. Chem. Soc., Dalton Trans.* **1979**, No. 6, 1093–1094. https://doi.org/10.1039/DT9790001093.

(10) Crockett, M. P.; Zhang, H.; Thomas, C. M.; Byers, J. A. Adding Diffusion Ordered NMR Spectroscopy (DOSY) to the Arsenal for Characterizing Paramagnetic Complexes. *Chem. Commun.* **2019**, *55* (96), 14426–14429. https://doi.org/10.1039/C9CC08229H.

(11) Gorman, C. B.; Parkhurst, B. L.; Su, W. Y.; Chen, K.-Y. Encapsulated Electroactive Molecules Based upon an Inorganic Cluster Surrounded by Dendron Ligands. *J. Am. Chem. Soc.* **1997**, *119* (5), 1141–1142. https://doi.org/10.1021/ja963541q.

(12) Fa Bamba, I.; Falaise, C.; Marrot, J.; Atheba, P.; Gbassi, G.; Landy, D.; Shepard, W.; Haouas, M.; Cadot, E. Host-Guest Complexation Between Cyclodextrins and Hybrid Hexavanadates: What Are the Driving Forces? *Chemistry A European J* **2021**, *27* (62), 15516–15527. https://doi.org/10.1002/chem.202102684.

(13) Yang, Y.; Li, H.; Shi, Y.; Wu, Y.; Jing, X.; Duan, C. Modifying the Oxidative Potentials of Imines in a Dye Loaded Capsule for Photocatalytic Cyclization with Hydrogen Evolution. *Angew Chem Int Ed* **2024**, *63* (11). https://doi.org/10.1002/anie.202319605.

(14) Dandliker, P. J.; Diederich, F.; Gross, M.; Knobler, C. B.; Louati, A.; Sanford, E. M. Dendritic Porphyrins: Modulating Redox Potentials of Electroactive Chromophores with Pendant Multifunctionality. *Angew. Chem. Int. Ed. Engl.* **1994**, *33* (17), 1739–1742. https://doi.org/10.1002/anie.199417391.

(15) Richert, S. A.; Tsang, P. K. S.; Sawyer, D. T. Ligand-Centered Redox Processes for Manganese, Iron and Cobalt, MnL_3, FeL_3, and CoL_3, Complexes (L = Acetylacetonate, 8-Quinolinate, Picolinate, 2,2'-Bipyridyl, 1,10-

Phenanthroline) and for Their Tetrakis(2,6-Dichlorophenyl)Porphinato Complexes[M(Por)]. *Inorg. Chem.* **1989**, *28* (12), 2471–2475. https://doi.org/10.1021/ic00311a044.

(16) Kadirov, M. K.; Kholin, K. V.; Tselishcheva, E. Yu.; Burilov, V. A.; Mustafina, A. R. Cyclic Voltammetry of Tris(2,2′-Bipyridine)Zinc(II) Diperchlorate Detected by Electron Spin Resonance. *Russ. Chem. Bull.* **2013**, *62* (6), 1327–1331. https://doi.org/10.1007/s11172-013-0187-x.

(17) Tanaka, N.; Sato, Y. Univalent Tris(2,2′-Bipyridine) Niccolate Anion in Acetonitrile. *Inorg. Nucl. Chem. Lett.* **1968**, *4* (9), 487–490. https://doi.org/10.1016/0020-1650(68)80016-9.

(18) Henne, B. J.; Bartak, D. E. Metal-Vapor Synthesis and Electrochemistry of Bis(Bipyridyl)Nickel(0). *Inorg. Chem.* **1984**, *23* (3), 369–373. https://doi.org/10.1021/ic00171a020.

(19) Lu, Z.; Lavendomme, R.; Burghaus, O.; Nitschke, J. R. A Zn_4L_6 Capsule with Enhanced Catalytic C−C Bond Formation Activity upon C_{60} Binding. *Angew. Chem. Int. Ed.* **2019**, *58* (27), 9073–9077. https://doi.org/10.1002/anie.201903286.

(20) Lu, Z.; Ronson, T. K.; Nitschke, J. R. Reversible Reduction Drives Anion Ejection and C_{60} Binding within an $Fe^{II}{}_4L_6$ Cage. *Chem. Sci.* **2020**, *11* (4), 1097–1101. https://doi.org/10.1039/c9sc05728e.

(21) Venkateswara Rao, P.; Holm, R. H. Synthetic Analogues of the Active Sites of Iron−Sulfur Proteins. *Chem. Rev.* **2004**, *104* (2), 527–560. https://doi.org/10.1021/cr020615+.

(22) Kennepohl, P.; Solomon, E. I. Electronic Structure Contributions to Electron-Transfer Reactivity in Iron−Sulfur Active Sites: 1. Photoelectron Spectroscopic Determination of Electronic Relaxation. *Inorg. Chem.* **2003**, *42* (3), 679–688. https://doi.org/10.1021/ic020330f.

(23) Kennepohl, P.; Solomon, E. I. Electronic Structure Contributions to Electron-Transfer Reactivity in Iron−Sulfur Active Sites: 2. Reduction Potentials. *Inorg. Chem.* **2003**, *42* (3), 689–695. https://doi.org/10.1021/ic0203318.

(24) DePamphilis, B. V.; Averill, B. A.; Herskovitz, T.; Que, L.; Holm, R. H. Synthetic Analogs of the Active Sites of Iron-Sulfur Proteins. VI. Spectral and Redox Characteristics of the Tetranuclear Clusters $[Fe_4S_4(SR)_4]^{2-}$. *J. Am. Chem. Soc.* **1974**, *96* (13), 4159–4167. https://doi.org/10.1021/ja00820a017.

(25) Mahata, K.; Frischmann, P. D.; Würthner, F. Giant Electroactive M_4L_6 Tetrahedral Host Self-Assembled with Fe(II) Vertices and Perylene Bisimide Dye Edges. *J. Am. Chem. Soc.* **2013**, *135* (41), 15656–15661. https://doi.org/10.1021/ja4083039.

(26) Ramírez Lázaro, L.; Sigurvinsson, L. C.; Curtin, N.; Ho, J.; Luis, E. T.; McAdams, D. A.; Gudmundsson, T. A.; Hawes, C. S.; Jacquemin, D.; O'Shea, D. F.; Scanlan, E. M.; Gunnlaugsson, T.; Henwood, A. F. Emissive Triphenylamine Functionalised 1,8-Naphthalimide and Naphthalene Diimide Fluorophores: Aggregation, Computation and Biological Studies. *J. Mater. Chem. B* **2025**, *13* (3), 929–942. https://doi.org/10.1039/D4TB01905A.

(27) Thordarson, P. Determining Association Constants from Titration Experiments in Supramolecular Chemistry. *Chem. Soc. Rev.* **2011**, *40* (3), 1305–1323. https://doi.org/10.1039/C0CS00062K.

(28) Neese, F. Software Update: The ORCA Program System—Version 6.0. *WIREs Comput Mol Sci* **2025**, *15* (2), e70019. https://doi.org/10.1002/wcms.70019.

(29) Que, L. Jr.; Bobrik, M. A.; Ibers, J. A.; Holm, R. H. Synthetic Analogs of the Active Sites of Iron-Sulfur Proteins. VII. Ligand Substitution Reactions of the Tetranuclear Clusters $[Fe_4S_4(SR)_4]^{2-}$ and the Structure of Bis(Tetramethylammonium) [Tetra-.Mu.-Sulfide-Tetrakis(Benzenethiolato)Tetrairon. *J. Am. Chem. Soc.* **1974**, *96* (13), 4168–4178. https://doi.org/10.1021/ja00820a018.

(30) Mascharak, P. K.; Hagen, K. S.; Spence, J. T.; Holm, R. H. Structural Distortions of the $[Fe_4S_4]^{2+}$ Core of $[Fe_4S_4(S\text{-}t\text{-}C_4H_9)_4]^{2-}$ in Different Crystalline Environments and Detection and Instability of Oxidized ($[Fe_4S_4]^{3+}$) Clusters. *Inorg. Chim. Acta* **1983**, *80*, 157–170. https://doi.org/10.1016/s0020-1693(00)91277-5.

(31) Black, S. P.; Wood, D. M.; Schwarz, F. B.; Ronson, T. K.; Holstein, J. J.; Stefankiewicz, A. R.; Schalley, C. A.; Sanders, Jeremy. K. M.; Nitschke, J. R. Catenation and Encapsulation Induce Distinct Reconstitutions within a Dynamic Library of Mixed-Ligand Zn4L6 Cages. *Chem. Sci.* **2016**, *7* (4), 2614–2620. https://doi.org/10.1039/C5SC04906G.

(32) Tao, J.; Perdew, J. P.; Staroverov, V. N.; Scuseria, G. E. Climbing the Density Functional Ladder: Nonempirical Meta–Generalized Gradient Approximation Designed for Molecules and Solids. *Phys. Rev. Lett.* **2003**, *91* (14), 146401. https://doi.org/10.1103/PhysRevLett.91.146401.

(33) Weigend, F.; Ahlrichs, R. Balanced Basis Sets of Split Valence, Triple Zeta Valence and Quadruple Zeta Valence Quality for H to Rn: Design and Assessment of Accuracy. *Phys. Chem. Chem. Phys.* **2005**, *7* (18), 3297. https://doi.org/10.1039/b508541a.

(34) Grimme, S.; Ehrlich, S.; Goerigk, L. Effect of the Damping Function in Dispersion Corrected Density Functional Theory. *J Comput Chem* **2011**, *32* (7), 1456–1465. https://doi.org/10.1002/jcc.21759.

(35) Sure, R.; Grimme, S. Corrected Small Basis Set Hartree-Fock Method for Large Systems. *J Comput Chem* **2013**, *34* (19), 1672–1685. https://doi.org/10.1002/jcc.23317.

(36) Noodleman, Louis.; Case, D. A.; Aizman, Arie. Broken Symmetry Analysis of Spin Coupling in Iron-Sulfur Clusters. *J. Am. Chem. Soc.* **1988**, *110* (4), 1001–1005. https://doi.org/10.1021/ja00212a003.
